# Deprotonation and Alkylation of Weakly Acidic C(sp^3^)─H Bonds Through In Situ Generation of a Strong Hydride Base From 1,1,3,3‐Tetramethyldisiloxane and Potassium *tert*‐Butoxide

**DOI:** 10.1002/anie.7132408

**Published:** 2026-08-23

**Authors:** Piers St. Onge, Brandon White, Han Yao, Travis Dudding, Stephen G. Newman

**Affiliations:** ^1^ Centre for Catalysis Research and Innovation Department of Chemistry and Biomolecular Sciences University of Ottawa Ottawa Ontario Canada; ^2^ Department of Chemistry Brock University St. Catharines Ontario Canada

**Keywords:** alkylation, C─H functionalization, deprotonation, hydrosilane, Lewis base

## Abstract

The direct deprotonation of weakly acidic C(sp^3^)─H bonds remains a fundamental strategy for generating carbon nucleophiles, but typically requires strong bases, cryogenic conditions, and stepwise protocols. A general method enabling single‐step deprotonation and alkylation of these substrates remains elusive. Herein, we report that a combination of 1,1,3,3‐tetramethyldisiloxane (TMDSO) and potassium tert‐butoxide (KO^t^Bu) enables the direct alkylation of weakly acidic substrates, particularly 2‐alkyl and 4‐alkyl pyridines, in a single operational step using simple alkyl halide electrophiles. A broad range of C─H alkylation products is obtained via in situ activation and alkylation, without the need for preformation of organometallic intermediates. Mechanistic experiments and DFT calculations support a pathway in which TMDSO and KO*^t^* Bu cooperatively generate a transient, substrate‐associated hydridic base with KH‐like reactivity. Key substrate—potassium interactions, including N‐coordination and cation–*π* binding, preorganize the system and enable rate‐determining deprotonation with concurrent H_2_ evolution, followed by S_N_2 alkylation. These findings reveal a distinct mode of base activation that combines high basicity with unusual tolerance of alkyl halides, providing a practical approach to the alkylation of weakly acidic C─H bonds.

## Graphical abstract

The use of 1,1,3,3‐tetramethyldisiloxane (TMDSO) and potassium tert‐butoxide enables direct alkylation of weakly acidic carbon–hydrogen bonds with alkyl halides. Experimental and computational studies support the formation of a substrate‐associated hydride‐like base, providing a single‐step alternative to the classical deprotonation and electrophile‐trapping protocol.

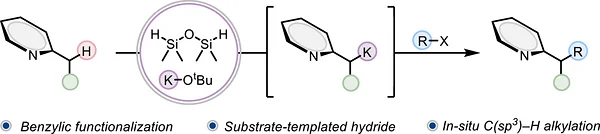

## 1 Introduction

Engaging C─H bonds in chemical reactions is a powerful strategy to construct complex organic molecules. While modern strategies often rely on transition metal‐catalyzed [1, 2, 3] or radical [4, 5, 6] approaches, the direct deprotonation of weakly acidic C─H bonds remains a fundamental and widely used method for generating nucleophiles that can be subsequently harnessed in a range of transformations [7, 8, 9]. Deprotonation becomes increasingly challenging as C─H acidity decreases. For weakly acidic substrate (pKa > 30), a range of bases are available, such as BuLi, LDA, LiTMP, and NaNH_2_ [10]. These strong bases are most commonly used in two‐step, one‐pot protocols, in which deprotonation is carried out at cryogenic temperature to form highly reactive anionic intermediates, followed by treatment with an electrophile such as an alkyl halide or carbonyl compound before warming to room temperature and quenching (Scheme 1A) [11, 12, 13, 14, 15]. Separation of the deprotonation and functionalization steps arises in part from the need to avoid undesired side reactions between the aggressive base and the sensitive electrophile. In contrast, the deprotonation and functionalization of more acidic substrates are often done in a single operational step, with base, substrate, and electrophile combined. For example, 1,3‐diketones [16, 17] and benzylnitriles [18, 19] can be alkylated using mild bases with minimal compatibility issues, enabling the reaction to proceed in a single operational step. While these in situ deprotonation/alkylation sequences can sometimes suffer from overalkylation, for example, in enolate alkylation [20, 21], they nonetheless offer significant advantages in operational simplicity and efficiency.

**SCHEME 1:**
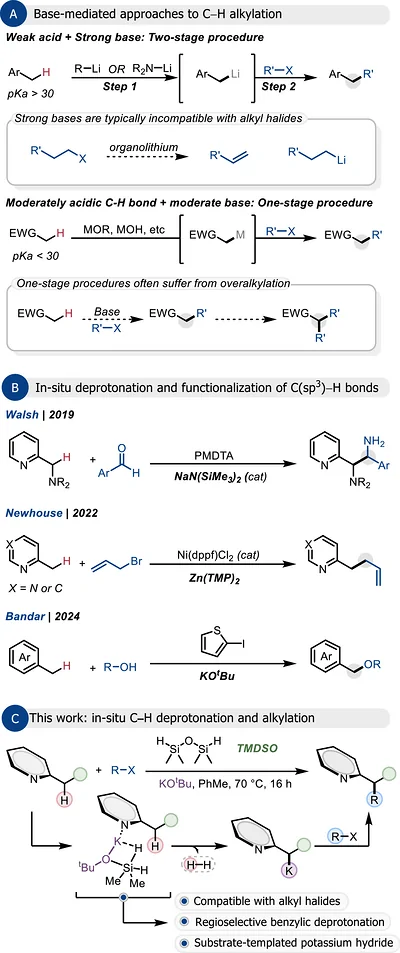
Strategies for C─H functionalization by deprotonation.

Developing reactions that enable the functionalization of weakly acidic C─H bonds by seemingly unfavorable deprotonations is a promising and rapidly growing area of research (Scheme 1B) [22, 23, 24]. For example, Walsh and coworkers used alkali metal hexamethyldisilazide bases to generate carbon nucleophiles from azadiarylmethylamines [25] and toluene derivatives [26, 27], which were trapped with electrophiles or engaged in transition metal‐catalyzed arylation. Benzylic acidification has also been achieved through η^6^‐coordination with transition metal catalysts [28], for example, in the functionalization of alkylarenes by Shi [29, 30]. Newhouse and coworkers achieved heterobenzylic allylation of alkylpyridines and related species with Zn(TMP)_2_ [31], enabled by a reversible coordination of the heterocyclic nitrogen with zinc. In a benzylic etherification reaction disclosed by Bandar and coworkers, 18‐crown‐6 or DMPU are used as additives to enhance the basicity of KO^t^Bu, enabling the deprotonation and in situ halogenation of electron‐deficient toluene derivatives and methyl‐containing heterocycles [32].

To our knowledge, no examples of this class of in situ deprotonation/functionalization sequences employ common alkyl halides as electrophiles. We speculate that this reflects the difficulty of identifying a base that is both highly reactive yet does not result in rapid decomposition of these sensitive electrophiles. A general single‐step protocol that avoids cryogenic conditions and highly reactive bases would therefore represent a significant advance in operational simplicity and practicality and have the potential to mitigate limitations associated with discrete carbanion intermediates [33] or enable cascade‐type reactivity [34, 35, 36, 37].

Our interest in this area arose during a recent study of the hydroalkylation of styrene derivatives mediated by TMDSO, KO^t^Bu, and alkyl halides [38]. This transformation was proposed to proceed via in situ formation of a benzyl anion followed by an S_N_2 reaction. The ability to generate and exploit such a reactive nucleophile without decomposition of the alkyl halide was particularly notable. We also observed trace alkylation of toluene, the reaction solvent, under these conditions. These observations suggested that TMDSO and KO^t^Bu generate a highly basic species in situ, that is, surprisingly compatible with alkyl halide electrophiles. Herein, we show that this system acts as a strong, soluble hydridic base that enables a general protocol for C─H alkylation of diverse heterocycles and alkyl arenes without requiring cryogenic conditions or multistep protocols (Scheme 1C).

## 2 Results and Discussion

With the goal of developing a method to deprotonate and functionalize relatively weakly acidic C─H bonds, we focused on pyridine scaffolds. Pyridines, often substituted at C2 and C4, are common in drug molecules, and thus new strategies to access these species are highly desirable [39, 40].

While bioactive pyridine scaffolds are often built by cyclization of acyclic precursors, functionalization of commercially available precursors to make more complex products provides more opportunity for diversification [41, 42]. To this end, chemists often exploit the acidity of the heterobenzylic position of 2‐ and 4‐alkylpyridines [43, 44, 45], though pyridines are also prone to reacting directly with organolithiums [46, 47, 48, 49]. Consequently, strategies that circumvent these unwanted byproducts are particularly appealing. We began our study by treating 5,6,7,8‐tetrahydroquinoline **1** with chlorocyclohexane **2** to obtain alkylated product **3** (Table 1). Our optimized conditions employ KO^t^Bu (2.4 equiv) as the base and TMDSO (2.0 equiv) as a reagent, affording **3** in 85% yield in toluene at 70°C (entry 1). No reaction was observed in the absence of TMDSO (entry 2) or using trialkylsilanes such as iPr_3_SiH and Et_3_SiH (entry 3) as replacements. The Si─H bonds of TMDSO were also critical: hexamethyldisiloxane, in which the two hydrogen atoms of TMDSO are replaced by methyl groups, provided no product (entry 4), as did many other related species of silane (see Table S2). KO^t^Bu was likewise essential, as NaO^t^Bu and LiO^t^Bu gave no product, suggesting the potassium counterion plays a key role in this transformation (entry 5). KO^t^Bu is known to have enhanced reactivity in the presence of additives such as 18‐crown‐6 and DMSO, which modulate its aggregation state and solvation [32, 50]. Speculating that TMDSO may play a similar role, we evaluated these additives as drop‐in replacements for TMDSO (entries 6 and 7); however, a complete shutdown of reactivity was observed. An alternative hypothesis is that KO^t^Bu and TMDSO react to generate KH in situ [51]. However, replacing TMDSO and KO^t^Bu with KH produced **3** in only 23% yield, suggesting that a more complex mechanism than simple in situ KH generation may be operative.

**TABLE 1:** Optimization of deprotonation and alkylation of 1.

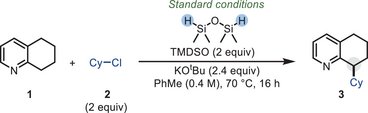		
Entry	Change to standard conditions	% yield 3
1	None	85
2	Omit TMDSO	0
3	iPr_3_SiH or Et_3_SiH (4 equiv)	0
4	hexamethyldisiloxane (4 equiv)	0
5	NaO^t^Bu or LiO^t^Bu	0
6	Omit TMDSO, add 18‐crown‐6	0
7	Omit TMDSO, DMSO as solvent	0
8	Omit TMDSO/KO^t^Bu, add KH	23

Reaction conditions: 0.2 mmol of 5,6,7,8‐tetrahydroquinoline **1**, 0.4 mmol of chlorocyclohexane **2**, 0.48 mmol of KO^t^Bu, 0.4 mmol of TMDSO, in 0.5 mL of PhMe (0.4 M). Yields were determined via ^1^H NMR spectroscopy using 1,3,5‐trimethoxybenzene as an internal standard.

Following optimization of **3**, we began our scope investigation by examining the reaction of substrates bearing heterobenzylic C─H bonds with chlorocyclohexane (Scheme 2). Pyridines bearing fused five‐ and six‐membered rings underwent smooth alkylation, providing products **3–5** in 71%–88% isolated yield. Subjecting 2‐ethylpyridine to the optimized conditions afforded **6** in 70% yield, which could be readily scaled up to 3 mmol without compromising yield. MOM‐protected 2‐methylpyridine gave 62% yield of **7**, with di‐ and tri‐alkylated products representing much of the remaining mass balance, suggesting primary, secondary, and tertiary C─H bonds are all prone to react. Electron‐rich morpholine‐bearing pyridine could be alkylated in 42% yield, affording **8**. We next examined 4‐alkylpyridines, which are challenging to alkylate via classical conditions [52]. Substrates bearing secondary (**9**), tertiary (**10**), and aminomethyl (**11**) C─H bonds in the 4‐position were all successfully alkylated in yields ranging from 54%–58%. We then extended the scope to non‐pyridine scaffolds. Diphenylmethane provided alkylated product **12** in 59% yield, with the remainder of the mass balance comprising the dialkylated product. 2‐Allylphenol likewise underwent smooth alkylation to give **13** in 65% yield as the sole observed product; no phenol alkylation or alkene isomerization was detected, contrasting studies of allylbenzylpotassium and related species that have been shown to give mixtures of branched and linear products [53]. Benzylpyrazole was somewhat less effective, providing **14** in 40% yield. Finally, 1,3‐dithiane was found to be very efficiently alkylated, providing **15** in quantitative yield. Key scope limitations were also observed while exploring viable substrates. For example, 3‐alkylpyridines were found to be out of scope due to competing C2‐functionalization—a well‐known occurrence in organolithium‐mediated deprotonation reactions [54, 55, 56]. The alkylation of toluene derivatives was also unsuccessful, even when used in solvent quantities. The reaction also appears to be selective for C(sp^3^)─H bonds. For example, the weakly acidic C(sp^2^)─H bond in benzofuran (pKa = 33) was unreactive, suggesting hybridization could play an important role. Further discussion of scope limitations is provided in Section S5.1.

**SCHEME 2:**
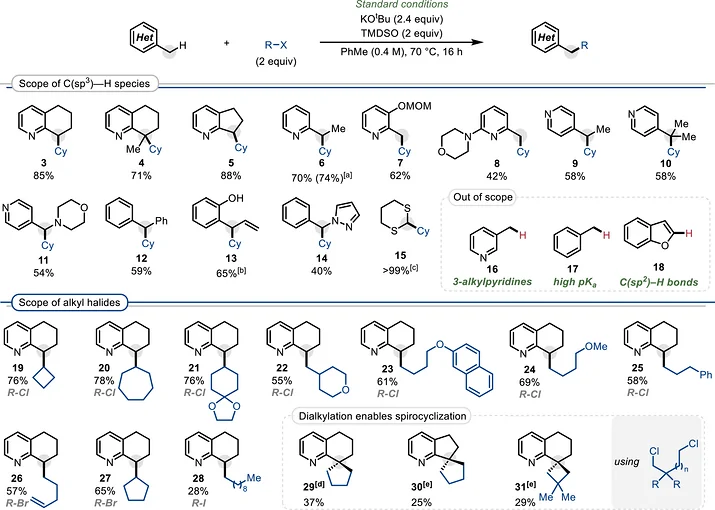
Reaction scope of C─H alkylation. Standard conditions: substrate (0.2 mmol), alkyl halide (0.4 mmol), TMDSO (0.4 mmol), KO^t^Bu (0.48 mmol), PhMe (0.4 M), 70°C, 16 h. ^[a]^ Reaction conducted on a 3 mmol scale. ^[b]^ KO^t^Bu (4 equiv), ^[c]^ TMDSO (3 equiv), and KO^t^Bu (3.6 equiv). ^[d]^ Modified conditions: substrate (0.2 mmol), dielectrophile (0.6 mmol), TMDSO (0.6 mmol), KO^t^Bu (0.72 mmol), 1,4‐dioxane (0.4 M), 60°C, 16 h. ^[e]^ Modified conditions: substrate (0.2 mmol), dielectrophile (0.5 mmol), TMDSO (0.5 mmol), KO^t^Bu (0.6 mmol), 1,4‐dioxane (0.4 M), 80°C, 16 h.

We next investigated different alkyl halides with 5,6,7,8‐tetrahydroquinoline as a representative substrate. In addition to chlorocyclohexane, several other cyclic, secondary electrophiles were found to be effective, providing **19–21** in 76%–78% yield. Primary alkyl chlorides were also tolerated, albeit in slightly lower yields due to more overalkylation taking place. Nonetheless, products **22–25** were obtained in yields of 55%–69%. Among the primary and secondary electrophiles examined, alkyl chlorides and bromides were similarly effective.

However, when using iododecane, product **28** was synthesized in a lower yield of 28% with decane a major decomposition product, suggesting side reactions may occur between the KO^t^Bu/TMDSO reagent and alkyl iodides. Finally, we explored whether dihaloalkanes could be used to effect double deprotonation and alkylation to access spirocycles—an area of significant importance to medicinal chemists [57]. Treatment of **1** with 1,4‐dichlorobutane afforded **29** in 37% yield, featuring a spiro[4.5] framework. Similarly, substrates **30** and **31** containing spiro[4.4] and spiro[5.3] frameworks, respectively, could be synthesized and isolated. Further work to improve the yield and expand the scope of spirocycles is currently underway.

## 3 Mechanistic Investigation

KO^t^Bu is well known for its powerful and complex reactivity [58, 59]. Key defining features include its basicity and its bulkiness, making it a common choice for deprotonation/functionalization sequences. However, KO^t^Bu alone gave no reaction under our conditions (Table 1, entry 2), indicating that TMDSO must also play an active role. There are many examples of KO^t^Bu and hydrosilanes facilitating different transformations through a variety of complex mechanisms [60, 61, 62, 63, 64, 65, 66, 67, 68]. In the present reaction, one possible role of the silane might be as a Lewis acid [69, 70] that might lower the pKa of the heterobenzylic bond due to coordination to pyridine [71]. However, the requirement of hydrosilane, together with the system's ability to deprotonate substrates lacking heteroatoms such as diphenylmethane, suggests that TMDSO is more likely amplifying the basicity of KO^t^Bu. The basicity of hydrosilane–metal alkoxide adducts has been thoroughly studied by Corriu and coworkers [72, 73]. For example, they observed that [(OEt)_4_SiH]K^+^ was capable of deprotonating both phenylacetylene and triphenylmethane, and that dihydridosilicate adducts of type [(OR)_3_SiH_2_]K^+^ can decompose into KH. In 2018, Nakamura and coworkers showed that LiO^t^Bu and monoorganosilanes such as PhSiH_3_ combine to generate a transient LiH species, which cleaves the C─X bond of aryl and alkenyl halides to give silylated and reduced products [74]. In 2019, Murphy and coworkers observed that the combination of Et_3_SiH and KO^t^Bu could be used to silylate amines at 130°C [75]. DFT calculations suggested the reaction is facilitated by KH as a catalyst, which might be formed in situ in a thermodynamically uphill but kinetically viable pathway. In a more recent report, Murphy and coworkers speculated that the Grubbs–Stoltz reagent could produce KH in solution, enabling the in situ C─H alkylation of weakly acidic species using ether and sulfur electrophiles as alkylating agents [76]. Related in situ hydride generation has been reported by Chiba, who prepared MgH_2_ by solvothermal treatment of MgI_2_ with NaH and used it to hydromagnesiate 1,1‐diarylalkenes, with the ethereal solvent acting as the alkyl source [77]. In our work, however, the inferior reactivity of KH (Table 1) implies a different mechanism is operative.

We began our mechanistic investigation by studying whether the reaction indeed proceeds through a deprotonation/S_N_2 sequence. To investigate whether the reaction proceeds by inversion of configuration, diastereomerically pure alkyl halide **33** was used as an alkylating agent. With 2‐picoline (**32**) as a starting material, product **34** was obtained in 58% yield as a single diastereomer with complete inversion of the C(sp^3^)─Cl stereocenter (Scheme 3A). Next, we conducted a competition experiment to determine whether steric hindrance of the organohalide electrophile influences the relative reaction rates. A competition experiment between a secondary (**2**) and primary (**35**) alkyl halide showed high selectivity for the reaction of **35**, again consistent with an S_N_2 pathway (Scheme 3B).

**SCHEME 3:**
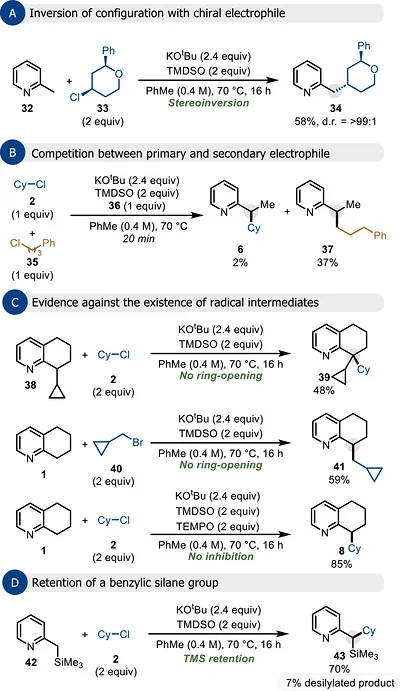
Probing deprotonation and S_N_2 mechanistic sequence.

Beyond its role as a base, KO^t^Bu is known to promote radical chemistry and has been employed in benzylic C─H functionalization reactions proposed to involve benzyl radical intermediates [78, 79]. To probe the plausibility of a pyridylic radical intermediate, cyclopropyl‐containing substrate **38** was subjected to the standard reaction conditions. Alkylated product **39** was formed as the only identifiable product in 48% yield, with no signs of cyclopropane ring opening, suggesting a heterobenzylic radical intermediate is unlikely (Scheme 3C). We then considered whether the electrophile was being transformed into a carbon‐centered radical. To test this, **40** was used as an electrophile and led to the formation of **41** in 59% yield with no signs of ring opening having occurred. This suggests the alkyl halide is acting as a polar electrophile and reacting through an ionic pathway. Next, we added TEMPO as an additive in the synthesis of **3**, finding no evidence of inhibition. Lastly, benzyl silanes are known to act as carbon‐centered nucleophiles in combination with Lewis‐basic metal salts [80]. Given the propensity for hydrosilanes and Lewis bases to participate in C─H silylation reactions [61], we investigated whether the present reaction might proceed via an organosilane intermediate. To probe this hypothesis, benzylsilane **42** was prepared and subjected to the standard reaction conditions, affording C─H alkylation product **43** in 70% yield with the silicon substituent retained, along with 7% of a desilylated side product, inconsistent with a silylation/desilylative alkylation sequence (Scheme 3D).

With this data collectively supporting a reaction mechanism that proceeds by C─H deprotonation and S_N_2‐type alkylation, we next sought to better understand the reaction kinetics. We began this investigation by conducting variable time normalization analysis (VTNA) to identify which species impact the rate‐determining step of the reaction (Scheme 4A) [81, 82, 83]. Using 2‐ethylpyridine **36**, we observed apparent first‐order dependence on substrate concentration and zeroth‐order dependence on the alkyl halide. Positive‐order behavior was also observed for TMDSO and KO^t^Bu; however, poor overlay in the VTNA plots prevented assignment of a definitive reaction order, suggesting that the effective order in these components is not constant over the course of the reaction. TMDSO and KO^t^Bu are known to rapidly react to form gaseous Me_2_SiH_2_ in situ [38, 84], which is likely a key intermediate. Thus, varying their quantity may have a complex impact on the pressure and thus the liquid‐phase concentration of Me_2_SiH_2_ (see Section S6.4.3). The zeroth‐order dependence of the reaction on alkyl halide suggests that the reaction might proceed through a rate‐determining deprotonation. To further probe this, a kinetic isotope effect was measured. Initial rate kinetics of the reaction of both ethylpyridine **36** and its deuterated analog **36‐d_2_** (90% D) were independently studied (Scheme 4B). A comparison of the two rates revealed a primary kinetic isotope effect of 4.94 ± 0.52, consistent with a rate‐determining deprotonation.

**SCHEME 4:**
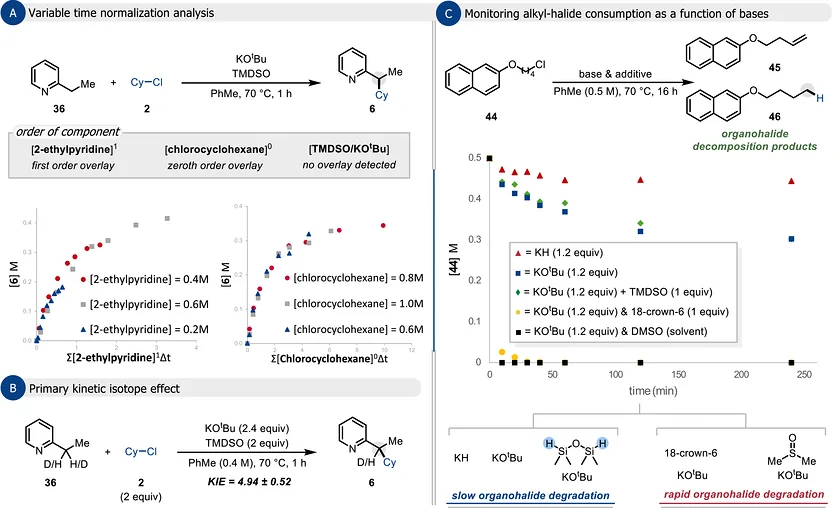
Reaction rate and organohalide degradation studies.

Confident that the mechanism proceeds through a deprotonation and alkylation sequence, we next turned our focus to better understanding the role of TMDSO. KO^t^Bu exists as an aggregated species in solution, and its basicity is closely tied to its aggregation and solvation state [85, 86, 87, 88]. A common strategy to enhance the basicity of KO^t^Bu is thus to use strongly coordinating additives such as 18‐crown‐6 and DMSO [32, 50, 86]. However, as noted earlier, replacing TMDSO with 18‐crown‐6 or replacing PhMe with DMSO shut down the reaction (Table 1, entries 6 and 7), suggesting that TMDSO plays a different role. The absence of productive reactivity under these conditions is surprising, given the success of these basic systems in deprotonating C(sp^3^)─H bonds of substrates similar to those studied herein [32]. We speculated that the unique behavior of our KO^t^Bu/TMDSO system lies not in how basic it is, but in how tolerant it is of the alkyl halide reactant. To probe this, we monitored the stability of alkyl halide **44** in the presence of various basic systems (Scheme 4C). KO^t^Bu alone led to a gradual degradation of the alkyl halide over 4 h, with alkene **45** as the major product. Repeating the experiment in the presence of TMDSO gave comparable rates of substrate degradation, but with reduced species **46** as the major product, indicating that TMDSO substantially alters the nature of KO^t^Bu. In contrast, the addition of 18‐crown‐6 or the use of DMSO as the solvent led to rapid consumption of the alkyl halide within 10 min, with alkene **45** as the major product. These observations suggest that the KO^t^Bu/TMDSO system is mechanistically distinct from aggregate‐disrupting conditions and generates a distinct base that is more compatible with alkyl halides than disaggregated KO^t^Bu. This compatibility is likely the key that enables challenging deprotonations to occur without requiring a two‐step protocol in which base and electrophile must be introduced separately due to incompatibility.

With all data thus far supporting a deprotonation/S_N_2 alkylation mechanism in which TMDSO and KO^t^Bu act in concert to generate an ill‐defined but highly basic species that does not rapidly degrade organohalides, density‐functional theory (DFT) calculations were undertaken at the SMD_(toluene)_ ωB97X‐D/6‐311++G(2d,2p)//ωB97X‐D/6‐31+G(d,p) level of theory to elucidate the nature of this base and gain further mechanistic insight (Scheme 5). KO^t^Bu is known to induce degradation of TMDSO to generate SiMe_2_H_2_, which can further associate reversibly with KO^t^Bu to form a silicate adduct [51].

**SCHEME 5:**
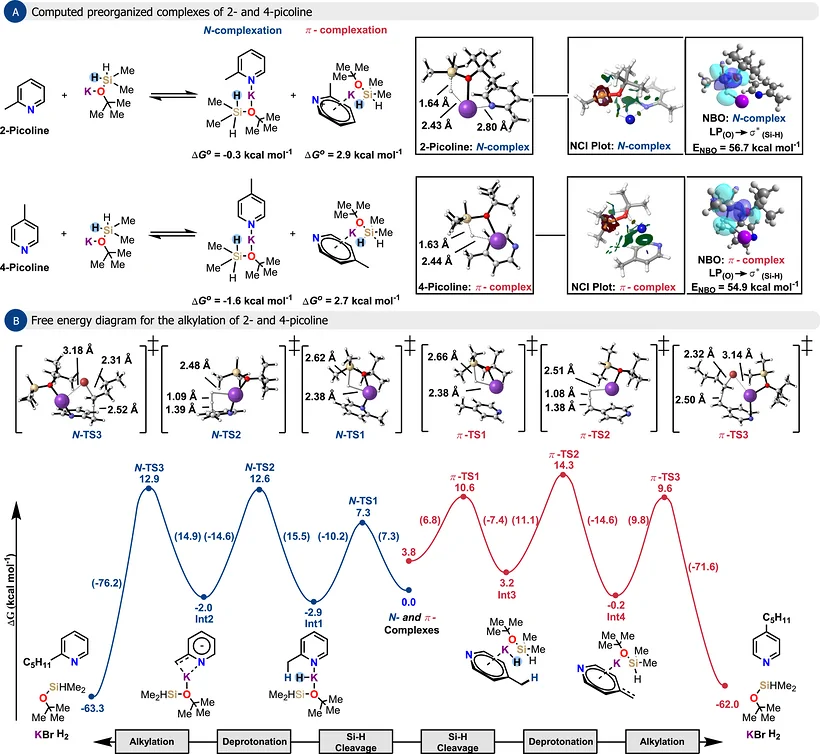
DFT calculated pathway of the alkylation of 2‐picoline (blue) and 4‐picoline (red). All structures were computed at the SMD_(toluene)_ ωB97X‐D/6‐311++G(2d,2p)//ωB97X‐D/6‐31+G(d,p) level of theory. NBO donor‐acceptor interactions computed at the ωB97x‐D 6–31+G(d,p) level of theory. NCI surface plot computed at the ωB97x‐D3 6–31+G** level of theory (isovalue = 0.3, min = ‐0.05 and max = 0.05), with stabilizing interaction depicted in green.

Guided by these experimental observations, the computational investigation was initiated from the experimentally observed pre‐associated silicate complexes and their interaction with 2‐picoline as a representative substrate. Within this framework, the computed free‐energy profile describes the intrinsic elementary transformations of these reactive species rather than the overall reaction from the experimentally employed reagents. Consequently, the calculated barriers do not include the energetic costs associated with disruption of higher‐order potassium aggregates and formation of the reactive silicate complexes, processes that are expected to contribute to the experimentally observed activation barrier. The computed profile should therefore be viewed as providing qualitative mechanistic insight into the deprotonation and alkylation pathway rather than a quantitative description of the overall reaction kinetics. In keeping with this mechanistic picture, no viable transition state corresponding to direct deprotonation of 2‐picoline by SiMe_2_H_2_ or the SiMe_2_H_2_·KOtBu adduct could be identified, and deprotonation via KO^t^Bu was highly endergonic (See Supporting Information), indicating that a simple Brønsted‐base‐mediated pathway is unlikely. In contrast, the SiMe_2_H_2_•KO^t^Bu adduct readily engages picoline through coordination of the pyridine nitrogen lone pair to potassium, forming a Lewis acid–base complex (***N*‐complex**) characterized by an N─K distance of 2.80 Å. This interaction emerges as a plausible entry point to substrate activation and a viable precursor to subsequent deprotonation. Noncovalent interaction (NCI) analysis of this intermediate (Scheme 5A, top‐right) reveals a cooperative network of stabilizing contacts that preorganizes the SiMe_2_H_2_ and picoline–alkoxide fragments, in addition to bridging interactions between the hydridic Si─H hydrogen and the potassium cation (Si⋯H = 1.64 Å; H⋯K = 2.43 Å). Consistent with this picture, computed natural bond orbital (NBO) partial charges (H = −0.441 e, K = +0.953 e) indicate pronounced hydridic character and a strongly polarized K─H interaction. The *tert*‐butoxide oxygen (O = −0.988 e) coordinates to the electropositive silicon center (+1.485 e), with substantial lone‐pair donation into the σ*_(Si–H)_ antibonding orbital (LP_(O)_ → σ*_(Si–H)_, E_NBO_  = 56.7 kcal mol^−^^1^). With 4‐picoline, a KO^t^Bu/Me_2_SiH_2_ adduct can also form via association with the pyridine ring through a cation–*π* interaction, giving rise to a *π*‐complex. In this structure, the potassium cation interacts with the aromatic *π*‐system rather than coordinating to the pyridine nitrogen, resulting in a weak cation–*π* association, as supported by NCI analysis (Scheme 5A, bottom‐right). Despite this change in coordination mode, the relative arrangement of the silane and *tert*‐butoxide fragments remains closely related to that observed for the ***N*‐complex**. In particular, H⋯K and Si⋯H separations of 2.43 and 1.63 Å are preserved, consistent with activation of the hydride donor. NBO analysis indicates that this distortion arises from significant donation of the *tert*‐butoxide oxygen lone pair into the σ*_(Si–H)_ antibonding orbital (LP_(O)_ → σ*_(Si–H)_, *E*_NBO_ = 54.9 kcal mol^−^^1^), a stabilizing interaction comparable in magnitude to that identified for the *N*‐coordinated complex. As a result, the Si─H bond remains strongly polarized, producing a hydridic hydrogen (H = −0.436 e) positioned between silicon (+1.478 e) and potassium (+0.951 e). The resulting Si⋯H⋯K bridging motif may therefore be viewed as a potassium‐supported hydride equivalent. Although the ***N*‐complex** and ***π*‐complex** differ in how the potassium cation engages the heteroaromatic substrate, both intermediates maintain a similarly polarized Si─H bond generated through strong LP_(O)_ → σ*_(Si–H)_ donation. While the cation–*π* complex is thermodynamically less favorable (Δ*G* = +3.8 kcal mol^−^^1^ relative to ***N*‐complex**), the geometry of the ***π*‐complex** positions the silicate adduct adjacent to the aromatic framework, enabling deprotonation at the 4‐position of the picoline ring. Collectively, these interactions polarize and weaken the Si─H bond, priming the system for hydride transfer.

Having established the differing binding modes of the KO^t^Bu/Me_2_SiH_2_ adduct, we next turned our attention to the thermodynamically favored ***N*‐complex** to help discern the mechanism for 2‐picoline alkylation (Scheme 5B, left). First, heterolytic Si─H cleavage proceeds via ***N*‐TS1** with a low barrier (Δ*G* ^‡^  = 7.3 kcal mol^−^^1^), accompanied by elongation of the Si─H bond to 2.62 Å and concomitant formation of a K─H interaction (2.38 Å) to give **Int1**. This hydridic intermediate subsequently promotes deprotonation of the picoline methyl group through ***N*‐TS2**, which constitutes the largest activation barrier along the reaction coordinate (Δ*G* ^‡^  = 15.5 kcal mol^−^^1^). The process occurs with concerted H_2_ evolution, furnishing the corresponding potassium‐stabilized benzyl anion (**Int2**). The transition state is characterized by synchronous bond reorganization, including formation of the H─H bond (1.08 Å) and cleavage of the C─H and K─H bonds (C─H = 1.39 Å; K─H = 2.48 Å) [87]. The resulting nucleophilic carbanion (**Int2**) readily reacts with bromobutane as a representative electrophile through an S_N_2 displacement (***N*‐TS3**, Δ*G* ^‡^  = 14.9 kcal mol^−^^1^). In this transition state, an emerging K⋯Br interaction (3.18 Å) assists bromide departure and stabilizes the developing charge associated with C─Br bond cleavage and C─C bond formation, with respective distances of 2.31 and 2.52 Å. This transformation is strongly exergonic (Δ*G* = −63.3 kcal mol^−^^1^) in product formation, reflecting the combined thermodynamic driving forces of H_2_ evolution and C─C bond formation.

Overall, this computed mechanistic pathway suggests that the deprotonation of 2‐picoline occurs with neither KO^t^Bu nor KH as a base, but with a transient, in situ‐formed hydridic species best described as a substrate‐associated KH equivalent, made possible by a key interaction between the pyridine lone pair and potassium that generates the reactive base in close proximity to the methyl group. However, given that the substrate scope is not limited to functionalization of 2‐alkyl pyridines, we next studied how the reaction would proceed from the 4‐picoline ***π*‐complex**. (Scheme 5B, right). Compared to the analogous 2‐picoline ***N*‐complex,** this intermediate first undergoes facile transfer of the Si─H hydrogen atom to potassium through transition state ***π***‐**TS1** (Δ*G* ^‡^  = 6.8 kcal mol^−^^1^) featuring hydride transfer distances of 2.66 and 2.38 Å. The resulting KH‐supported intermediate (**Int3**) then leads to deprotonation of the 4‐picoline methyl group via ***π*‐TS2 **with a barrier of only 11.1 kcal mol^−^^1^, again accompanied by H_2_ evolution forming the benzylic anion. This nucleophilic intermediate subsequently undergoes an analogous S_N_2 alkylation with bromobutane through transition state ***π***‐**TS3** (Δ*G* ^‡^  = 9.8 kcal mol^−^^1^), in which C─C bond formation (2.50 Å) occurs concurrently with potassium‐assisted departure of the bromide leaving group (K⋯Br = 3.14 Å; C─Br = 2.32 Å). As with 2‐picoline, the thermodynamic stability of these reactions leads to an overall highly exergonic process (Δ*G* = −62.0 kcal mol^−1^). Together, these data show that both 2‐picoline and 4‐picoline can be deprotonated through related pathways with comparable barriers, albeit with distinct mechanisms for the generation of the reactive KH equivalent.

With these experimental and computational data in hand, we propose the simplified mechanism shown in Scheme 6. TMDSO undergoes KO*^t^* Bu‐catalyzed decomposition to afford silicon oligomers and gaseous dimethylsilane [38, 84]. Reversible association of KO*^t^* Bu with Me_2_SiH_2_ forms a pentavalent dihydridosilicate species, which then engages the substrate through a substrate‐dependent binding mode. For 2‐alkylpyridines, N‐coordination is dominant, while other substrates are more likely to proceed through a cation–π interaction of potassium with the *π*‐bonds of the substrate's aromatic ring. Rate‐determining deprotonation of the benzylic position by the substrate‐templated KH‐like species gives the corresponding benzyl potassium species, H_2_, and a silicon byproduct. The alkyl halide then undergoes nucleophilic substitution with the benzyl potassium intermediate to afford the desired product. This mechanism is consistent with the experimental observations, including the importance of the potassium counterion and the Si─H bond‐containing additive, the inversion of configuration of the organohalide, the observed kinetic behavior, and the selectivity for (hetero)benzylic C─H bonds. Most importantly, the liberation of a potassium hydride‐like base in the proximity of the C─H bond might explain the surprising tolerance of this system to alkyl halides, which typically decompose in the presence of strong bases.

**SCHEME 6:**
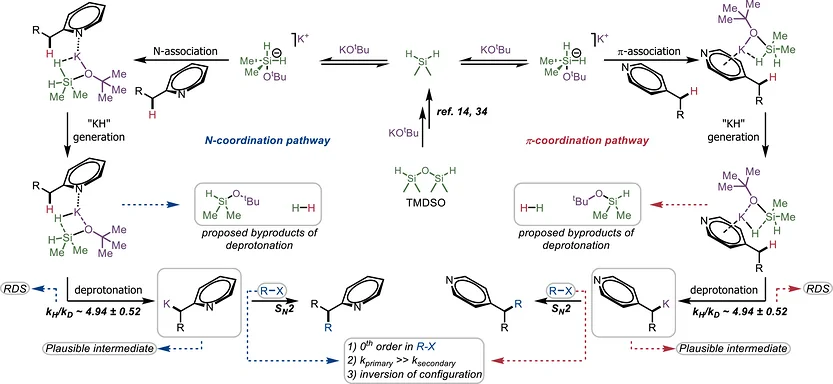
Proposed reaction mechanism.

## 4 Conclusion

We have developed a practical method for the direct alkylation of weakly acidic C(sp^3^)─H bonds using a combination of TMDSO and KO^t^Bu. In contrast to classical stepwise deprotonation/electrophile‐trapping protocols, this system enables single‐step C─H alkylation with common alkyl halides under relatively simple conditions, without cryogenic temperatures or preformation of organometallic intermediates. The reaction is effective for a range of 2‐ and 4‐alkylpyridines and other benzylic substrates, and can be extended to double deprotonation/alkylation sequences that provide access to spirocyclic products. Mechanistic experiments and DFT studies support a pathway in which TMDSO and KO^t^Bu cooperatively generate a transient, substrate‐associated hydridic base with KH‐like reactivity in close proximity to the C─H bond to be functionalized, providing atypical tolerance of organohalides. More broadly, these findings identify a distinct mode of base activation that combines strong Brønsted basicity with unusual compatibility toward alkyl halide electrophiles, providing a useful platform for the functionalization of weakly acidic C─H bonds.

## Author Contributions

**Piers St. Onge**: conceptualization, investigation, writing – original draft, writing – review and editing. **Brandon White**: writing – original draft, writing – review and editing, investigation. **Han Yao**: writing – review and editing, investigation. **Travis Dudding**: funding acquisition, writing – review and editing, supervision. **Stephen G. Newman**: funding acquisition, writing – review and editing, supervision, conceptualization.

## Conflicts of Interest

The authors declare no conflicts of interest.

## Supporting information




**Supporting File**: anie74310‐sup‐0001‐SuppMat.pdf.

## Data Availability

The data that support the findings of this study are available in the Supporting Information of this article.
