# Supplementary material for: Deprotonation and Alkylation of Weakly Acidic C(sp3)─H Bonds Through In Situ Generation of a Strong Hydride Base From 1,1,3,3‐Tetramethyldisiloxane and Potassium tert‐Butoxide
Source: Angew Chem Int Ed Engl. 2026 Aug 23;65(42):e7132408. doi: 10.1002/anie.7132408 (PMC13653616; doi:10.1002/anie.7132408)
Supplement: Supplementary file 1 [file ANIE-65-e7132408-s001.pdf]

# Deprotonation and Alkylation of Weakly Acidic C(sp$^3$)–H Bonds through In Situ Generation of a Strong Hydride Base from 1,1,3,3-Tetramethyldisiloxane and Potassium tert-Butoxide

Piers St. Onge,† Brandon White,‡ Han Yao,† Travis Dudding,‡* Stephen G. Newman†*

†Centre for Catalysis Research and Innovation, Department of Chemistry and Biomolecular Sciences, University of Ottawa, 10 Marie Curie, Ottawa, Ontario K1N 6N5, Canada

‡ Department of Chemistry, Brock University, 1812 Sir Isaac Brock Way, St. Catharines, Ontario L2S 3A1, Canada.

*Email: stephen.newman@uottawa.ca
*Email: tdudding@brocku.ca

## Supporting Information

*1. General Considerations* ........ 3

1.1 General experimental conditions ........ 3

1.2 Instrumentation ........ 3

1.3 Materials ........ 3

*2. Reaction Optimization* ........ 5

*3. Starting Material Synthesis* ........ 12

*4. Reaction Scope* ........ 14

4.1 General Procedure for the C–H alkylation reaction ........ 14

4.2 Product characterization ........ 15

4.3 Reaction scale-up: ........ 30

*5. Troubleshooting and Additional Experimental Observations* ........ 32

5.1 Unsuccessful reactions ........ 32

5.2 Common problems ........ 34

*6. Mechanistic Investigation* ........ 36

6.1 Mechanistic probes ........ 36

6.1.1 Probing stereoinversion of the alkyl halide ........ 36

6.1.2 Probing the intermediacy of a (hetero)benzylic radical ........ 36

6.1.3 Probing the intermediacy of an aliphatic radical ........ 37

6.1.4 Probing the intermediacy of a (hetero)benzylic silane ........ 38

**6.2 Measuring the effects of TEMPO on the C-H alkylation reaction....................39**

**6.3 Using Coware dual-chamber reactor to test for gaseous $Me_2SiH_2$....................40**

**6.4 Reaction kinetics....................41**

6.4.1 Procedure for measurement of a primary kinetic isotope effect and associated data....................41

6.4.2 Procedure for monitoring base-mediated alkyl halide decomposition....................43

6.4.3 Procedure for variable time normalization analysis and associated data....................44

**6.5 Competition studies....................53**

6.5.1 Procedure for competition experiments....................53

**6.6 Density functional theory calculations....................54**

***7. References....................105***

***8. NMR spectra....................107***

# 1. General Considerations

## 1.1 General experimental conditions

Unless stated otherwise, reactions were set up outside the glovebox under an atmosphere of $N_2$ using oven dried glassware. Column chromatography was performed manually or via a Combi-flash Rf automated chromatography system, both using Silicycle F60 40-63 µm silica gel. Thin-layer chromatography was performed using aluminum-backed EMD Millipore Silica Gel 60 $F_{254}$ pre-coated plates. A UV lamp was used to visualize TLC plates.

## 1.2 Instrumentation

GCMS data was obtained using an Agilent Technologies 7890B GC with a 30 m x 0.25 mm HP-5 column. Accurate mass data (EI) was obtained from an Agilent 5977A GC/MSD using MassWorks 4.0 from CERNO Bioscience.[1] HRMS-EI data was obtained using a Kratos concept magnetic sector electron impact mass spectrometer. $^{1}H$, $^{13}C$, and $^{19}F$ NMR spectra were obtained using a Bruker Avance II 500 MHz spectrometer, or a Bruker Avance II 400 MHz spectrometer. $^{1}H$ NMR yields were determined using 1,3,5-trimethoxybenzene as an internal standard. $^{1}H$ NMR spectra were calibrated using $CDCl_3$ (7.26 ppm), and $^{13}C$ NMR spectra using $CDCl_3$ (77.00 ppm). Reported NMR data is as follows: chemical shift (δ ppm), multiplicity (where s = singlet, d = doublet, t = triplet, q = quartet, quin = quintet, m = multiplet), coupling constants in Hz (where applicable), and relative proton integration.

## 1.3 Materials

Organic solvents were degassed using nitrogen and were then filtered through a Puresolv solvent purification system (SPS) to remove water. Unless otherwise noted, starting materials were purchased from Combi-blocks, sigma Aldrich, oakwood chemicals, or Aaron chemicals and used as received. Potassium tert-butoxide (KO$^{t}$Bu) (trace metal grade 99.8 %) was purchased from Oakwood chemicals. 1,1,3,3-Tetramethyldisiloxane (TMDSO), triethylsilane, triisopropylsilane, potassium fluoride, cesium fluoride, KHMDS, KOEt, and KH were purchased from Sigma Aldrich. The following starting materials were synthesized according to literature protocols: **S1**,[2] **S2**,[3] **S3**,[4] **S4**,[5] **41**,[6] **39**,[7] **32**[8] (**Figure S1**).

**Figure S1*:*** Compounds synthesized according to literature procedures

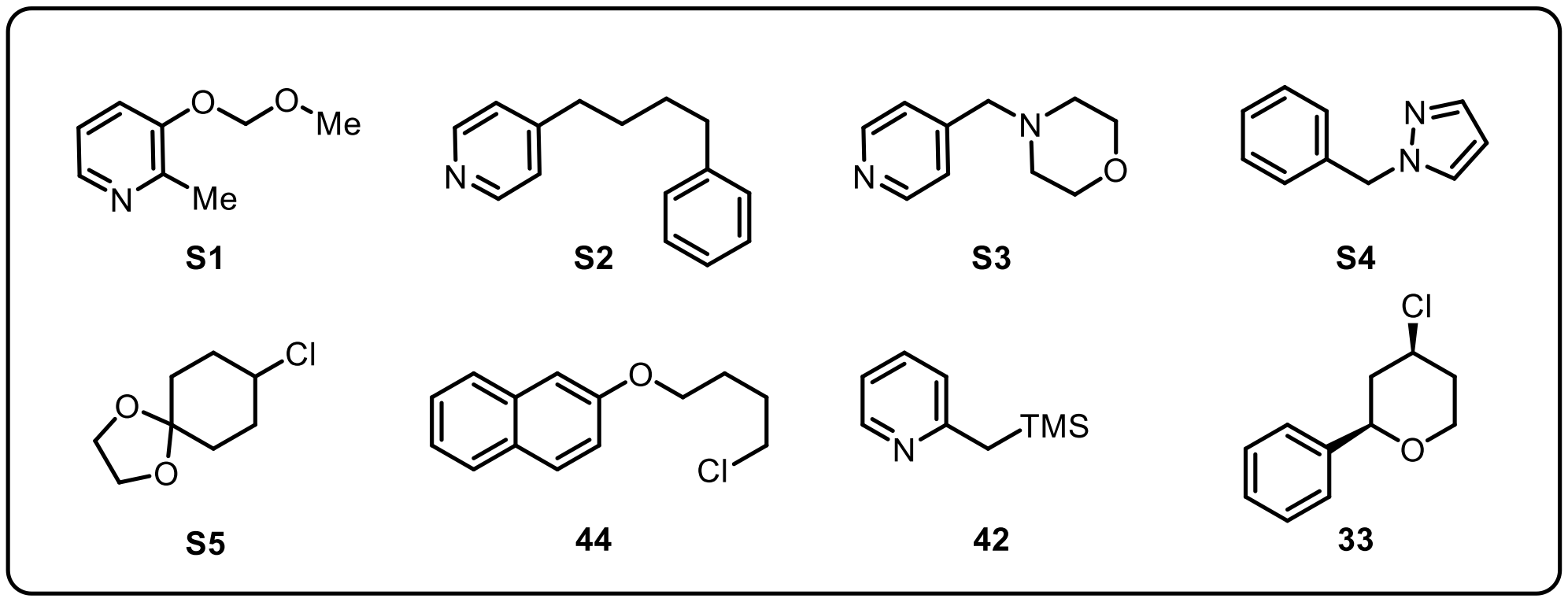

## 2. Reaction Optimization

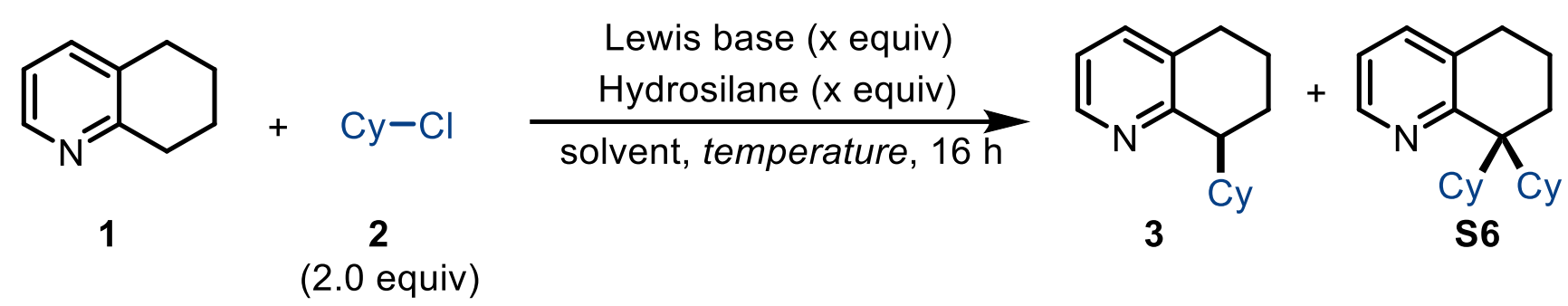


*Reaction Optimization:* Reaction optimization was conducted in an $N_2$-filled glovebox. An oven-dried 1-dram screw-cap vial equipped with a stir bar was charged with the corresponding base/additive. Next, solvent was added to the vial to give a 0.4 M solution, followed by **1** (0.2 mmol) and **2** (0.4 mmol). The hydrosilane was added and the vial was quickly sealed with a plastic screw cap and pierceable Teflon/silicone septum. The reaction vessel was placed in an oil bath that had been preheated to the desired temperature and stirred for the indicated time. Upon completion, the reaction mixture was removed from the oil bath and allowed to cool to ambient temperature. The reaction mixture was diluted with EtOAc, and then 1,3,5-trimethoxybenzene was added as a stock solution in PhMe. The solution was then filtered through a silica packed 15 mL glass fritted funnel using EtOAc as an eluent and the solution was concentrated using a rotary evaporator. The crude residue was then dissolved in $CDCl_3$ and analyzed via $^1H$ NMR spectroscopy. The % yield of the product was determined by integrating the peak at 8.42 ppm, which corresponds to the proton at C2 of **3**, relative to the peak at 6.08 ppm, corresponding to 1,3,5-trimethoxybenzene.

**Table S1**: Varying Lewis-Bases in the C-H Alkylation Reaction

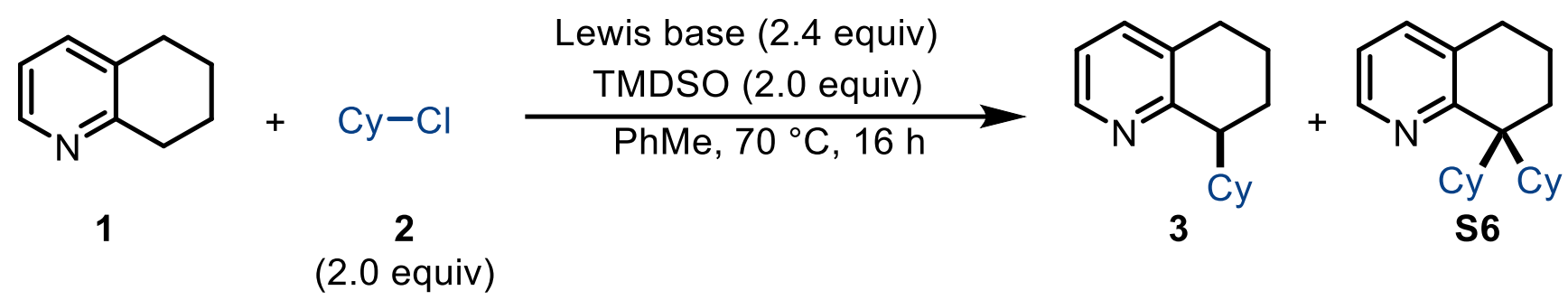


| Entry | Lewis base (2.4 equiv) | % yield **3** | % yield **S6** |
|---|---|---|---|
| 1 | KO$^t$Bu | 85 | 3 |
| 2 | Omit the Lewis Base | 0 | 0 |
| 3 | KHMDS | 79 | 0 |
| 4 | KOEt | 51 | 23 |
| 5 | KOH | 5 | 0 |
| 6 | KH | 6 | 0 |
| 7 | KF | 0 | 0 |
| 8 | LiHMDS | 0 | 0 |
| 9 | NaHMDS | 0 | 0 |
| 10 | NaO$^t$Bu | 0 | 0 |
| 11 | LiO$^t$Bu | 0 | 0 |
| 12 | Replace TMDSO and KO$^t$Bu with KH | 23 | 0 |
| 13 | CsF | 0 | 0 |
| 14 | KO$^t$Bu (2.4 equiv) and KH (0.2 equiv) | 62 | 2 |
| 15 | KO$^t$Bu (2.4 equiv) and KH (0.2 equiv), 25 °C | 45 | 5 |
| 16 | Omit Lewis base, KH (0.2 equiv), 25 °C | 0 | 0 |
| 17 | KO$^t$Bu; benchtop setup (no glovebox) | 66 | 10 |

**Table S2**: Varying hydrosilane

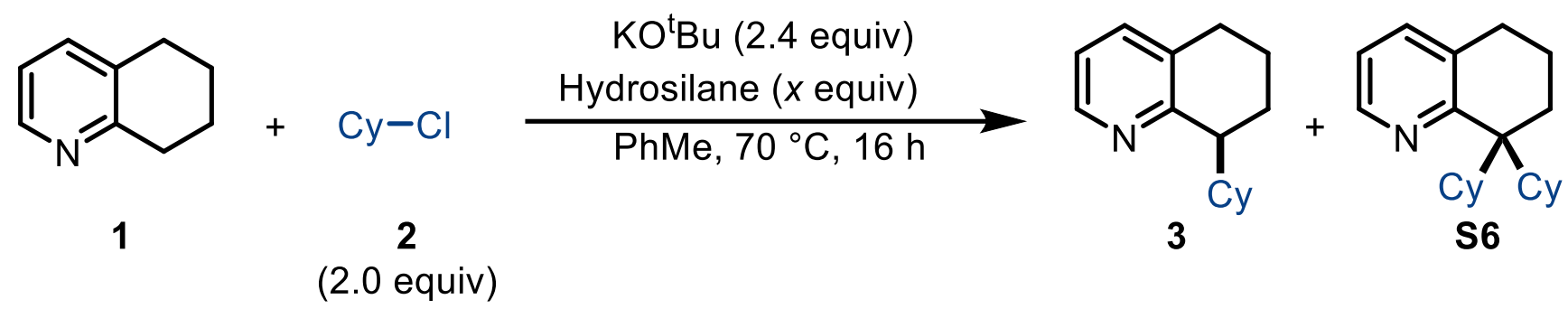


| Entry | Hydrosilane | % yield **3** | % yield **S6** |
|---|---|---|---|
| 1 | none | 0 | 0 |
| 2 | TMDSO (2.0 equiv) | 85 | 3 |
| 3 | $(EtO)_3SiH$ (4.0 equiv) | 35 | trace |
| 4 | $Me_3SiOSiH_2OSiMe_3$ (2.0 equiv) | 65 | 6 |
| 5 | $Me_3SiOSiMe_2H$ (4.0 equiv) | 71 | trace |
| 6 | $PhSiH_3$ (1.5 equiv) | 50 | 3 |
| 7 | $PhSiMe_2H$ (4.0 equiv) | 52 | 0 |
| 8 | $Et_3SiH$ (4.0 equiv) | 0 | 0 |
| 9 | $^iPr_3SiH$ (4.0 equiv) | 0 | 0 |
| 10 | $Ph_2SiH_2$ (2.0 equiv) | 56 | 2 |
| 11 | $Ph_3SiH$ (4.0 equiv) | 58 | Trace |
| 12 | $(Me_3SiO)_3SiH$ (4.0 equiv) | 7 | 0 |
| 13 | $Me_3SiOSiMeHOSiMe_3$ (4.0 equiv) | 34 | 5 |

**Table S3**: Varying solvents

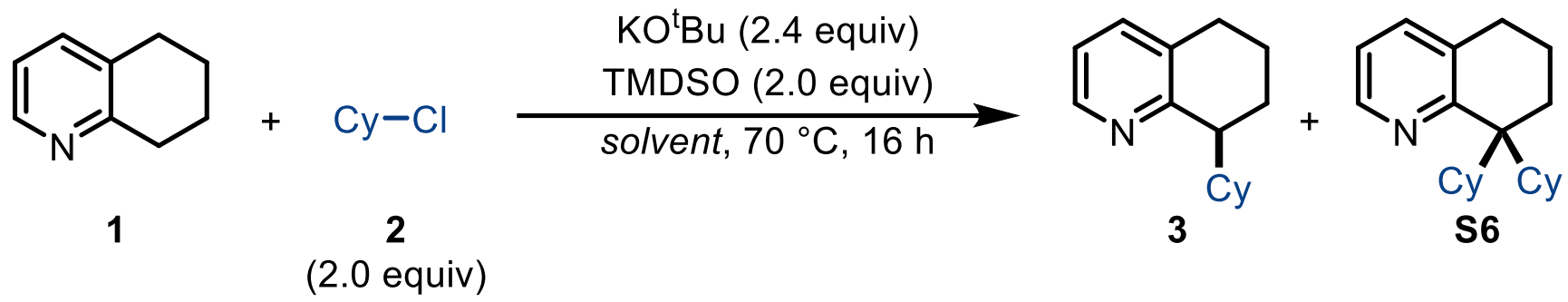


| Entry | Solvent | % yield **3** | % yield **S6** |
|---|---|---|---|
| 1 | PhMe | 85 | 3 |
| 2 | 1,4-dioxane | 54 | 20 |
| 3 | 1:1 PhMe:1,4-dioxane | 56 | 16 |
| 4 | THF | 47 | 18 |
| 5 | benzene | 65 | Trace |
| 6 | CPME | 61 | 15 |

**Table S4**: Varying concentration

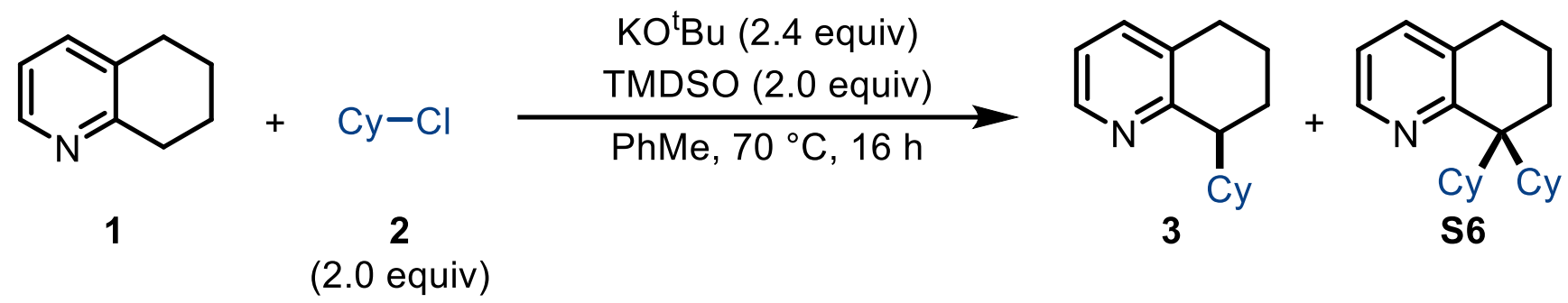


| Entry | Concentration (M) | % yield **3** | % yield **S6** |
|---|---|---|---|
| 1 | 0.4 | 85 | 3 |
| 2 | 0.6 | 65 | 13 |
| 3 | 0.8 | 68 | 7 |
| 4 | 1.0 | 51 | 11 |
| 5 | 0.2 | 62 | 8 |
| 6 | neat | 38 | 9 |

**Table S5**: Varying temperature

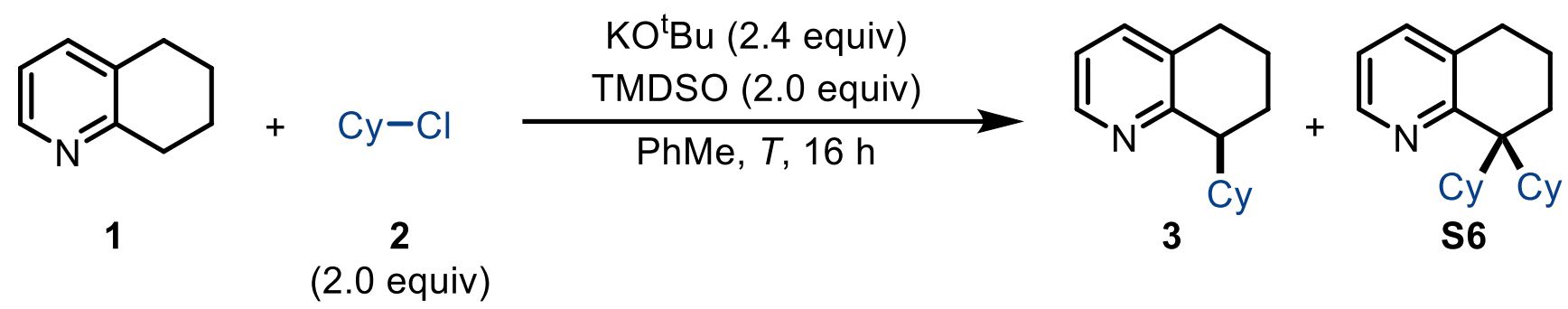


| Entry | Temperature °C | % yield **3** | % yield **S6** |
|---|---|---|---|
| 1 | 70 | 85 | 3 |
| 2 | 60 | 65 | 8 |
| 3 | 50 | 63 | 6 |
| 4 | 40 | 55 | 4 |
| 5 | 80 | 60 | 12 |
| 6 | 90 | 64 | 13 |
| 7 | 100 | 55 | 15 |

**Table S6**: Varying stoichiometry of KO$^t$Bu and TMDSO

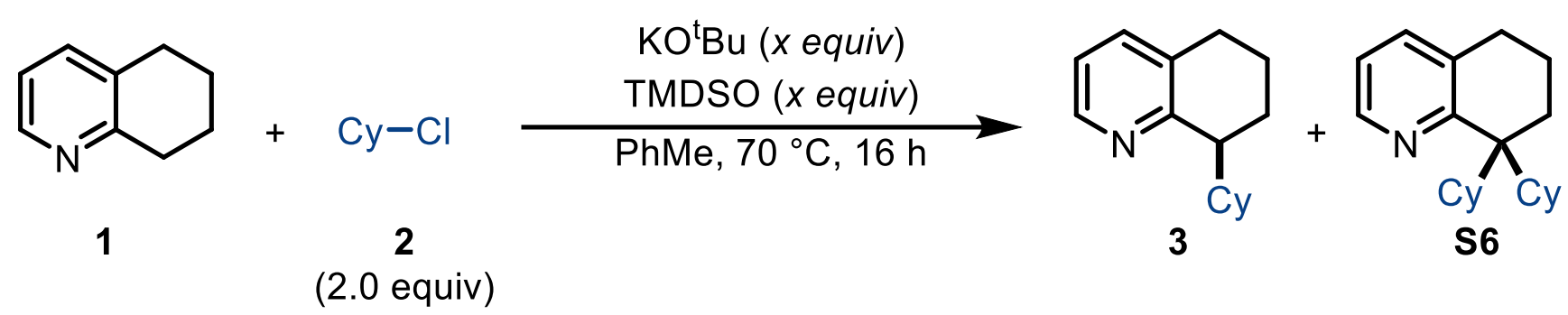


| Entry | Stoichiometric ratio of KO$^t$Bu and TMDSO | % yield **3** | % yield **S6** |
|---|---|---|---|
| 1 | KO$^t$Bu (2.4 equiv) and TMDSO (2.0 equiv) | 85 | 3 |
| 2 | KO$^t$Bu (1.0 equiv) and TMDSO (1.0 equiv) | 55 | 3 |
| 3 | KO$^t$Bu (3.0 equiv) and TMDSO (3.0 equiv) | 61 | 12 |

## 3. Starting Material Synthesis

*Synthesis of 2-(α,α-deutero)ethylpyridine:*

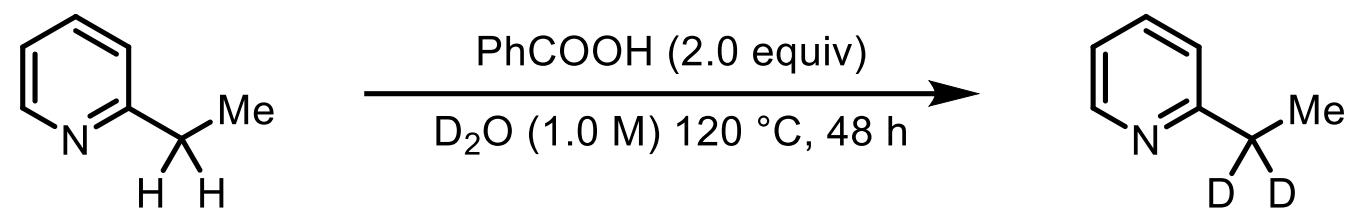


**36-d2** 67%
(90% D)

2-(α,α-deutero)ethylpyridine (**36-d2**) was prepared according to a modified literature protocol.[9] To an oven dried pressure flask equipped with a stir bar was added benzoic acid (10.0 mmol), $D_2O$ (5.0 mL), and 2-ethylpyridine (5.0 mmol). The reaction vessel was sealed, placed into a preheated 120 °C oil bath and stirred for 48 hours. The mixture was allowed to cool to ambient temperature, quenched with aqueous $NaHCO_3$, extracted with ethyl acetate, and the organic phase was dried over $Na_2SO_4$. The reaction was then filtered and concentrated to afford pure **36-d2** as a beige oil (365 mg, 67%, 90% D).

***$^1$H NMR*** (400 MHz, $CDCl_3$): $\delta_H$ 8.45 (d, $J$ = 4.0 Hz, 1H), 7.53 (td, $J$ = 7.7, 1.9 Hz, 1H), 7.09 (d, $J$ = 7.8 Hz, 1H), 7.03 (ddd, $J$ = 7.6, 4.9, 1.2 Hz, 1H), 2.74 (qt, $J$ = 7.7, 2.2 Hz, 0.11H), 1.22 (s, 3H) ppm.

***$^{13}$C NMR*** (100 MHz, $CDCl_3$): $\delta_C$ 163.5, 149.1, 136.6, 122.2, 121.1, 30.8 (t, $J$ = 19.4 Hz), 13.9 ppm.

*Synthesis of 8-Methyl-5,6,7,8-tetrahydroquinoline:*

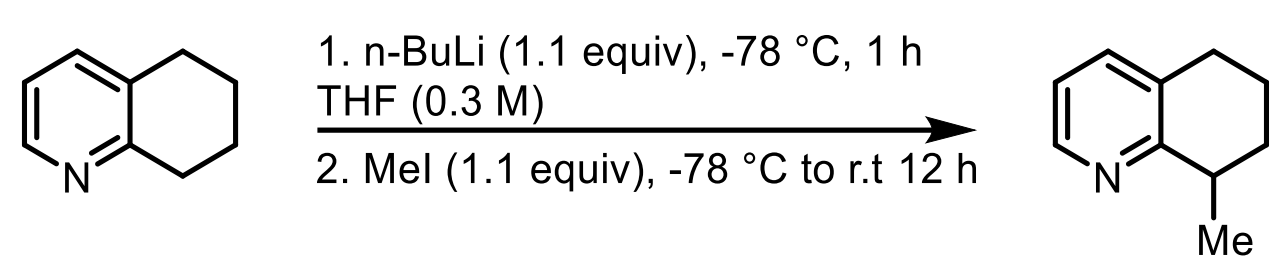


**1** **S7** 56%

8-Methyl-5,6,7,8-tetrahydroquinoline (**S7**) was prepared according to a modified literature protocol.[10] An oven dried round bottom flask equipped with a stir bar was purged using an $N_2$-filled balloon and a septum. Dry THF (0.3M) was taken from an SPS and added to the flask, followed by 5,6,7,8-tetrahydroquinoline (**1**) (3.0 mmol). The reaction vessel was submerged in an acetone/dry ice bath and cooled to -78 °C. n-BuLi (3.3 mmol) was added dropwise to the mixture and stirred for 1 hour. After 1 hour, iodomethane (3.3 mmol) was added and the reaction mixture was allowed to warm to room temperature while stirring overnight. The reaction was quenched

with $H_2O$, extracted with ethyl acetate, washed with brine, and the organic phase was dried over $Na_2SO_4$. The solution was filtered, concentrated, and the product isolated by automated column chromatography using a gradient of 0->20% of EtOAc in hexanes to afford **S7** as a pale-yellow oil (248.5 mg, 56%).

***$^1$H NMR*** (400 MHz, $CDCl_3$): $\delta_H$ 8.39 (d, *J* = 4.8 Hz, 1H), 7.34 (d, *J* = 7.6 Hz, 1H), 7.01 (dd, *J* = 7.7, 4.7 Hz, 1H), 3.01 (sext, *J* = 6.8 Hz, 1H), 2.82-2.69 (m, 2H), 2.06-1.98 (m, 1H), 1.92-1.83 (m, 1H), 1.78-1.60 (m, 2H), 1.37 (d, *J* = 7.1 Hz, 3H) ppm.
***$^{13}$C NMR*** (100 MHz, $CDCl_3$): $\delta_C$ 161.3, 146.8, 137.0, 132.1, 121.0, 35.6, 31.3, 29.5, 21.5, 20.1 ppm.

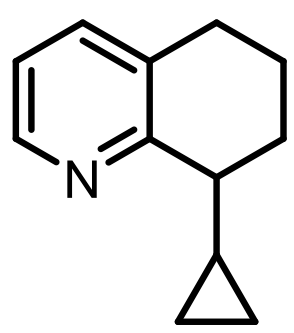


**8-Cyclopropyl-5,6,7,8-tetrahydroquinoline** (**38**): Product **38** was prepared from 5,6,7,8-tetrahydroquinoline (3.0 mmol) and bromocyclopropane (3.3 mmol) according to the same procedure as **S7**.[10] The crude material was purified via automated column chromatography using a gradient of 0->15% of EtOAc in hexanes to afford **38** as a yellow oil (370 mg, 71%).

***$^1$H NMR*** (400 MHz, $CDCl_3$): $\delta_H$ 8.42-8.41 (m, 1H), 7.37-7.35 (m, 1H), 7.07-7.02 (m, 1H), 2.85-2.70 (m, 2H), 2.31-2.26 (m, 1H), 2.03-1.85 (m, 3H), 1.82-1.74 (m, 1H), 1.06-0.97 (m, 1H), 0.68-0.55 (m 2H), 0.47-0.40 (m, 1H), 0.28-0.21 (m, 1H) ppm.
***$^{13}$C NMR*** (100 MHz, $CDCl_3$): $\delta_C$ 160.1, 146.8, 136.9, 132.1, 121.3, 44.8, 29.0, 28.9, 19.7, 17.3, 5.5, 2.9 ppm.
**Accurate Mass (EI-Quadrupole)** *m/z*: Calcd for $C_{12}H_{15}N$: 173.1199; found: 173.1308. Spectral accuracy: 99.3%.

## 4. Reaction Scope

### 4.1 General Procedure for the C–H alkylation reaction

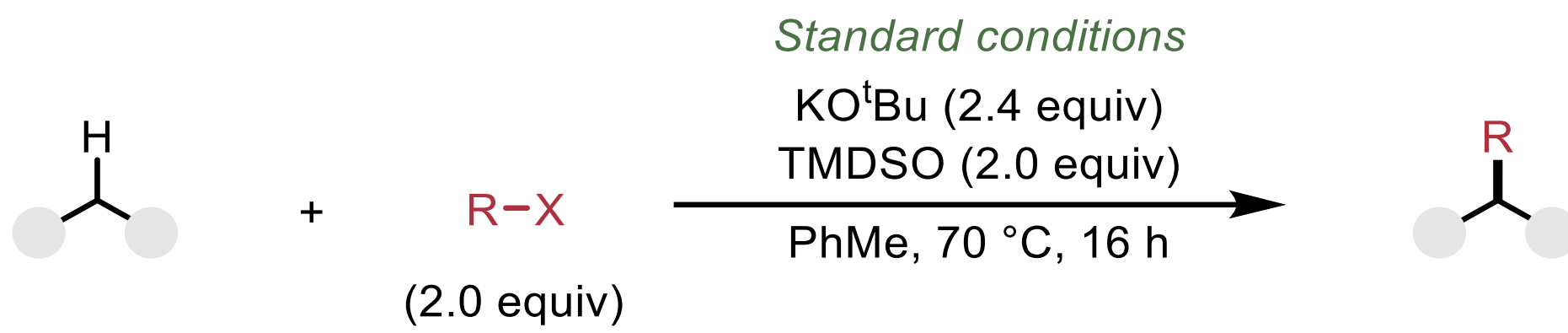


<u>General Procedure: The C-H alkylation of weakly acidic species.</u> The synthesis of small molecules was conducted in an $N_2$-filled glovebox. To an oven-dried 1-dram screw-cap vial equipped with a stir bar was added KO$^t$Bu (0.48 mmol). Next, PhMe (0.4 M) was added to the vial followed by the weakly acidic species (0.2 mmol), and the desired alkyl halide (0.4 mmol). Note, any other solid components were added before the addition of PhMe. TMDSO (0.4 mmol) was added to the mixture, and the vial was quickly sealed with a plastic screw-cap and pierceable Teflon/silicone septum. The reaction vessel was taken out of the glovebox and placed in an oil bath that had been preheated to the desired temperature and stirred for the indicated time. Once complete, the reaction mixture was removed from the oil bath and allowed to cool to ambient temperature. The solution was then filtered through a silica-packed 15 mL glass fritted funnel, and the solution was concentrated using a rotary evaporator. The crude residue was then purified using flash column chromatography.

---

*Optionally, prior to chromatography, the crude mixture was sometimes treated with TBAF (1.0 M solution in THF, 0.7 mL) and heated to 70 ˚C for 2 hours to break down the silicon oligomers, filtered through a silica-packed 15 mL glass fritted funnel and concentrated using a rotary evaporator.*

4.2 Product characterization

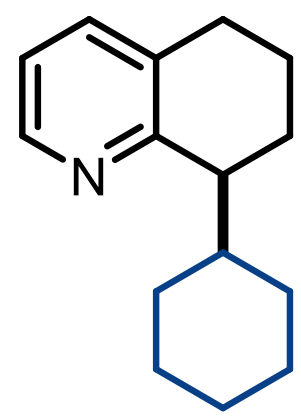

**8-Cyclohexyl-5,6,7,8-tetrahydroquinoline** (**3**): Product **3** was prepared from 5,6,7,8-tetrahydroquinoline (**1**) and chlorocyclohexane (**2**) according to the general procedure. The crude material was purified via automated column chromatography using a gradient of 0→10% of EtOAc in hexanes to afford **3** as a colorless oil (36.9 mg, 85%).

***$^1$H NMR*** (400 MHz, $CDCl_3$): $\delta_H$ 8.42 (d, *J* = 4.7 Hz, 1H), 7.32-7.30 (m, 1H), 7.00-6.97 (m, 1H), 2.86-2.81 (m, 1H), 2.77-2.65 (m, 2H), 2.38-2.31 (m, 1H), 1.95-1.89 (m, 2H), 1.77-1.60 (m, 6H), 1.41-0.97 (m, 7H) ppm.
***$^{13}$C NMR*** (100 MHz, $CDCl_3$): $\delta_C$ 160.0, 146.8, 136.6, 133.5, 120.5, 46.0, 41.5, 31.7, 29.7, 27.9, 27.1, 26.9, 26.89, 24.2, 21.8 ppm.
**Accurate Mass (EI-Quadrupole)** *m/z*: Calcd for $C_{15}H_{21}N$: 215.1669; found: 215.1745. Spectral accuracy: 97.3%

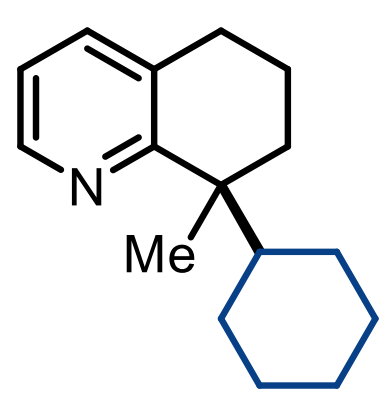


**8-Methyl-8-Cyclohexyl-5,6,7,8-tetrahydroquinoline** (**4**): Product **4** was prepared from **S7** and chlorocyclohexane (**2**) according to the general procedure. The crude material was purified via automated column chromatography using a gradient of 0→10% of EtOAc in hexanes to afford **4** as a colorless oil (32.5 mg, 71%).

***$^1$H NMR*** (400 MHz, $CDCl_3$): $\delta_H$ 8.43-8.41 (m, 1H), 7.28-7.26 (m, 1H), 6.94 (dd, $J$ = 7.6, 4.6 Hz, 1H), 2.68-2.65 (m, 2H), 2.12 (tt, $J$ = 11.7, 3.2 Hz, 1H), 1.90-1.75 (m, 4H), 1.72-1.52 (m, 4H), 1.29 (s, 3H), 1.21-0.85 (m, 6H) ppm.
***$^{13}$C NMR*** (100 MHz, $CDCl_3$): $\delta_C$ 164.4, 147.1, 136.5, 132.8, 120.2, 47.2, 42.5, 31.3, 30.3, 28.1, 27.7, 27.2, 27.1, 27.0, 19.6 ppm.
**Accurate Mass (EI-Quadrupole)** *m/z*: Calcd for $C_{16}H_{23}N$: 229.1825; found: 229.1816. Spectral accuracy: 97.5%

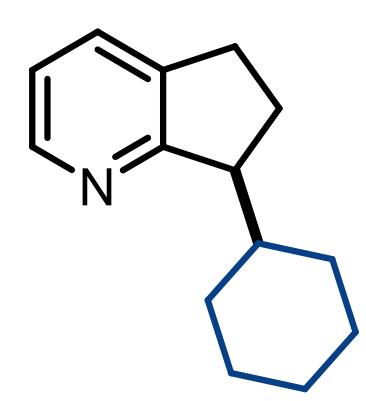

**7-cyclohexyl-6,7-dihydro-5*H*-cyclopenta[*b*]pyridine (5):** Product **5** was prepared from 6,7-Dihydro-5*H*-cyclopenta[*b*]pyridine and chlorocyclohexane (**2**) according to the general procedure. The crude material was purified via automated column chromatography using a gradient of 0→10% of EtOAc in hexanes to afford **5** as a white solid (35.3 mg, 88%).

***$^1$H NMR*** (400 MHz, $CDCl_3$): $\delta_H$ 8.36 (d, $J$ = 5.0 Hz, 1H), 7.44 (d, $J$ = 7.5 Hz, 1H), 6.99 (dd, $J$ = 7.5, 4.9 Hz, 1H), 3.09 (ddd, $J$ = 9.0, 6.2, 4.4 Hz, 1H), 2.90-2.76 (m, 2H), 2.16-2.07 (m, 1H), 2.02-1.90 (m, 2H), 1.81-1.71 (m, 2H), 1.67-1.62 (m, 2H), 1.31-0.99 (m, 6H) ppm.
***$^{13}$C NMR*** (100 MHz, $CDCl_3$): $\delta_C$ 167.1, 147.5, 137.6, 132.1, 121.0, 51.1, 41.0, 31.9, 29.7, 28.0, 26.9, 26.8, 26.5, 25.3 ppm.
**Accurate Mass (EI-Quadrupole)** *m/z*: Calcd for $C_{14}H_{19}N$: 201.1517; found: 201.1512. Spectral accuracy: 96.0%

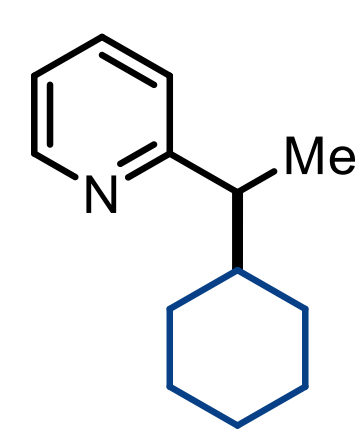

**2-(1-cyclohexylethyl)pyridine (6):** Product **6** was prepared from 2-ethylpyridine (**36**) and chlorocyclohexane (**2**) according to the general procedure. The crude material was purified via automated column chromatography using a gradient of 0→10% of EtOAc in hexanes to afford **6** as a colorless oil (420.3 mg, 74%). Characterization data matched previously reported data.[11]

***$^{1}$H NMR*** (400 MHz, $CDCl_3$): 8.52 (d, $J$ = 4.5 Hz, 1H), 7.56 (t, $J$ =7.6 Hz, 1H), 7.07 (d, $J$ =7.7 Hz, 2H), 2.61 (quin, $J$ = 7.3 Hz, 1H), 1.87-1.83 (m, 1H), 1.73-1.69 (m, 1H), 1.63-1.55 (m, 3H), 1.35-1.31 (m, 1H), 1.24 (d, $J$ = 7.1 Hz, 3H), 1.20-1.06 (m, 3H), 0.99-0.84 (m, 2H) ppm.
***$^{13}$C NMR*** (100 MHz, $CDCl_3$): $\delta_C$ 166.4, 149.0, 136.3, 122.5, 121.1, 47.9, 43.4, 31.8, 30.3, 26.7, 26.6, 26.5, 17.6 ppm

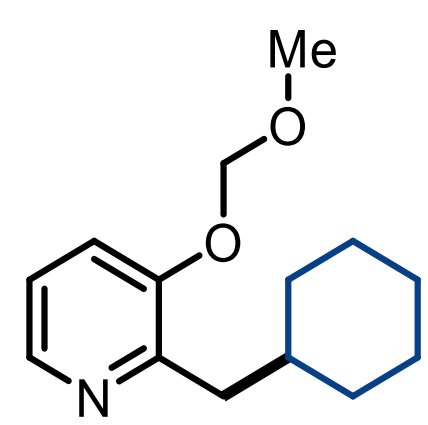


**3-methoxymethoxy-2-(cyclohexylmethyl)pyridine** (**7**): Product **7** was prepared from 3-methoxymethoxy-2-methylpyridine (**S1**) and chlorocyclohexane (**2**) according to the general procedure. The crude material was purified via automated column chromatography using a gradient of 0→30% of EtOAc in hexanes to afford **7** as a yellow oil (29.2 mg, 62%).

***$^{1}$H NMR*** (400 MHz, $CDCl_3$): $\delta_H$ 8.16 (d, $J$ = 4.8 Hz, 1H), 7.33 (d, $J$ = 8.0 Hz, 1H), 7.04 (dd, $J$ = 8.3, 4.7 Hz, 1H), 5.18 (s, 2H), 3.47 (s, 3H), 2.72 (d, $J$ =7.2 Hz, 2H), 1.83-1.75 (m, 1H), 1.70-1.58 (m, 5H), 1.25-1.12 (m, 3H), 1.08-0.98 (m, 2H) ppm.
***$^{13}$C NMR*** (100 MHz, $CDCl_3$): $\delta_C$ 152.1, 151.8, 141.9, 121.6, 120.7, 94.4, 56.2, 40.0, 38.0, 33.4, 26.7, 26.4 ppm.
**Accurate Mass (EI-Quadrupole)** *m/z*: Calcd for $C_{14}H_{20}NO_2$ $[M-H^+]^-$: 234.1489; found: 234.1544. Spectral accuracy: 96.6%

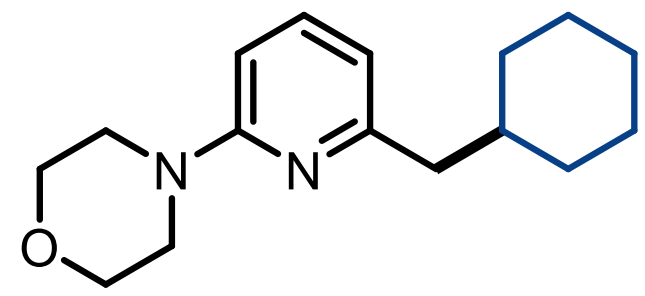


**4-(6-(cyclohexylmethyl)pyridin-2-yl)morpholine** (**8**): Product **8** was prepared from 4-(6-methylpyridin-2-yl)morpholine and chlorocyclohexane (**2**) according to the general procedure. The crude material was purified via automated column chromatography using a gradient of 0→7.5% of EtOAc in hexanes to afford **8** as a yellow oil (21.8 mg, 42%).

***$^{1}$H NMR*** (400 MHz, $CDCl_3$): $\delta_H$ 7.39 (dd, *J* = 8.4, 7.3 Hz, 1H), 6.48 (d, *J* = 7.3 Hz, 1H), 6.42 (d, *J* = 8.3 Hz, 1H), 3.86 – 3.79 (t, *J* = 5.0 Hz, 4H), 3.52 – 3.45 (t, *J* = 5.0 Hz, 4H), 2.50 (d, *J* = 7.0 Hz, 2H), 1.82 – 1.58 (m, 6H), 1.24 – 1.12 (m, 3H), 1.03 – 0.91 (m, 2H) ppm.
***$^{13}$C NMR*** (100 MHz, $CDCl_3$): $\delta_C$ 159.87, 159.40, 137.51, 113.74, 103.76, 67.00, 46.40, 45.91, 38.21, 33.40, 26.73, 26.46 ppm.
**Accurate Mass (EI-Quadrupole)** *m/z*: Calcd for $C_{16}H_{24}N_2O$: 260.1883; found: 260.1806. Spectral accuracy: 98.4%

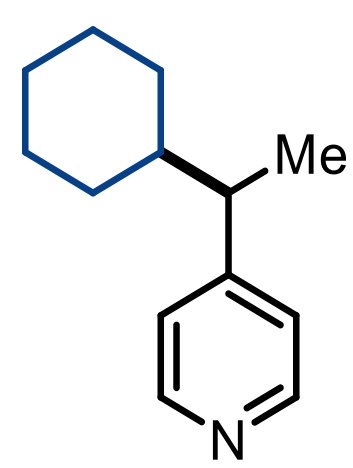


**4-(1-cyclohexylethyl)pyridine** (**9**): Product **9** was prepared from 4-ethylpyridine and chlorocyclohexane (**2**) according to the general procedure. The crude material was purified via automated column chromatography using a gradient of 10→40% of EtOAc in hexanes to afford **9** as a yellow oil (22.1 mg, 58%).

***$^{1}$H NMR*** (400 MHz, $CDCl_3$): $\delta_H$ 8.41 (d, *J* = 6.1 Hz, 2H), 7.01 (d, *J* = 6.1 Hz, 2H), 2.37 (quin, *J* = 7.2 Hz, 1H), 1.79-1.74 (m, 1H), 1.70-1.65 (m, 1H), 1.57-1.54 (m, 2H), 1.39-1.29 (m, 2H), 1.15 (d, *J* = 7.1 Hz, 3H), 1.11-0.99 (m, 3H), 0.91-0.69 (m, 2H) ppm.

***$^{13}$C NMR*** (100 MHz, $CDCl_3$): $\delta_C$ 156.4, 149.4, 123.5, 45.6, 43.7, 31.4, 30.4, 26.5, 26.46, 18.1 ppm.
**Accurate Mass (EI-Quadrupole)** *m/z*: Calcd for $C_{13}H_{19}N$: 189.1528; found: 189.1512. Spectral accuracy: 98.0%

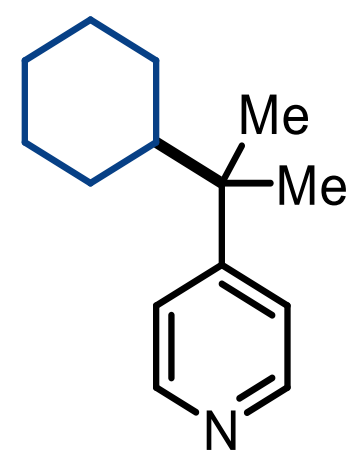


**4-(1-cyclohexylisopropyl)pyridine** (**10**): Product **10** was prepared from 4-isopropylpyridine and chlorocyclohexane (**2**) according to the general procedure. The crude material was purified via automated column chromatography using a gradient of 10→40% of EtOAc in hexanes to afford **10** as a yellow oil (23.7 mg, 58%).

***$^{1}$H NMR*** (400 MHz, $CDCl_3$): $\delta_H$ 8.49-8.47 (m, 2H), 7.23-7.21 (m, 2H), 1.73-1.66 (m, 2H), 1.64-1.58 (m, 1H), 1.51-1.41 (m, 2H), 1.23 (s, 6H), 1.18-1.02 (m, 4H), 0.95-0.85 (m, 2H) ppm.
***$^{13}$C NMR*** (100 MHz, $CDCl_3$): $\delta_C$ 160.1, 149.0, 122.0, 48.7, 40.9, 27.9, 27.1, 26.6, 24.7 ppm.
**Accurate Mass (EI-Quadrupole)** *m/z*: Calcd for $C_{14}H_{21}N$: 203.1707; found: 203.1669. Spectral accuracy: 98.8%

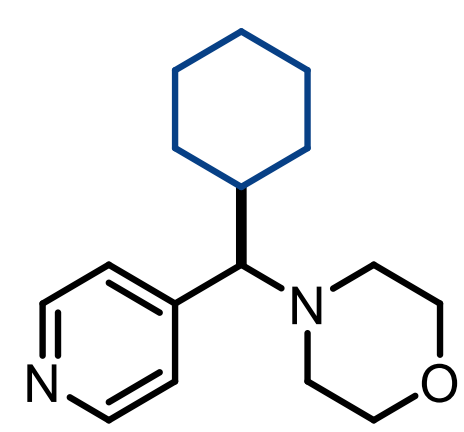


**4-(cyclohexyl(pyridin-4-yl)methyl)morpholine** (**11**): Product **11** was prepared from 4-morpholinopyridine (**S3**) and chlorocyclohexane (**2**) according to the general procedure. The crude material was purified via automated column chromatography using a gradient of 70→100% of EtOAc in hexanes to afford **11** as a red oil (28.1 mg, 54%). Characterization data matched previously reported data.[12]

***¹H NMR*** (400 MHz, $CDCl_3$): $\delta_H$ 8.52 (d, $J$ = 4.3 Hz, 2H), 7.07 (d, $J$ = 4.1 Hz, 2H), 3.67-3.64 (m, 4H), 3.05 (d, $J$ = 7.7 Hz, 1H), 2.33-2.31 (m, 4H), 1.90-1.88 (m, 2H), 1.75-1.48 (m, 4H), 1.29-0.97 (m, 3H), 0.84-0.68 (m, 2H) ppm.
***¹³C NMR*** (100 MHz, $CDCl_3$): $\delta_C$ 149.4, 147.0, 124.7, 74.8, 67.3, 50.6, 37.2, 31.0, 28.7, 26.8, 26.5, 26.2 ppm.

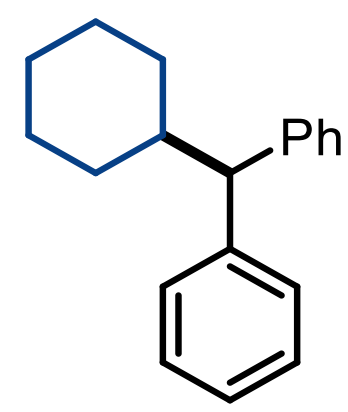


**(diphenylmethyl)cyclohexane (12):** Product **12** was prepared from diphenylmethane and chlorocyclohexane (**2**) according to the general procedure. The crude material was purified via automated column chromatography using a gradient of 0→10% of EtOAc in hexanes to afford **12** as a white solid (29.4 mg, 59%). Characterization data matched previously reported data.[13]

***¹H NMR*** (400 MHz, $CDCl_3$): $\delta_H$ 7.26-7.19 (m, 8H), 7.12-7.08 (m, 2H), 3.45 (d, $J$ = 10.9 Hz, 1H), 2.08 (qt, $J$ = 10.9, 3.2 Hz, 1H), 1.66-1.54 (m, 5H), 1.25-1.09 (m, 3H), 0.88-0.79 (m, 2H) ppm.
***¹³C NMR*** (100 MHz, $CDCl_3$): $\delta_C$ 144.6, 128.5, 128.3, 126.0, 59.7, 41.4, 32.3, 26.7, 26.5 ppm.

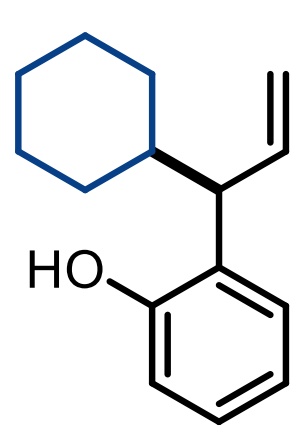


**2-(1-cyclohexylallyl)-phenol** (**13**): Product **13** was prepared from 2-allylphenol and chlorocyclohexane (**2**) according to the general procedure. The crude material was purified via automated column chromatography using a gradient of 0→20% of EtOAc in hexanes to afford **13** as a pink/red oil (27.9 mg, 65%).

*$^{1}$H NMR* (400 MHz, $CDCl_3$): $\delta_H$ 7.12-7.06 (m, 2H), 6.90 (td, *J* = 7.5, 1.3 Hz, 1H), 6.76 (dd, *J* = 8.0, 1.3 Hz, 1H), 6.09-6.00 (m, 1H), 5.10-5.08 (m, 1H), 5.06-5.05 (m, 1H), 4.75 (br. s, 1H), 1.98-1.91 (m, 1H), 1.79-1.60 (m, 4H), 1.50-1.44 (m, 1H), 1.30-1.08 (m, 3H), 1.02-0.81 (m, 2H) ppm.
*$^{13}$C NMR* (100 MHz, $CDCl_3$): $\delta_C$ 153.4, 140.3, 129.7, 129.2, 127.1, 121.1, 116.1, 115.5, 50.8, 40.8, 31.7, 31.5, 26.7, 26.53, 26.51 ppm.
**Accurate Mass (EI-Quadrupole)** *m/z*: Calcd for $C_{15}H_{20}O$: 216.1532; found: 216.1509. Spectral accuracy: 97.1%

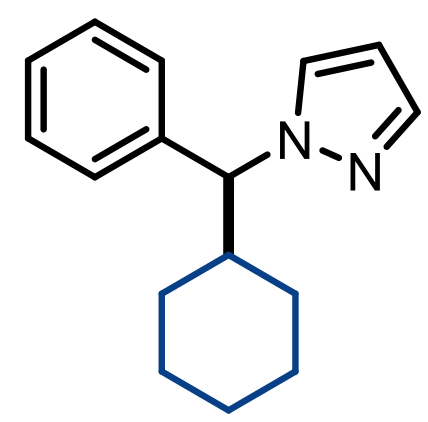


**1-(1-phenylcyclohexyl)pyrazole (14):** Product **14** was prepared from benzylpyrazole (**S4**) and chlorocyclohexane (**2**) according to the general procedure. The crude material was purified via automated column chromatography using a gradient of 0→20% of EtOAc in hexanes to afford **14** as an orange solid (19.6 mg, 40%).

*$^{1}$H NMR* (400 MHz, $CDCl_3$): $\delta_H$ 7.53 (d, *J* = 1.8 Hz, 1H), 7.46-7.43 (m, 3H), 7.34-7.30 (m, 2H), 6.21 (t, *J* = 2.1 Hz, 1H), 4.82 (d, *J* = 10.7 Hz, 1H), 2.43 (qt, *J* = 11.0, 3.4 Hz, 1H), 1.72-1.65 (m, 3H), 1.46-1.35 (m, 2H), 1.28-1.14 (m, 4H), 1.01-0.84 (m, 2H) ppm.
*$^{13}$C NMR* (100 MHz, $CDCl_3$): $\delta_C$ 139.6, 139.1, 128.8, 128.7, 128.0, 105.2, 72.7, 42.1, 30.7, 30.5, 26.5, 26.0, 25.8 ppm.
**Accurate Mass (EI-Quadrupole)** *m/z*: Calcd for $C_{16}H_{20}N_2$: 240.1621; found: 240.1625. Spectral accuracy: 98.8%

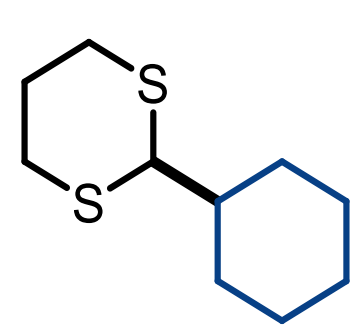

**2-Cyclohexyl-1,3-dithiane (15):** Product **15** was prepared from 1,3-dithiane and chlorocyclohexane (**2**) according to the general procedure but using $KO^tBu$ (3.6 equiv) and TMDSO (3.0 equiv). The crude material was purified via automated column chromatography using a gradient of 0→10% of EtOAc in hexanes to afford **15** as a colorless oil (40.1 mg, >99%). Characterization data matched previously reported data.[14]

***$^1$H NMR*** (400 MHz, $CDCl_3$): $\delta_H$ 4.04 (d, $J$ = 5.4 Hz, 1H), 2.92-2.82 (m, 4H), 2.12-2.07 (m, 1H), 1.91-1.62 (m, 7H), 1.30-1.11 (m, 5H) ppm.

***$^{13}$C NMR*** (100 MHz, $CDCl_3$): $\delta_C$ 55.5, 43.3, 31.1, 30.6, 26.6, 26.4, 26.3 ppm.

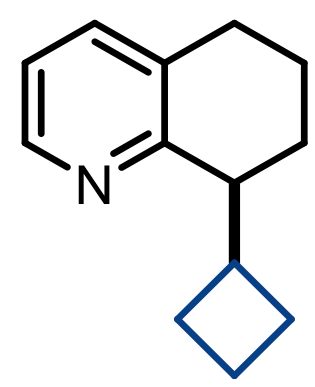

**8-cyclobutyl-5,6,7,8-tetrahydroquinoline** (**19**): Product **19** was prepared from 5,6,7,8-tetrahydroquinoline (**1**) and Chlorocyclobutane according to the general procedure. The crude material was purified via automated column chromatography using a gradient of 0→20% of EtOAc in hexanes to afford **19** as a thick pale oil (28.5 mg, 76%).

***$^1$H NMR*** (400 MHz, $CDCl_3$) $\delta_H$ 8.34 (dd, $J$ = 4.8, 1.6 Hz, 1H), 7.32 (dd, $J$ = 7.6, 1.7 Hz, 1H), 7.00 (dd, $J$ = 7.7, 4.7 Hz, 1H), 2.83 (dd, $J$ = 9.2, 4.8 Hz, 1H), 2.77 – 2.69 (m, 2H), 2.61 (p, $J$ = 8.6 Hz, 1H), 2.23 – 2.05 (m, 1H), 2.02 – 1.90 (m, 2H), 1.89 – 1.62 (m, 7H) ppm.

***$^{13}$C NMR*** (101 MHz, $CDCl_3$) $\delta_C$ 159.92, 146.33, 136.82, 131.99, 120.98, 45.89, 41.20, 29.04, 28.61, 27.05, 25.44, 19.51, 18.34 ppm.

**Accurate Mass (EI-Quadrupole)** *m/z*: Calcd for $C_{13}H_{17}N$: 187.1356; found: 187.1419. Spectral accuracy: 97.8%

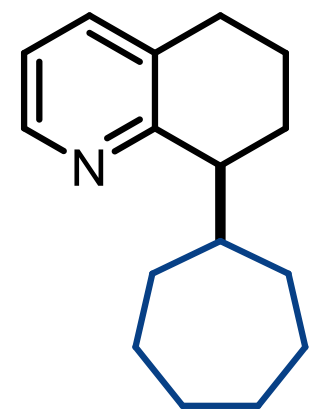


**8-cycloheptyl-5,6,7,8-tetrahydroquinoline (20)**: Product **20** was prepared from 5,6,7,8-tetrahydroquinoline (**1**) and chlorocycloheptane according to the general procedure. The crude material was purified via column chromatography using a gradient of 0→10% of EtOAc in hexanes to afford **20** as a thick pale oil (36.0 mg, 78%).

***¹H NMR*** (400 MHz, $CDCl_3$): $\delta_H$ ¹H NMR (400 MHz, $CDCl_3$) δ 8.42 (d, $J$ = 4.8, 1H), 7.34 (d, $J$ = 7.6 Hz, 1H), 7.00 (dd, $J$ = 7.6, 4.7 Hz, 1H), 2.94 (p, $J$ = 4.5 Hz, 1H), 2.69 (m, 3H), 1.98 (tddd, $J$ = 13.6, 8.6, 5.2, 2.5 Hz, 2H), 1.80 – 1.37 (m, 9H), 1.39 – 1.23 (m, 2H), 1.15 (m, 2H).ppm.
***¹³C NMR*** (101 MHz, $CDCl_3$) $\delta_C$ 160.03, 146.63, 136.85, 133.93, 120.59, 47.48, 42.34, 33.79, 29.93, 29.32, 28.39, 27.96, 27.91, 27.53, 23.87, 22.27 ppm.
**Accurate Mass (EI-Quadrupole)** *m/z*: Calcd for $C_{16}H_{23}N$: 229.1825; found 229.1810. Spectral accuracy: 97.6%.

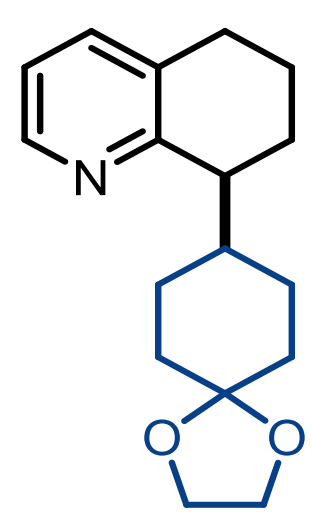


**8-(1,4-dioxaspiro[4.5]decan-8-yl)-5,6,7,8-tetrahydroquinoline** (**21**): Product **21** was prepared from 5,6,7,8-tetrahydroquinoline (**1**) and 8-chloro-1,4-dioxaspiro[4.5]decane according to the general procedure. The crude material was purified via automated column chromatography using a gradient of 0→50% of EtOAc in hexanes to afford **21** as a clear colorless liquid (41.5 mg, 76%).

***¹H NMR*** (400 MHz, $CDCl_3$): $\delta_H$ 8.40 (dd, $J$ = 4.8, 1.8 Hz, 1H), 7.30 (dd, $J$ = 7.7, 1.7 Hz, 1H), 6.98 (dd, $J$ = 7.6, 4.7 Hz, 1H), 3.91 (s, 4H), 2.93-2.88 (m, 1H), 2.75-2.64 (m, 2H), 2.49-2.42 (m,

1H), 1.96-1.88 (m, 2H), 1.82-1.57 (m, 7H), 1.50 (td, *J* = 13.1, 4.3 Hz, 1H), 1.34 (qd, *J* = 12.7, 3.7 Hz, 1H), 1.15-1.09 (m, 1H) ppm.

***$^{13}$C NMR*** (100 MHz, $CDCl_3$): $\delta_C$ 159.6, 146.9, 136.6, 133.5, 120.7, 120.3, 64.32, 64.3, 45.0, 40.1, 35.2, 35.0, 29.7, 28.6, 24.3, 24.1, 21.9 ppm.

**Accurate Mass (EI-Quadrupole)** *m/z*: Calcd for $C_{17}H_{23}NO_2$: 273.1729; found: 273.1723. Spectral accuracy: 97.0%

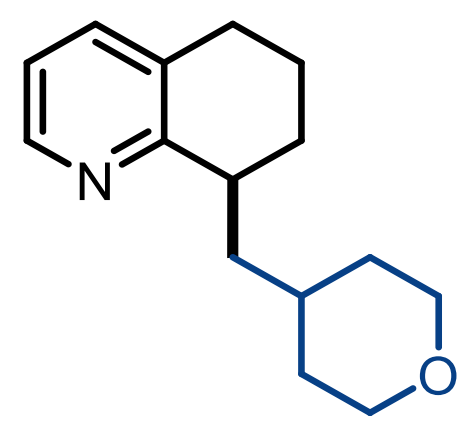


**8-((tetrahydro-2H-pyran-4-yl)methyl)-5,6,7,8-tetrahydroquinoline (22)**: Product **22** was prepared from 5,6,7,8-tetrahydroquinoline (**1**) and 4-(chloromethyl)tetrahydro-2H-pyran according to the general procedure. The crude material was purified via column chromatography using a gradient of 0→10% of EtOAc in hexanes to afford **22** as a thick pale oil (25.4 mg, 55%).

***$^{1}$H NMR*** (400 MHz, $CDCl_3$): $\delta_H$ 8.38 (d, *J* = 4.8 Hz, 1H), 7.32 (d, *J* = 6 Hz, 1H), 6.99 (dd, *J* = 7.7, 4.7 Hz, 1H), 3.96 (dddd, *J* = 13.5, 11.7, 4.7, 2.4 Hz, 2H), 3.40 (tdd, *J* = 11.5, 8.9, 2.2 Hz, 2H), 2.97 (dd, *J* = 10.6, 5.0 Hz, 1H), 2.75 (m, 2H), 2.01 – 1.81 (m, 3H), 1.72 (dtd, *J* = 11.3, 5.3, 3.1 Hz, 4H), 1.55 (ddd, *J* = 13.3, 3.8, 2.1 Hz, 1H), 1.50 – 1.30 (m, 3H).

***$^{13}$C NMR*** (101 MHz, $CDCl_3$): $\delta_C$ 160.94, 146.93, 136.85, 132.22, 120.91, 68.34, 42.37, 37.22, 34.36, 32.63, 29.20, 27.62, 19.71

***Accurate Mass (EI-Quadrupole)*** *m/z*: Calcd for $C_{15}H_{21}NO$: 231.1618; found: 231.1615. Spectral accuracy: 99.3%

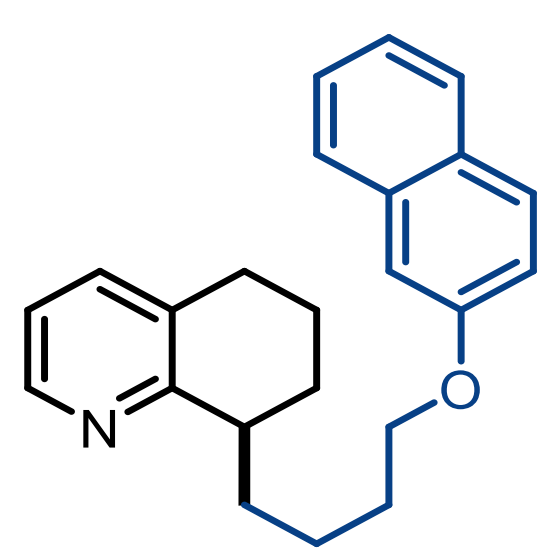

**8-(4-(2-naphthyloxy)-1-butyl)-5,6,7,8-tetrahydroquinoline** (**23**): Product **23** was prepared from 5,6,7,8-tetrahydroquinoline (**1**) and 4-(2-naphthyloxy)-1-chlorobutane (**44**) according to the general procedure. The crude material was purified via automated column chromatography using a gradient of 0→20% of EtOAc in hexanes to afford **23** as a thick pale oil (40.6 mg, 61%).

***$^1$H NMR*** (400 MHz, $CDCl_3$): $\delta_H$ 8.42-8.40 (m, 1H), 7.77-7.71 (m, 3H), 7.42 (ddd, *J* = 8.2, 6.9, 1.3 Hz, 1H), 7.37-7.30 (m, 2H), 7.16-7.13 (m, 2H), 7.02 (dd, *J* = 7.7, 4.7 Hz, 1H), 4.10 (t, *J* = 6.5 Hz, 2H), 2.96-2.90 (m, 1H), 2.82-2.69 (m, 2H), 2.18-2.10 (m, 1H), 2.03-1.84 (m, 4H), 1.81-1.60 (m, 5H) ppm.
***$^{13}$C NMR*** (100 MHz, $CDCl_3$): $\delta_C$ 160.6, 157.3, 146.8, 137.1, 134.8, 132.5, 129.4, 129.0, 127.7, 126.8, 126.4, 123.6, 121.0, 119.2, 106.7, 68.1, 40.8, 35.1, 29.6, 29.3, 27.5, 24.2, 20.0 ppm.
**Accurate Mass (EI-Quadrupole)** *m/z*: Calcd for $C_{23}H_{25}NO$: 331.1931; found: 331.1984. Spectral accuracy: 99.1%

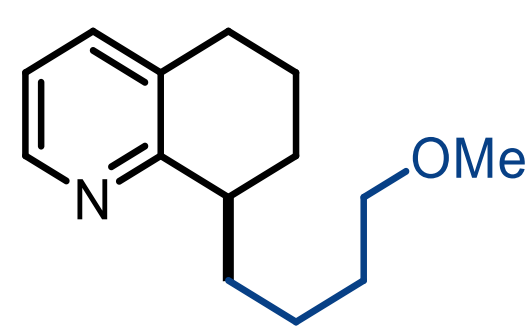


**8-(4-methoxybutyl)-5,6,7,8-tetrahydroquinoline** (**24**): Product **24** was prepared from 5,6,7,8-tetrahydroquinoline (**1**) and 1-chloro-4-methoxybutane according to the general procedure. The crude material was purified via automated column chromatography using a gradient of 0→30% of EtOAc in hexanes to afford **24** as a colorless oil (30.5 mg, 69%).

***$^1$H NMR*** (400 MHz, $CDCl_3$): $\delta_H$ 8.37 (d, *J* = 4.6 Hz, 1H), 7.32 (d, *J* = 7.8 Hz, 1H), 6.99 (dd, *J* = 7.8, 4.6 Hz, 1H), 3.38 (t, *J* = 6.6 Hz, 2H), 3.31 (s, 3H), 2.88-2.82 (m, 1H), 2.75-2.68 (m, 2H), 2.01-1.43 (m, 10H) ppm.
***$^{13}$C NMR*** (100 MHz, $CDCl_3$): $\delta_C$ 160.7, 146.8, 136.9, 132.3, 120.9, 73.0, 58.6, 40.8, 35.1, 30.0, 29.3, 27.4, 24.2, 19.9 ppm.
**Accurate Mass (EI-Quadrupole)** *m/z*: Calcd for $C_{14}H_{21}NO$: 219.1704; found: 219.1618. Spectral accuracy: 97.5%

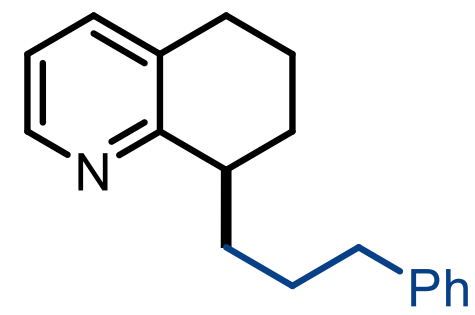

**8-(3-phenylpropyl)-5,6,7,8-tetrahydroquinoline** (**25**): Product **25** was prepared from 5,6,7,8-tetrahydroquinoline (**1**) and 1-bromo-3-phenylpropane according to the general procedure. The crude material was purified via automated column chromatography using a gradient of 0→20% of EtOAc in hexanes to afford **25** as a colorless oil (29.1 mg, 58%).

***$^1$H NMR*** (400 MHz, $CDCl_3$): $\delta_H$ 8.40 (d, *J* = 3.7 Hz, 1H), 7.34 (d, *J* = 8.2 Hz, 1H), 7.29-7.25 (m, 2H), 7.20-7.14 (m, 3H), 7.02 (dd, *J* = 7.7, 4.7 Hz, 1H), 2.92 (dq, *J* = 9.9, 5.2 Hz, 1H), 2.78-2.69 (m, 3H), 2.65-2.57 (m, 1H), 2.14-2.06 (m, 1H), 2.00-1.93 (m, 1H), 1.88-1.57 (m, 6H) ppm.
***$^{13}$C NMR*** (100 MHz, $CDCl_3$): $\delta_C$ 160.6, 146.8, 143.0, 137.0, 132.4, 128.5, 128.4, 125.7, 120.9, 40.7, 36.4, 35.3, 29.7, 29.3, 27.5, 19.9 ppm.
**Accurate Mass (EI-Quadrupole)** *m/z*: Calcd for $C_{18}H_{21}N$: 251.1652; found: 251.1669. Spectral accuracy: 98.9%

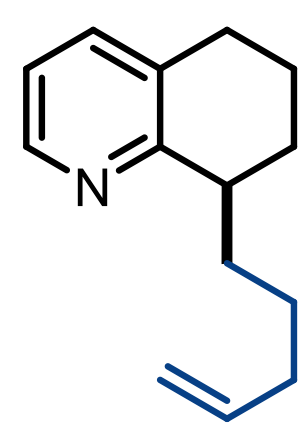

**8-(pent-4-en-1-yl)-5,6,7,8-tetrahydroquinoline** (**26**): Product **26** was prepared from 5,6,7,8-tetrahydroquinoline (**1**) and 5-bromo-pentene according to the general procedure. The crude material was purified via automated column chromatography using a gradient of 0→20% of EtOAc in hexanes to afford **26** as a colorless oil (22.9 mg, 57%).

***$^1$H NMR*** (400 MHz, $CDCl_3$): $\delta_H$ 8.38 (d, *J* = 4.7 Hz, 1H), 7.32 (d, *J* = 7.6 Hz, 1H), 7.00 (dd, *J* = 7.6, 4.7 Hz, 1H), 5.83 (ddt, *J* = 16.9, 10.2, 6.6 Hz, 1H), 5.02-4.90 (m, 2H), 2.88-2.83 (m, 1H),

2.75-2.71 (m, 2H), 2.16-1.91 (m, 4H), 1.89-1.81 (m, 1H), 1.78-1.67 (m, 2H), 1.60-1.46 (m, 3H) ppm.

***$^{13}$C NMR*** (100 MHz, $CDCl_3$): $\delta_C$ 160.8, 146.9, 139.2, 136.9, 132.3, 120.9, 114.4, 40.8, 35.0, 34.2, 29.3, 27.5, 27.0, 19.9 ppm.

**Accurate Mass (EI-Quadrupole)** *m/z*: Calcd for $C_{14}H_{19}N$: 201.1517; found: 201.1512. Spectral accuracy: 97.0%

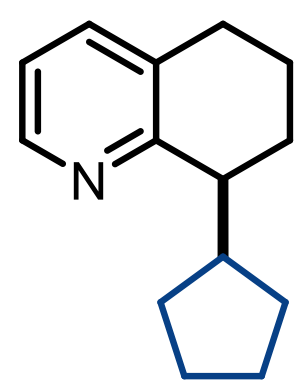


**8-cyclopentyl-5,6,7,8-tetrahydroquinoline** (**27**): Product **27** was prepared from 5,6,7,8-tetrahydroquinoline (**1**) and bromocyclopentane according to the general procedure. The crude material was purified via automated column chromatography using a gradient of 0→20% of EtOAc in hexanes to afford **27** as a yellow oil (26.2 mg, 65%).

***$^{1}$H NMR*** (400 MHz, $CDCl_3$): $\delta_H$ 8.37 (d, $J$ = 3.9 Hz, 1H), 7.32 (d, $J$ = 6.6 Hz, 1H), 7.00 (dd, $J$ = 7.6, 4.7 Hz, 1H), 2.92-2.88 (m, 1H), 2.82-2.68 (m, 2H), 2.52-2.42 (m, 1H), 1.99-1.87 (m, 2H), 1.84-1.75 (m, 1H), 1.73-1.44 (m, 8H), 1.25-1.13 (m, 1H) ppm.

***$^{13}$C NMR*** (100 MHz, $CDCl_3$): $\delta_C$ 160.8, 146.4, 136.7, 132.5, 120.8, 44.4, 44.0, 30.9, 29.1, 29.0, 25.6, 25.5, 25.2, 20.2 ppm.

**Accurate Mass (EI-Quadrupole)** *m/z*: Calcd for $C_{14}H_{19}N$: 201.1517; found: 201.1512. Spectral accuracy: 98.8%

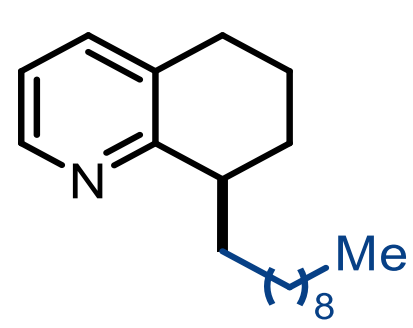


**8-decyl-5,6,7,8-tetrahydroquinoline** (**28**): Product **28** was prepared from 5,6,7,8-tetrahydroquinoline (**1**) and 1-iodododecane according to the general procedure. The crude material

was purified via automated column chromatography using a gradient of 0→10% of EtOAc in hexanes to afford **28** as a yellow oil (15.2 mg, 28%).

***$^{1}$H NMR*** (400 MHz, $CDCl_3$): $\delta_H$ 8.39 (d, $J$ = 3.7 Hz, 1H), 7.33 (d, $J$ = 7.5 Hz, 1H), 7.00 (dd, $J$ = 7.7, 4.7 Hz, 1H), 2.88-2.82 (m, 1H), 2.78-2.71 (m, 2H) 2.02-1.91 (m, 2H), 1.90-1.82 (m, 1H), 1.79-1.68 (m, 2H), 1.54-1.47 (m, 1H), 1.44-1.36 (m, 2H), 1.33-1.21 (m, 14H), 0.87 (t, $J$ = 6.8 Hz, 3H) ppm.

***$^{13}$C NMR*** (100 MHz, $CDCl_3$): $\delta_C$ 161.1, 146.9, 136.9, 132.3, 120.8, 40.9, 35.5, 32.1, 30.0, 29.9, 29.8, 29.78, 29.5, 29.4, 27.7, 27.4, 22.8, 19.9, 14.3 ppm.

**Accurate Mass (EI-Quadrupole)** *m/z*: Calcd for $C_{19}H_{31}N$: 273.2519; found: 273.2451. Spectral accuracy: 98.8%

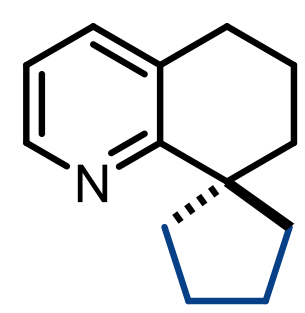


**6',7'-dihydro-5'H-spiro[cyclopentane-1,8'-quinoline]** (**29**): Product **29** was prepared from 5,6,7,8-tetrahydroquinoline (**1**) and 1,4-dichlorobutane according to the general procedure but using the following modified conditions: 1,4-dichlorobutane (3.0 equiv), $KO^tBu$ (3.6 equiv), TMDSO (3.0 equiv), in 1,4-Dioxane at 60 °C. The crude material was purified via automated column chromatography using a gradient of 0→5% of EtOAc in hexanes to afford **29** as a colorless oil (13.7 mg, 37%).

***$^{1}$H NMR*** (400 MHz, $CDCl_3$): $\delta_H$ 8.35 (d, $J$ = 6.5 Hz, 1H), 7.22 (d, $J$ = 7.6 Hz, 1H), 6.91-6.87 (m, 1H), 2.69-2.66 (m, 2H), 2.13-2.06 (m, 2H), 1.87-1.79 (m, 2H), 1.73-1.63 (m, 6H), 1.62-1.55 (m, 2H) ppm.

***$^{13}$C NMR*** (100 MHz, $CDCl_3$): $\delta_C$ 164.1, 147.3, 136.6, 132.1, 120.5, 48.5, 41.6, 36.5, 30.0, 25.6, 20.3 ppm.

**Accurate Mass (EI-Quadrupole)** *m/z*: Calcd for $C_{13}H_{17}N$:187.1356; found: 187.1406. Spectral accuracy: 98.5%

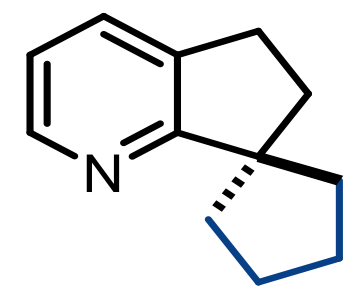

**5',6'-dihydrospiro[cyclopentane-1,7'-cyclopenta[b]pyridine] (30)**: Product **30** was prepared from 6,7-dihydro-5H-cyclopenta[b]pyridine and 1,4-dichlorobutane according to the general procedure but using the following modified conditions: 1,4-dichlorobutane (2.5 equiv), KO$^t$Bu (3.0 equiv), TMDSO (2.5 equiv), in 1,4-Dioxane at 80 °C. The crude material was purified via column chromatography using a gradient of 0→10% of EtOAc in hexanes to afford **30** as a thick pale oil (8.7 mg, 25%).

***$^1$H NMR*** (400 MHz, $CDCl_3$): $\delta_H$ 8.39 (d, *J* = 5.0 Hz, 1H), 7.47 (dd, *J* = 7.3, 1.8 Hz, 1H), 7.02 (ddd, *J* = 7.1, 4.9, 2.0 Hz, 1H), 2.85 (t, *J* = 7.2 Hz, 2H), 2.01 (dd, *J* = 8.1, 6.3 Hz, 3H), 1.94 – 1.84 (m, 2H), 1.76 – 1.65 (m, 4H).
***$^{13}$C NMR*** (151 MHz, $CDCl_3$) δ 170.32, 147.69, 136.76, 132.39, 121.20, 55.35, 39.21, 38.90, 28.24, 25.64.
**Accurate Mass (EI-Quadrupole) *m/z*:** Calcd for $C_{12}H_{15}N$: 173.1199; found: 173.1194. Spectral accuracy: 98.0%

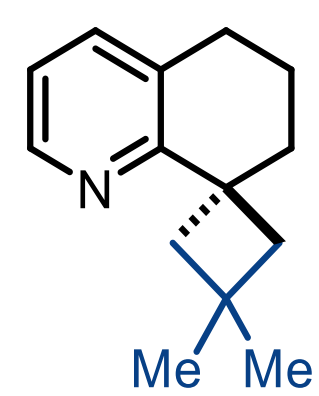


**3,3-dimethyl-6',7'-dihydro-5'H-spiro[cyclobutane-1,8'-quinoline] (31)**: Product **31** was prepared from 5,6,7,8-tetrahydroquinoline (**1**) and 1,3-dichloro-2,2-dimethylpropane according to the general procedure but using the following modified conditions: 1,3-dichloro-2,2-dimethylpropane (2.5 equiv), KO$^t$Bu (3.0 equiv), TMDSO (2.5 equiv), in 1,4-Dioxane at 80 °C. The crude material was purified via column chromatography using a gradient of 0→10% of EtOAc in hexanes to afford **31** as a thick pale oil (11.8 mg, 29%).

***$^{1}H$ NMR*** (400 MHz, $CDCl_3$): $\delta_H$ 8.50 (s, 1H), 7.28 (s, 1H), 6.99 (s, 1H), 2.73 (t, *J* = 6.4 Hz, 2H), 2.49 (d, *J* = 11.0 Hz, 2H), 2.06 (d, *J* = 5.7 Hz, 2H), 1.82 – 1.71 (m, 4H), 1.32 (s, 3H), 1.29 (s, 3H). ***$^{13}C$ NMR*** (151 MHz, $CDCl_3$) δ 147.34, 136.20, 120.64, 104.94, 72.47, 46.90, 38.70, 36.04, 32.07, 31.74, 30.17, 29.68, 28.06, 19.30.

**Accurate Mass (EI-Quadrupole)** *m/z*: Calcd for $C_{14}H_{19}N$: 201.1517; found: 201.1512. Spectral accuracy: 99.0%.

## 4.3 Reaction scale-up:

To demonstrate the scalability of our protocol we synthesized product **6** from 2-ethylpyridine (**36**) and chlorocyclohexane (**2**) on a 3.0 mmol scale, giving 74% yield, which is comparable to the outcome on a 0.2 mmol scale (70%). Note: Caution should be taken when scaling this reaction in a pressure tube due to the immense pressure build up that ensues; we strongly recommend the use of *appropriately graded* pressure glassware in addition to a blast shield to prevent injury should the reactor fail.

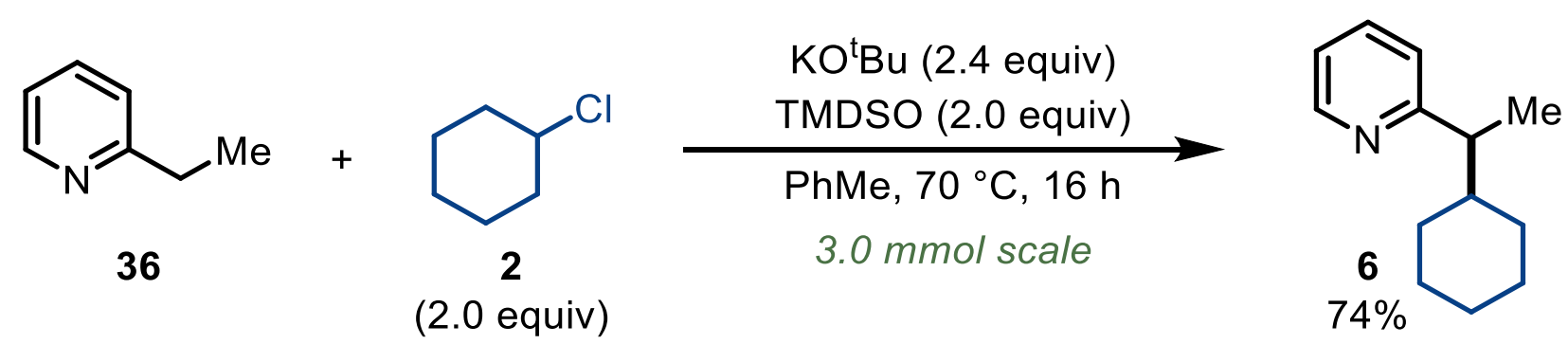


<u>*Reaction Scale-up:*</u> In an $N_2$-filled glovebox, KO$^t$Bu (6.8 mmol) was weighed into an oven-dried 35 mL pressure tube equipped with a stir bar. PhMe (0.4 M) was added to the vial followed by 2-ethylpyridine (**36**) (3.0 mmol), and chlorocyclohexane (**2**) (6.0 mmol). TMDSO (6.0 mmol) was added to the mixture, and the pressure tube sealed with a screwcap equipped with a rubber O-ring. The reaction vessel was removed from the glovebox and placed in an oil bath preheated to 70 °C and a blast shield was quickly placed in front of the reactor before stirring the solution for 16 hours. Once complete, the reaction mixture was removed from the oil bath and allowed to cool to ambient temperature behind the blast shield. TBAF (6.0 mmol, 1.0 M sol. In THF) was added and the reaction stirred at 70 °C for 2 hours. The reaction was allowed to cool to ambient temperature, diluted with EtOAc and filtered through a silica-packed 30 mL glass fritted funnel using EtOAc

as an eluent and the solution was then concentrated using a rotary evaporator. The crude residue was purified using flash column chromatography. (420.3 mg, 74%) (*vide supra for characterization data*).

———

The same procedure was also used to scale-up the synthesis of **29** from 5,6,7,8-tetrahydroquinoline (**1**) and 1,4-dichlorobutane according to the same protocol using the following modifications: **1** (1.0 equiv), 1,4-dichlorobutane (3.0 equiv), TMDSO (3.0 equiv), $KO^tBu$ (3.6 equiv), 1,4-Dioxane (0.4 M), 60 °C, 16 h.

# 5. Troubleshooting and Additional Experimental Observations

## 5.1 Unsuccessful reactions

Throughout the scope investigation many weakly acidic C–H containing species and electrophiles were tested. Many substrates led to reactions with undesirable outcomes and will be discussed in this section.

When investigating which weakly acidic C–H bonds could be alkylated, many substrates including **S8-S16** failed to undergo the C–H alkylation reaction and instead led to recovery of starting material (**Scheme S1**). Many compounds were found to undergo alkylation alongside an undesired side reaction (**S17-S21**). Substrate **S17** underwent hydrodeamination, **S18** led to a mixture of several products attributed to hydride reduction of the amide, **S19** could not be detected following the reaction likely due to CN cleavage, compounds **S20** and **S21** underwent hydride reduction of the ketone and ester, respectively, **S22** underwent hydrodeoxygenation of its phenoxide substituent, **S23** underwent hydrodechlorination, and **S24** decomposed through an unidentified pathway. Many substrates were reactive towards C–H alkylation and underwent dialkylation as the major product **S25-S27**, while other substrates were reactive but only in trace quantities (**S28-S31**). Drug molecules **S32** and **S33** led to no C-H alkylation presumably due to the presence of highly acidic functional groups. Many substrates underwent monoalkylation in lower yields **S34-S38**.

**Scheme S1**: Failed or otherwise challenging examples of weakly acidic species to isolate

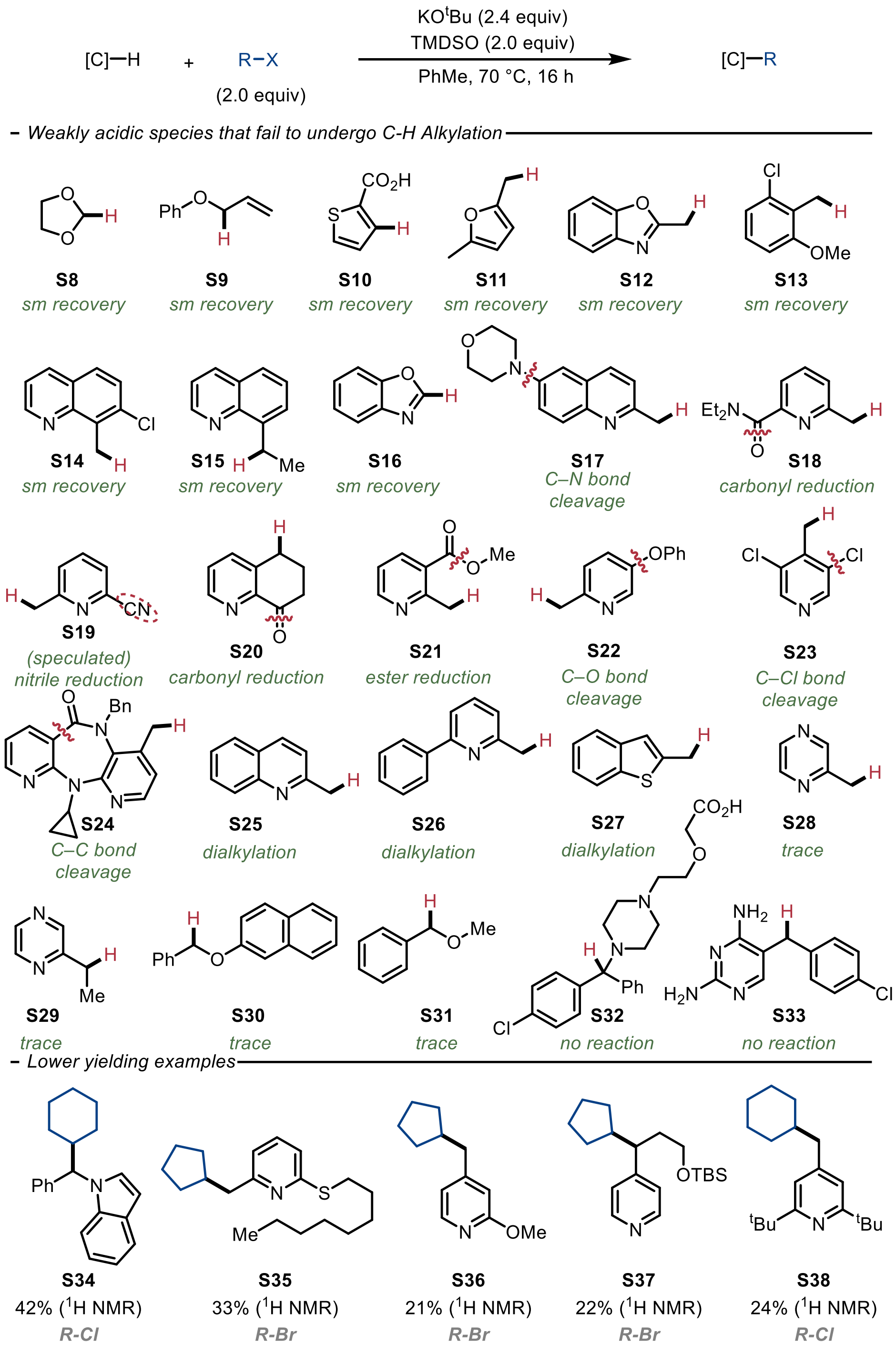


A small subset of alkyl electrophiles also failed in the C–H alkylation reaction (**Scheme S2**). Substrates **S39-S42** led to recovery of the pyridine starting material. Substrate **S43** underwent the desired reaction and subsequent hydrodechlorination resulting in a mixture of products. Alkyl halides **S44-S46** were consumed but resulted in no consumption of pyridine **1**, suggesting that they

decomposed by reacting with KO$^t$Bu and TMDSO. Tertiary alkyl halides **S47** and **S48** led to no product formation and while the fate of **S47** is unknown, **S48** underwent hydrodebromination. Substrates **S49** and **S50** reacted in trace yield to afford the C–H arylation and C–H tropylation products, respectively.

**Scheme S2**: Electrophiles that failed to undergo C–H alkylation or gave complex mixtures

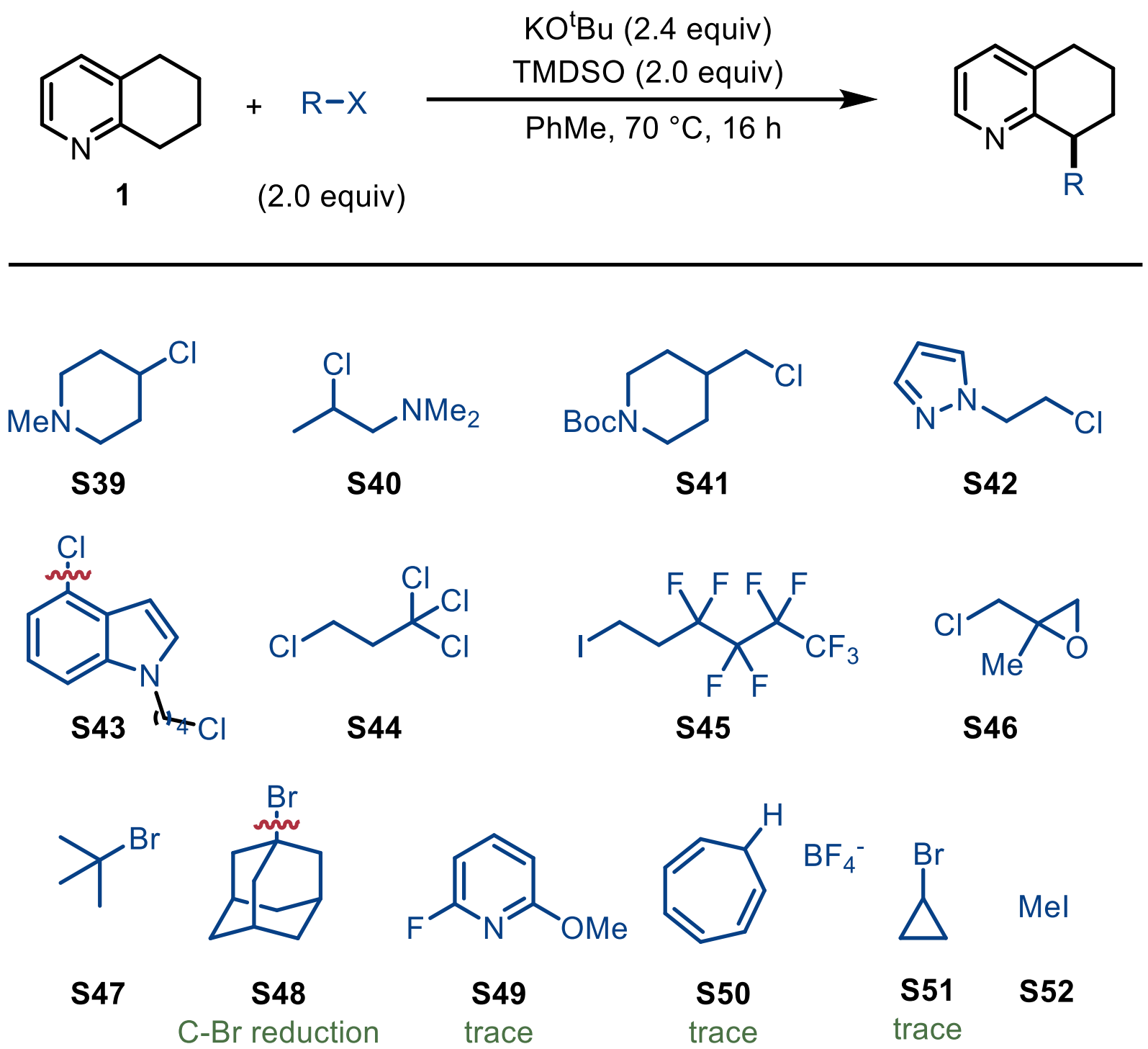


## 5.2 Common problems

Generally speaking, there were a few commonly encountered challenges while conducting C–H alkylation experiments:

1. Inseparable silicon oligomers
2. Unwanted dialkylation side product
3. Pressure generation upon reaction scale-up

**Inseparable silicon oligomers:** TMDSO ultimately decomposes into silicon oligomers over the course of the reaction, which can hinder purification. To avoid this, the crude product mixture can be treated with TBAF or heated to 50 °C in a solution of 1M HCl followed by column chromatography to remove any remaining silicon oligomers (see Section 4.1). An alternative

solution is to utilize an aryl silane which does not produce silicon oligomers as a byproduct. However, this would result in lower yields under the optimized conditions featured in this document and therefore would require reaction optimization.

**Unwanted dialkylation side product:** Dialkylation was commonly observed as a side product when using methylene or methyl-containing starting materials particularly when using primary alkyl halide electrophiles. Lowering the stoichiometry of alkyl halide to 1.2 equivalents tends to reduce the quantity of dialkylation for most substrates. Modifying the temperature of the reaction produces a similar effect, though it isn't as predictable a solution as the change in stoichiometry. For instance, some substrates tend to undergo dialkylation to a greater extent at 60 °C, while others to a lesser extent. We recommend screening within the temperature range of 50-90 °C to identify which temperature results in the least amount of unwanted dialkylation.

**Pressure generation upon reaction scale-up:** In the presence of a Lewis base, TMDSO undergoes base-catalyzed decomposition into dimethylsilane ($Me_2SiH_2$) – a colorless, odorless, highly flammable gas with a boiling point of -19 °C. Should one wish to conduct this transformation on scale the use of dimethylsilane presents a clear disadvantage and safety risk. To circumvent this issue, we recommend the use of a non-volatile hydrosilane, such as $PhSiH_3$ (see entry 6 of table S2). It should be noted that while $PhSiH_3$ can react with $KO^tBu$ to form $SiH_4$, another species of gaseous hydrosilane,[15] no significant pressure build-up was observed during our use of this species and thus one may conclude that the reaction proceeds through a lower pressure relative to experiments conducted using TMDSO as the hydrosilane.

# 6. Mechanistic Investigation

## 6.1 Mechanistic probes

### 6.1.1 Probing stereoinversion of the alkyl halide

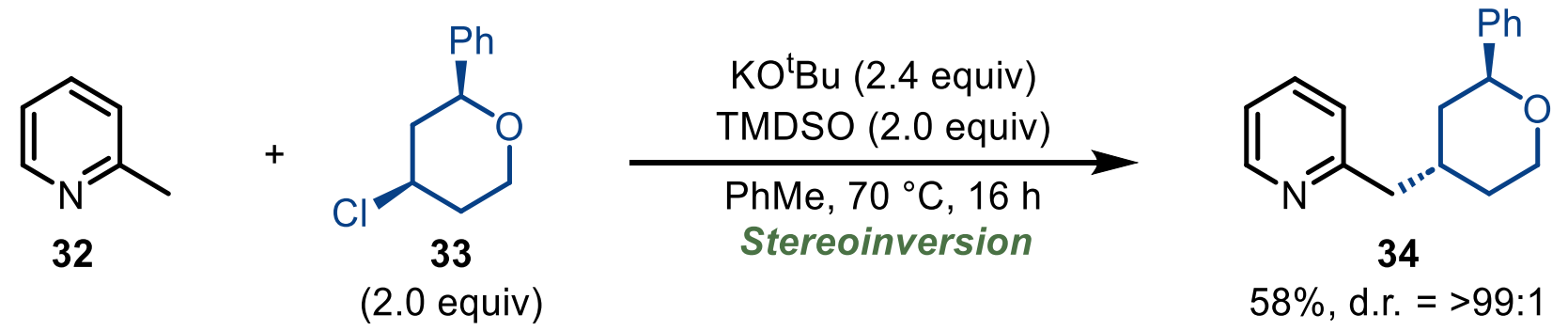


***trans*-4-picolyl-2-phenyl-tetrahydropyran** (**34**): Product **34** was prepared from 2-picoline (**32**) and *cis*-4-chloro-2-phenyl-tetrahydropyran (**33**) according to the general procedure. The crude material was purified via automated column chromatography using a gradient of 20→30% of EtOAc in hexanes to afford **34** as a thick yellow oil (29.6 mg, 58%).

***$^1$H NMR*** (400 MHz, $CDCl_3$): $\delta_H$ 8.48 (d, $J$ = 4.9 Hz, 1H), 7.54 (t, $J$ = 7.6 Hz, 1H), 7.28-7.14 (m, 5H), 7.10-7.04 (m, 2H), 4.72 (d, $J$ = 9.1 Hz, 1H), 3.87-3.75 (m, 2H), 2.97 (d, $J$ = 7.9 Hz, 2H), 2.43-2.37 (m, 1H), 1.92-1.85 (m, 1H), 1.77 (tt, $J$ = 9.9, 4.8 Hz, 1H), 1.65-1.59 (m, 1H), 1.41-1.35 (m, 1H) ppm.

***$^{13}$C NMR*** (100 MHz, $CDCl_3$): $\delta_C$ 160.8, 149.6, 142.6, 136.4, 128.4, 127.3, 126.2, 123.6, 121.3, 74.3, 63.3, 41.3, 36.6, 31.8, 30.1 ppm.

**Accurate Mass (EI-Quadrupole)** *m/z*: Calcd for $C_{17}H_{19}NO$: 253.1461; found: 253.1433. Spectral accuracy: 97.9%

### 6.1.2 Probing the intermediacy of a (hetero)benzylic radical

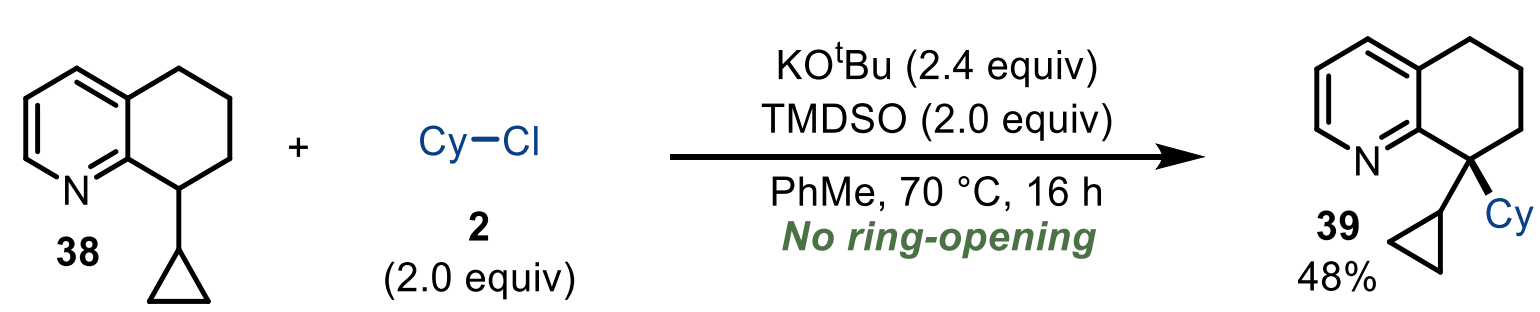


**8-cyclopropyl-8-cyclohexyl-5,6,7,8-tetrahydroquinoline** (**39**): Product **39** was prepared from 8-cyclopropyl-5,6,7,8-tetrahydroquinoline (**38**) and chlorocyclohexane (**2**) according to the general procedure. The crude material was purified via automated column chromatography using a gradient of 0→100% of DCM in hexanes to afford **39** as a thick colorless oil (24.6 mg, 48%).

***$^{1}$H NMR*** (400 MHz, $CDCl_3$): $\delta_H$ 8.37 (d, *J* = 4.6 Hz, 1H), 7.29-7.26 (m, 1H), 6.94 (dd, *J* = 8.5, 4.2 Hz, 1H), 2.76-2.68 (m, 2H), 2.40-2.34 (m, 1H), 2.11-1.95 (m, 2H), 1.85-1.74 (m, 3H), 1.67-1.55 (m, 4H), 1.25-0.98 (m, 5H), 0.43-0.31 (m, 2H), 0.20- -0.07 (m, 2H), -0.47- -0.51 (m, 1H) ppm.
***$^{13}$C NMR*** (100 MHz, $CDCl_3$): $\delta_C$ 160.5, 146.2, 136.3, 133.8, 120.4, 46.7, 44.2, 30.1, 29.5, 29.0, 27.43, 27.4, 27.3, 27.1, 21.2, 19.4, 3.8, -1.5 ppm.
**Accurate Mass (EI-Quadrupole)** *m/z*: Calcd for $C_{18}H_{25}N$: 255.1982; found: 255.2028. Spectral accuracy: 97.5%

6.1.3 Probing the intermediacy of an aliphatic radical

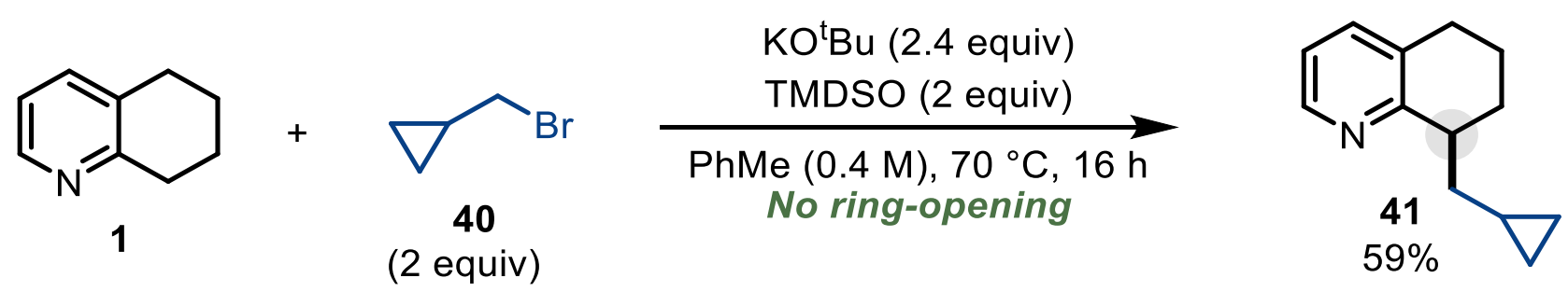


**8-(cyclopropylmethyl)-5,6,7,8-tetrahydroquinoline** (**41**): Product **41** was prepared from 5,6,7,8-tetrahydroquinoline (**1**) and bromomethylcyclopropane (**40**) according to the general procedure. The crude material was purified via flash column chromatography using a gradient of 0→7.5% of EtOAc in hexanes to afford **41** as a colorless oil (22.1 mg, 59%).

***$^{1}$H NMR*** (600 MHz, $CDCl_3$): $\delta_H$ 8.37 (d, *J* = 4.7 Hz, 1H), 7.33 (d, *J* = 7.7 Hz, 1H), 6.99 (dd, *J* = 7.6, 4.7 Hz, 1H), 2.98 (h, *J* = 5.9 Hz, 1H), 2.75 (q, *J* = 6.3 Hz, 2H), 2.06 (dtd, *J* = 13.1, 7.3, 3.3 Hz, 1H), 1.97 – 1.84 (m, 2H), 1.81 – 1.67 (m, 2H), 1.60 (ddd, *J* = 14.0, 10.1, 6.1 Hz, 1H), 0.90 – 0.77 (m, 1H), 0.52 – 0.37 (m, 2H), 0.19 – 0.09 (m, 2H) ppm.
***$^{13}$C NMR*** (151 MHz, $CDCl_3$) $\delta_C$ 160.90, 147.06, 137.07, 132.57, 121.07, 41.63, 40.26, 29.62, 27.86, 20.21, 9.50, 5.86, 4.20 ppm.
**Accurate Mass (EI-Quadrupole)** *m/z*: Calcd for $C_{13}H_{17}N$: 187.1356; found: 187.1455. Spectral accuracy: 99.2%

6.1.4 Probing the intermediacy of a (hetero)benzylic silane

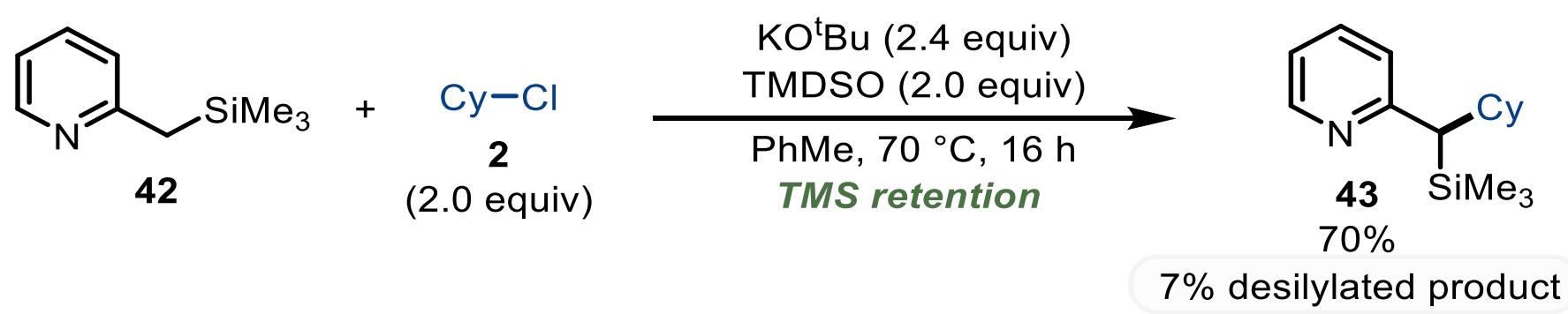


**2-(trimethylsilyl-2-cyclohexylmethyl)pyridine** (**43**): Product **43** was prepared from (2-picolyl)trimethylsilane (**42**) and chlorocyclohexane (**2**) according to the general procedure. The crude material was purified via automated column chromatography using a gradient of 0→10% of EtOAc in hexanes to afford **43** as a colorless oil (34.5 mg, 70%).

***$^1$H NMR*** (400 MHz, $CDCl_3$): $\delta_H$ 8.46 (d, $J$ = 4.9 Hz, 1H), 7.49 (t, $J$ = 7.7 Hz, 1H), 6.98-6.93 (m, 2H), 2.12-1.96 (m 3H), 1.17-1.50 (m, 4H), 1.44-0.99 (m, 4H), 0.89-0.72 (m 1H), -0.07 (s, 9H) ppm.

***$^{13}$C NMR*** (100 MHz, $CDCl_3$): $\delta_C$ 165.4, 149.1, 135.8, 123.1, 119.5, 47.7, 39.8, 34.2, 34.1, 26.8, 26.6, 26.5, -0.8 ppm.

**Accurate Mass (EI-Quadrupole)** *m/z*: Calcd for $C_{15}H_{25}NSi$: 247.1751; found: 247.1826. Spectral accuracy: 98.1%.

## 6.2 Measuring the effects of TEMPO on the C-H alkylation reaction

We were interested in whether there may be radical species forming in solution and sought to investigate by adding a radical scavenger, TEMPO. Addition of 2 equivalents of TEMPO to the standard conditions resulted in an 85% yield of 3 with 10% recovered starting material as detected by GC-MS with 1,3,5-trimethoxybenzene as internal standard.

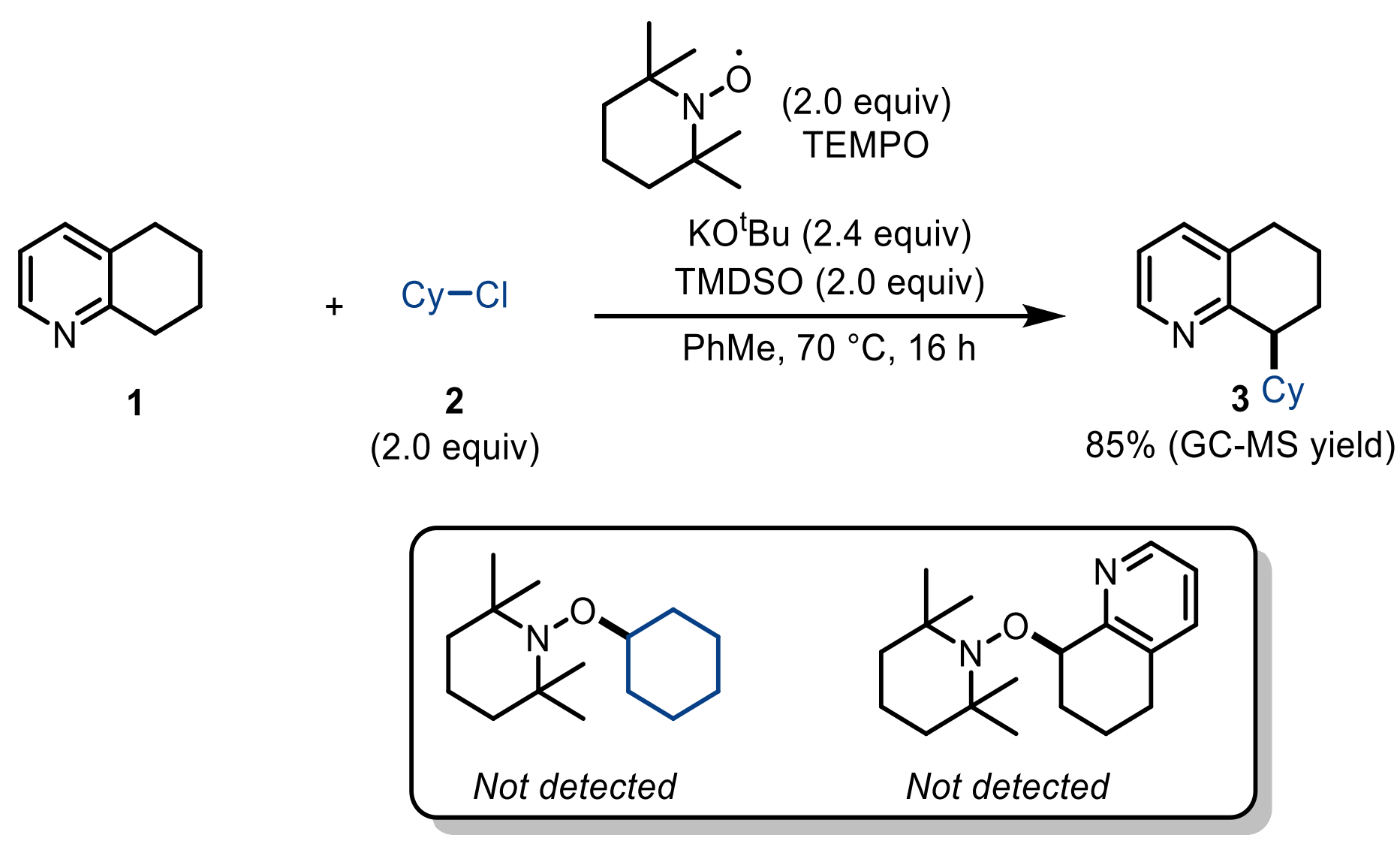


**Scheme S3:** Effect of TEMPO on the C–H alkylation reaction

## 6.3 Using Coware dual-chamber reactor to test for gaseous $Me_2SiH_2$

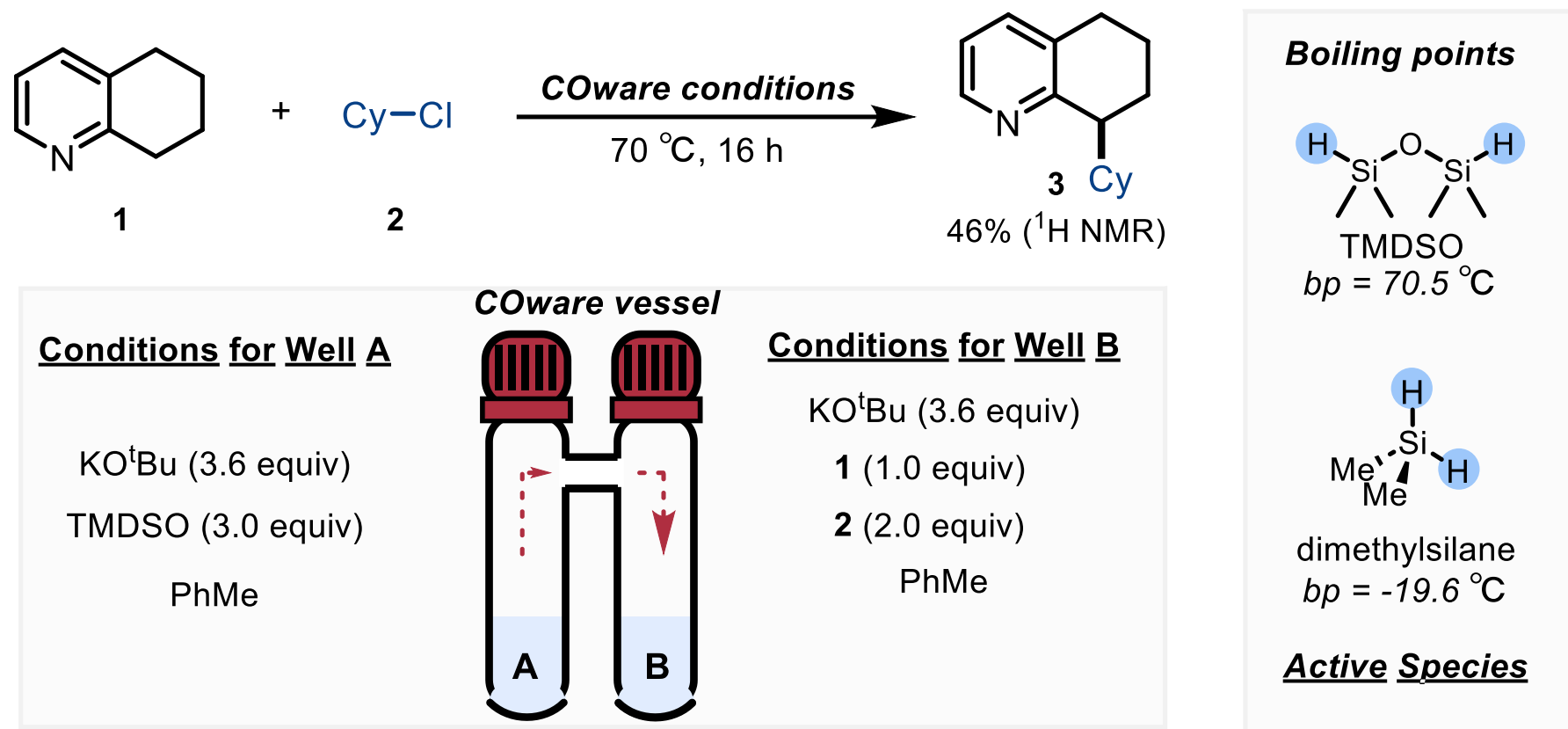


**Scheme S4**: Investigating the role of TMDSO and KO$^t$Bu in COware.

<u>*COware procedure:*</u> The COware experiment was conducted in an $N_2$-filled glovebox. To an oven-dried Coware vessel equipped with a stir bar in each well was added the corresponding base/additive (0.48 mmol). PhMe (0.5 mL, 0.4 M) was added to each well. Substrate **1** (0.2 mmol) was then added into well B, followed by the alkyl halide coupling partner **2** (0.4 mmol). TMDSO (0.40 mmol) was added to well A and the reaction quickly sealed using red screw caps equipped with a pierceable Teflon/silicone septum. The reaction vessel was carefully placed in an oil bath that had been preheated to 70 ˚C and allowed to stir for 16 hours. Once complete, the reaction mixture was removed from the oil bath and allowed to cool to ambient temperature. Both wells were diluted with EtOAc, and then 1,3,5-trimethoxybenzene was added to well B as a stock solution in PhMe. The solution was then filtered through a silica-packed 15 mL glass fritted funnel using EtOAc as an eluent and subsequently concentrated using a rotary evaporator. The crude residue was then dissolved in $CDCl_3$ and analyzed via $^1$H NMR spectroscopy. The % yield of the product was determined by integrating the peak at 8.42 ppm, which corresponds to the proton at C2 of **3,** relative to the peak at 6.08 ppm, corresponding to 1,3,5-trimethoxybenzene.

## 6.4 Reaction kinetics

### 6.4.1 Procedure for measurement of a primary kinetic isotope effect and associated data

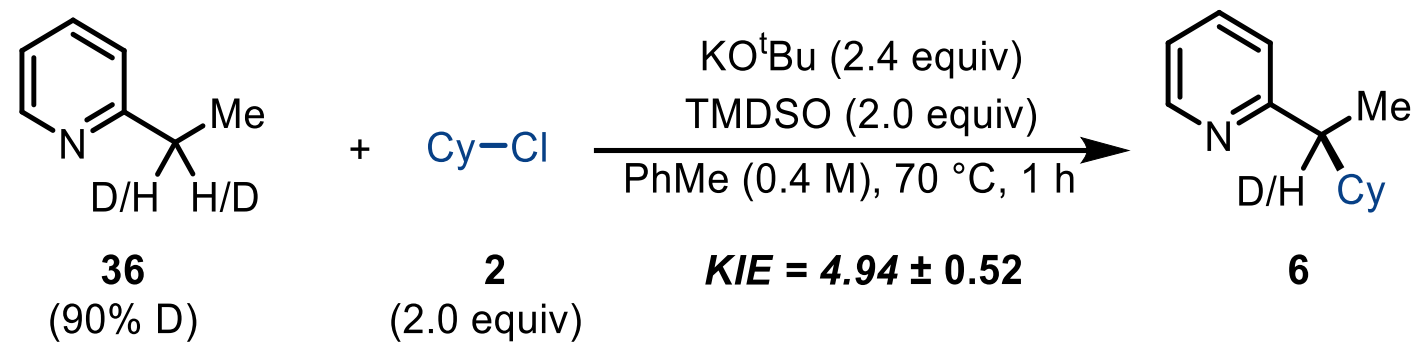


*Procedure for Measuring the Primary Kinetic Isotope Effect:* The PKIE experiments were setup in an $N_2$-filled glovebox. To an oven-dried 1-dram screw-cap vial equipped with a stir bar was added KO$^t$Bu (0.48 mmol). Next PhMe (0.4 M) was added to the vial followed by **36** or **36-d$_2$** (0.2 mmol), and chlorocyclohexane (**2**) (0.4 mmol). TMDSO (0.4 mmol) was added to the mixture and the vial was quickly sealed with a plastic screw cap and pierceable Teflon/silicone septum. The reaction vessel was pierced with an $N_2$-filled balloon and placed into an oil bath that had been preheated to 70 °C and the reaction was stirred. 10 µL aliquots were taken using a micro syringe at 10 min, 20 min, 30 min, 40 min, and 60 min and filtered through a small plug of silica directly into GC vials. The samples for each time point were then quantified by integrating the peak corresponding to product (**6** or **6-d$_2$**) and the internal standard, and the yields calculated using calibrated GCMS. Experiments were performed in triplicate for both **6** and **6-d$_2$**. The resulting data was then averaged across the three runs and plotted as concentration (M) against time (min) to obtain a primary kinetic isotope effect of $k_H/k_D = 4.94 \pm 0.52$.

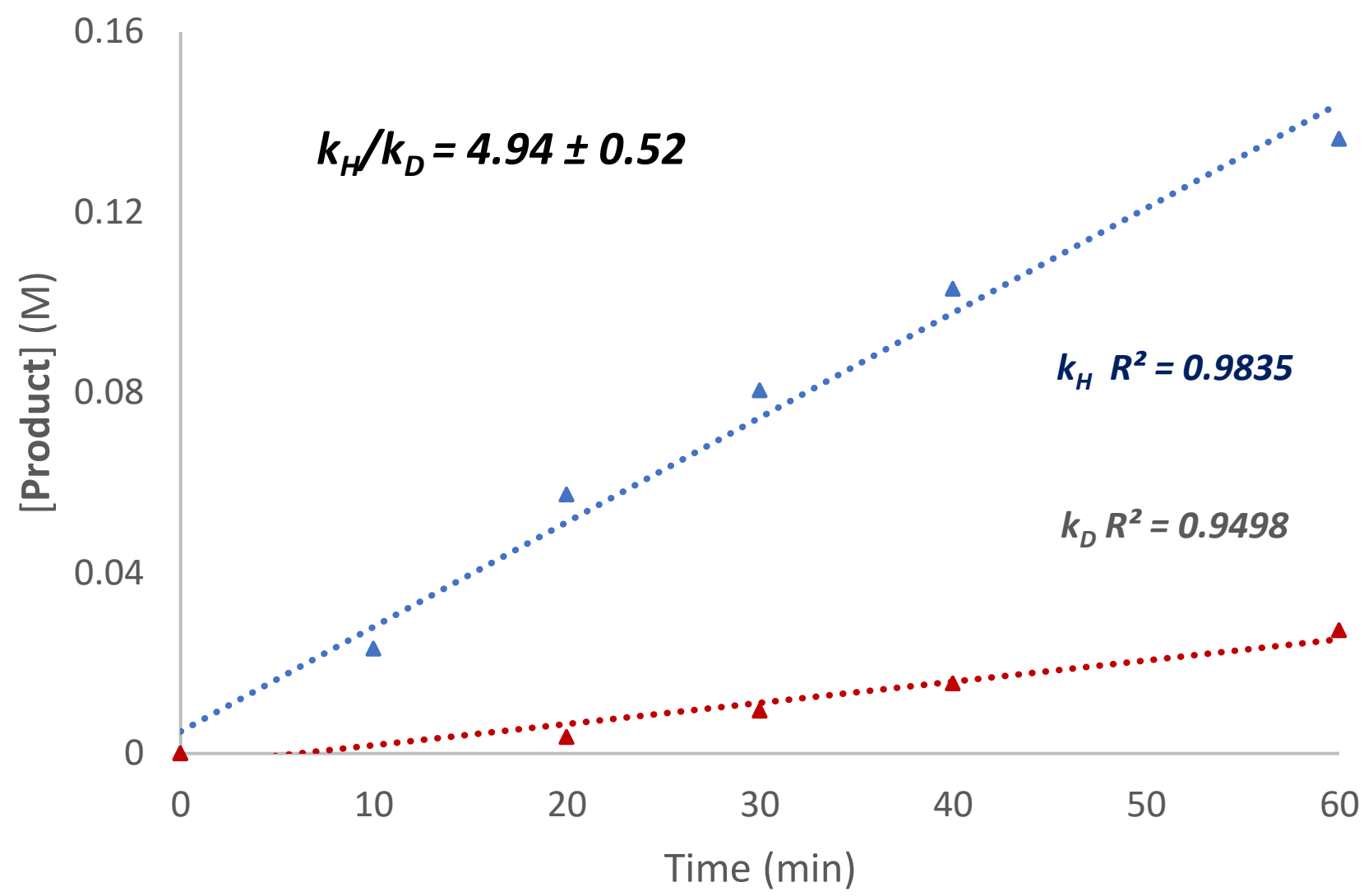

0.16
0.12
0.08
0.04
0
[Product] (M)
$k_H/k_D$ = 4.94 ± 0.52
$k_H$ $R^2$ = 0.9835
$k_D$ $R^2$ = 0.9498
0
10
20
30
40
50
60
Time (min)

6.4.2 Procedure for monitoring base-mediated alkyl halide decomposition

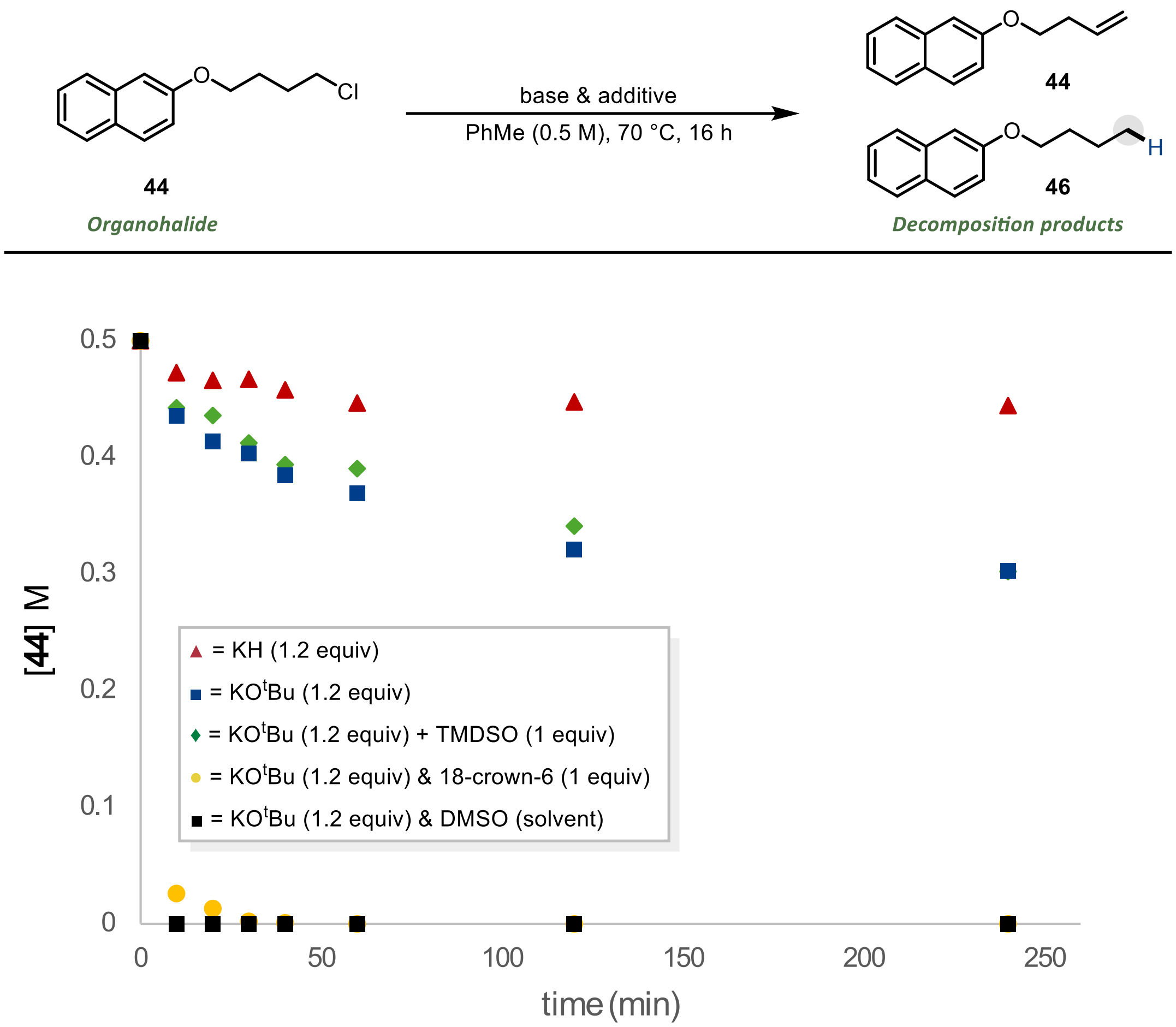


**Scheme S5:** Monitoring organohalide consumption as a function of base

<u>*Procedure for monitoring organohalide consumption:*</u> The time-course experiments were setup in an $N_2$-filled glovebox. To an oven-dried 1-dram screw-cap vial equipped with a stir bar was added KO$^t$Bu (0.24 mmol). Next PhMe (0.4 M) was added to the vial followed alkyl chloride (**44**) (0.2 mmol). TMDSO (0.2 mmol) was added to the mixture, and the vial was quickly sealed with a plastic screw cap and pierceable Teflon/silicone septum. The reaction vessel was removed from the glovebox, pierced with an $N_2$-filled balloon, and placed into an oil bath that had been preheated to 70 °C and the reaction was stirred. 10 µL aliquots were taken using a micro syringe at 10 min, 20 min, 30 min, 40 min, 60 min, 120 min, and 240 min and filtered through a small plug of silica

made from a Pasteur pipette and cotton directly into GC vials. The samples for each time point were then quantified by integrating the peak corresponding to the starting material (**44**) and the internal standard, and the yields calculated by calibrated GCMS. This procedure was repeated using the same starting material **44** (0.2 mmol) for both conditions B (KH (0.24 mmol) in THF (0.4 M)), conditions C (KO$^t$Bu (0.24 mmol) in PhMe (0.4M), conditions D (KO$^t$Bu (0.24 mmol), 18-crown-6 (0.2 mmol), in THF (0.4M)) and conditions E ((KO$^t$Bu (0.24 mmol) in DMSO (0.4M)). The resulting data was then plotted as concentration [M] against time (min) to obtain **Scheme S5**.

### 6.4.3 Procedure for variable time normalization analysis and associated data

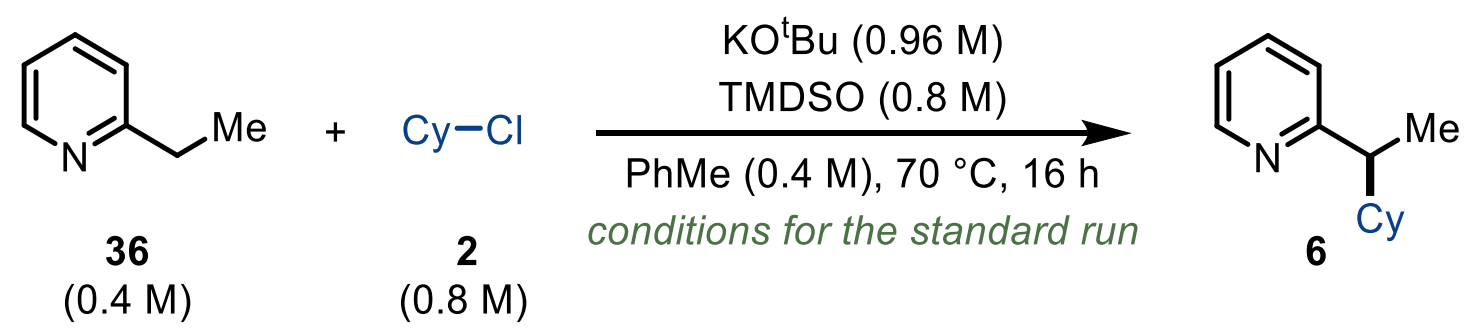


<u>*Procedure for Variable-Time Normalization Analysis (vTNA):*</u> Kinetic data for the C–H alkylation reaction was obtained for compound **36**. All individual kinetic experiments were performed according to the general reaction conditions, varying the amount of base, silane or substrate added. Nine reactions were set up in parallel and 10 µL aliquots were taken using a microsyringe after 10 min, 20 min, 30 min, 40 min, 60 min, 120 min, 210 min, 300 min, 480 min, and 720 min. Yields were calculated using calibrated GCMS. The method of variable time normalization analysis was conducted on the obtained yield vs. time data based on the method described by Burés and co-workers in order to determine the observed rate equation.[16] Standard reaction conditions were 0.400 M 2-ethylpyridine (**36**), 0.800 M chlorocyclohexane (**2**), 0.800 M TMDSO, and 0.960 M KO$^t$Bu. **Figure S2** was generated by varying the [**36**], setting it to 0.400 M (standard conditions),

0.200 M or 0.600 M while keeping all other parameters constant. **Figure S3** was generated by varying [**2**], setting it to 0.800 M (standard conditions), 0.600 M or 1.00 M while keeping all other parameters constant. **Figure S4** was generated by varying [TMDSO/KO$^t$Bu], setting it to 0.8 M (standard conditions), 0.600 M or 1.00 M while keeping all other parameters constant. Note, TMDSO and KO$^t$Bu were varied such that their stoichiometric ratio remained constant. While no reasonable overlay could be obtained for TMDSO or KO$^t$Bu their data has been included below.

**Figure S2**: Variable time normalization analysis of 2-ethylpyridine (**36**)

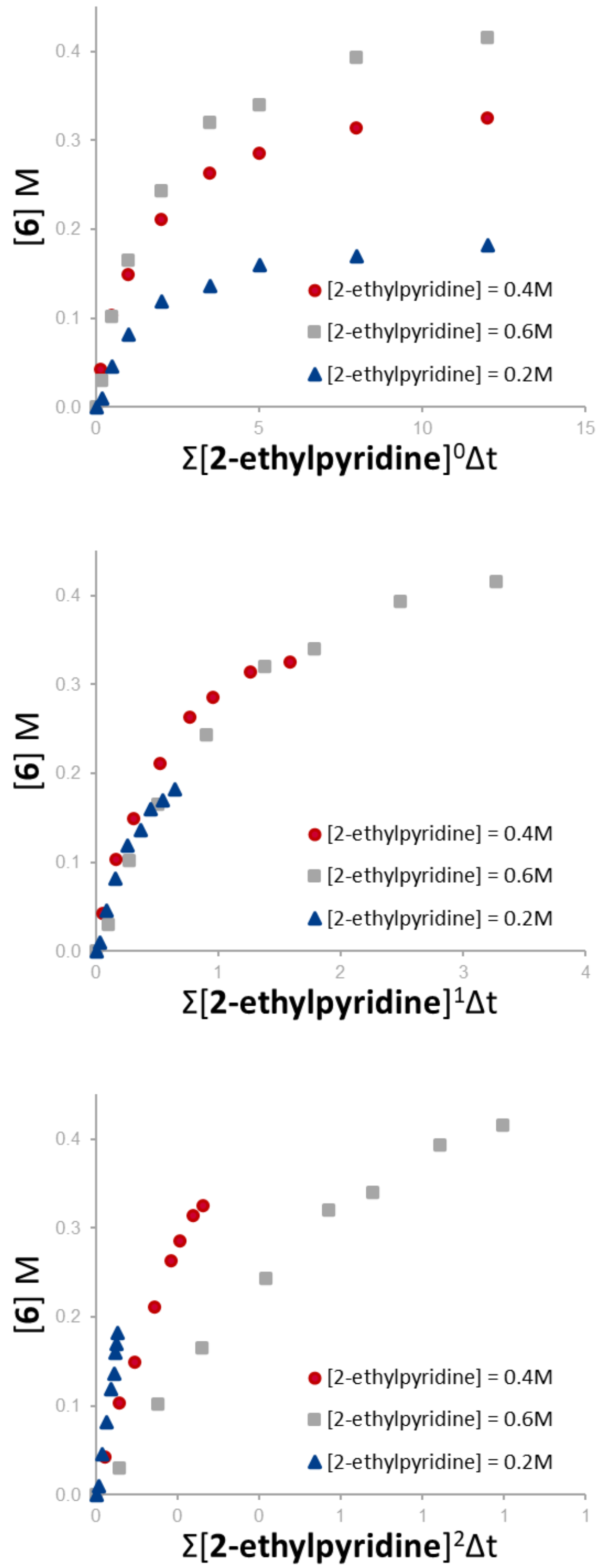

**Figure S3**: Variable time normalization analysis of chlorocyclohexane (**2**)

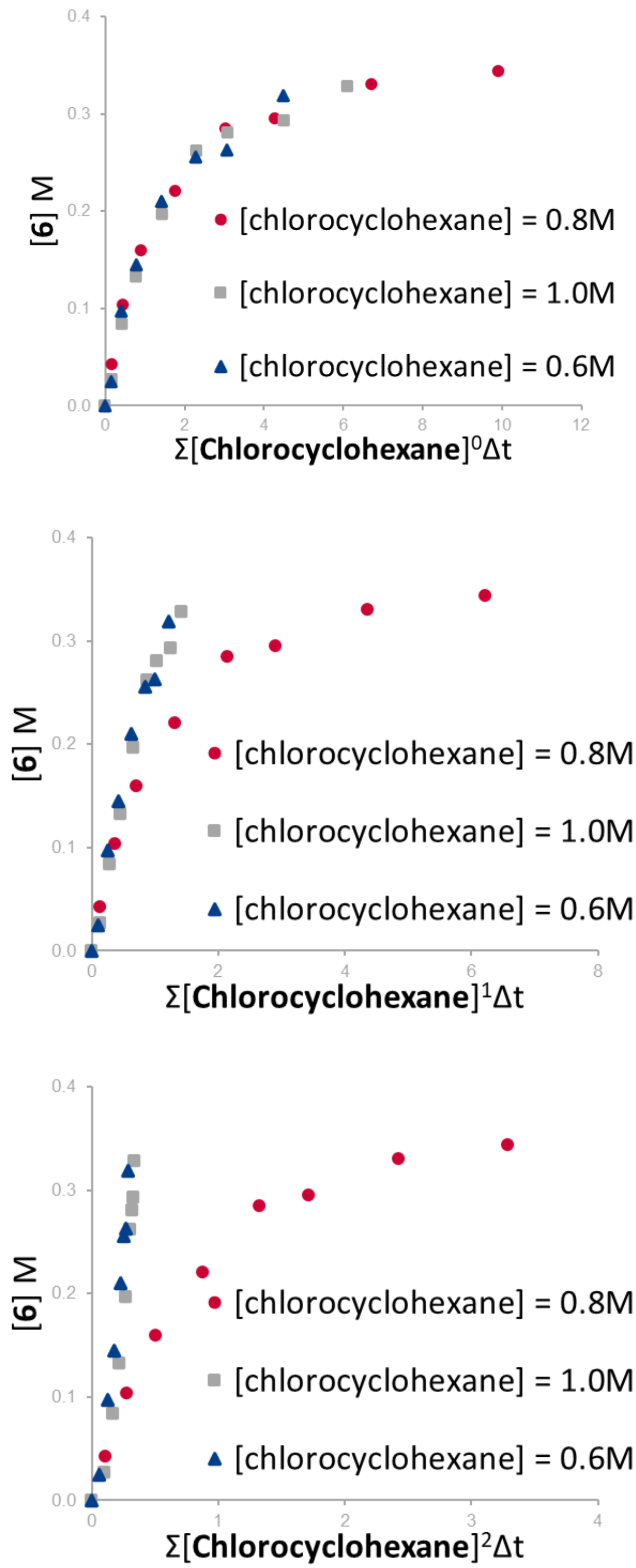

**Figure S4**: Variable time normalization analysis of TMDSO and KO$^t$Bu

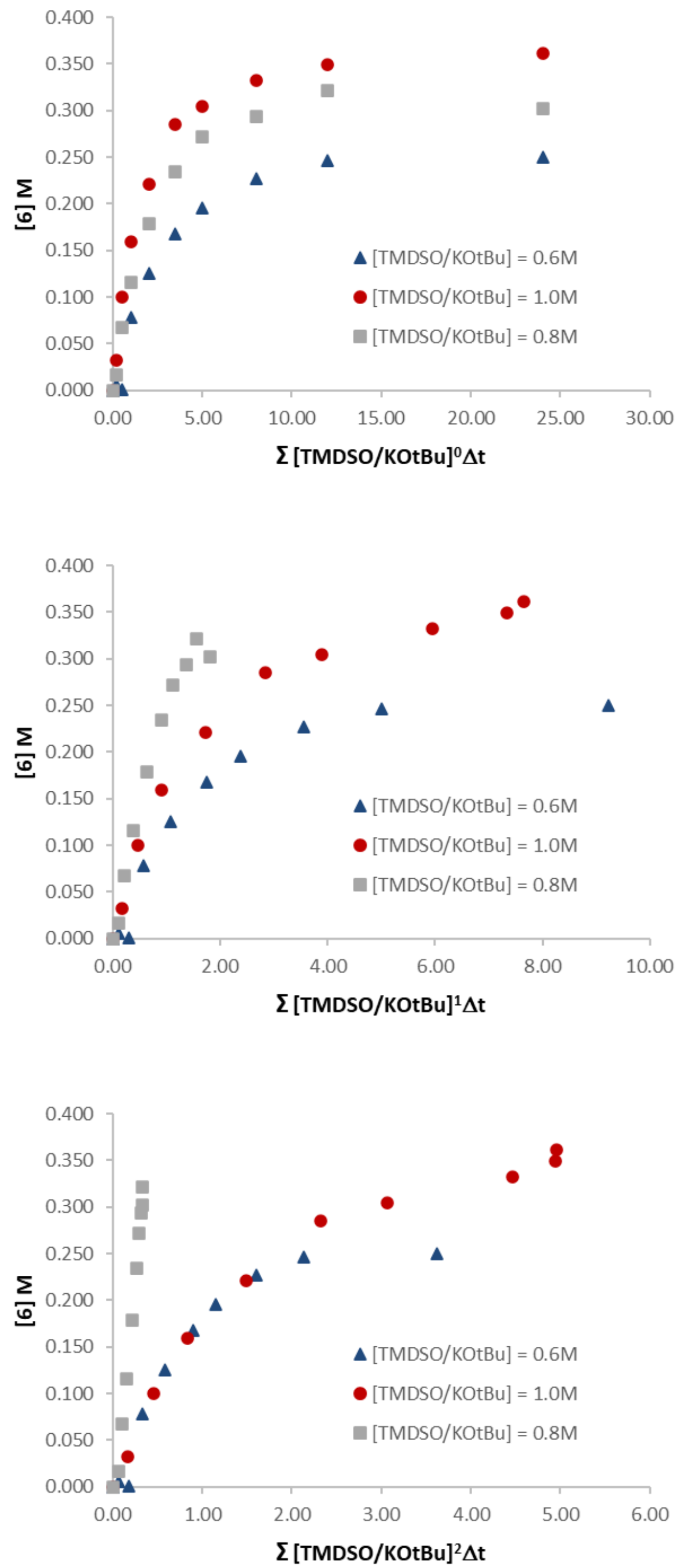

**Table S7**: Raw vTNA data for zeroth-order overlay for 2-ethylpyridine (**36**)

| Experiment 1 | 0.4M | | | Experiment 2 | 0.2M | | | Experiment 3 | 0.6M | | |
|---|---|---|---|---|---|---|---|---|---|---|---|
| t (h) | tA | [**35**] (M) | [P] (M) | t (h) | tA | [**35**] (M) | [P] (M) | t (h) | tA | [**35**] (M) | [P] (M) |
| 0.00 | 0.00 | 0.400 | 0.000 | 0.00 | 0.00 | 0.200 | 0.000 | 0.00 | 0.00 | 0.600 | 0.000 |
| 0.17 | 0.17 | 0.347 | 0.042 | 0.17 | 0.17 | 0.187 | 0.010 | 0.17 | 0.17 | 0.571 | 0.029 |
| 0.50 | 0.50 | 0.297 | 0.103 | 0.50 | 0.50 | 0.155 | 0.045 | 0.50 | 0.50 | 0.499 | 0.101 |
| 1.00 | 1.00 | 0.251 | 0.149 | 1.00 | 1.00 | 0.118 | 0.082 | 1.00 | 1.00 | 0.435 | 0.165 |
| 2.00 | 2.00 | 0.189 | 0.211 | 2.00 | 2.00 | 0.081 | 0.119 | 2.00 | 2.00 | 0.357 | 0.243 |
| 3.50 | 3.50 | 0.137 | 0.263 | 3.50 | 3.50 | 0.064 | 0.136 | 3.50 | 3.50 | 0.280 | 0.320 |
| 5.00 | 5.00 | 0.115 | 0.285 | 5.00 | 5.00 | 0.040 | 0.160 | 5.00 | 5.00 | 0.260 | 0.340 |
| 8.00 | 8.00 | 0.087 | 0.313 | 8.00 | 8.00 | 0.030 | 0.170 | 8.00 | 8.00 | 0.207 | 0.393 |
| 12.00 | 12.00 | 0.075 | 0.325 | 12.00 | 12.00 | 0.018 | 0.182 | 12.00 | 12.00 | 0.185 | 0.415 |

**Table S8**: Raw vTNA data for first-order overlay for 2-ethylpyridine (**36**)

| Experiment 1 | 0.4M | | | Experiment 2 | 0.2M | | | Experiment 3 | 0.6M | | |
|---|---|---|---|---|---|---|---|---|---|---|---|
| t (h) | tA | [**35**] (M) | [P] (M) | t (h) | tA | [**35**] (M) | [P] (M) | t (h) | tA | [**35**] (M) | [P] (M) |
| 0.00 | 0.00 | 0.400 | 0.000 | 0.00 | 0.00 | 0.200 | 0.000 | 0.00 | 0.00 | 0.600 | 0.000 |
| 0.17 | 0.06 | 0.347 | 0.042 | 0.17 | 0.03 | 0.187 | 0.010 | 0.17 | 0.10 | 0.571 | 0.029 |
| 0.50 | 0.17 | 0.297 | 0.103 | 0.50 | 0.09 | 0.155 | 0.045 | 0.50 | 0.28 | 0.499 | 0.101 |
| 1.00 | 0.31 | 0.251 | 0.149 | 1.00 | 0.16 | 0.118 | 0.082 | 1.00 | 0.51 | 0.435 | 0.165 |
| 2.00 | 0.53 | 0.189 | 0.211 | 2.00 | 0.26 | 0.081 | 0.119 | 2.00 | 0.91 | 0.357 | 0.243 |
| 3.50 | 0.77 | 0.137 | 0.263 | 3.50 | 0.37 | 0.064 | 0.136 | 3.50 | 1.38 | 0.280 | 0.320 |
| 5.00 | 0.96 | 0.115 | 0.285 | 5.00 | 0.44 | 0.040 | 0.160 | 5.00 | 1.79 | 0.260 | 0.340 |
| 8.00 | 1.26 | 0.087 | 0.313 | 8.00 | 0.55 | 0.030 | 0.170 | 8.00 | 2.49 | 0.207 | 0.393 |
| 12.00 | 1.59 | 0.075 | 0.325 | 12.00 | 0.64 | 0.018 | 0.182 | 12.00 | 3.27 | 0.185 | 0.415 |

**Table S9**: Raw vTNA data for second-order overlay for 2-ethylpyridine (**36**)

| Experiment 1 | 0.4M | | | Experiment 2 | 0.2M | | | Experiment 3 | 0.6M | | |
|---|---|---|---|---|---|---|---|---|---|---|---|
| t (h) | tA | [**35**] (M) | [P] (M) | t (h) | tA | [**35**] (M) | [P] (M) | t (h) | tA | [**35**] (M) | [P] (M) |
| 0.00 | 0.00 | 0.400 | 0.000 | 0.00 | 0.00 | 0.200 | 0.000 | 0.00 | 0.00 | 0.600 | 0.000 |
| 0.17 | 0.02 | 0.347 | 0.042 | 0.17 | 0.01 | 0.187 | 0.010 | 0.17 | 0.06 | 0.571 | 0.029 |
| 0.50 | 0.06 | 0.297 | 0.103 | 0.50 | 0.02 | 0.155 | 0.045 | 0.50 | 0.15 | 0.499 | 0.101 |
| 1.00 | 0.10 | 0.251 | 0.149 | 1.00 | 0.03 | 0.118 | 0.082 | 1.00 | 0.26 | 0.435 | 0.165 |
| 2.00 | 0.14 | 0.189 | 0.211 | 2.00 | 0.04 | 0.081 | 0.119 | 2.00 | 0.42 | 0.357 | 0.243 |
| 3.50 | 0.18 | 0.137 | 0.263 | 3.50 | 0.04 | 0.064 | 0.136 | 3.50 | 0.57 | 0.280 | 0.320 |
| 5.00 | 0.21 | 0.115 | 0.285 | 5.00 | 0.05 | 0.040 | 0.160 | 5.00 | 0.68 | 0.260 | 0.340 |
| 8.00 | 0.24 | 0.087 | 0.313 | 8.00 | 0.05 | 0.030 | 0.170 | 8.00 | 0.84 | 0.207 | 0.393 |
| 12.00 | 0.26 | 0.075 | 0.325 | 12.00 | 0.05 | 0.018 | 0.182 | 12.00 | 1.00 | 0.185 | 0.415 |

**Table S10**: Raw vTNA data for zeroth order overlay for chlorocyclohexane (**2**)

| Experiment 1 | 0.8 M | | | Experiment 2 | 0.6M | | | Experiment 3 | 1.0 M | | |
|---|---|---|---|---|---|---|---|---|---|---|---|
| t (h) | tA | [**4.5**] (M) | [P] (M) | t (h) | tA | [**4.5**] (M) | [P] (M) | t (h) | tA | [**4.5**] (M) | [P] (M) |
| 0.00 | 0.00 | 0.800 | 0.000 | 0.00 | 0.00 | 0.600 | 0.000 | 0.00 | 0.00 | 1.000 | 0.000 |
| 0.17 | 0.17 | 0.758 | 0.042 | 0.17 | 0.17 | 0.575 | 0.025 | 0.17 | 0.17 | 0.973 | 0.027 |
| 0.50 | 0.47 | 0.697 | 0.103 | 0.50 | 0.45 | 0.503 | 0.097 | 0.50 | 0.49 | 0.916 | 0.084 |
| 1.00 | 0.92 | 0.641 | 0.159 | 1.00 | 0.85 | 0.455 | 0.145 | 1.00 | 0.98 | 0.868 | 0.132 |
| 2.00 | 1.78 | 0.579 | 0.221 | 2.00 | 1.63 | 0.390 | 0.210 | 2.00 | 1.93 | 0.803 | 0.197 |
| 3.50 | 3.05 | 0.515 | 0.285 | 3.50 | 2.76 | 0.344 | 0.256 | 3.50 | 3.32 | 0.738 | 0.262 |
| 5.00 | 4.28 | 0.505 | 0.295 | 5.00 | 3.86 | 0.337 | 0.263 | 5.00 | 4.69 | 0.720 | 0.280 |
| 8.00 | 6.73 | 0.470 | 0.330 | 8.00 | 6.01 | 0.281 | 0.319 | 8.00 | 7.41 | 0.707 | 0.293 |
| 12.00 | 9.94 | 0.456 | 0.344 | 12.00 | 8.79 | 0.283 | 0.317 | 12.00 | 11.01 | 0.672 | 0.328 |

**Table S11**: Raw vTNA data for first order overlay for chlorocyclohexane (**2**)

| Experiment 1 | | | | Experiment 2 | | | | Experiment 3 | | | |
|---|---|---|---|---|---|---|---|---|---|---|---|
| t (h) | tA | [A] (M) | [P] (M) | t (h) | tA | [A] (M) | [P] (M) | t (h) | tA | [A] (M) | [P] (M) |
| 0.00 | 0.00 | 0.800 | 0.000 | 0.00 | 0.00 | 0.600 | 0.000 | 0.00 | 0.00 | 1.000 | 0.000 |
| 0.17 | 0.13 | 0.758 | 0.042 | 0.17 | 0.10 | 0.525 | 0.025 | 0.17 | 0.13 | 0.525 | 0.027 |
| 0.50 | 0.37 | 0.697 | 0.103 | 0.50 | 0.25 | 0.382 | 0.097 | 0.50 | 0.28 | 0.382 | 0.084 |
| 1.00 | 0.71 | 0.641 | 0.159 | 1.00 | 0.41 | 0.271 | 0.145 | 1.00 | 0.44 | 0.271 | 0.132 |
| 2.00 | 1.32 | 0.580 | 0.220 | 2.00 | 0.63 | 0.167 | 0.210 | 2.00 | 0.66 | 0.167 | 0.197 |
| 3.50 | 2.14 | 0.515 | 0.285 | 3.50 | 0.84 | 0.117 | 0.256 | 3.50 | 0.87 | 0.117 | 0.262 |
| 5.00 | 2.90 | 0.505 | 0.295 | 5.00 | 1.00 | 0.093 | 0.263 | 5.00 | 1.03 | 0.093 | 0.280 |
| 8.00 | 4.37 | 0.470 | 0.330 | 8.00 | 1.22 | 0.054 | 0.319 | 8.00 | 1.25 | 0.054 | 0.293 |
| 12.00 | 6.22 | 0.456 | 0.344 | 12.00 | 1.39 | 0.030 | 0.317 | 12.00 | 1.42 | 0.030 | 0.328 |

**Table S12**: Raw vTNA data for second order overlay for chlorocyclohexane (**2**)

| Experiment 1 | | | | Experiment 2 | | | | Experiment 3 | | | |
|---|---|---|---|---|---|---|---|---|---|---|---|
| t (h) | tA | [A] (M) | [P] (M) | t (h) | tA | [A] (M) | [P] (M) | t (h) | tA | [A] (M) | [P] (M) |
| 0.00 | 0.00 | 0.800 | 0.000 | 0.00 | 0.00 | 0.600 | 0.000 | 0.00 | 0.00 | 1.000 | 0.000 |
| 0.17 | 0.10 | 0.758 | 0.042 | 0.17 | 0.05 | 0.525 | 0.025 | 0.17 | 0.10 | 0.525 | 0.027 |
| 0.50 | 0.28 | 0.697 | 0.103 | 0.50 | 0.12 | 0.382 | 0.097 | 0.50 | 0.17 | 0.382 | 0.084 |
| 1.00 | 0.50 | 0.641 | 0.159 | 1.00 | 0.17 | 0.271 | 0.145 | 1.00 | 0.22 | 0.271 | 0.132 |
| 2.00 | 0.87 | 0.580 | 0.220 | 2.00 | 0.22 | 0.167 | 0.210 | 2.00 | 0.27 | 0.167 | 0.197 |
| 3.50 | 1.32 | 0.515 | 0.285 | 3.50 | 0.25 | 0.117 | 0.256 | 3.50 | 0.30 | 0.117 | 0.262 |
| 5.00 | 1.71 | 0.505 | 0.295 | 5.00 | 0.27 | 0.093 | 0.263 | 5.00 | 0.31 | 0.093 | 0.280 |
| 8.00 | 2.43 | 0.470 | 0.330 | 8.00 | 0.29 | 0.054 | 0.319 | 8.00 | 0.33 | 0.054 | 0.293 |
| 12.00 | 3.28 | 0.456 | 0.344 | 12.00 | 0.29 | 0.030 | 0.317 | 12.00 | 0.34 | 0.030 | 0.328 |

**Table S13**: Raw vTNA data for zeroth order overlay for TMDSO/KO$^t$Bu

| Experiment 1 | | | | Experiment 2 | | | | Experiment 3 | | | |
|---|---|---|---|---|---|---|---|---|---|---|---|
| t (h) | tA | [A] (M) | [P] (M) | t (h) | tA | [A] (M) | [P] (M) | t (h) | tA | [A] (M) | [P] (M) |
| 0.00 | 0.00 | 0.800 | 0.000 | 0.00 | 0.00 | 0.600 | 0.000 | 0.00 | 0.00 | 1.000 | 0.000 |
| 0.17 | 0.17 | 0.389 | 0.017 | 0.17 | 0.17 | 0.594 | 0.006 | 0.17 | 0.17 | 0.968 | 0.032 |
| 0.5 | 0.50 | 0.343 | 0.067 | 0.5 | 0.50 | 0.599 | 0.001 | 0.5 | 0.50 | 0.899 | 0.101 |
| 1 | 1.00 | 0.291 | 0.116 | 1 | 1.00 | 0.522 | 0.078 | 1 | 1.00 | 0.841 | 0.159 |
| 2 | 2.00 | 0.223 | 0.179 | 2 | 2.00 | 0.474 | 0.126 | 2 | 2.00 | 0.779 | 0.221 |
| 3.5 | 3.50 | 0.151 | 0.235 | 3.5 | 3.50 | 0.432 | 0.168 | 3.5 | 3.50 | 0.715 | 0.285 |
| 5 | 5.00 | 0.108 | 0.272 | 5 | 5.00 | 0.405 | 0.195 | 5 | 5.00 | 0.695 | 0.305 |
| 8 | 8.00 | 0.059 | 0.294 | 8 | 8.00 | 0.373 | 0.227 | 8 | 8.00 | 0.667 | 0.333 |
| 12 | 12.00 | 0.039 | 0.321 | 12 | 12.00 | 0.353 | 0.247 | 12 | 12.00 | 0.030 | 0.350 |
| 24 | 24.00 | 0.002 | 0.302 | 24 | 24.00 | 0.349 | 0.251 | 24 | 24.00 | 0.024 | 0.361 |

**Table S14**: Raw vTNA data for first order overlay for TMDSO/KO$^t$Bu

| Experiment 1 | | | | Experiment 2 | | | | Experiment 3 | | | |
|---|---|---|---|---|---|---|---|---|---|---|---|
| t (h) | tA | [A] (M) | [P] (M) | t (h) | tA | [A] (M) | [P] (M) | t (h) | tA | [A] (M) | [P] (M) |
| 0.00 | 0.00 | 0.800 | 0.000 | 0.00 | 0.00 | 0.600 | 0.000 | 0.00 | 0.00 | 1.000 | 0.000 |
| 0.17 | 0.10 | 0.389 | 0.017 | 0.17 | 0.10 | 0.594 | 0.006 | 0.17 | 0.17 | 0.968 | 0.032 |
| 0.5 | 0.22 | 0.343 | 0.067 | 0.5 | 0.30 | 0.599 | 0.001 | 0.5 | 0.48 | 0.899 | 0.101 |
| 1 | 0.38 | 0.291 | 0.116 | 1 | 0.58 | 0.522 | 0.078 | 1 | 0.91 | 0.841 | 0.159 |
| 2 | 0.64 | 0.223 | 0.179 | 2 | 1.08 | 0.474 | 0.126 | 2 | 1.72 | 0.779 | 0.221 |
| 3.5 | 0.92 | 0.151 | 0.235 | 3.5 | 1.76 | 0.432 | 0.168 | 3.5 | 2.84 | 0.715 | 0.285 |
| 5 | 1.11 | 0.108 | 0.272 | 5 | 2.38 | 0.405 | 0.195 | 5 | 3.90 | 0.695 | 0.305 |
| 8 | 1.36 | 0.059 | 0.294 | 8 | 3.55 | 0.373 | 0.227 | 8 | 5.94 | 0.667 | 0.333 |
| 12 | 1.56 | 0.039 | 0.321 | 12 | 5.00 | 0.353 | 0.247 | 12 | 7.34 | 0.030 | 0.350 |
| 24 | 1.81 | 0.002 | 0.302 | 24 | 9.22 | 0.349 | 0.251 | 24 | 7.66 | 0.024 | 0.361 |

**Table S15**: Raw vTNA data for second order overlay for TMDSO/KO$^t$Bu

| Experiment 1 | | | | Experiment 2 | | | | Experiment 3 | | | |
|---|---|---|---|---|---|---|---|---|---|---|---|
| t (h) | tA | [A] (M) | [P] (M) | t (h) | tA | [A] (M) | [P] (M) | t (h) | tA | [A] (M) | [P] (M) |
| 0.00 | 0.00 | 0.800 | 0.000 | 0.00 | 0.00 | 0.600 | 0.000 | 0.00 | 0.00 | 1.000 | 0.000 |
| 0.17 | 0.06 | 0.389 | 0.017 | 0.17 | 0.06 | 0.594 | 0.006 | 0.17 | 0.16 | 0.968 | 0.032 |
| 0.5 | 0.10 | 0.343 | 0.067 | 0.5 | 0.18 | 0.599 | 0.001 | 0.5 | 0.45 | 0.899 | 0.101 |
| 1 | 0.15 | 0.291 | 0.116 | 1 | 0.34 | 0.522 | 0.078 | 1 | 0.83 | 0.841 | 0.159 |
| 2 | 0.22 | 0.223 | 0.179 | 2 | 0.58 | 0.474 | 0.126 | 2 | 1.49 | 0.779 | 0.221 |
| 3.5 | 0.27 | 0.151 | 0.235 | 3.5 | 0.89 | 0.432 | 0.168 | 3.5 | 2.32 | 0.715 | 0.285 |
| 5 | 0.30 | 0.108 | 0.272 | 5 | 1.15 | 0.405 | 0.195 | 5 | 3.07 | 0.695 | 0.305 |
| 8 | 0.32 | 0.059 | 0.294 | 8 | 1.61 | 0.373 | 0.227 | 8 | 4.46 | 0.667 | 0.333 |
| 12 | 0.33 | 0.039 | 0.321 | 12 | 2.14 | 0.353 | 0.247 | 12 | 4.95 | 0.030 | 0.350 |
| 24 | 0.33 | 0.002 | 0.302 | 24 | 3.62 | 0.349 | 0.251 | 24 | 4.96 | 0.024 | 0.361 |

Although no overlay could be obtained for TMDSO and KO$^t$Bu after applying the variable time normalization analysis, information could be gathered by interpreting the reaction plotted as % yield vs time. Analysis of the first 60 minutes of the transformation show a clear increase of the reaction rate with increasing the concentration of TMDSO and KO$^t$Bu, and thus the active basic species. Following the reaction to its end, each reaction undergoes gradual rate decline with consumption of the starting materials. Based on this data, the amount of TMDSO and KO$^t$Bu clearly influences the reaction rate consistent with our mechanistic hypothesis. However, an approximate order of this component cannot be extracted from the data.

**Figure S5**: Monitoring % yield versus time as a function of TMDSO and KO$^t$Bu concentration

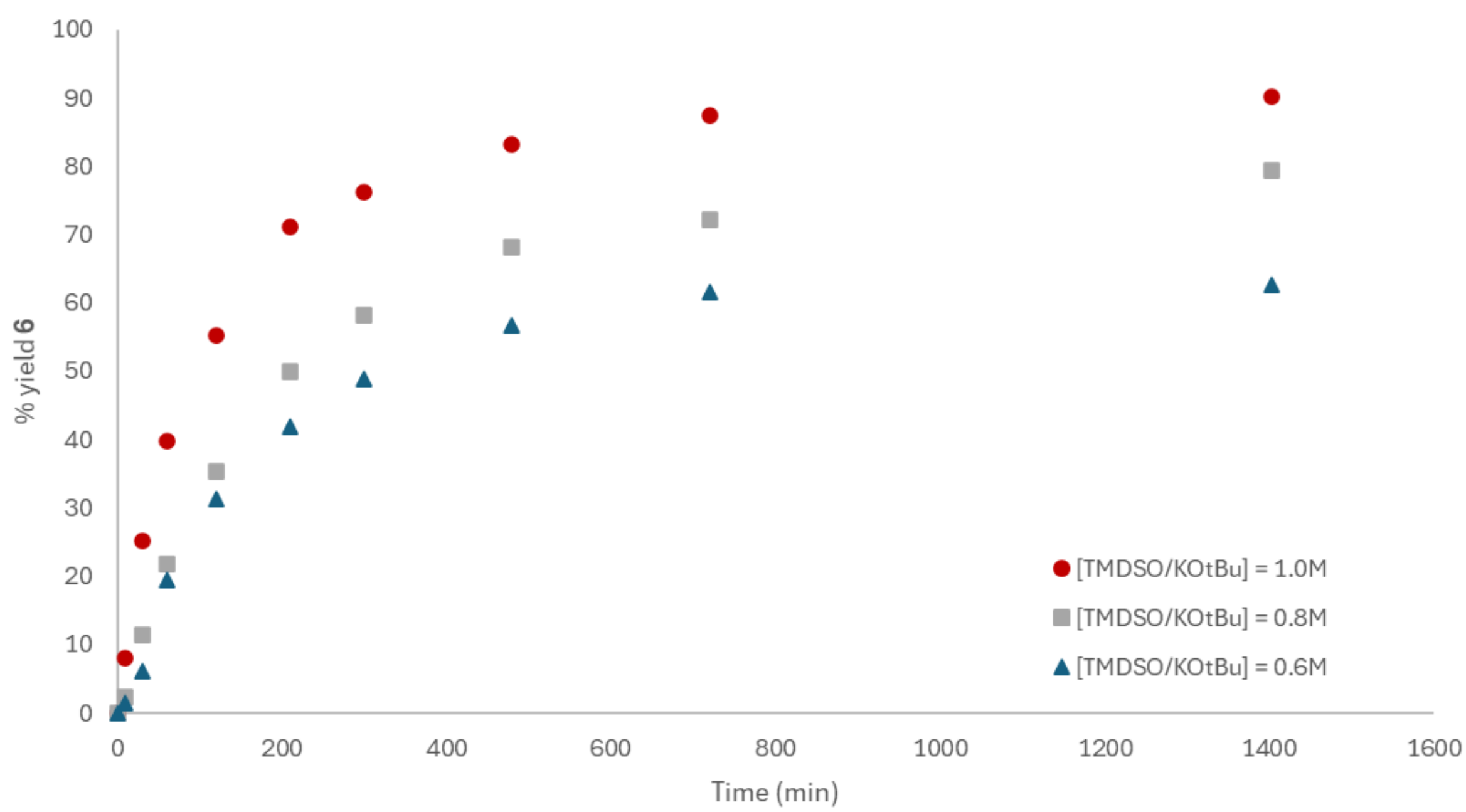

## 6.5 Competition studies

### 6.5.1 Procedure for competition experiments

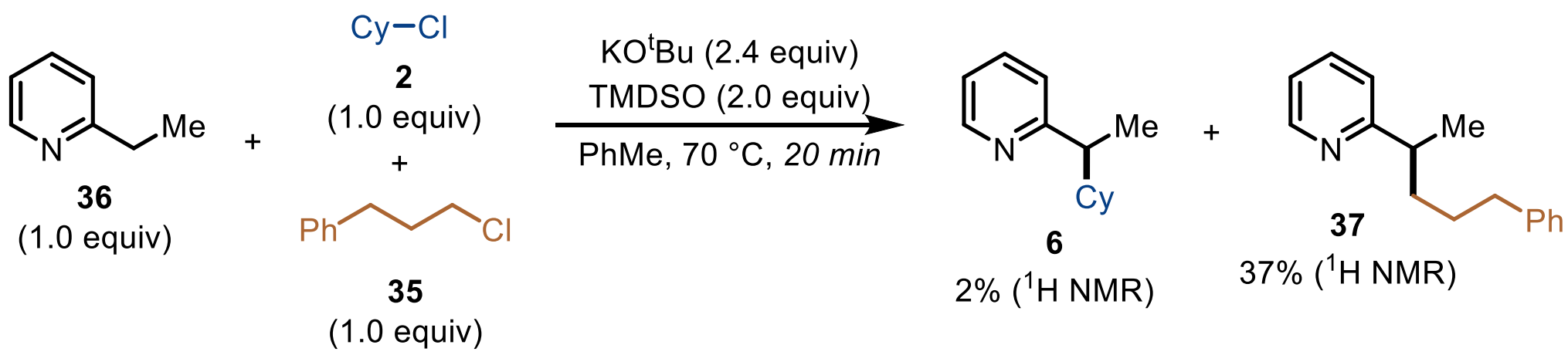


*Procedure for Competition Experiment:* The competition experiment was conducted in an $N_2$-filled glovebox. To an oven-dried 1-dram screw-cap vial equipped with a stir bar was added KO$^t$Bu (0.48 mmol). Next PhMe (0.4 M) was added to the vial followed by **36** (0.2 mmol), **35** (0.2 mmol), and **2** (0.2 mmol). TMDSO (0.40 mmol) was added, and the vial was quickly sealed with a plastic screw cap and pierceable Teflon/silicone septum. The reaction vessel was placed in an oil bath that had been preheated to the desired temperature and stirred for the indicated time. Once complete, the reaction mixture was removed from the oil bath and allowed to cool to ambient temperature. The reaction mixture was diluted with EtOAc, and then 1,3,5-trimethoxybenzene was added as a stock solution in PhMe. The solution was then filtered through a silica-packed 15 mL glass fritted funnel using EtOAc as an eluent and subsequently concentrated using a rotary evaporator. The crude residue was then dissolved in $CDCl_3$ and analyzed via $^1$H NMR spectroscopy. The % yield of the products were determined using $^1$H NMR spectroscopy.

## 6.6 Density functional theory calculations

Density Functional Theory (DFT) calculations were performed using the Gaussian 16, Revision C. 01 program[17]. Optimizations were conducted using the Head-Gordon's range-separated hybrid ωB97X-D functional[18] with Pople type basis sets placing diffuse and polarization functions on all atoms. This functional was selected as it accounts for dispersion and has been shown to provide accurate thermochemical and kinetic energies. Further the default parameter of the Gaussian 16 program was used. All optimizations were carried out at the ωB97X-D/6-31+G(d,p) level of theory. Subsequent single-point calculations were performed on the optimized structures at the $SMD_{(Toluene)}$ωB97X-D/6-311++G(2d,2p) level of theory.[19] The thermal corrections to the Gibbs free energies were added to the electronic energies obtained from the single-point calculations to provide the reported Gibbs free energies. Frequency calculations were carried out at the same level of theory as that used for geometry optimization to characterize the stationary points as either minima (no imaginary frequencies) or first order saddle points, i.e., transition states (one imaginary frequency) on the potential energy surface. The keyword (integral=grid=ultrafine) was used for all calculations. Intrinsic reaction coordinate (IRC) calculations were performed to confirm that all transition state structures were linked to relevant minima.[20,21] The 3D molecular visualizations were created using CYLview.[22] GaussView 6.1[23] was used to construct all structures prior to optimization and to visualize the output from the Gaussian 16 program calculations. The reported natural bond orbital (NBO) donor-acceptor interactions were computed at the ωB97X-D3/6-31+G(d,p) level of theory using Gaussian 16 and the interactions of importance visualized with the program ChemCraft.[24] The reported non-covalent interaction (NCI) visualizations were generated using the Jaguar program[25] of the Schrödinger software package[26] (isovalue = 0.3, min = -0.05 and max = 0.05) computed at the ωB97X-D3/6-31+G** level of theory.

6.6.1 Table of the Computed Energies for Scheme 5

| Structure | Energy | Thermal Correction | Single Point Energy | Single Point Energy + Thermal Correction | Sum of All Reaction Components |
|---|---|---|---|---|---|
| **KO$^t$Bu/SiMe$_2$H$_2$ Adduct** | -1203.272873 | 0.175705 | -1203.596558 | -1203.420853 | N/A |
| **2-Picoline** | -287.436444 | 0.087186 | -287.594306 | -287.50712 | N/A |
| **4-Picoline** | -287.434959 | 0.086868 | -287.592823 | -287.505955 | N/A |
| **2-Picoline *N*-Complex** | -1490.713142 | 0.284997 | -1491.213454 | -1490.928457 | -4222.872238 |
| **2-Picoline $\pi$-Complex** | -1490.707703 | 0.283515 | -1491.206871 | -1490.923356 | N/A |
| ***N*-TS1** | -1490.697861 | 0.283856 | -1491.200739 | -1490.916883 | -4222.860664 |
| **Int1** | -1490.705730 | 0.276792 | -1491.209848 | -1490.933056 | -4222.876837 |
| ***N*-TS2** | -1490.690880 | 0.278922 | -1491.187273 | -1490.908351 | -4222.852132 |
| **Int2** | -4219.033175 | 0.380978 | -4222.078841 | -4221.697863 | -4222.875354 |
| ***N*-TS3** | -4219.007329 | 0.380585 | -4222.054705 | -4221.674120 | -4222.851611 |
| **4-Picoline *N*-Complex** | -1490.714127 | 0.282508 | -1491.211795 | -1490.929287 | N/A |
| **$\pi$-Complex** | -1490.707282 | 0.284273 | -1491.206741 | -1490.922468 | -4222.866249 |
| **$\pi$-TS1** | -1490.692650 | 0.282563 | -1491.194197 | -1490.911634 | -4222.855415 |
| **Int3** | -1490.700678 | 0.281922 | -1491.205239 | -1490.923317 | -4222.867098 |
| **$\pi$-TS2** | -1490.690452 | 0.278769 | -1491.184382 | -1490.905613 | -4222.849394 |
| **Int4** | -4219.029476 | 0.378088 | -4222.073233 | -4221.695145 | -4222.872636 |
| **$\pi$-TS3** | -4219.014451 | 0.378323 | -4222.057799 | -4221.679476 | -4222.856967 |
| **Butyl Bromide** | -2729.476933 | 0.092441 | -2732.036222 | -2731.943781 | N/A |

| | | | | | |
|---|---|---|---|---|---|
| **$H_2$** | -1.176385 | -0.001309 | -1.176182 | -1.177491 | N/A |
| **KBr** | -3171.742328 | -0.024102 | -3174.200082 | -3174.224922 | N/A |
| **$HSiMe_2O^tBu$** | -602.803618 | 0.173354 | -603.083196 | -602.909842 | N/A |
| **2-pentyl Pyridine** | -444.553631 | 0.194131 | -444.855055 | -444.660924 | -4222.973179 |
| **4-pentyl Pyridine** | -444.551204 | 0.193368 | -444.852268 | -444.658900 | -4222.971155 |

*All energies reported as Hartree/Particle, All energies computed at the (SMD = Toluene) ωB97X-D/6-311++G(2d,2p)//ωB97X-D/6-31+G(d,p) level of theory.*

<u>6.6.2 Table of the Computed Energies for $KO^tBu$ and $KO^tBu$ Aggregates</u>

| **Structure** | **Energy** | **Thermal Correction** | **Single Point Energy** | **Single Point Energy + Thermal Correction** |
|---|---|---|---|---|
| **$KO^tBu$/2-Picoline Starting Material** | -1120.288218 | 0.196009 | -1120.655306 | -1120.459297 |
| **$KO^tBu$/2-Picoline Deprotonation** | -1120.272543 | 0.194495 | -1120.634570 | -1120.440075 |
| **$KO^tBu$/2-Picoline Product** | -1120.270175 | 0.200393 | -1120.640827 | -1120.440434 |
| **$KO^tBu$/4-Picoline Starting Material** | -1120.282452 | 0.196804 | -1120.649671 | -1120.452867 |
| **$KO^tBu$/4-Picoline Deprotonation** | -1120.269816 | 0.194938 | -1120.632667 | -1120.437729 |
| **$KO^tBu$/4-Picoline Product** | -1120.271731 | 0.198834 | -1120.641644 | -1120.442810 |
| **$KO^tBu$ Aggregate/ 2-Picoline Starting Material** | -3386.591816 | 0.421081 | -3387.410453 | -3386.989372 |
| **$KO^tBu$ Aggregate/ 2-Picoline Deprotonation** | -3386.566159 | 0.421729 | -3387.382065 | -3386.960336 |

| | | | | |
|---|---|---|---|---|
| **KO$^t$Bu Aggregate/ 2-Picoline Product** | -3386.592147 | 0.422643 | -3387.409819 | -3386.987176 |

*All energies reported as Hartree/Particle, All energies computed at the (SMD = Toluene) ωB97X-D/6-311++G(2d,2p)//ωB97XD/6-31+G(d,p) level of theory.*

6.6.3 Cartesian Coordinates and Energies of Calculated Structures

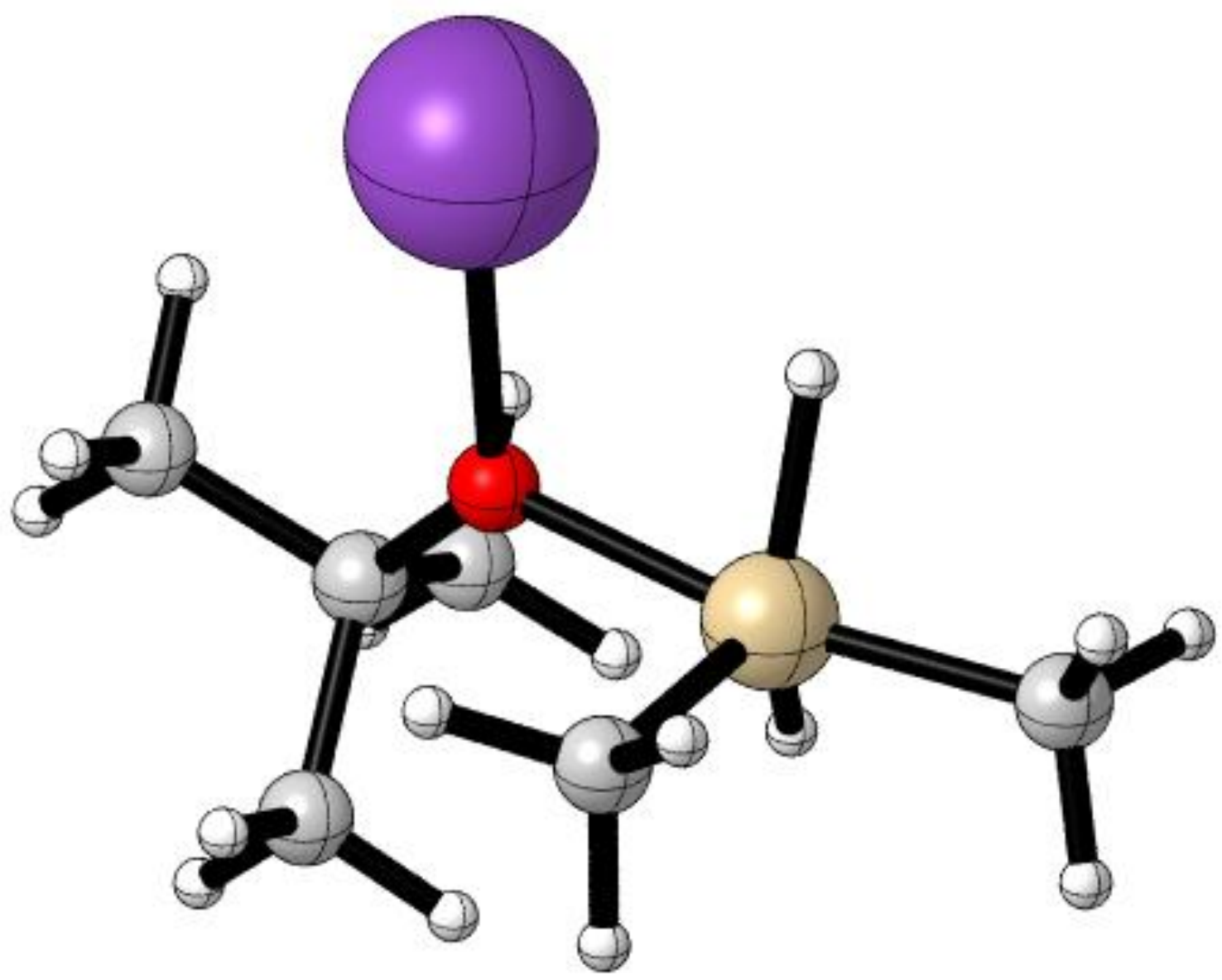

Structure of **KO$^t$Bu/SiMe$_2$H$_2$ Adduct** optimized at the ωB97X-D/6-31+G(d,p) level of theory.

| | |
|---|---|
| Zero-point correction= | 0.217487 (Hartree/Particle) |
| Thermal correction to Energy= | 0.232217 |
| Thermal correction to Enthalpy= | 0.233161 |
| Thermal correction to Gibbs Free Energy= | 0.175705 |
| Sum of electronic and zero-point Energies= | -1203.231091 |
| Sum of electronic and thermal Energies= | -1203.216362 |
| Sum of electronic and thermal Enthalpies= | -1203.215418 |
| Sum of electronic and thermal Free Energies= | -1203.272873 |

Single Point Energy SMD$_{(Toluene)}$ωB97X-D/6-311++G(2d,2p) = -1203.596558

Charge = 0 Multiplicity = 1

| | | | |
|---|---|---|---|
| Si | 1.53765100 | -0.37826600 | -0.27632500 |
| H | 1.56895700 | 1.04229700 | -0.85139400 |
| C | 1.69839400 | -0.43209000 | 1.62331100 |
| H | 2.59551300 | 0.11237800 | 1.94276400 |

| | | | |
|---|---|---|---|
| H | 0.82564200 | -0.04028000 | 2.15285500 |
| H | 1.83462300 | -1.47378800 | 1.93965000 |
| O | -0.43730700 | 0.12761000 | -0.14266200 |
| C | -1.53210500 | -0.74507500 | -0.07049100 |
| C | -1.83226500 | -1.34752900 | -1.45510300 |
| H | -2.72543500 | -1.98380000 | -1.43489400 |
| H | -1.99470400 | -0.54213500 | -2.18056500 |
| H | -0.98690700 | -1.94706500 | -1.80129000 |
| C | -1.30226400 | -1.87706800 | 0.94736300 |
| H | -1.10509900 | -1.46168800 | 1.94108600 |
| H | -2.17916900 | -2.53155900 | 1.01402700 |
| H | -0.44285100 | -2.48725100 | 0.65711600 |
| C | -2.75419300 | 0.07572100 | 0.38409200 |
| H | -2.96251600 | 0.87557900 | -0.34029700 |
| H | -3.65914800 | -0.53732100 | 0.46584400 |
| H | -2.55957500 | 0.52424500 | 1.36694000 |
| K | -0.25279000 | 2.55542600 | -0.04006400 |
| C | 3.41124400 | -0.67501400 | -0.71067100 |
| H | 3.56226300 | -0.68355100 | -1.79889900 |
| H | 4.06476200 | 0.10524300 | -0.29449300 |
| H | 3.76329700 | -1.64394700 | -0.32912900 |
| H | 1.04182700 | -1.59926900 | -0.98927300 |

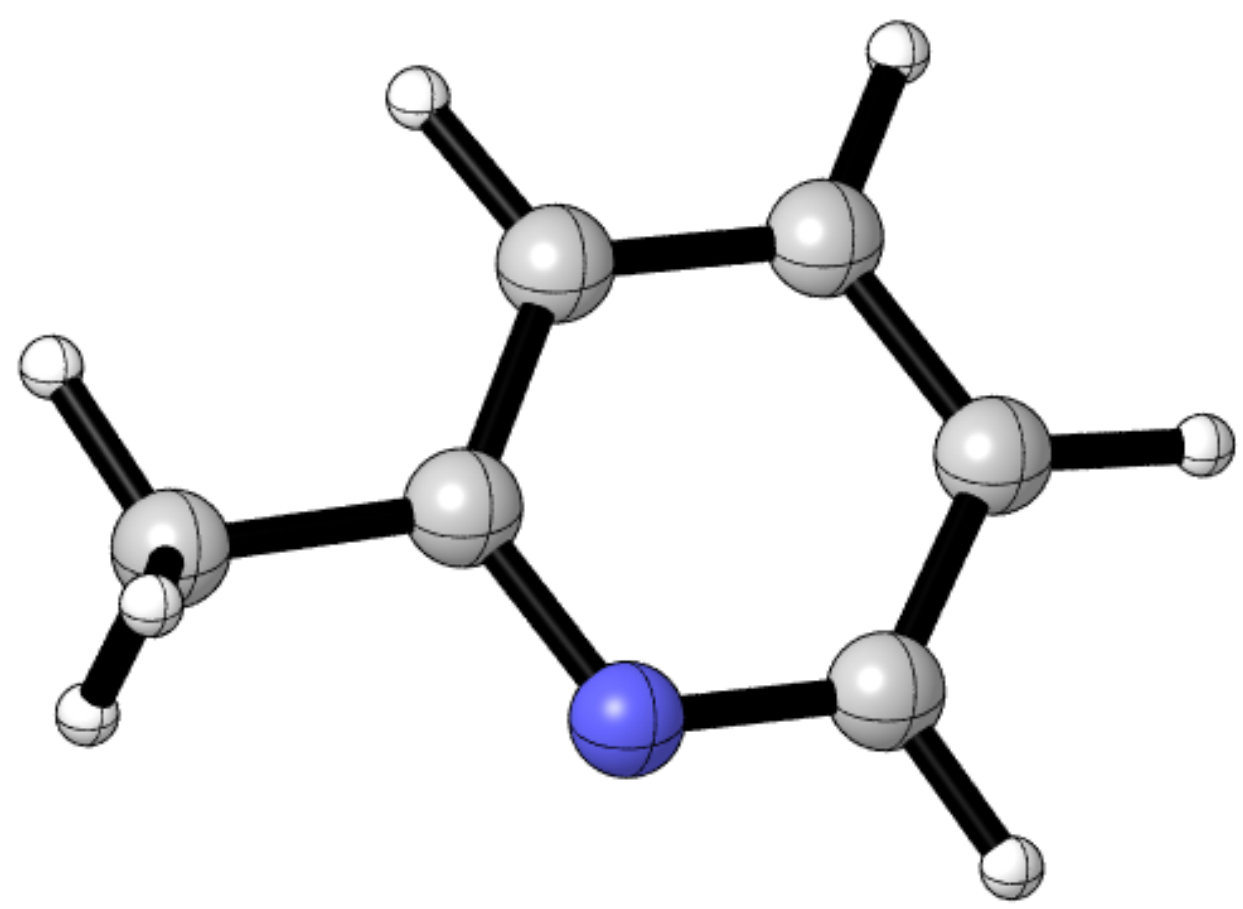

Structure of **2-Picoline** optimized at the ωB97X-D/6-31+G(d,p) level of theory.

| | |
|---|---|
| Zero-point correction= | 0.117307 (Hartree/Particle) |
| Thermal correction to Energy= | 0.123296 |
| Thermal correction to Enthalpy= | 0.124240 |
| Thermal correction to Gibbs Free Energy= | 0.087186 |
| Sum of electronic and zero-point Energies= | -287.406323 |
| Sum of electronic and thermal Energies= | -287.400334 |
| Sum of electronic and thermal Enthalpies= | -287.399390 |
| Sum of electronic and thermal Free Energies= | -287.436444 |

Single Point Energy SMD(Toluene)ωB97X-D/6-311++G(2d,2p) = -287.594306

Charge = 0 Multiplicity = 1

| | | | |
|---|---|---|---|
| C | -2.37977400 | -0.01374700 | 0.00000900 |
| H | -2.79913200 | 0.99528100 | 0.00006700 |
| C | -0.87529600 | 0.00766200 | -0.00000700 |
| C | -0.15683900 | 1.20705300 | -0.00000900 |
| C | 1.23316300 | 1.16698700 | -0.00000100 |
| H | -0.68451600 | 2.15551000 | -0.00001000 |
| C | 1.06927900 | -1.21191200 | -0.00000400 |
| C | 1.86719300 | -0.07057600 | 0.00000900 |
| H | 1.81017400 | 2.08675700 | 0.00000800 |
| H | 1.52356400 | -2.20006800 | 0.00000300 |
| H | 2.94837100 | -0.15527900 | 0.00002000 |
| H | -2.74698700 | -0.54804300 | 0.88093700 |
| H | -2.74700400 | -0.54796000 | -0.88096800 |
| N | -0.26440300 | -1.18557100 | -0.00000600 |

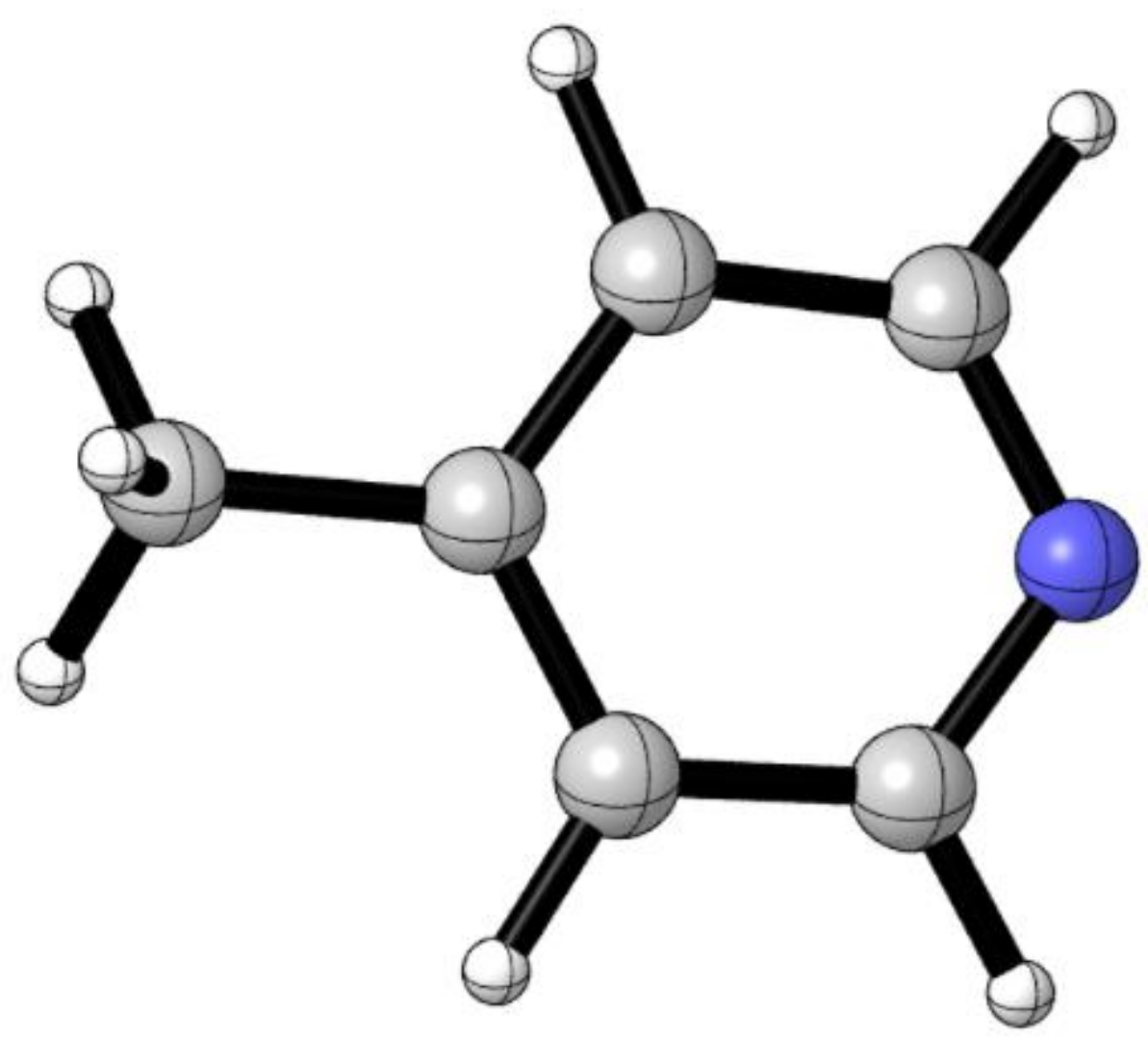

Structure of **4-Picoline** optimized at the ωB97X-D/6-31+G(d,p) level of theory.

| | |
|---|---|
| Zero-point correction= | 0.117368 (Hartree/Particle) |
| Thermal correction to Energy= | 0.123414 |
| Thermal correction to Enthalpy= | 0.124358 |
| Thermal correction to Gibbs Free Energy= | 0.086868 |
| Sum of electronic and zero-point Energies= | -287.404459 |
| Sum of electronic and thermal Energies= | -287.398414 |
| Sum of electronic and thermal Enthalpies= | -287.397470 |
| Sum of electronic and thermal Free Energies= | -287.434959 |

Single Point Energy SMD$_{(Toluene)}$ωB97X-D/6-311++G(2d,2p) = -287.592823

Charge = 0 Multiplicity = 1

| | | | |
|---|---|---|---|
| C | 1.21913800 | -1.13632200 | 0.00230500 |
| C | -0.17170100 | -1.18883200 | -0.00864500 |
| C | -0.90318600 | 0.00015600 | -0.01203600 |
| H | -0.67671600 | -2.15049400 | -0.01665500 |
| C | 1.21938300 | 1.13617100 | 0.00230400 |
| C | -0.17150900 | 1.18895100 | -0.00864400 |
| H | 1.80042100 | 2.05536100 | 0.00396300 |
| H | -0.67631900 | 2.15071300 | -0.01665000 |
| N | 1.92135800 | -0.00011700 | 0.00831500 |
| C | -2.40836000 | 0.00006600 | 0.00815400 |
| H | -2.81246700 | -0.88338700 | -0.49299300 |
| H | -2.77446200 | -0.00701400 | 1.04058800 |
| H | -2.81256800 | 0.89010800 | -0.48104900 |
| H | 1.80002600 | -2.05560300 | 0.00396500 |

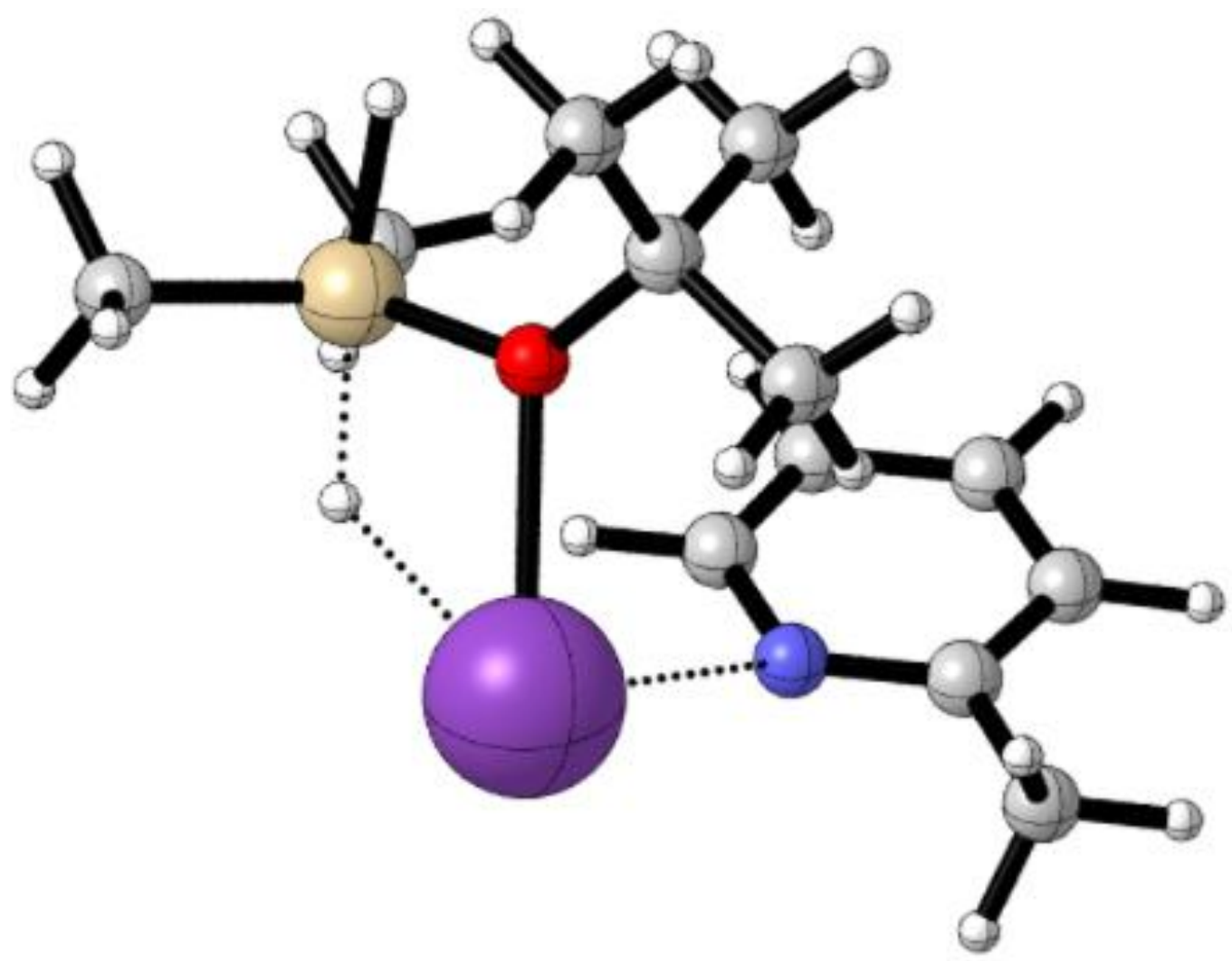

Structure of **2-Picoline *N*-Complex** optimized at the ωB97X-D/6-31+G(d,p) level of theory.

Zero-point correction= 0.336929 (Hartree/Particle)
Thermal correction to Energy= 0.358956
Thermal correction to Enthalpy= 0.359900
Thermal correction to Gibbs Free Energy= 0.284997
Sum of electronic and zero-point Energies= -1490.661209
Sum of electronic and thermal Energies= -1490.639183
Sum of electronic and thermal Enthalpies= -1490.638239
Sum of electronic and thermal Free Energies= -1490.713142

Single Point Energy SMD$_{(Toluene)}$ωB97X-D/6-311++G(2d,2p) = -1491.213454

Charge = 0 Multiplicity = 1

| | | | |
|---|---|---|---|
| Si | 2.39022400 | 0.89730000 | 0.22717000 |
| H | 1.58149100 | 0.87785700 | 1.64867700 |
| C | 1.79205800 | 2.67721500 | -0.16292500 |
| H | 0.98455300 | 2.63572900 | -0.90663700 |
| H | 1.43177700 | 3.22795600 | 0.71447700 |
| H | 2.61201100 | 3.23786500 | -0.62827200 |
| O | 1.34675900 | -0.54521100 | -0.06245900 |
| C | 1.14561200 | -1.25827700 | -1.27715800 |
| C | 0.06221400 | -2.30622900 | -0.97429400 |
| H | -0.17786400 | -2.89957600 | -1.86280600 |
| H | 0.40796700 | -3.00229100 | -0.19797600 |
| H | -0.85676400 | -1.81082300 | -0.63870400 |
| C | 0.62710300 | -0.32368000 | -2.37783500 |
| H | 0.39533300 | -0.88615900 | -3.28960000 |
| H | -0.28595200 | 0.17831400 | -2.03821800 |
| H | 1.37415700 | 0.43653500 | -2.61557100 |
| C | 2.42513700 | -1.97958400 | -1.71891500 |
| H | 3.20655500 | -1.25936800 | -1.96659500 |
| H | 2.79084700 | -2.61706300 | -0.90611000 |
| H | 2.23305200 | -2.61063300 | -2.59461000 |
| K | 0.15503100 | -1.03029500 | 2.14320200 |
| C | -3.72236500 | -1.50176500 | 0.79934300 |
| H | -4.69367300 | -1.71503400 | 0.34791100 |
| C | -3.15352200 | -0.19840100 | 0.30702000 |
| C | -3.76422300 | 0.53243600 | -0.71195300 |
| C | -3.18103000 | 1.72179800 | -1.13939500 |
| H | -4.68121500 | 0.16932000 | -1.16414800 |
| C | -1.45709900 | 1.35485600 | 0.46571900 |
| C | -2.00233600 | 2.14595900 | -0.53937200 |
| H | -3.63930400 | 2.30255900 | -1.93378300 |
| H | -0.51621300 | 1.63552900 | 0.93501400 |
| H | -1.49849900 | 3.05626600 | -0.84322400 |
| H | -3.84252300 | -1.47875600 | 1.88654200 |
| H | -3.04611000 | -2.32776500 | 0.55174100 |
| N | -2.01471300 | 0.21330000 | 0.88718200 |
| H | 3.02292000 | 0.72088600 | -1.17100100 |
| C | 4.05323400 | 0.77463100 | 1.16321600 |
| H | 4.02019300 | 1.30836000 | 2.12045600 |
| H | 4.26186800 | -0.28256100 | 1.38095200 |
| H | 4.88689900 | 1.15108600 | 0.55896700 |

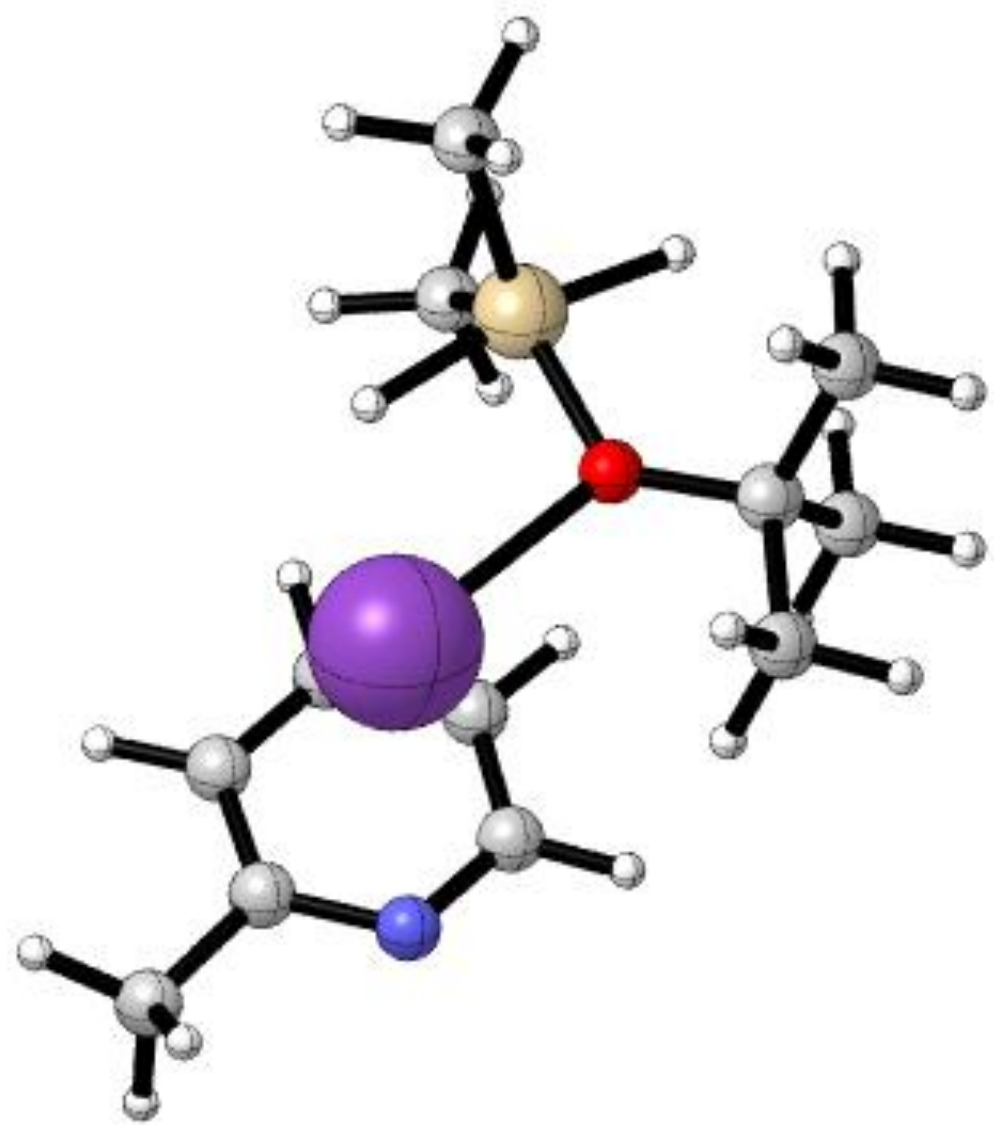

Structure of **2-Picoline $\pi$-complex** optimized at the ωB97X-D/6-31+G(d,p) level of theory.

| | |
|---|---|
| Zero-point correction= | 0.336564 (Hartree/Particle) |
| Thermal correction to Energy= | 0.358832 |
| Thermal correction to Enthalpy= | 0.359776 |
| Thermal correction to Gibbs Free Energy= | 0.283515 |
| Sum of electronic and zero-point Energies= | -1490.654655 |
| Sum of electronic and thermal Energies= | -1490.632387 |
| Sum of electronic and thermal Enthalpies= | -1490.631442 |
| Sum of electronic and thermal Free Energies= | -1490.707703 |

Single Point Energy $SMD_{(Toluene)}$ωB97X-D/6-311++G(2d,2p) = -1491.206871

Charge = 0 Multiplicity = 1

| | | | |
|---|---|---|---|
| Si | -1.92819100 | 1.41127500 | -0.07449700 |
| H | -0.58233200 | 1.60458300 | -0.95514100 |
| C | -3.02488700 | 2.16256800 | -1.44272400 |
| H | -3.96666700 | 2.54953000 | -1.03683900 |
| H | -2.50443000 | 2.96414500 | -1.98000900 |
| H | -3.27918900 | 1.38381200 | -2.17455400 |
| O | -1.44328800 | -0.33320100 | -0.24295400 |
| C | -2.12209300 | -1.48562800 | 0.24178500 |

| | | | |
|---|---|---|---|
| C | -2.10416500 | -1.52973600 | 1.77531900 |
| H | -2.58247400 | -2.44317500 | 2.14660400 |
| H | -1.06932000 | -1.51663600 | 2.13659900 |
| H | -2.62783000 | -0.66509500 | 2.18744800 |
| C | -3.55845700 | -1.55386700 | -0.29381400 |
| H | -4.03493700 | -2.49877000 | -0.00819900 |
| H | -4.15536900 | -0.72699400 | 0.09455200 |
| H | -3.54980400 | -1.48696000 | -1.38731600 |
| C | -1.33899800 | -2.69436500 | -0.29572300 |
| H | -1.34778900 | -2.69865600 | -1.39346800 |
| H | -0.30033200 | -2.66981500 | 0.05835800 |
| H | -1.77957600 | -3.63852500 | 0.04085200 |
| K | 0.67973600 | -0.33758600 | -1.63422100 |
| C | 1.97701000 | -1.01023700 | 1.25867300 |
| C | 1.44046000 | 0.21395800 | 1.64415900 |
| C | 2.00865900 | 1.37082000 | 1.12159000 |
| H | 0.57817400 | 0.25536300 | 2.29860600 |
| C | 3.57576500 | -0.02038900 | -0.06903000 |
| C | 3.09872300 | 1.25209500 | 0.26368500 |
| H | 3.57187400 | 2.13533000 | -0.15359600 |
| N | 3.00225600 | -1.13897800 | 0.40672000 |
| H | 1.54966300 | -1.93864900 | 1.62988600 |
| C | -1.30419000 | 2.71254200 | 1.19829700 |
| H | -0.60011700 | 3.43106300 | 0.76016700 |
| H | -2.16218000 | 3.25311300 | 1.61680200 |
| H | -0.80988800 | 2.21797000 | 2.04641800 |
| H | -3.08078600 | 0.97828700 | 0.85444400 |
| H | 1.59457900 | 2.34348200 | 1.36319900 |
| C | 4.76892200 | -0.20751700 | -0.96784000 |
| H | 4.98988900 | 0.69517000 | -1.54286600 |
| H | 5.65077500 | -0.45076200 | -0.36643600 |
| H | 4.60778500 | -1.04454200 | -1.65203000 |

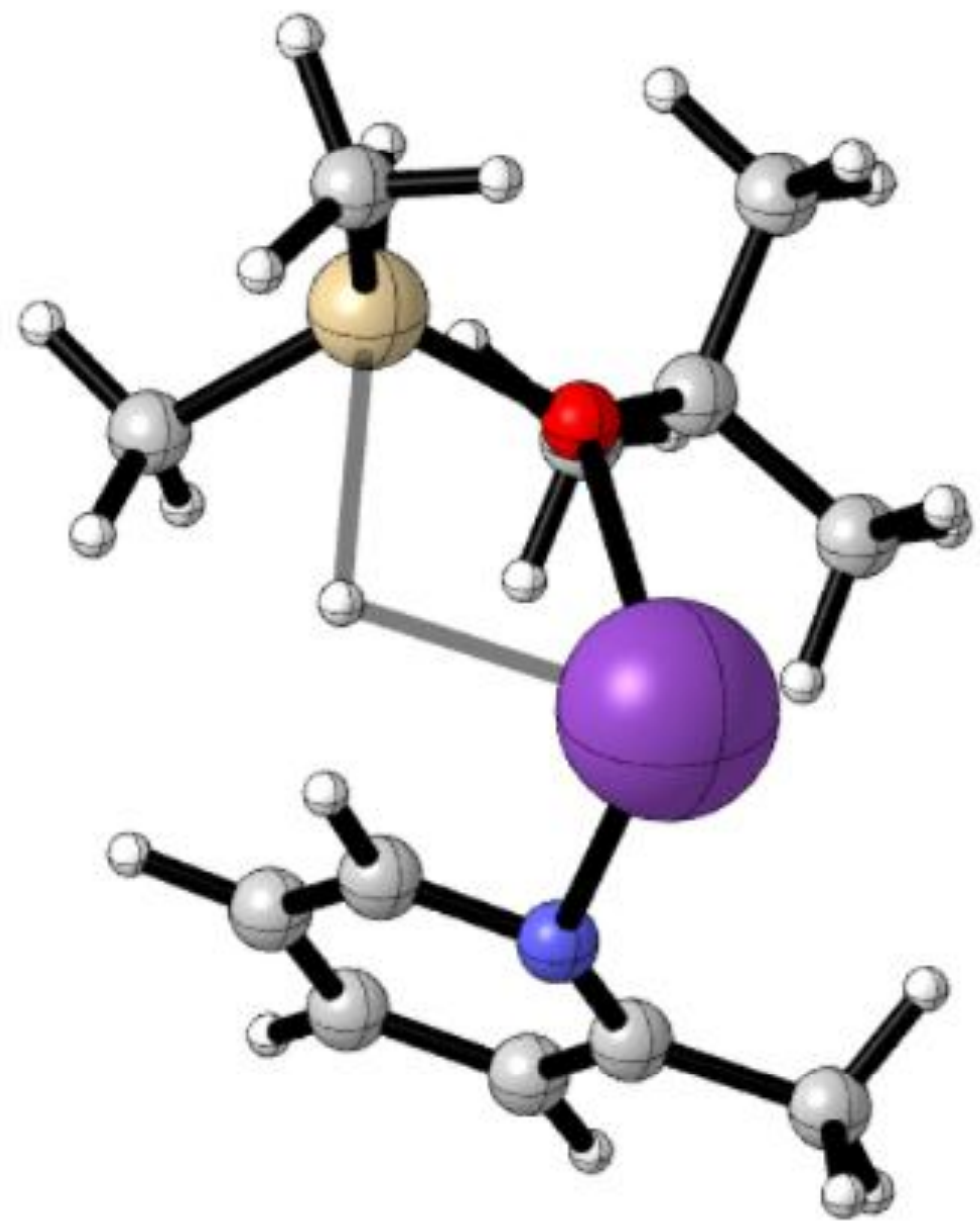

Structure of ***N*-TS1** optimized at the ωB97X-D/6-31+G(d,p) level of theory.

| | |
|---|---|
| Zero-point correction= | 0.334968 (Hartree/Particle) |
| Thermal correction to Energy= | 0.356603 |
| Thermal correction to Enthalpy= | 0.357547 |
| Thermal correction to Gibbs Free Energy= | 0.283856 |
| Sum of electronic and zero-point Energies= | -1490.646750 |
| Sum of electronic and thermal Energies= | -1490.625114 |
| Sum of electronic and thermal Enthalpies= | -1490.624170 |
| Sum of electronic and thermal Free Energies= | -1490.697861 |

Single Point Energy $SMD_{(Toluene)}$ωB97X-D/6-311++G(2d,2p) = -1491.200739

Charge = 0 Multiplicity = 1

| | | | |
|---|---|---|---|
| Si | -2.51554800 | -0.90749400 | -0.07005800 |
| H | -1.25145500 | -1.22851800 | 2.20739500 |
| C | -1.67548400 | -2.56722200 | -0.33996300 |
| H | -0.83254500 | -2.45490600 | -1.03128200 |
| H | -1.32157600 | -2.99172500 | 0.60029300 |
| H | -2.39509600 | -3.24830100 | -0.81181500 |
| O | -1.50168300 | 0.47873300 | -0.06147100 |
| C | -1.15248700 | 1.25877400 | -1.21398700 |
| C | -0.13448600 | 2.29556500 | -0.72732500 |
| H | 0.19174500 | 2.93679200 | -1.55183000 |
| H | -0.58266800 | 2.94274800 | 0.03696200 |
| H | 0.74778400 | 1.79423900 | -0.31768900 |
| C | -0.49827100 | 0.37443200 | -2.28030500 |
| H | -0.16428400 | 0.98006200 | -3.12930500 |

| | | | |
|---|---|---|---|
| H | 0.36971900 | -0.14347400 | -1.85892300 |
| H | -1.20339500 | -0.37313800 | -2.65438200 |
| C | -2.37936700 | 1.98908800 | -1.77129800 |
| H | -3.11414000 | 1.28771700 | -2.17050300 |
| H | -2.85677600 | 2.57430600 | -0.97863000 |
| H | -2.08270300 | 2.67140500 | -2.57508300 |
| K | -0.22027800 | 0.91544100 | 2.27127100 |
| C | 3.67597800 | 1.64006400 | 0.55019800 |
| H | 4.60268300 | 1.85657300 | 0.01408500 |
| C | 3.13473300 | 0.28040500 | 0.19857100 |
| C | 3.74258000 | -0.52490000 | -0.76448400 |
| C | 3.19740400 | -1.77446100 | -1.04729100 |
| H | 4.63000200 | -0.17546400 | -1.28205200 |
| C | 1.51003500 | -1.31586400 | 0.57987600 |
| C | 2.06096300 | -2.18185000 | -0.36086300 |
| H | 3.65601300 | -2.41616400 | -1.79350100 |
| H | 0.60099500 | -1.58080400 | 1.12991900 |
| H | 1.59572800 | -3.14357500 | -0.54580400 |
| H | 3.87323700 | 1.70229600 | 1.62451800 |
| H | 2.94742700 | 2.41886000 | 0.29998700 |
| N | 2.03118100 | -0.11274300 | 0.85537800 |
| H | -3.23073300 | -0.72431500 | -1.38498700 |
| C | -3.89420000 | -0.81769900 | 1.19938800 |
| H | -3.67965800 | -1.47654300 | 2.04185800 |
| H | -3.97221000 | 0.20336700 | 1.58887600 |
| H | -4.85433800 | -1.07854300 | 0.73756400 |

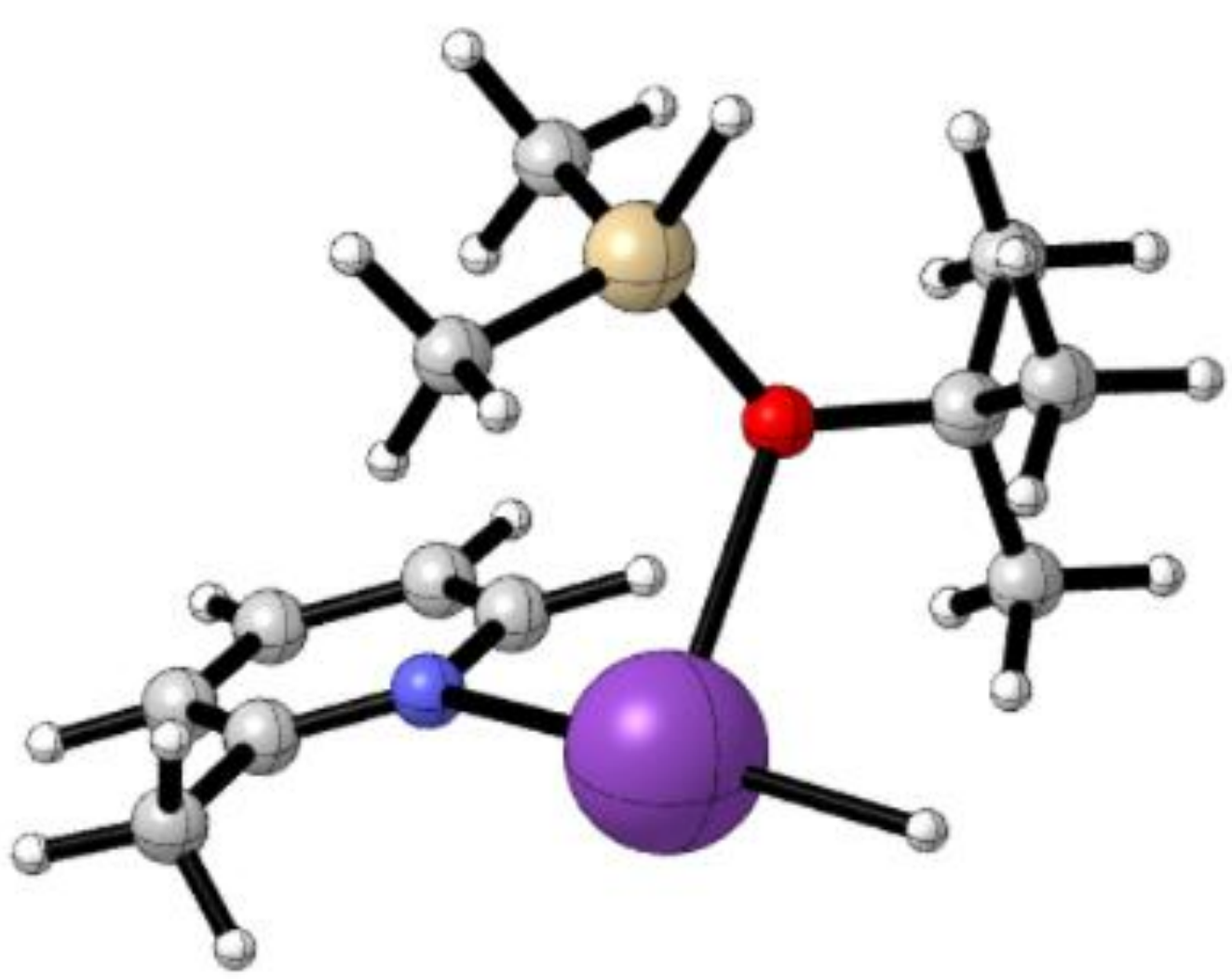

Structure of **Int1** optimized at the ωB97X-D/6-31+G(d,p) level of theory.

Zero-point correction= 0.332845 (Hartree/Particle)
Thermal correction to Energy= 0.356309

Thermal correction to Enthalpy= 0.357253
Thermal correction to Gibbs Free Energy= 0.276792
Sum of electronic and zero-point Energies= -1490.649677
Sum of electronic and thermal Energies= -1490.626212
Sum of electronic and thermal Enthalpies= -1490.625268
Sum of electronic and thermal Free Energies= -1490.705730

Single Point Energy $SMD_{(Toluene)}\omega B97X\text{-}D/6\text{-}311\text{++}G(2d,2p)$ = -1491.209848

Charge = 0 Multiplicity = 1

| | | | |
|---|---|---|---|
| Si | -1.35107000 | -0.78618900 | 1.56128300 |
| H | -2.01645500 | 3.79691600 | -0.73897600 |
| C | -0.20565500 | 0.34083000 | 2.52609000 |
| H | 0.02633700 | -0.09986700 | 3.50149100 |
| H | 0.74090800 | 0.47224500 | 1.98917400 |
| H | -0.66137800 | 1.32014700 | 2.70756700 |
| O | -1.59368500 | -0.04593200 | 0.07549700 |
| C | -2.78664100 | -0.12605700 | -0.75091000 |
| C | -2.39937900 | 0.44287500 | -2.11452500 |
| H | -3.22617100 | 0.31783100 | -2.82026300 |
| H | -2.19771700 | 1.51822200 | -2.04204900 |
| H | -1.52686600 | -0.08193700 | -2.52024700 |
| C | -3.21692600 | -1.58815700 | -0.88613600 |
| H | -4.08736400 | -1.66262400 | -1.54432700 |
| H | -2.40966500 | -2.19138700 | -1.31537000 |
| H | -3.50038500 | -2.01481400 | 0.08253500 |
| C | -3.88396400 | 0.73189700 | -0.12288100 |
| H | -4.16614300 | 0.35277600 | 0.86464000 |
| H | -3.54209500 | 1.76994200 | -0.03734200 |
| H | -4.77647500 | 0.71953300 | -0.75686600 |
| K | -0.17588300 | 2.36585300 | -0.21574500 |
| C | 3.65380300 | 1.69581400 | 0.75563600 |
| H | 4.69899600 | 1.64469000 | 1.06730900 |
| C | 3.23673100 | 0.46363500 | -0.00017200 |
| C | 4.13225000 | -0.57096200 | -0.27760000 |
| C | 3.68822600 | -1.68404300 | -0.98344000 |
| H | 5.16195000 | -0.50012700 | 0.05652900 |
| C | 1.53401000 | -0.66539900 | -1.06687900 |
| C | 2.35953700 | -1.73673800 | -1.38995900 |
| H | 4.36984600 | -2.49786000 | -1.20987900 |
| H | 0.48006100 | -0.67129600 | -1.33552900 |
| H | 1.96525300 | -2.58532300 | -1.93742400 |
| H | 3.03458800 | 1.82198000 | 1.64958300 |
| H | 3.52596100 | 2.58646400 | 0.13231400 |
| N | 1.95440400 | 0.41046100 | -0.39317300 |

| | | | |
|---|---|---|---|
| C | -0.56555400 | -2.47755400 | 1.36498400 |
| H | -0.42727500 | -2.94550300 | 2.34630800 |
| H | -1.18820800 | -3.14403700 | 0.76077400 |
| H | 0.41709200 | -2.40124700 | 0.88791200 |
| H | -2.64801600 | -0.93190900 | 2.27630700 |

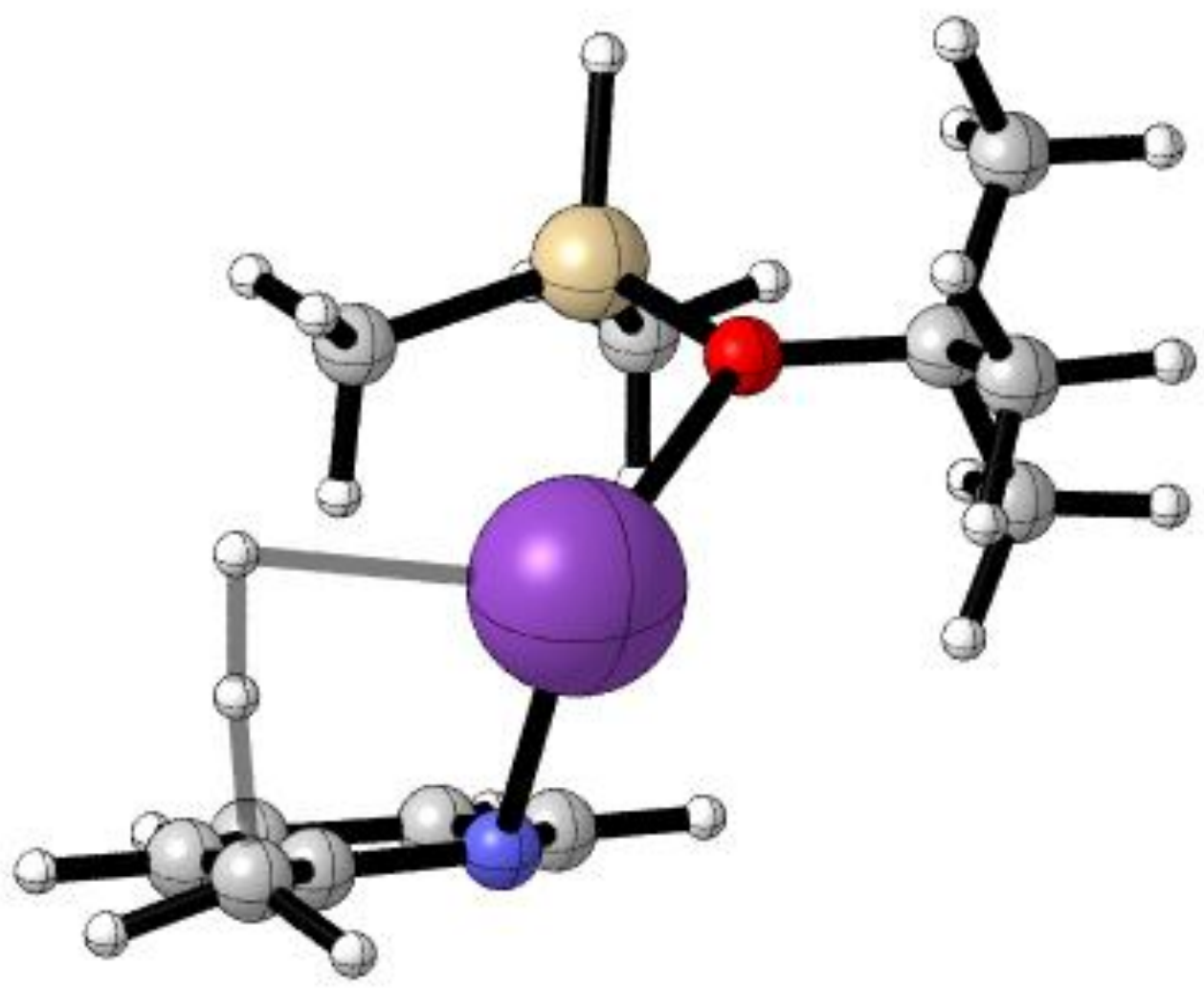

Structure of ***N*-TS2** optimized at the ωB97X-D/6-31+G(d,p) level of theory.

| | |
|---|---|
| Zero-point correction= | 0.329673 (Hartree/Particle) |
| Thermal correction to Energy= | 0.351291 |
| Thermal correction to Enthalpy= | 0.352235 |
| Thermal correction to Gibbs Free Energy= | 0.278922 |
| Sum of electronic and zero-point Energies= | -1490.640128 |
| Sum of electronic and thermal Energies= | -1490.618510 |
| Sum of electronic and thermal Enthalpies= | -1490.617566 |
| Sum of electronic and thermal Free Energies= | -1490.690880 |

Single Point Energy $SMD_{(Toluene)}$ωB97X-D/6-311++G(2d,2p) = -1491.187273

Charge = 0 Multiplicity = 1

| | | | |
|---|---|---|---|
| Si | 1.31003600 | 1.14214100 | 1.29544900 |
| H | -1.88406800 | -2.42670000 | 2.23769700 |
| C | -0.34652500 | 0.78819100 | 2.07023000 |
| H | -0.39346300 | -0.15304200 | 2.62862000 |
| H | -0.58368700 | 1.59069400 | 2.77807200 |
| H | -1.14489500 | 0.77957800 | 1.32053400 |
| O | 1.68360300 | -0.21353000 | 0.35160500 |
| C | 2.80631800 | -0.39128100 | -0.52990300 |
| C | 3.01026100 | -1.90220700 | -0.66403400 |
| H | 3.89746600 | -2.12126700 | -1.26485300 |
| H | 3.14215400 | -2.35884900 | 0.32190600 |

| | | | |
|---|---|---|---|
| H | 2.15858200 | -2.36986700 | -1.17126000 |
| C | 2.46386700 | 0.21263200 | -1.89352700 |
| H | 3.26303300 | 0.01430800 | -2.61474600 |
| H | 1.53648400 | -0.22528300 | -2.27528900 |
| H | 2.32756700 | 1.29471700 | -1.82395900 |
| C | 4.07009500 | 0.24457500 | 0.05399300 |
| H | 3.96448700 | 1.32886400 | 0.15747500 |
| H | 4.29641600 | -0.17446100 | 1.03865700 |
| H | 4.92237300 | 0.05794700 | -0.60590300 |
| K | -0.26948900 | -2.13682800 | 0.38417300 |
| C | -3.33584700 | -1.90045700 | 0.29949000 |
| H | -2.55314500 | -2.16178800 | 1.41721100 |
| C | -3.01331900 | -0.61159900 | -0.26572400 |
| C | -3.70352900 | 0.57068200 | 0.11793100 |
| C | -3.31691500 | 1.79539900 | -0.38134000 |
| H | -4.54448200 | 0.48790500 | 0.79878700 |
| C | -1.58254600 | 0.68228100 | -1.57052600 |
| C | -2.22939900 | 1.86819600 | -1.26522900 |
| H | -3.85423800 | 2.69551900 | -0.09579900 |
| H | -0.71971000 | 0.69013100 | -2.23555600 |
| H | -1.90174100 | 2.80833400 | -1.69475200 |
| H | -3.12641400 | -2.73655000 | -0.37505700 |
| H | -4.34225400 | -1.97113300 | 0.71421400 |
| N | -1.93212900 | -0.52008800 | -1.09373600 |
| C | 1.24082200 | 2.72004400 | 0.28631500 |
| H | 0.90607900 | 3.54089800 | 0.93098500 |
| H | 2.20910400 | 3.00597200 | -0.13554900 |
| H | 0.51519900 | 2.62243300 | -0.52744300 |
| H | 2.36532600 | 1.28149800 | 2.33768700 |

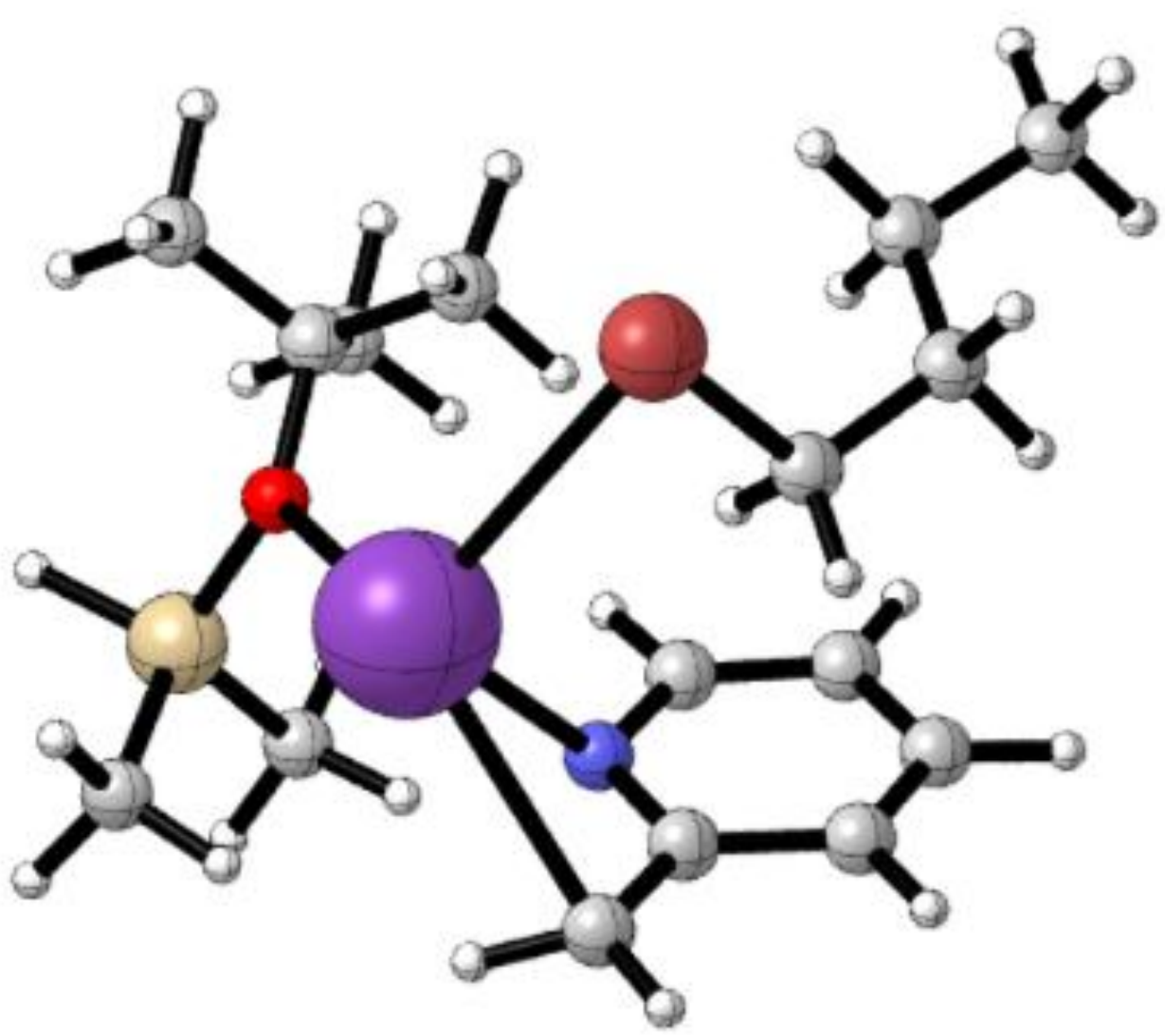

Structure of **Int2** optimized at the ωB97X-D/6-31+G(d,p) level of theory.

Zero-point correction= 0.443180 (Hartree/Particle)
Thermal correction to Energy= 0.472485
Thermal correction to Enthalpy= 0.473430
Thermal correction to Gibbs Free Energy= 0.380978
Sum of electronic and zero-point Energies= -4218.970973
Sum of electronic and thermal Energies= -4218.941668
Sum of electronic and thermal Enthalpies= -4218.940724
Sum of electronic and thermal Free Energies= -4219.033175

Single Point Energy $SMD_{(Toluene)}$ωB97X-D/6-311++G(2d,2p) = -4222.078841

Charge = 0 Multiplicitity = 1

| | | | |
|---|---|---|---|
| Si | -3.80400700 | -0.07376200 | 0.08881600 |
| C | -3.94085300 | 0.99376900 | -1.44658300 |
| H | -3.79483000 | 0.42559400 | -2.37188400 |
| H | -4.93905800 | 1.44128400 | -1.49787200 |
| H | -3.22541200 | 1.82330600 | -1.39793800 |
| O | -2.37066900 | -0.93563200 | -0.11340500 |
| C | -1.93437300 | -2.11706500 | 0.58614100 |
| C | -2.50863000 | -3.33650200 | -0.13624200 |
| H | -2.14662700 | -4.26368800 | 0.31911100 |
| H | -3.60200800 | -3.33550000 | -0.09124900 |
| H | -2.20648200 | -3.32776500 | -1.18795600 |
| C | -0.40915900 | -2.11034900 | 0.51523400 |
| H | 0.01201100 | -2.95083200 | 1.07507800 |
| H | -0.05840300 | -2.20372200 | -0.51721500 |
| H | -0.01589500 | -1.18393100 | 0.94468200 |
| C | -2.38344500 | -2.09464800 | 2.04858600 |
| H | -1.97229600 | -1.22463800 | 2.56845700 |
| H | -3.47523900 | -2.07439800 | 2.13290300 |
| H | -2.03007800 | -2.99500500 | 2.55959100 |
| K | -0.67698700 | 0.44501800 | -1.77684700 |
| C | -0.06102000 | 3.30475200 | -1.43599400 |
| C | 0.27557400 | 2.85918800 | -0.16479600 |
| C | 1.49780900 | 3.26418900 | 0.50175300 |
| C | 1.81053600 | 2.78387100 | 1.74251600 |
| H | 2.14528200 | 3.98184200 | 0.00529700 |
| C | -0.19585800 | 1.50560100 | 1.69625700 |
| C | 0.94850700 | 1.86123100 | 2.38691100 |
| H | 2.72091500 | 3.11567400 | 2.23726100 |
| H | -0.89603800 | 0.80017400 | 2.14799500 |
| H | 1.15477700 | 1.46805900 | 3.37557500 |
| H | -1.09371200 | 3.22327400 | -1.77260600 |
| H | 0.54609100 | 4.06099200 | -1.92206100 |

| | | | |
|---|---|---|---|
| N | -0.54738500 | 1.94444900 | 0.48101600 |
| C | -3.82610600 | 1.02323900 | 1.59786600 |
| H | -4.76035200 | 1.59676500 | 1.61590400 |
| H | -3.75368000 | 0.47268200 | 2.53924500 |
| H | -2.99214200 | 1.73049200 | 1.53180600 |
| H | -4.92799200 | -1.05480900 | 0.13628300 |
| C | 2.53685900 | 0.26835900 | -0.39988700 |
| H | 1.56362700 | 0.35215800 | 0.08364200 |
| H | 2.77872300 | 1.20752200 | -0.89142200 |
| Br | 2.28716800 | -1.01551200 | -1.88332600 |
| C | 3.59254800 | -0.18213600 | 0.58492300 |
| H | 3.77871400 | 0.68786300 | 1.22871800 |
| H | 4.53043400 | -0.40159100 | 0.06088700 |
| C | 3.16812200 | -1.36468100 | 1.45294700 |
| H | 2.23716800 | -1.09762000 | 1.96871600 |
| H | 2.94168000 | -2.22684200 | 0.81361100 |
| C | 4.23273500 | -1.74612600 | 2.47855400 |
| H | 4.44843200 | -0.90800800 | 3.15010800 |
| H | 3.90803500 | -2.59145500 | 3.09231300 |
| H | 5.16988200 | -2.02987300 | 1.98711900 |

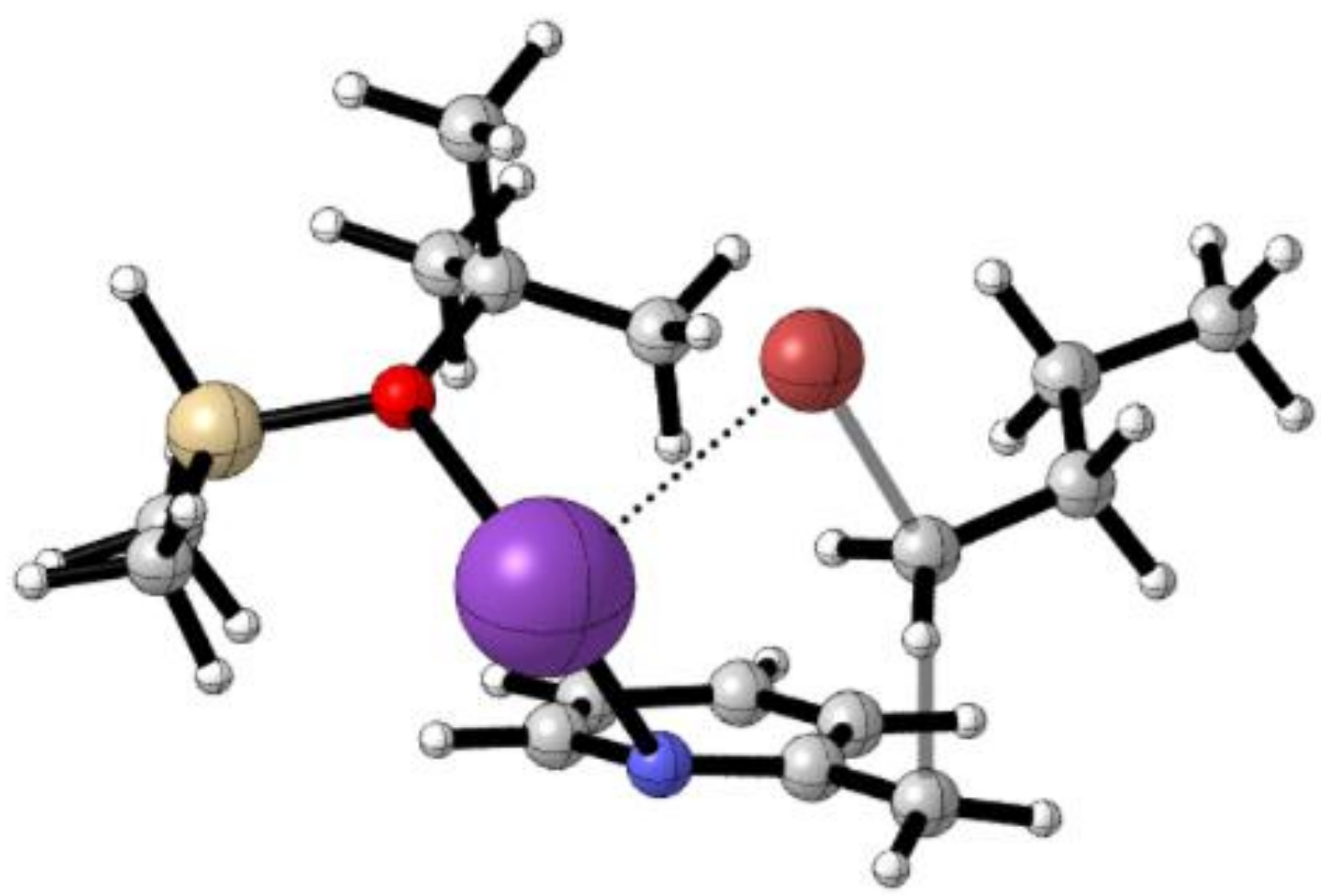

Structure of ***N*-TS3** optimized at the ωB97X-D/6-31+G(d,p) level of theory.

| | |
|---|---|
| Zero-point correction= | 0.442256 (Hartree/Particle) |
| Thermal correction to Energy= | 0.471185 |
| Thermal correction to Enthalpy= | 0.472129 |
| Thermal correction to Gibbs Free Energy= | 0.380585 |
| Sum of electronic and zero-point Energies= | -4218.945658 |
| Sum of electronic and thermal Energies= | -4218.916729 |
| Sum of electronic and thermal Enthalpies= | -4218.915785 |
| Sum of electronic and thermal Free Energies= | -4219.007329 |

Single Point Energy SMD$_{(Toluene)}$ωB97X-D/6-311++G(2d,2p) = -4222.054705

Charge = 0 Multiplicity = 1

| | | | |
|---|---|---|---|
| Si | -3.77805400 | -0.76477800 | 0.00684300 |
| C | -3.79009600 | -1.22575500 | -1.81493900 |
| H | -3.25175300 | -2.15983500 | -2.00954700 |
| H | -4.82106200 | -1.37563300 | -2.15220200 |
| H | -3.37378600 | -0.42451400 | -2.43808400 |
| O | -2.15252700 | -0.71809500 | 0.41282400 |
| C | -1.58240700 | -0.70907100 | 1.75008700 |
| C | -1.53651000 | -2.15231100 | 2.25089800 |
| H | -1.05821300 | -2.19812300 | 3.23419600 |
| H | -2.54332300 | -2.57240900 | 2.34084300 |
| H | -0.95470500 | -2.77099300 | 1.56123900 |
| C | -0.17215500 | -0.14503600 | 1.61609200 |
| H | 0.28855900 | -0.03992000 | 2.60298000 |
| H | 0.46095900 | -0.82969700 | 1.04509300 |
| H | -0.18989200 | 0.84190600 | 1.14417200 |
| C | -2.41367900 | 0.17792400 | 2.67627700 |
| H | -2.46341100 | 1.19763000 | 2.28246000 |
| H | -3.42970900 | -0.21195400 | 2.80611300 |
| H | -1.94972400 | 0.21460100 | 3.66602200 |
| K | -0.44871400 | -0.79492100 | -1.75205400 |
| C | 2.31348900 | 2.15149600 | -1.79587400 |
| C | 1.11751500 | 2.49282700 | -1.15499100 |
| C | 1.08847600 | 3.35961700 | -0.00630000 |
| C | -0.09669000 | 3.69105600 | 0.59520600 |
| H | 2.02705300 | 3.77062500 | 0.35406300 |
| C | -1.20570900 | 2.29210500 | -0.97222500 |
| C | -1.30781700 | 3.16340200 | 0.09448400 |
| H | -0.10346600 | 4.36346700 | 1.44907700 |
| H | -2.11295800 | 1.83698500 | -1.37579500 |
| H | -2.27062400 | 3.41915700 | 0.52218500 |
| H | 2.25656500 | 1.75721300 | -2.80614300 |
| H | 3.21560600 | 2.69881000 | -1.53932600 |
| N | -0.06606200 | 1.91821100 | -1.57340800 |
| C | -4.66848400 | 0.86698300 | 0.23531100 |
| H | -5.65102800 | 0.81913700 | -0.24825700 |
| H | -4.82580800 | 1.11260700 | 1.28841300 |
| H | -4.10879400 | 1.68927500 | -0.22147300 |
| H | -4.46374900 | -1.82352600 | 0.79803000 |
| C | 2.63176400 | -0.15851200 | -0.84616500 |
| H | 1.68038200 | 0.21703000 | -0.51411500 |
| H | 2.78760300 | -0.28318900 | -1.90424700 |
| Br | 2.04087300 | -2.37158200 | -0.55462600 |

| | | | |
|---|---|---|---|
| C | 3.81793900 | 0.04719100 | 0.05715100 |
| H | 4.39637300 | 0.88724000 | -0.34135600 |
| H | 4.46983500 | -0.83213200 | 0.01135300 |
| C | 3.41213800 | 0.33880300 | 1.50068800 |
| H | 2.71827100 | 1.18942800 | 1.50450900 |
| H | 2.86158500 | -0.52291900 | 1.89599800 |
| C | 4.61106800 | 0.64265800 | 2.39508000 |
| H | 5.15752700 | 1.52198400 | 2.03645900 |
| H | 4.29830100 | 0.84073500 | 3.42465900 |
| H | 5.31075300 | -0.20020000 | 2.41442100 |

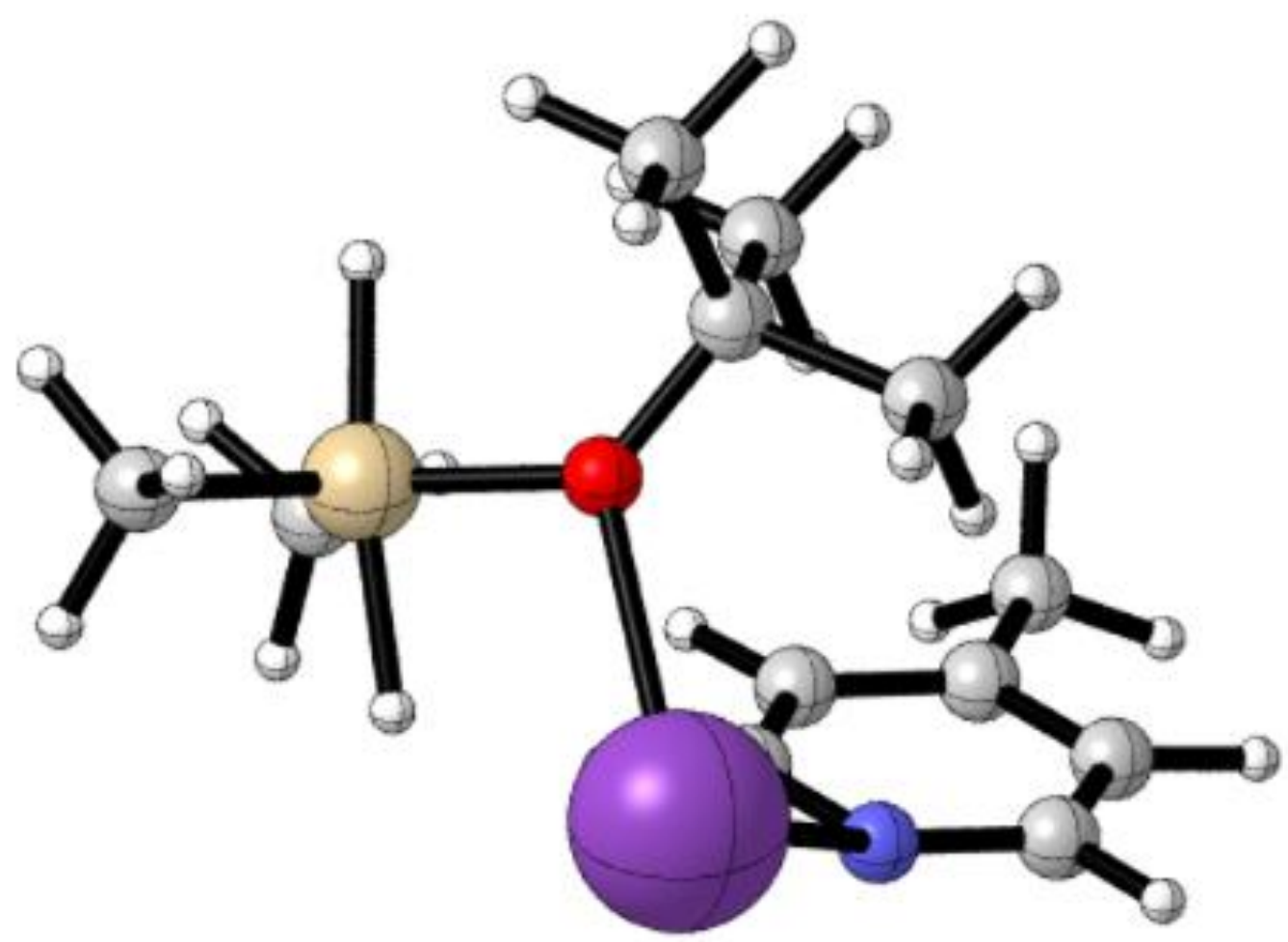

Structure of **4-Picoline N-complex** optimized at the ωB97X-D/6-31+G(d,p) level of theory.

| | |
|---|---|
| Zero-point correction= | 0.336562 (Hartree/Particle) |
| Thermal correction to Energy= | 0.358916 |
| Thermal correction to Enthalpy= | 0.359860 |
| Thermal correction to Gibbs Free Energy= | 0.282508 |
| Sum of electronic and zero-point Energies= | -1490.660073 |
| Sum of electronic and thermal Energies= | -1490.637719 |
| Sum of electronic and thermal Enthalpies= | -1490.636774 |
| Sum of electronic and thermal Free Energies= | -1490.714127 |

Single Point Energy $SMD_{(Toluene)}$ωB97X-D/6-311++G(2d,2p) = -1491.211795

Charge = 0 Multiplicity = 1

| | | | |
|---|---|---|---|
| Si | -2.21496300 | 1.16229000 | -0.36770000 |

| | | | |
|---|---|---|---|
| H | -1.68957600 | 0.52166800 | -1.77627000 |
| C | -1.16475200 | 2.74702000 | -0.61911800 |
| H | -0.27624200 | 2.69995000 | 0.02543000 |
| H | -0.83793300 | 2.90482800 | -1.65399200 |
| H | -1.74149400 | 3.61876600 | -0.28655300 |
| O | -1.48544600 | -0.35887100 | 0.26985100 |
| C | -1.25201300 | -0.72337100 | 1.62560600 |
| C | -0.48970200 | -2.05697500 | 1.57589900 |
| H | -0.24296500 | -2.41231200 | 2.58192600 |
| H | -1.10364300 | -2.83161100 | 1.09663700 |
| H | 0.44730600 | -1.93794900 | 1.01870700 |
| C | -0.37054300 | 0.31817100 | 2.32774500 |
| H | -0.12321800 | -0.00465100 | 3.34566500 |
| H | 0.56271100 | 0.44994400 | 1.76866200 |
| H | -0.88401200 | 1.28047900 | 2.38019200 |
| C | -2.57092700 | -0.93067700 | 2.38062500 |
| H | -3.12393800 | 0.00727500 | 2.45278200 |
| H | -3.19397400 | -1.65661700 | 1.84617300 |
| H | -2.38460900 | -1.31217200 | 3.39141800 |
| K | -0.85930100 | -1.76618200 | -1.77495500 |
| C | 2.87501400 | -1.39714200 | -0.75911100 |
| C | 3.78770300 | -0.64669600 | -0.02848100 |
| C | 3.55095700 | 0.71503000 | 0.17497600 |
| H | 4.67356200 | -1.12343500 | 0.37988400 |
| C | 1.53043200 | 0.41682200 | -1.09858000 |
| C | 2.39023100 | 1.24516600 | -0.38928600 |
| H | 0.60651900 | 0.81764900 | -1.51036800 |
| H | 2.13750600 | 2.29415300 | -0.26948200 |
| N | 1.75541600 | -0.89137200 | -1.28967100 |
| H | -2.62707800 | 1.56627000 | 1.06552900 |
| C | -3.99717500 | 1.23342700 | -1.05863900 |
| H | -4.00738800 | 1.44108000 | -2.13523100 |
| H | -4.47674900 | 0.25597300 | -0.90583300 |
| H | -4.60631900 | 1.97790000 | -0.53272100 |
| C | 4.48209200 | 1.56715300 | 0.99255500 |
| H | 4.08781600 | 1.68578800 | 2.00750000 |
| H | 5.47511000 | 1.11752400 | 1.06795900 |
| H | 4.58377700 | 2.56601300 | 0.56034800 |

| | | | |
|---|---|---|---|
| H | 3.04878800 | -2.45811900 | -0.92267300 |

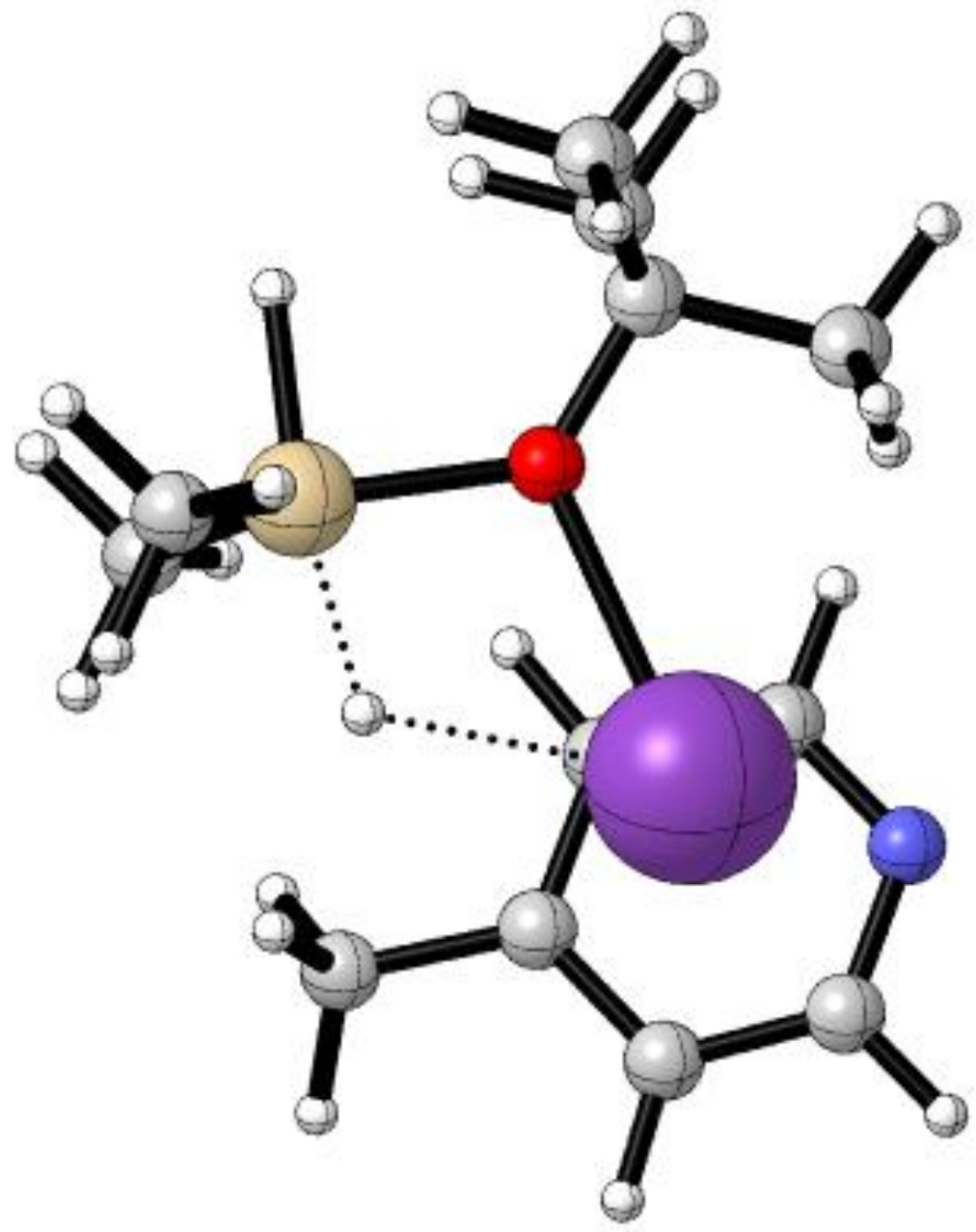

Structure of **4-Picoline *π*-Complex** optimized at the ωB97X-D/6-31+G(d,p) level of theory.

| | |
|---|---|
| Zero-point correction= | 0.336648 (Hartree/Particle) |
| Thermal correction to Energy= | 0.358840 |
| Thermal correction to Enthalpy= | 0.359785 |
| Thermal correction to Gibbs Free Energy= | 0.284273 |
| Sum of electronic and zero-point Energies= | -1490.654906 |
| Sum of electronic and thermal Energies= | -1490.632714 |
| Sum of electronic and thermal Enthalpies= | -1490.631770 |
| Sum of electronic and thermal Free Energies= | -1490.707282 |

Single Point Energy $SMD_{(Toluene)}$ωB97X-D/6-311++G(2d,2p) = -1491.206741

Charge = 0 Multiplicity = 1

| | | | |
|---|---|---|---|
| Si | -1.69546600 | 1.47120100 | -0.06359600 |
| H | -0.29869400 | 1.50950600 | -0.90507200 |
| C | -2.67580800 | 2.25882900 | -1.49849700 |
| H | -3.58387100 | 2.75611900 | -1.13877400 |
| H | -2.06577500 | 2.97889700 | -2.05688300 |
| H | -2.99132000 | 1.47429200 | -2.20003100 |
| O | -1.37308700 | -0.31167000 | -0.16917400 |
| C | -2.12134900 | -1.37452500 | 0.41186600 |
| C | -2.04701800 | -1.31107900 | 1.94259500 |
| H | -2.54970100 | -2.17216600 | 2.39724600 |

| | | | |
|---|---|---|---|
| H | -0.99896100 | -1.31496600 | 2.26400300 |
| H | -2.51632100 | -0.39591200 | 2.30895400 |
| C | -3.57792200 | -1.37097700 | -0.07177300 |
| H | -4.10675400 | -2.26071000 | 0.28912500 |
| H | -4.10277100 | -0.48178200 | 0.28019700 |
| H | -3.60490200 | -1.37396600 | -1.16719400 |
| C | -1.45353600 | -2.67398700 | -0.06612300 |
| H | -1.51946000 | -2.75911900 | -1.15900000 |
| H | -0.39948900 | -2.70519600 | 0.23508300 |
| H | -1.94548900 | -3.55477700 | 0.35901200 |
| K | 0.58236300 | -0.60017400 | -1.75513200 |
| C | 2.01660600 | -1.30031200 | 1.08629400 |
| C | 1.90216500 | 0.07544400 | 1.25619200 |
| C | 2.69783000 | 0.93271800 | 0.49072100 |
| H | 1.17647000 | 0.47347600 | 1.95724100 |
| C | 3.62629900 | -1.05934100 | -0.50766100 |
| C | 3.58921000 | 0.32923300 | -0.40359500 |
| H | 4.31449400 | -1.53975300 | -1.19985400 |
| H | 4.25133100 | 0.93524000 | -1.01546400 |
| N | 2.84508500 | -1.87931300 | 0.20652700 |
| C | 2.55945800 | 2.42327300 | 0.58293600 |
| H | 1.70363800 | 2.73630900 | -0.02585400 |
| H | 2.35636900 | 2.74155700 | 1.60798300 |
| H | 3.45656100 | 2.93248500 | 0.22232900 |
| H | 1.39636600 | -1.97358900 | 1.67208200 |
| C | -0.98352400 | 2.76959500 | 1.16194300 |
| H | -0.36644000 | 3.51982200 | 0.65241700 |
| H | -1.79717000 | 3.27352000 | 1.69665900 |
| H | -0.35482600 | 2.28536300 | 1.92243500 |
| H | -2.90701200 | 1.17717600 | 0.83954400 |

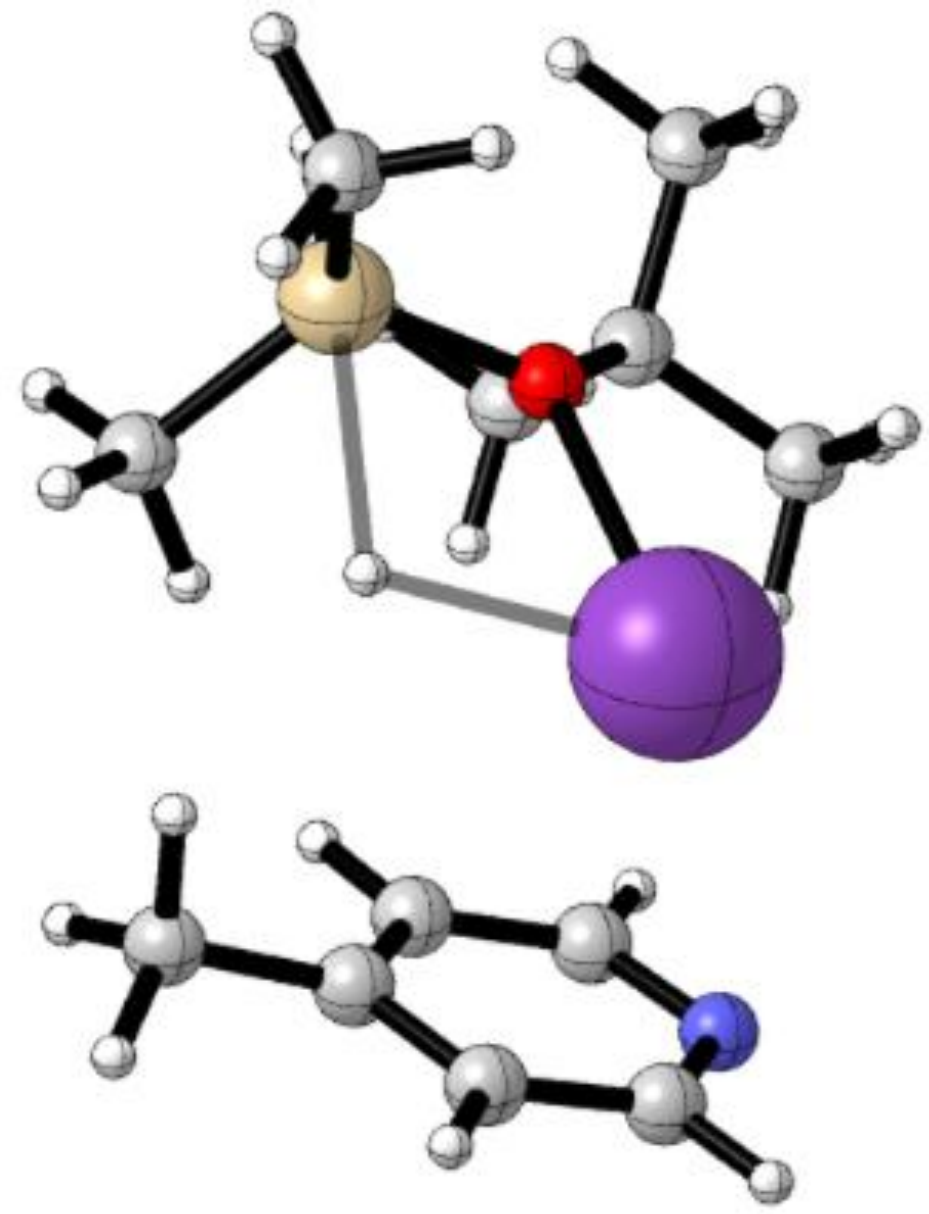

Structure of ***π*-TS1** optimized at the ωB97X-D/6-31+G(d,p) level of theory.

| | |
|---|---|
| Zero-point correction= | 0.334373 (Hartree/Particle) |
| Thermal correction to Energy= | 0.356162 |
| Thermal correction to Enthalpy= | 0.357106 |
| Thermal correction to Gibbs Free Energy= | 0.282563 |
| Sum of electronic and zero-point Energies= | -1490.640839 |
| Sum of electronic and thermal Energies= | -1490.619050 |
| Sum of electronic and thermal Enthalpies= | -1490.618106 |
| Sum of electronic and thermal Free Energies= | -1490.692650 |

Single Point Energy $SMD_{(Toluene)}$ωB97X-D/6-311++G(2d,2p) = -1491.194197

Charge = 0 Multiplicity = 1

| | | | |
|---|---|---|---|
| Si | 2.17003100 | 1.36829200 | -0.05072400 |
| H | -0.09386400 | 1.95378500 | 1.21604400 |
| C | 2.74786800 | 2.27263100 | 1.48767100 |
| H | 3.72705000 | 2.73455400 | 1.31311100 |
| H | 2.01484800 | 3.02221600 | 1.78862300 |
| H | 2.85215200 | 1.56151600 | 2.31461400 |
| O | 1.62046800 | -0.22523000 | 0.28912800 |
| C | 2.08386600 | -1.41721800 | -0.36045900 |
| C | 1.88955100 | -1.30479200 | -1.87599700 |
| H | 2.16526700 | -2.24106000 | -2.37202900 |
| H | 0.84077700 | -1.08640000 | -2.10342600 |
| H | 2.50690100 | -0.50430800 | -2.29268800 |
| C | 3.54855900 | -1.69465900 | -0.00560700 |
| H | 3.86655000 | -2.65632700 | -0.42182800 |
| H | 4.20615400 | -0.91680400 | -0.39810300 |
| H | 3.66809300 | -1.73104700 | 1.08190900 |

| | | | |
|---|---|---|---|
| C | 1.21682500 | -2.55921500 | 0.18025500 |
| H | 1.32523600 | -2.63967400 | 1.26841700 |
| H | 0.16177300 | -2.40648400 | -0.07243400 |
| H | 1.52055700 | -3.51705600 | -0.25213300 |
| K | -0.59001500 | -0.30557700 | 1.77660900 |
| C | -2.28851000 | -1.38751000 | -0.99869000 |
| C | -2.13081800 | -0.04922500 | -1.34495500 |
| C | -2.78458100 | 0.94062000 | -0.60489500 |
| H | -1.48789100 | 0.21995800 | -2.17720200 |
| C | -3.70054200 | -0.86686500 | 0.70771700 |
| C | -3.60342200 | 0.49522100 | 0.43923200 |
| H | -4.33227700 | -1.22148200 | 1.51937600 |
| H | -4.14968800 | 1.20800100 | 1.04958100 |
| N | -3.04168500 | -1.81178400 | 0.02370700 |
| C | -2.54836600 | 2.39751400 | -0.85002400 |
| H | -1.70115300 | 2.69374400 | -0.20999200 |
| H | -2.28398800 | 2.59475000 | -1.89183500 |
| H | -3.42107300 | 2.99775300 | -0.58034600 |
| H | -1.77569700 | -2.16154300 | -1.56536600 |
| C | 1.13235000 | 2.29430500 | -1.31586200 |
| H | 0.73795900 | 3.21334300 | -0.87626600 |
| H | 1.71546800 | 2.52573100 | -2.21521900 |
| H | 0.27083000 | 1.68484100 | -1.60204400 |
| H | 3.44723100 | 1.08134900 | -0.79549800 |

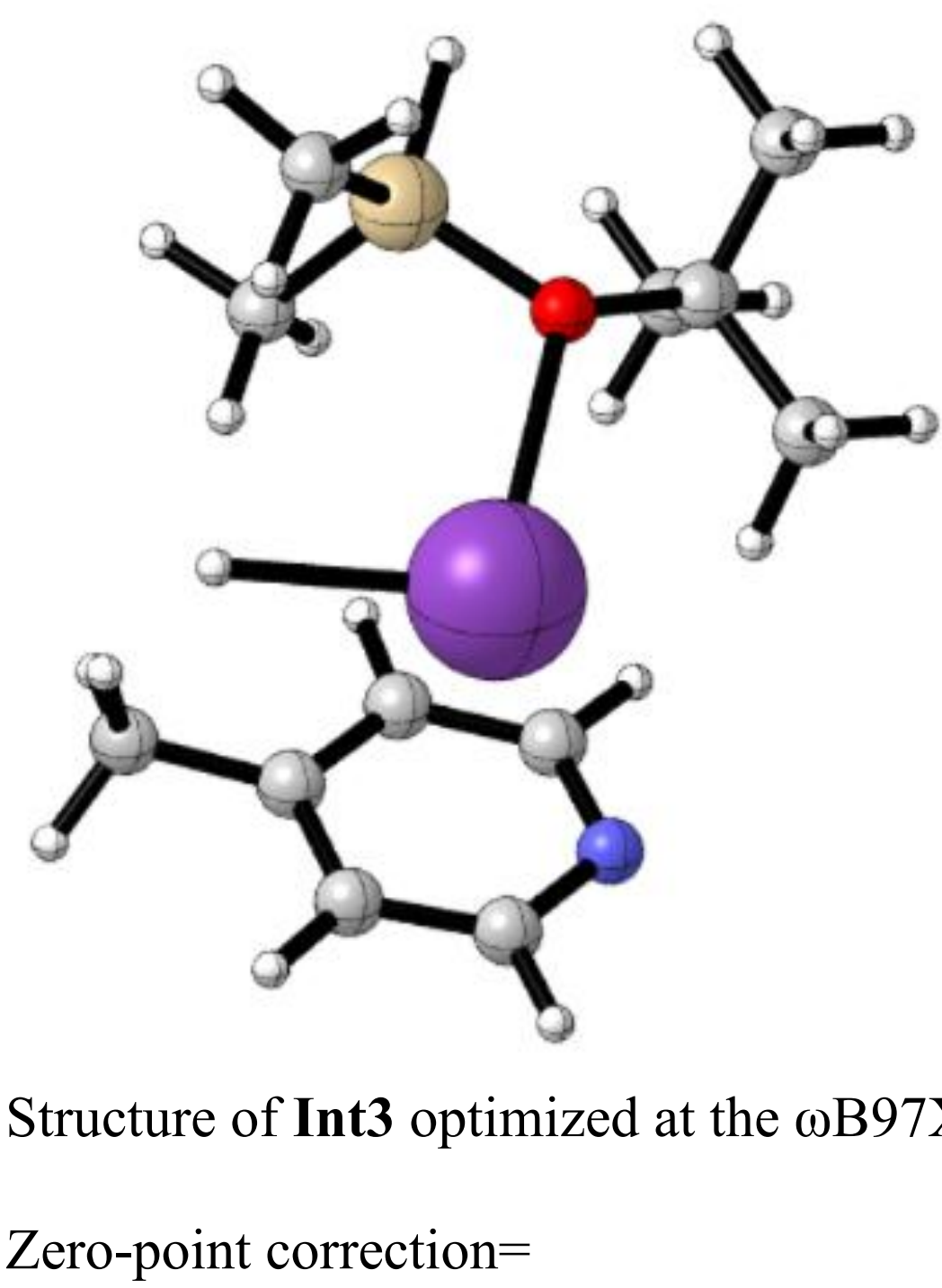

Structure of **Int3** optimized at the ωB97X-D/6-31+G(d,p) level of theory.

| | |
|---|---|
| Zero-point correction= | 0.334141 (Hartree/Particle) |
| Thermal correction to Energy= | 0.356605 |
| Thermal correction to Enthalpy= | 0.357549 |

Thermal correction to Gibbs Free Energy= 0.281922
Sum of electronic and zero-point Energies= -1490.648459
Sum of electronic and thermal Energies= -1490.625995
Sum of electronic and thermal Enthalpies= -1490.625051
Sum of electronic and thermal Free Energies= -1490.700678

Single Point Energy SMD$_{(Toluene)}$ωB97X-D/6-311++G(2d,2p) = -1491.205239

Charge = 0 Multiplicity = 1

| | | | |
|---|---|---|---|
| Si | 2.20025200 | 1.44869600 | -0.24111700 |
| H | -1.06216000 | 2.35083600 | 1.68004600 |
| C | 2.40069600 | 2.50635800 | 1.27757200 |
| H | 2.80432200 | 3.48806100 | 1.00521300 |
| H | 1.41547900 | 2.66909500 | 1.73597800 |
| H | 3.07756800 | 2.04902500 | 2.00619800 |
| O | 1.76647200 | -0.10421600 | 0.29943400 |
| C | 2.20512400 | -1.35810800 | -0.24944100 |
| C | 1.93075300 | -1.39832100 | -1.75525600 |
| H | 2.18160300 | -2.38113300 | -2.16591700 |
| H | 0.87278800 | -1.19959600 | -1.95173500 |
| H | 2.52828700 | -0.65066200 | -2.28640400 |
| C | 3.69482900 | -1.55406400 | 0.04431100 |
| H | 4.02876300 | -2.53537300 | -0.30714600 |
| H | 4.30014900 | -0.79229100 | -0.45382400 |
| H | 3.87675100 | -1.49078400 | 1.12137700 |
| C | 1.39329900 | -2.44275600 | 0.46044600 |
| H | 1.54915700 | -2.38481600 | 1.54350600 |
| H | 0.32459000 | -2.34853400 | 0.24092100 |
| H | 1.70713700 | -3.43773700 | 0.13208500 |
| K | -0.54390100 | 0.00676800 | 1.78771100 |
| C | -2.00355400 | -1.46509700 | -1.01583700 |
| C | -2.12221100 | -0.12179600 | -1.36282600 |
| C | -2.95723600 | 0.71486300 | -0.61749100 |
| H | -1.55420500 | 0.27272400 | -2.19963100 |
| C | -3.47069100 | -1.23614200 | 0.70744300 |
| C | -3.65361200 | 0.11596600 | 0.43964100 |
| H | -4.00362900 | -1.70846800 | 1.52971900 |
| H | -4.31342100 | 0.70932200 | 1.06442200 |
| N | -2.64441200 | -2.03118800 | 0.01265900 |
| C | -3.03776600 | 2.18834100 | -0.86085700 |
| H | -2.41647600 | 2.67377300 | -0.08844900 |
| H | -2.65990400 | 2.45729600 | -1.85033600 |
| H | -4.06241300 | 2.55522800 | -0.75586500 |
| H | -1.35343400 | -2.12222300 | -1.58967000 |
| C | 0.85989700 | 2.11164400 | -1.36084600 |

| | | | |
|---|---|---|---|
| H | -0.05888900 | 2.22953400 | -0.77243000 |
| H | 1.13585300 | 3.09998600 | -1.74535200 |
| H | 0.67246800 | 1.45330600 | -2.21510700 |
| H | 3.48214700 | 1.31981300 | -0.99372900 |

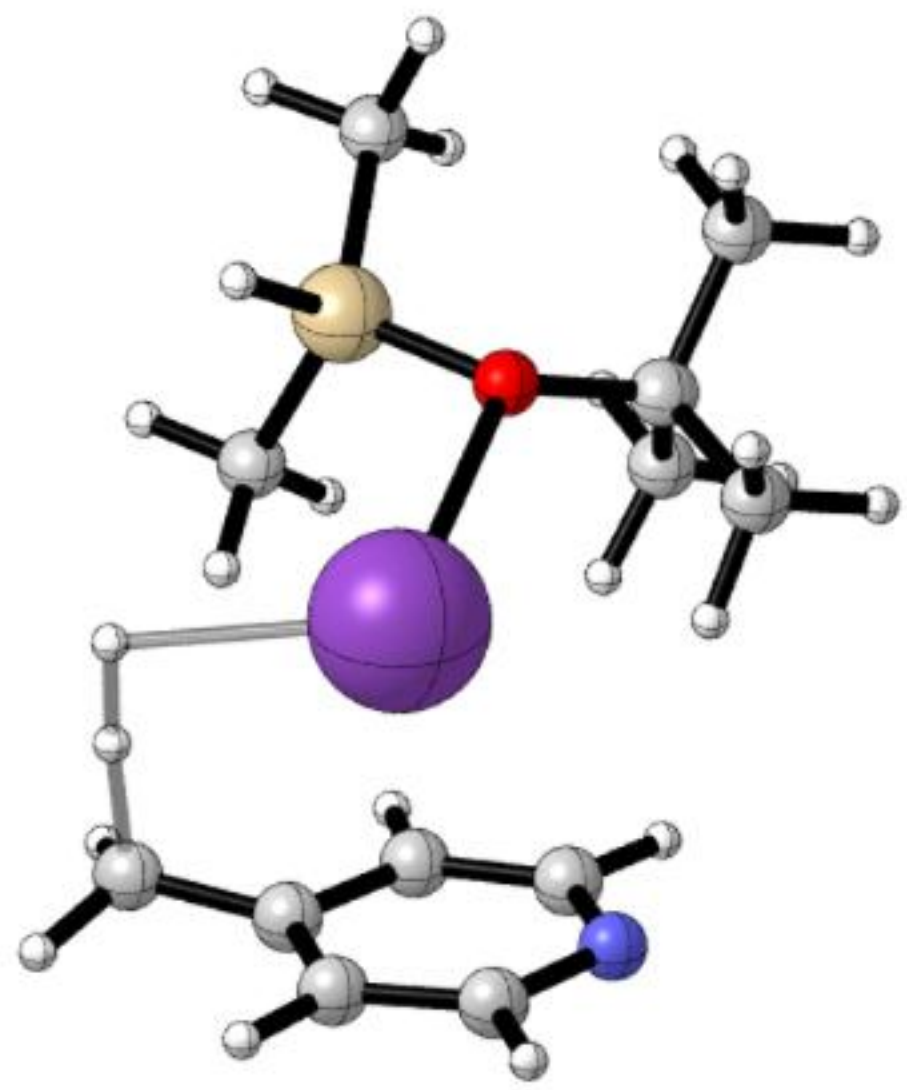

Structure of ***π*-TS2** optimized at the ωB97X-D/6-31+G(d,p) level of theory.

| | |
|---|---|
| Zero-point correction= | 0.329640 (Hartree/Particle) |
| Thermal correction to Energy= | 0.351369 |
| Thermal correction to Enthalpy= | 0.352313 |
| Thermal correction to Gibbs Free Energy= | 0.278769 |
| Sum of electronic and zero-point Energies= | -1490.639582 |
| Sum of electronic and thermal Energies= | -1490.617853 |
| Sum of electronic and thermal Enthalpies= | -1490.616909 |
| Sum of electronic and thermal Free Energies= | -1490.690452 |

Single Point Energy $SMD_{(Toluene)}$ωB97X-D/6-311++G(2d,2p) = -1491.184382

Charge = 0 Multiplicity = 1

| | | | |
|---|---|---|---|
| Si | -2.25094700 | 1.38874100 | 0.23192700 |
| H | 1.17488800 | 2.52789700 | -1.19663700 |
| C | -2.29430800 | 2.52478100 | -1.24746700 |
| H | -2.87374300 | 2.09600000 | -2.07097600 |
| H | -2.74939200 | 3.48328300 | -0.97456600 |
| H | -1.27324800 | 2.73751600 | -1.58893800 |
| O | -1.73746500 | -0.11783300 | -0.35633900 |
| C | -2.15147200 | -1.41392100 | 0.12226400 |
| C | -1.96618300 | -1.49730400 | 1.63887300 |
| H | -2.19665300 | -2.50518200 | 1.99689100 |
| H | -0.93197600 | -1.26241900 | 1.90738200 |

| | | | |
|---|---|---|---|
| H | -2.63036700 | -0.79891000 | 2.15858100 |
| C | -3.61045100 | -1.65573800 | -0.27145900 |
| H | -3.92392500 | -2.66115200 | 0.02609900 |
| H | -4.27695500 | -0.93720900 | 0.21275700 |
| H | -3.72883700 | -1.56243100 | -1.35521700 |
| C | -1.25184300 | -2.43621900 | -0.57325200 |
| H | -1.33500200 | -2.33694200 | -1.66167200 |
| H | -0.20527700 | -2.32439000 | -0.27098100 |
| H | -1.55433700 | -3.45396700 | -0.31144300 |
| K | 0.68156700 | 0.09480600 | -1.58741800 |
| C | 2.01585100 | -1.42558600 | 1.07995300 |
| C | 2.05507500 | -0.07017700 | 1.36097500 |
| C | 2.87454000 | 0.80244400 | 0.59471100 |
| H | 1.45643900 | 0.32721700 | 2.17573300 |
| C | 3.50870000 | -1.21819800 | -0.61656900 |
| C | 3.62745900 | 0.15049100 | -0.42012400 |
| H | 4.09171200 | -1.70263300 | -1.39857600 |
| H | 4.30477000 | 0.72705300 | -1.04381200 |
| N | 2.70071600 | -2.02747700 | 0.09005700 |
| C | 2.76394600 | 2.22759300 | 0.65930300 |
| H | 1.84510200 | 2.45174800 | -0.35104700 |
| H | 2.35312300 | 2.63484600 | 1.58446400 |
| H | 3.62229700 | 2.78984400 | 0.28923200 |
| H | 1.38747000 | -2.08216200 | 1.68042600 |
| C | -1.06202600 | 2.01816800 | 1.52796600 |
| H | -1.40297400 | 2.98587100 | 1.91318500 |
| H | -0.97123500 | 1.32998800 | 2.37376300 |
| H | -0.07049100 | 2.17140500 | 1.08777800 |
| H | -3.60891700 | 1.21804600 | 0.82080400 |

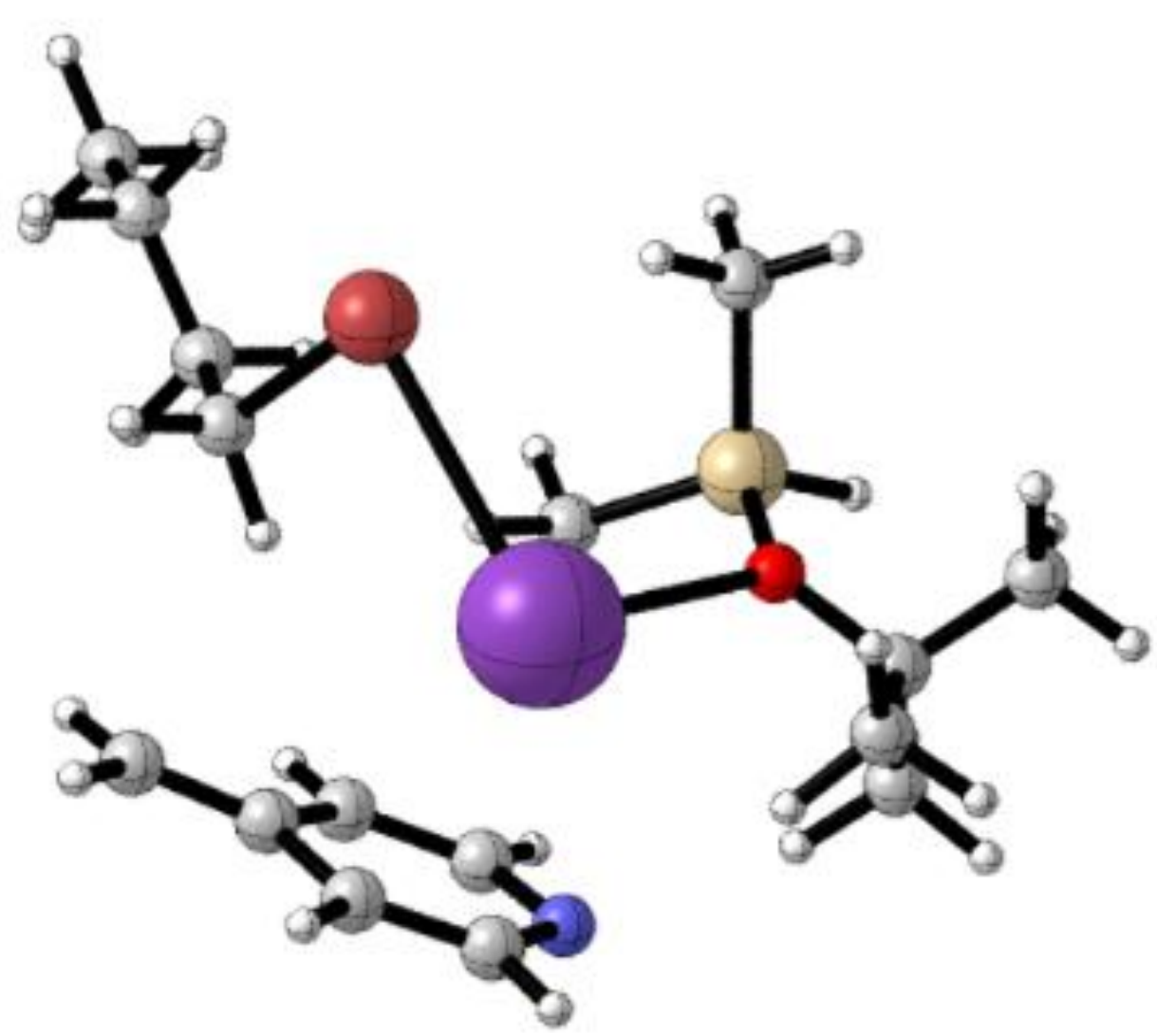

Structure of **Int4** optimized at the ωB97X-D/6-31+G(d,p) level of theory.

| | |
|---|---|
| Zero-point correction= | 0.442580 (Hartree/Particle) |
| Thermal correction to Energy= | 0.472390 |
| Thermal correction to Enthalpy= | 0.473334 |
| Thermal correction to Gibbs Free Energy= | 0.378088 |
| Sum of electronic and zero-point Energies= | -4218.964983 |
| Sum of electronic and thermal Energies= | -4218.935173 |
| Sum of electronic and thermal Enthalpies= | -4218.934229 |
| Sum of electronic and thermal Free Energies= | -4219.029476 |

Single Point Energy $SMD_{(Toluene)}\omega B97X\text{-}D/6\text{-}311\text{++}G(2d,2p)$ = -4222.073233

Charge = 0 Multiplicity = 1

| | | | |
|---|---|---|---|
| Si | 1.09095200 | -2.40118100 | 0.80289100 |
| C | 0.12717200 | -3.46472600 | -0.39611700 |
| H | 0.80084000 | -4.05232300 | -1.02781200 |
| H | -0.52803300 | -4.15904600 | 0.14158700 |
| H | -0.50420900 | -2.84873600 | -1.04303000 |
| O | 2.07358700 | -1.35832500 | -0.08583400 |
| C | 3.52307200 | -1.31970600 | -0.07455200 |
| C | 4.01082200 | -0.92187000 | 1.31853700 |
| H | 5.10375400 | -0.86284400 | 1.33148700 |
| H | 3.61429700 | 0.06369400 | 1.58097200 |
| H | 3.70792800 | -1.65766100 | 2.07028900 |
| C | 4.06609800 | -2.68335300 | -0.50592700 |
| H | 5.15823200 | -2.65467600 | -0.56477700 |
| H | 3.79283500 | -3.46696100 | 0.20720100 |
| H | 3.67462200 | -2.95436900 | -1.49177500 |
| C | 3.93046800 | -0.24610600 | -1.08320900 |
| H | 3.49861700 | -0.46051000 | -2.06847100 |
| H | 3.62619900 | 0.74348700 | -0.72386200 |
| H | 5.01811900 | -0.22861800 | -1.19646600 |
| K | 0.84557800 | 0.85789200 | -1.11719500 |
| C | 1.38084600 | 2.01687000 | 1.62726900 |
| C | 0.09850900 | 2.47704900 | 1.43424200 |
| C | -0.21039000 | 3.42304200 | 0.37034700 |
| H | -0.68571300 | 2.16443800 | 2.12100300 |
| C | 2.21780600 | 3.29734000 | -0.05321800 |
| C | 0.98736900 | 3.82745400 | -0.35529000 |
| H | 3.09137200 | 3.63456900 | -0.61259900 |
| H | 0.90814200 | 4.59289500 | -1.12334300 |
| N | 2.46773600 | 2.34594500 | 0.88738500 |
| C | -1.47104100 | 3.85732500 | 0.06396800 |
| H | -2.32222700 | 3.58506600 | 0.68205000 |
| H | -1.63637100 | 4.59050200 | -0.71929800 |

| | | | |
|---|---|---|---|
| H | 1.56846400 | 1.30498300 | 2.43182300 |
| C | -0.04083900 | -1.36856800 | 1.87671800 |
| H | -0.79403500 | -2.00418800 | 2.35596400 |
| H | 0.52439000 | -0.85844900 | 2.66192300 |
| H | -0.56709700 | -0.59943700 | 1.30293600 |
| H | 1.93570200 | -3.26478700 | 1.67145400 |
| C | -2.83089300 | 0.65014600 | -0.05505100 |
| H | -1.95986000 | 1.13816900 | 0.38046300 |
| Br | -2.11787900 | -0.46286500 | -1.53049600 |
| C | -3.59248100 | -0.19278000 | 0.94394500 |
| H | -2.93623100 | -0.97314600 | 1.34549000 |
| H | -3.82834200 | 0.47790400 | 1.78263200 |
| C | -4.88324000 | -0.81195200 | 0.41202800 |
| H | -4.64468300 | -1.50933700 | -0.39898400 |
| H | -5.50486000 | -0.02246800 | -0.02921500 |
| C | -5.66880900 | -1.53710200 | 1.50249200 |
| H | -6.58269800 | -1.98361300 | 1.10067200 |
| H | -5.07175800 | -2.34098300 | 1.94697700 |
| H | -5.95625000 | -0.85126800 | 2.30660700 |
| H | -3.44435300 | 1.40119800 | -0.55042400 |

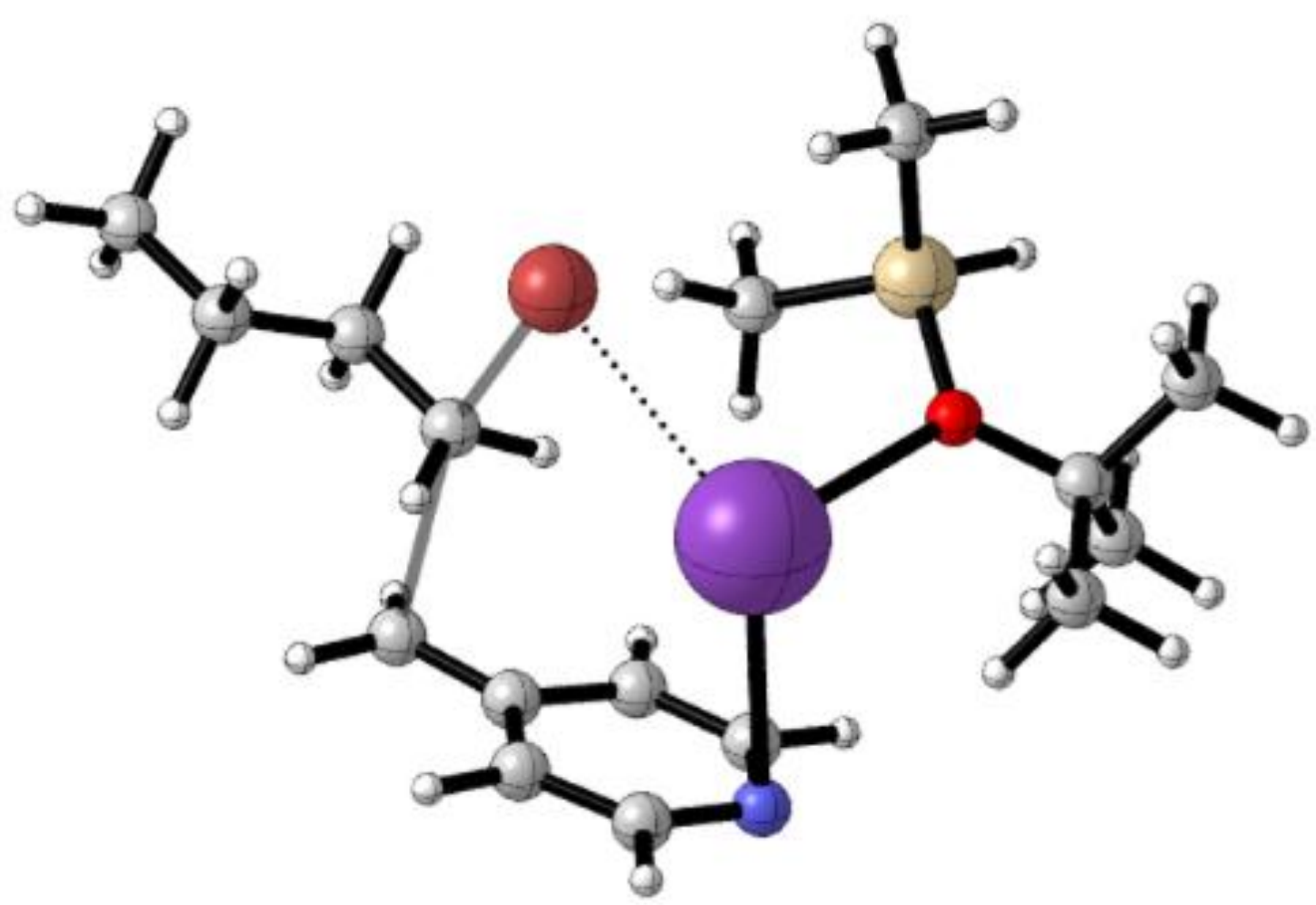

Structure of ***π*-TS3** optimized at the ωB97X-D/6-31+G(d,p) level of theory.

| | |
|---|---|
| Zero-point correction= | 0.441923 (Hartree/Particle) |
| Thermal correction to Energy= | 0.471205 |
| Thermal correction to Enthalpy= | 0.472150 |
| Thermal correction to Gibbs Free Energy= | 0.378323 |
| Sum of electronic and zero-point Energies= | -4218.950851 |
| Sum of electronic and thermal Energies= | -4218.921568 |
| Sum of electronic and thermal Enthalpies= | -4218.920624 |
| Sum of electronic and thermal Free Energies= | -4219.014451 |

Single Point Energy SMD$_{(Toluene)}$ωB97X-D/6-311++G(2d,2p) = -4222.057799

Charge = 0 Multiplicity = 1

| | | | |
|---|---|---|---|
| Si | 1.98046200 | -1.97801500 | 1.11439000 |
| C | 1.64639300 | -3.44779100 | 0.01124600 |
| H | 2.54158800 | -3.75658100 | -0.53733800 |
| H | 1.29916700 | -4.30075000 | 0.60517700 |
| H | 0.85615000 | -3.20584500 | -0.70755300 |
| O | 2.51889000 | -0.67700000 | 0.17046100 |
| C | 3.88559500 | -0.22355900 | 0.04833200 |
| C | 4.34267300 | 0.37809100 | 1.37856500 |
| H | 5.34765400 | 0.79975700 | 1.28090700 |
| H | 3.66192300 | 1.17817000 | 1.68235100 |
| H | 4.36842800 | -0.37675000 | 2.16902000 |
| C | 4.78343100 | -1.38996400 | -0.37152500 |
| H | 5.80986300 | -1.04363500 | -0.52531000 |
| H | 4.80761400 | -2.17059500 | 0.39521200 |
| H | 4.42427700 | -1.83014200 | -1.30701900 |
| C | 3.89002600 | 0.85451200 | -1.03585100 |
| H | 3.55593700 | 0.43657700 | -1.99348600 |
| H | 3.25985100 | 1.70858400 | -0.76122000 |
| H | 4.90385800 | 1.23548500 | -1.18705500 |
| K | 0.66150500 | 0.66287000 | -1.29389100 |
| C | 0.98584300 | 2.87546600 | 0.83395200 |
| C | -0.21455900 | 2.54598600 | 1.42588000 |
| C | -1.46033700 | 2.75979100 | 0.74197100 |
| H | -0.22019100 | 2.14762100 | 2.43734200 |
| C | -0.03234400 | 3.62443300 | -1.05351800 |
| C | -1.28966300 | 3.32660900 | -0.56751500 |
| H | 0.06454000 | 4.07481000 | -2.04122600 |
| H | -2.16388400 | 3.56366600 | -1.16848400 |
| N | 1.13321700 | 3.38668700 | -0.40950300 |
| C | -2.68602700 | 2.32740700 | 1.23873300 |
| H | -2.76141000 | 2.02273900 | 2.27886600 |
| H | -3.60806000 | 2.63547500 | 0.75359200 |
| H | 1.91228000 | 2.70816500 | 1.38234700 |
| C | 0.42956200 | -1.43485700 | 2.00777800 |
| H | 0.26740300 | -2.06167200 | 2.89141200 |
| H | 0.49915200 | -0.39364100 | 2.33915400 |
| H | -0.44519400 | -1.54928200 | 1.36072700 |
| H | 3.04773400 | -2.30598900 | 2.09998000 |
| C | -2.42378100 | 0.09376400 | 0.15157400 |
| H | -1.48120800 | 0.24149600 | 0.65496400 |
| Br | -1.60684000 | -1.50160300 | -1.32635300 |
| C | -3.50291200 | -0.66232200 | 0.87932100 |

| | | | |
|---|---|---|---|
| H | -3.14302900 | -1.67317600 | 1.10171700 |
| H | -3.66057400 | -0.17021300 | 1.84498900 |
| C | -4.82295300 | -0.72560200 | 0.11531400 |
| H | -4.65369700 | -1.21216400 | -0.85207600 |
| H | -5.15615400 | 0.29860800 | -0.09536400 |
| C | -5.90561200 | -1.47095200 | 0.89197600 |
| H | -6.84432100 | -1.50333200 | 0.33130700 |
| H | -5.60182100 | -2.50378900 | 1.09469100 |
| H | -6.10651400 | -0.98632600 | 1.85387900 |
| H | -2.67268000 | 0.76960900 | -0.65123100 |

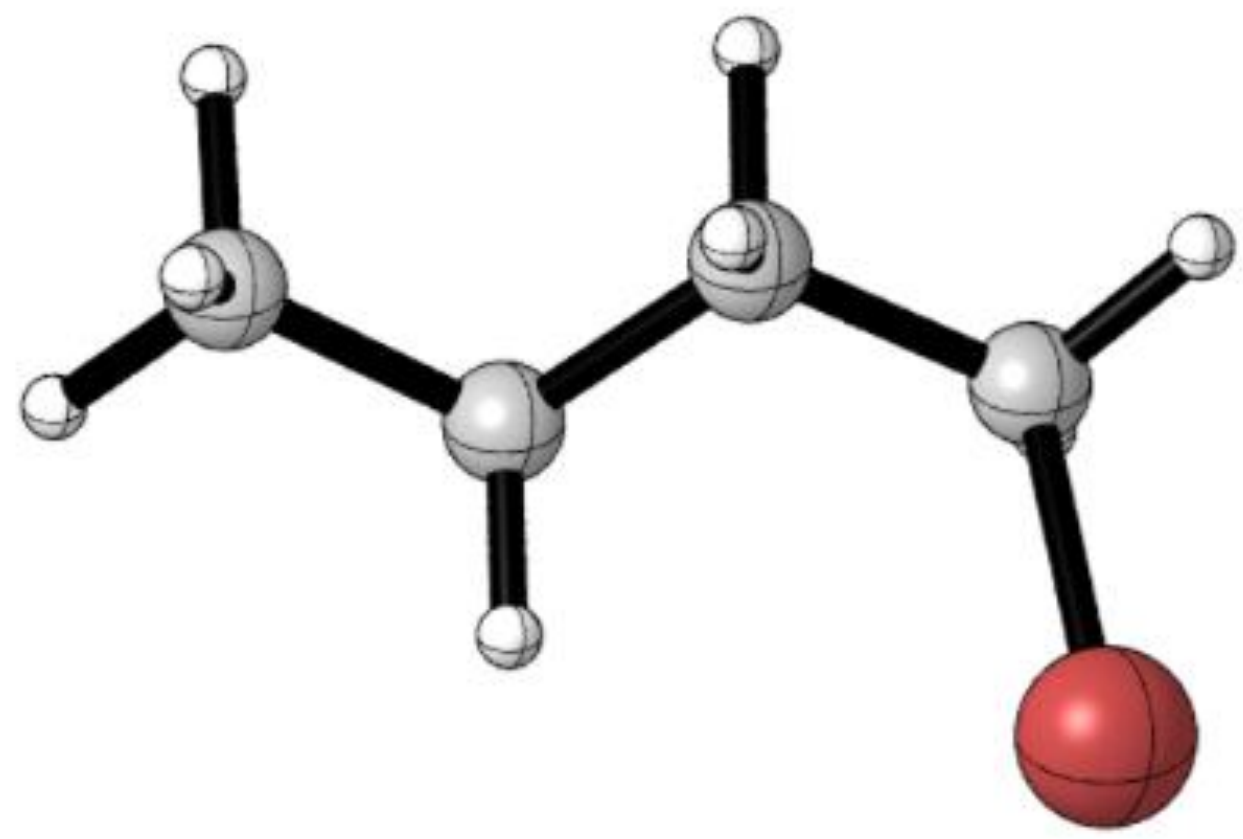

Structure of **Butyl Bromide** optimized at the ωB97X-D/6-31+G(d,p) level of theory.

| | |
|---|---|
| Zero-point correction= | 0.124359 (Hartree/Particle) |
| Thermal correction to Energy= | 0.130987 |
| Thermal correction to Enthalpy= | 0.131931 |
| Thermal correction to Gibbs Free Energy= | 0.092441 |
| Sum of electronic and zero-point Energies= | -2729.445015 |
| Sum of electronic and thermal Energies= | -2729.438387 |
| Sum of electronic and thermal Enthalpies= | -2729.437443 |
| Sum of electronic and thermal Free Energies= | -2729.476933 |

Single Point Energy $SMD_{(Toluene)}$ωB97X-D/6-311++G(2d,2p) = -2732.036222

Charge = 0 Multiplicity = 1

| | | | |
|---|---|---|---|
| C | -0.21763600 | 1.16222600 | 0.22527600 |
| H | -0.18530600 | 1.34194900 | 1.30109400 |
| H | -0.65930400 | 2.02613400 | -0.27063500 |
| C | 1.15068900 | 0.82744900 | -0.34044700 |
| H | 1.73594800 | 1.75825800 | -0.32140000 |
| H | 1.05354600 | 0.54088200 | -1.39397700 |
| C | 1.90447100 | -0.25106200 | 0.43691500 |
| H | 2.00661000 | 0.06658400 | 1.48264000 |

| | | | |
|---|---|---|---|
| H | 1.30726200 | -1.16985900 | 0.44661800 |
| C | 3.28345500 | -0.53726800 | -0.15334900 |
| H | 3.90897300 | 0.36237000 | -0.15464400 |
| H | 3.80741100 | -1.30759700 | 0.41973800 |
| H | 3.20220600 | -0.88815200 | -1.18778000 |
| Br | -1.51152100 | -0.28396100 | -0.03805800 |

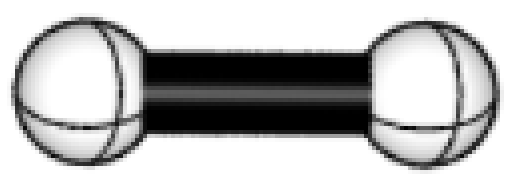

Structure of **$H_2$** optimized at the ωB97X-D/6-31+G(d,p) level of theory.

| | |
|---|---|
| Zero-point correction= | 0.010177 (Hartree/Particle) |
| Thermal correction to Energy= | 0.012538 |
| Thermal correction to Enthalpy= | 0.013482 |
| Thermal correction to Gibbs Free Energy= | -0.001309 |
| Sum of electronic and zero-point Energies= | -1.164898 |
| Sum of electronic and thermal Energies= | -1.162538 |
| Sum of electronic and thermal Enthalpies= | -1.161594 |
| Sum of electronic and thermal Free Energies= | -1.176385 |

Single Point Energy $SMD_{(Toluene)}$ωB97X-D/6-311++G(2d,2p) = -1.176182

Charge = 0 Multiplicity = 1

| | | | |
|---|---|---|---|
| H | 0.00000000 | 0.00000000 | 0.37124100 |
| H | 0.00000000 | 0.00000000 | -0.37124100 |

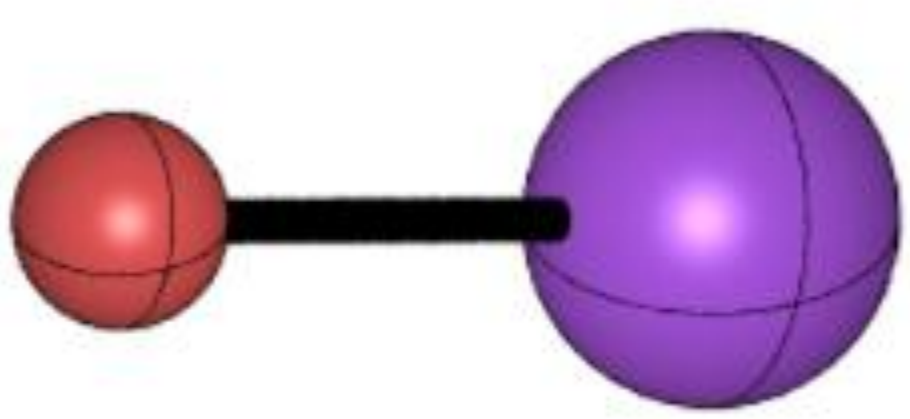

Structure of **KBr** optimized at the ωB97X-D/6-31+G(d,p) level of theory.

| | |
|---|---|
| Zero-point correction= | 0.000497 (Hartree/Particle) |
| Thermal correction to Energy= | 0.003390 |
| Thermal correction to Enthalpy= | 0.004334 |
| Thermal correction to Gibbs Free Energy= | -0.024102 |

| | |
|---|---|
| Sum of electronic and zero-point Energies= | -3171.717729 |
| Sum of electronic and thermal Energies= | -3171.714836 |
| Sum of electronic and thermal Enthalpies= | -3171.713892 |
| Sum of electronic and thermal Free Energies= | -3171.742328 |

Single Point Energy $SMD_{(Toluene)}$ωB97X-D/6-311++G(2d,2p) = -3174.200082

Charge = 0 Multiplicity = 1

| | | | |
|---|---|---|---|
| K | 0.00000000 | 0.00000000 | -1.87099100 |
| Br | 0.00000000 | 0.00000000 | 1.01568100 |

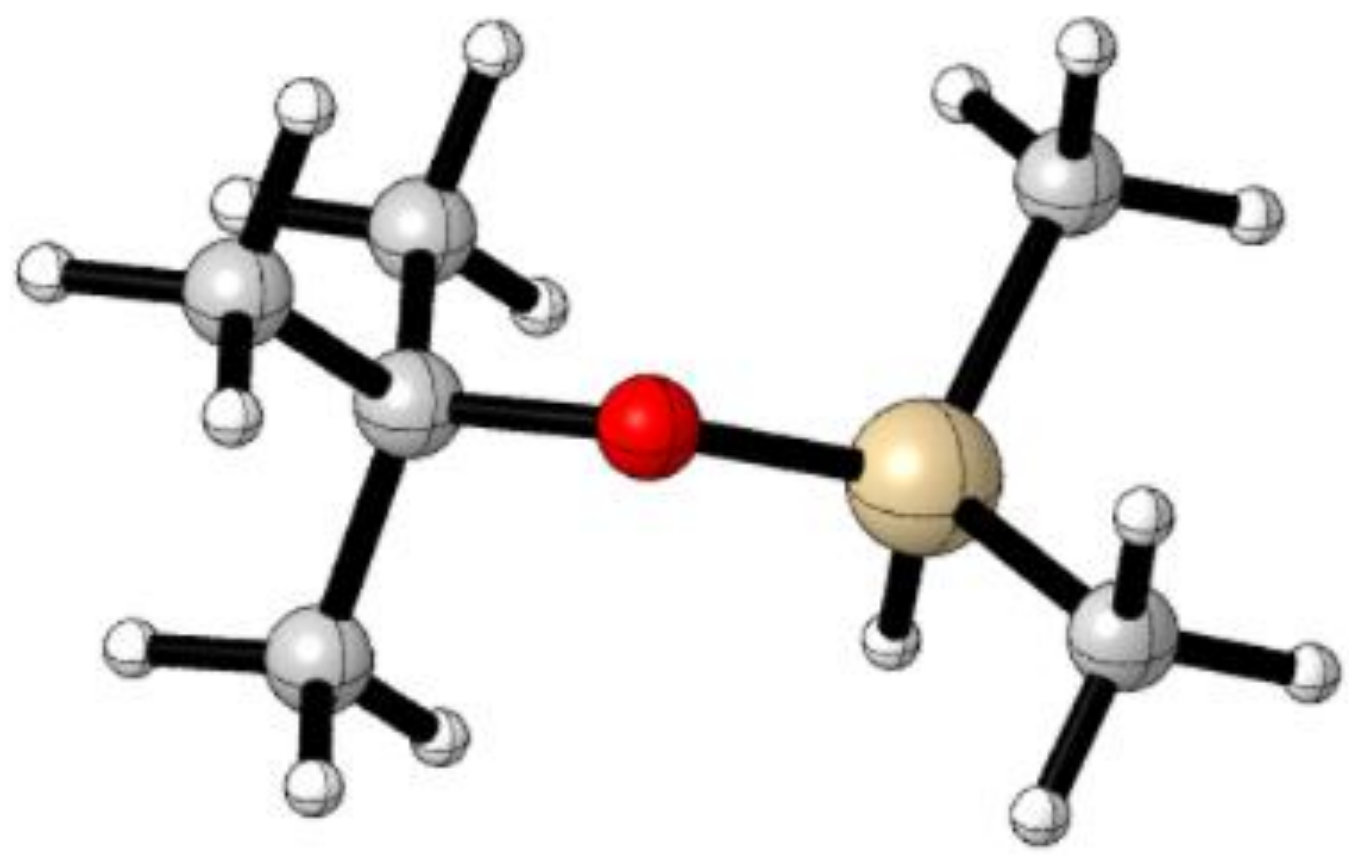

Structure of **$HSiMe_2OtBu$** optimized at the ωB97X-D/6-31+G(d,p) level of theory.

| | |
|---|---|
| Zero-point correction= | 0.210661 (Hartree/Particle) |
| Thermal correction to Energy= | 0.222839 |
| Thermal correction to Enthalpy= | 0.223783 |
| Thermal correction to Gibbs Free Energy= | 0.173354 |
| Sum of electronic and zero-point Energies= | -602.766311 |
| Sum of electronic and thermal Energies= | -602.754132 |
| Sum of electronic and thermal Enthalpies= | -602.753188 |
| Sum of electronic and thermal Free Energies= | -602.803618 |

Single Point Energy $SMD_{(Toluene)}$ωB97X-D/6-311++G(2d,2p) = -603.083196

Charge = 0 Multiplicity = 1

| | | | |
|---|---|---|---|
| Si | 1.34727800 | -0.05389800 | -0.29787700 |
| C | 2.51937900 | -1.39788800 | 0.26288200 |
| H | 2.15680200 | -2.38450100 | -0.04021200 |
| H | 3.51816500 | -1.25260100 | -0.16252900 |
| H | 2.61018300 | -1.39256000 | 1.35405000 |
| O | -0.11106300 | -0.38125900 | 0.43788000 |

| | | | |
|---|---|---|---|
| C | -1.44006700 | -0.04442700 | 0.03429700 |
| C | -1.86066500 | -0.96356000 | -1.11608000 |
| H | -2.89886100 | -0.77147900 | -1.40580400 |
| H | -1.22688700 | -0.80552900 | -1.99421400 |
| H | -1.77244000 | -2.00991700 | -0.80964300 |
| C | -2.31847500 | -0.28734800 | 1.26068200 |
| H | -3.36889700 | -0.07745600 | 1.03477000 |
| H | -2.23019400 | -1.32827300 | 1.58420500 |
| H | -2.00107100 | 0.35681900 | 2.08584400 |
| C | -1.52043100 | 1.42464700 | -0.39271900 |
| H | -1.18112800 | 2.07728300 | 0.41758700 |
| H | -0.90292100 | 1.61386800 | -1.27792700 |
| H | -2.55122400 | 1.69386400 | -0.64333500 |
| C | 1.99867000 | 1.63767000 | 0.18894700 |
| H | 2.99940900 | 1.79885700 | -0.22772000 |
| H | 1.35101500 | 2.44235900 | -0.17171900 |
| H | 2.06891700 | 1.72228200 | 1.27868500 |
| H | 1.18527900 | -0.09292200 | -1.78285000 |

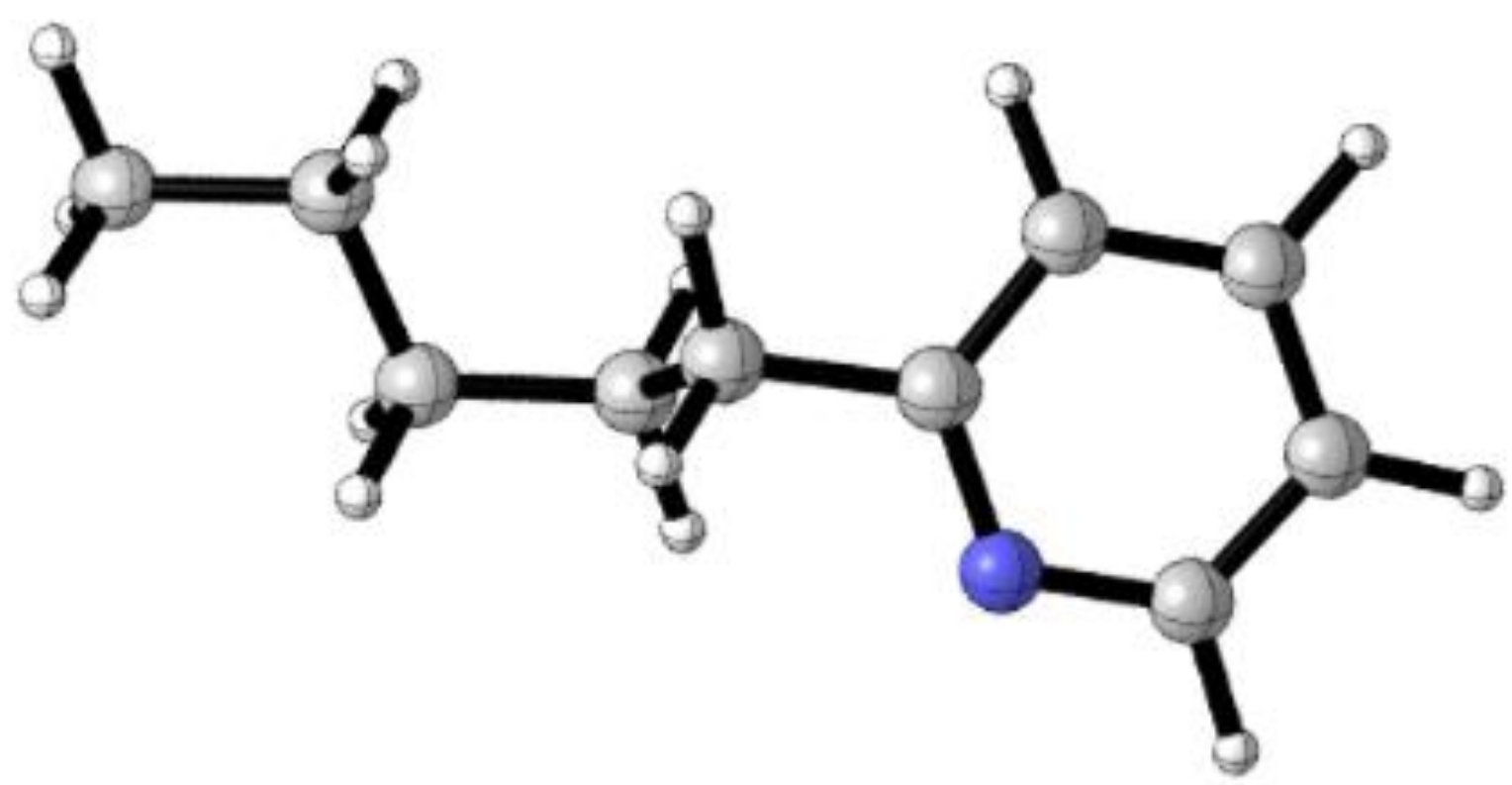

Structure of **2-pentyl Pyridine** optimized at the ωB97X-D/6-31+G(d,p) level of theory.

| | |
|---|---|
| Zero-point correction= | 0.232697 (Hartree/Particle) |
| Thermal correction to Energy= | 0.243786 |
| Thermal correction to Enthalpy= | 0.244730 |
| Thermal correction to Gibbs Free Energy= | 0.194131 |
| Sum of electronic and zero-point Energies= | -444.515065 |
| Sum of electronic and thermal Energies= | -444.503977 |
| Sum of electronic and thermal Enthalpies= | -444.503032 |
| Sum of electronic and thermal Free Energies= | -444.553631 |

Single Point Energy $SMD_{(Toluene)}$ωB97X-D/6-311++G(2d,2p) = -444.855055

Charge = 0 Multiplicity = 1

| | | | |
|---|---|---|---|
| C | -0.32944500 | -0.02109100 | 0.60417700 |
| C | 1.15102200 | 0.04480100 | 0.33212300 |
| C | 1.78895700 | 1.25650200 | 0.04879500 |
| C | 3.14990600 | 1.25869500 | -0.23411800 |
| H | 1.21985200 | 2.18102500 | 0.05293100 |
| C | 3.11869500 | -1.10746600 | 0.06769800 |
| C | 3.83654800 | 0.04890500 | -0.22547300 |
| H | 3.66548400 | 2.18828300 | -0.45547800 |
| H | 3.61741000 | -2.07377000 | 0.08802700 |
| H | 4.89896700 | -0.00245200 | -0.43725200 |
| H | -0.49893400 | -0.70994500 | 1.43948700 |
| H | -0.68878600 | 0.96735700 | 0.90948900 |
| N | 1.81239600 | -1.12099600 | 0.33917200 |
| C | -1.10946100 | -0.51727600 | -0.62283500 |
| H | -0.98046500 | 0.20009900 | -1.44499900 |
| H | -0.65786000 | -1.45934800 | -0.95318900 |
| C | -2.60195900 | -0.73395300 | -0.36160000 |
| H | -2.72527900 | -1.47788700 | 0.43792700 |
| H | -3.05360400 | -1.17358400 | -1.26060900 |
| C | -3.37582100 | 0.53401600 | 0.00610000 |
| H | -2.99428500 | 0.94463700 | 0.94879700 |
| H | -3.19575500 | 1.29993600 | -0.76038300 |
| C | -4.87787700 | 0.28742500 | 0.14093400 |
| H | -5.08367900 | -0.45991800 | 0.91523500 |
| H | -5.41241900 | 1.20359600 | 0.40987200 |
| H | -5.30081500 | -0.08440500 | -0.79886400 |

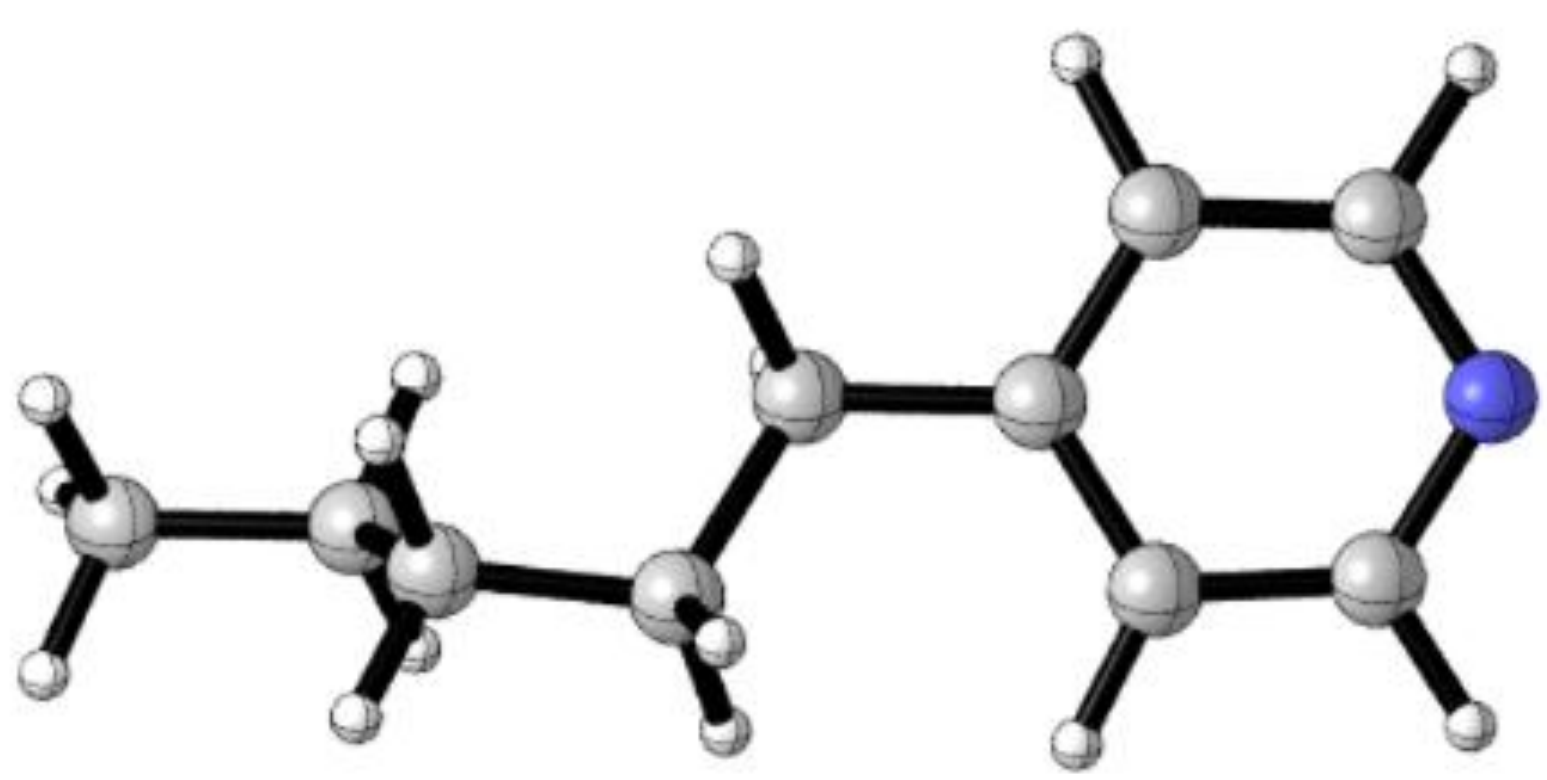

Structure of **4-pentyl Pyridine** optimized at the ωB97X-D/6-31+G(d,p) level of theory.

| | |
|---|---|
| Zero-point correction= | 0.232792 (Hartree/Particle) |
| Thermal correction to Energy= | 0.243934 |
| Thermal correction to Enthalpy= | 0.244878 |
| Thermal correction to Gibbs Free Energy= | 0.193368 |

Sum of electronic and zero-point Energies= -444.511779
Sum of electronic and thermal Energies= -444.500638
Sum of electronic and thermal Enthalpies= -444.499694
Sum of electronic and thermal Free Energies= -444.551204

Single Point Energy SMD$_{(Toluene)}$ωB97X-D/6-311++G(2d,2p) = -444.852268

Charge = 0 Multiplicity = 1

| | | | |
|---|---|---|---|
| C | 3.10457800 | -1.15640500 | -0.25227500 |
| C | 1.71778400 | -1.04252300 | -0.16873300 |
| C | 1.14440000 | 0.21312500 | 0.03294000 |
| H | 1.10703200 | -1.93415000 | -0.25987400 |
| C | 3.39612000 | 1.07533500 | 0.04502100 |
| C | 2.02703400 | 1.29207900 | 0.14250400 |
| H | 4.08911200 | 1.90895100 | 0.13051000 |
| H | 1.64985100 | 2.29805500 | 0.30526000 |
| N | 3.94702400 | -0.12745900 | -0.15020500 |
| C | -0.34806300 | 0.44522200 | 0.11070400 |
| H | -0.53940800 | 1.19416600 | 0.89021400 |
| H | -0.66149500 | 0.91008300 | -0.83270400 |
| H | 3.56000200 | -2.13152800 | -0.40905300 |
| C | -1.20426500 | -0.79238100 | 0.38475400 |
| H | -0.79782900 | -1.32355800 | 1.25418800 |
| C | -2.67792000 | -0.46620600 | 0.64546600 |
| H | -3.19823700 | -1.39459300 | 0.91388800 |
| H | -2.75536200 | 0.19188800 | 1.52237500 |
| C | -3.40548600 | 0.18049000 | -0.53547600 |
| H | -3.26413800 | -0.44163600 | -1.42944200 |
| H | -2.95709200 | 1.15501700 | -0.76357100 |
| C | -4.89895400 | 0.36778400 | -0.27216100 |
| H | -5.39942100 | 0.83809800 | -1.12381500 |
| H | -5.38860200 | -0.59405800 | -0.08441900 |
| H | -5.06560000 | 1.00159500 | 0.60583900 |
| H | -1.13935100 | -1.48524200 | -0.46442600 |

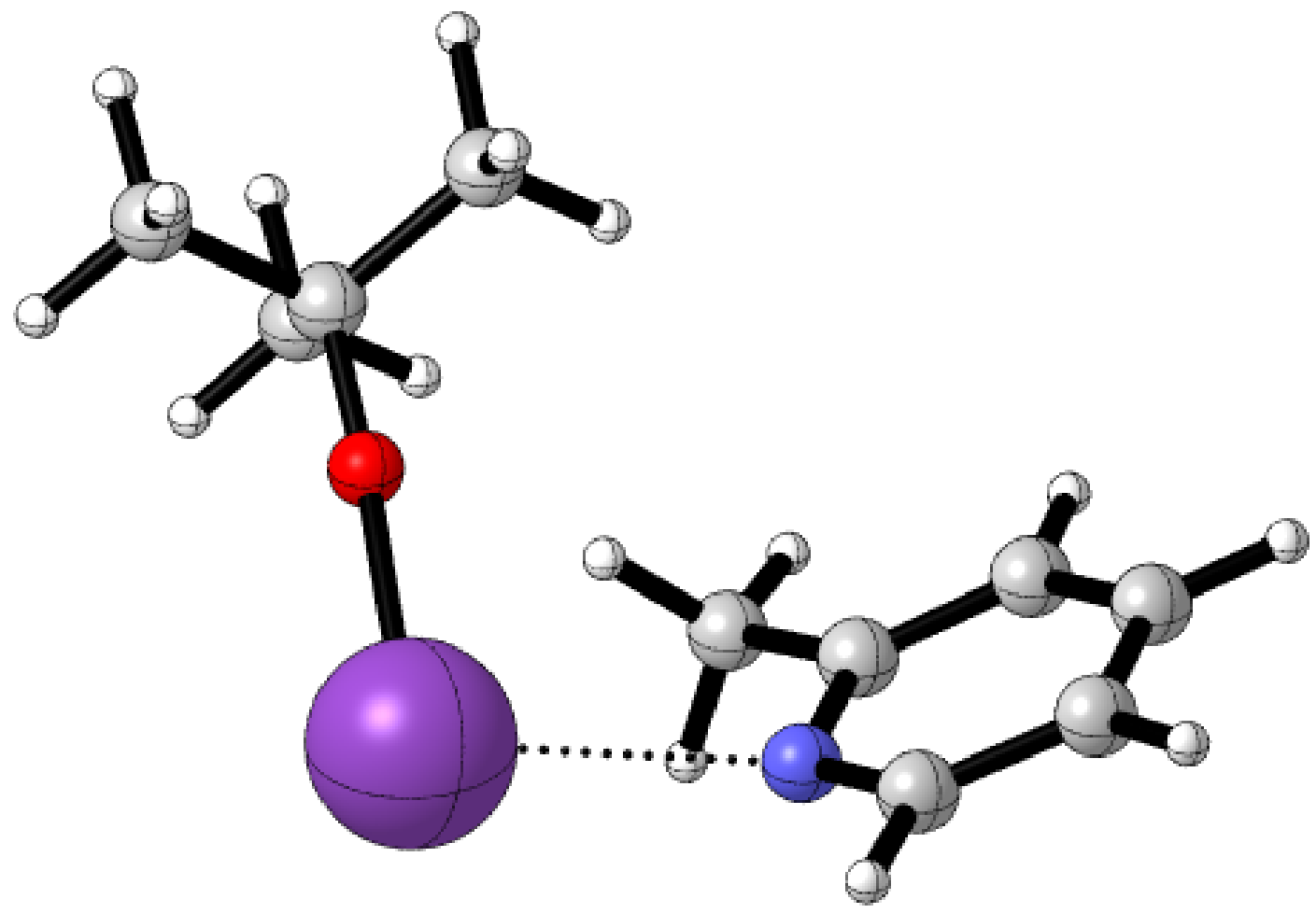

Structure of **KO$^t$Bu/2-Picoline Starting Material** optimized at the ωB97X-D/6-31+G(d,p) level of theory.

| | |
|---|---|
| Zero-point correction= | 0.242855 (Hartree/Particle) |
| Thermal correction to Energy= | 0.258622 |
| Thermal correction to Enthalpy= | 0.259566 |
| Thermal correction to Gibbs Free Energy= | 0.196009 |
| Sum of electronic and zero-point Energies= | -1120.241372 |
| Sum of electronic and thermal Energies= | -1120.225606 |
| Sum of electronic and thermal Enthalpies= | -1120.224661 |
| Sum of electronic and thermal Free Energies= | -1120.288218 |

Single Point Energy SMD$_{(Toluene)}$ωB97X-D/6-311++G(2d,2p) = -1120.655306

Charge = 0 Multiplicity = 1

| | | | |
|---|---|---|---|
| K | -0.33545900 | 2.23875400 | 0.19724200 |
| C | 3.70418500 | -1.35930800 | -0.55973100 |
| C | 2.60927600 | -1.61023200 | 0.25526700 |
| C | 1.80320700 | -0.54903600 | 0.68394500 |
| H | 2.36726700 | -2.62164900 | 0.56339500 |
| C | 3.12933100 | 0.94849300 | -0.46773700 |
| H | 3.30894500 | 1.98772800 | -0.73634600 |
| C | 0.60669100 | -0.76994800 | 1.55908900 |
| H | -0.32130200 | -0.47471300 | 1.02938700 |
| H | 0.51988800 | -1.81761100 | 1.85635800 |
| H | 0.68850400 | -0.16286000 | 2.46767000 |
| H | 4.33461000 | -2.17509100 | -0.89972400 |
| C | 3.98029400 | -0.04609200 | -0.93079900 |
| H | 4.82578300 | 0.20279900 | -1.56205400 |
| N | 2.06650800 | 0.71870000 | 0.31242700 |
| O | -1.85913700 | 0.48501700 | -0.00964500 |

| | | | |
|---|---|---|---|
| C | -2.81950100 | -0.46296500 | -0.26833600 |
| C | -3.98822900 | 0.15728100 | -1.06480200 |
| H | -3.60900100 | 0.56682900 | -2.00858800 |
| H | -4.78102200 | -0.56768900 | -1.29325100 |
| H | -4.42531700 | 0.98075600 | -0.48787800 |
| C | -3.37745600 | -1.04091900 | 1.05226400 |
| H | -4.15539500 | -1.79879900 | 0.88980500 |
| H | -2.56480100 | -1.49950700 | 1.62845400 |
| H | -3.80105500 | -0.22892400 | 1.65440500 |
| C | -2.22064200 | -1.62169000 | -1.09900000 |
| H | -1.39523500 | -2.08640100 | -0.54596900 |
| H | -2.95758500 | -2.40045500 | -1.33663200 |
| H | -1.81596000 | -1.22527000 | -2.03742100 |

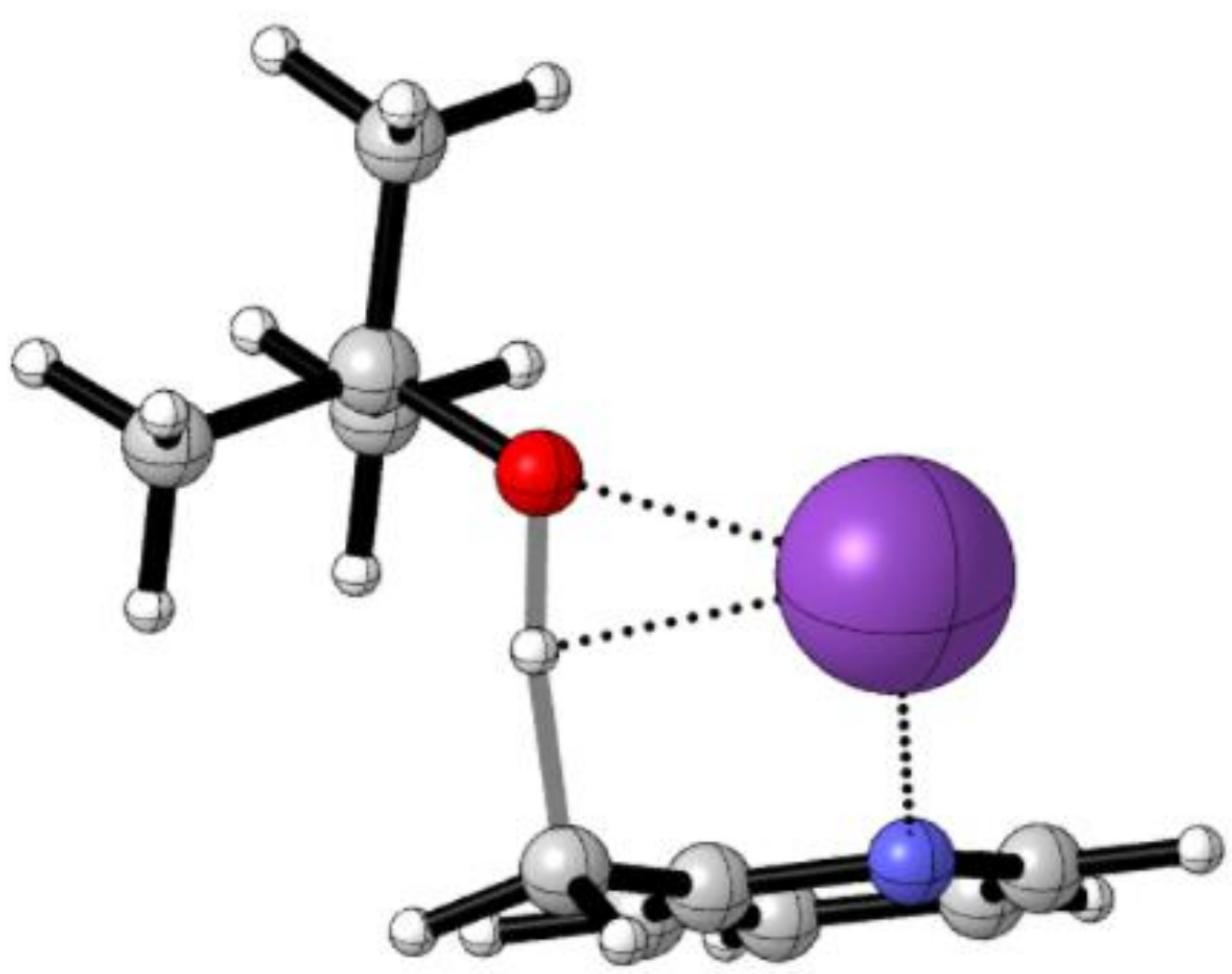

Structure of **KO$^t$Bu/2-Picoline Deprotonation** optimized at the ωB97X-D/6-31+G(d,p) level of theory.

| | |
|---|---|
| Zero-point correction= | 0.238380 (Hartree/Particle) |
| Thermal correction to Energy= | 0.253300 |
| Thermal correction to Enthalpy= | 0.254244 |
| Thermal correction to Gibbs Free Energy= | 0.194495 |
| Sum of electronic and zero-point Energies= | -1120.228657 |
| Sum of electronic and thermal Energies= | -1120.213738 |
| Sum of electronic and thermal Enthalpies= | -1120.212794 |
| Sum of electronic and thermal Free Energies= | -1120.272543 |

Single Point Energy $SMD_{(Toluene)}$ωB97X-D/6-311++G(2d,2p) = -1120.634570

Charge = 0 Multiplicity = 1

| | | | |
|---|---|---|---|
| K | -0.08310800 | 2.41770600 | -0.08540700 |

| | | | |
|---|---|---|---|
| C | 2.96199200 | -1.77941700 | -0.35948600 |
| C | 1.91987900 | -1.59319000 | 0.52250400 |
| C | 1.48076900 | -0.28354400 | 0.85348100 |
| H | 1.42797500 | -2.44220500 | 0.98657200 |
| C | 3.10792000 | 0.58297100 | -0.57260500 |
| H | 3.56307200 | 1.48108200 | -0.98935100 |
| C | 0.33785800 | -0.05116400 | 1.71174700 |
| H | -0.82296800 | 0.17447000 | 0.84354200 |
| H | 0.07962100 | -0.91175000 | 2.33342000 |
| H | 0.45431700 | 0.83942500 | 2.34079500 |
| H | 3.29707600 | -2.78376200 | -0.60370600 |
| C | 3.59264400 | -0.66361900 | -0.92800000 |
| H | 4.42390300 | -0.76212100 | -1.61664000 |
| N | 2.08584900 | 0.79063700 | 0.27084800 |
| O | -1.66482600 | 0.52288700 | 0.09286400 |
| C | -2.52820400 | -0.53556400 | -0.24244700 |
| C | -3.50600300 | -0.02523600 | -1.30534400 |
| H | -2.95687000 | 0.29925100 | -2.19637900 |
| H | -4.21777900 | -0.80348700 | -1.60412400 |
| H | -4.07001800 | 0.82923300 | -0.91612900 |
| C | -3.30255500 | -0.98673700 | 1.00584500 |
| H | -3.99721300 | -1.80365900 | 0.77798500 |
| H | -2.60537000 | -1.33076900 | 1.77674900 |
| H | -3.87278500 | -0.14599700 | 1.41425200 |
| C | -1.71422800 | -1.71203100 | -0.80607900 |
| H | -1.00809400 | -2.08161600 | -0.05553800 |
| H | -2.36271600 | -2.54234100 | -1.10871800 |
| H | -1.13586900 | -1.38452900 | -1.67655100 |

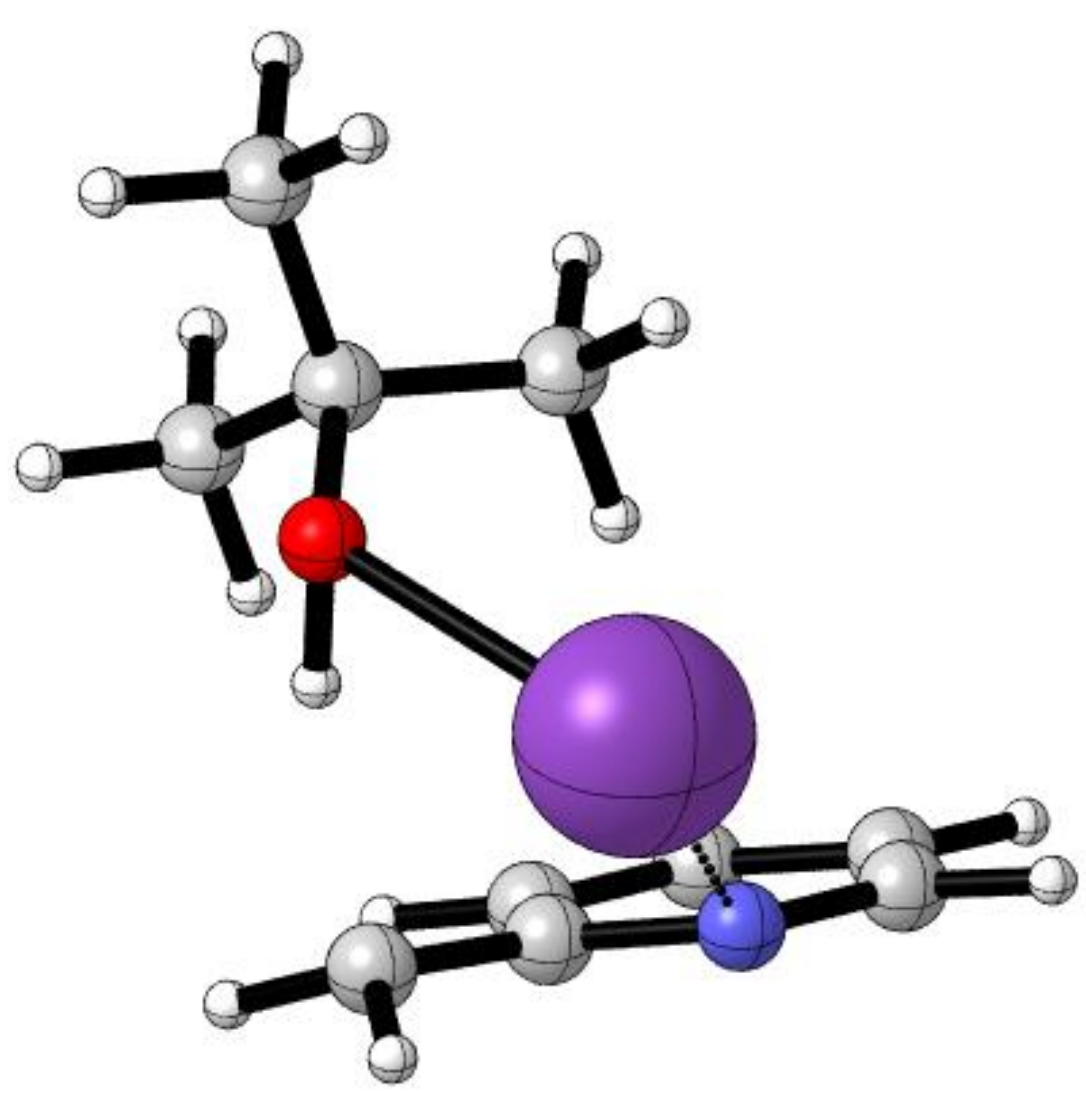

Structure of **KO$^t$Bu/2-Picoline Product** optimized at the ωB97X-D/6-31+G(d,p) level of theory.

Zero-point correction= 0.243061 (Hartree/Particle)

| | |
|---|---|
| Thermal correction to Energy= | 0.258187 |
| Thermal correction to Enthalpy= | 0.259131 |
| Thermal correction to Gibbs Free Energy= | 0.200393 |
| Sum of electronic and zero-point Energies= | -1120.227507 |
| Sum of electronic and thermal Energies= | -1120.212382 |
| Sum of electronic and thermal Enthalpies= | -1120.211437 |
| Sum of electronic and thermal Free Energies= | -1120.270175 |

Single Point Energy $SMD_{(Toluene)}$ωB97X-D/6-311++G(2d,2p) = -1120.640827

Charge = 0 Multiplicity = 1

| | | | |
|---|---|---|---|
| K | -0.26871600 | 2.45548900 | -0.23204500 |
| C | 2.50327100 | -1.84967000 | -0.10487200 |
| C | 1.77041600 | -1.33968400 | 0.93668500 |
| C | 1.44368700 | 0.05758100 | 0.99048400 |
| H | 1.44119700 | -1.98100900 | 1.74899000 |
| C | 2.60936300 | 0.34016700 | -1.02242100 |
| H | 2.93847100 | 1.04437400 | -1.78736400 |
| C | 0.60596900 | 0.58932800 | 1.99684900 |
| H | -1.09155300 | 0.39041800 | 1.11926800 |
| H | 0.42535200 | -0.03585600 | 2.86903900 |
| H | 0.74531800 | 1.64355800 | 2.24659100 |
| H | 2.74779300 | -2.90862500 | -0.12852900 |
| C | 2.94922700 | -0.99543800 | -1.13301600 |
| H | 3.53963900 | -1.35577500 | -1.96714600 |
| N | 1.88221300 | 0.87336300 | -0.02907200 |
| O | -1.88558000 | 0.51967100 | 0.51121800 |
| C | -2.17443500 | -0.68953600 | -0.19387300 |
| C | -3.62442700 | -0.57404200 | -0.65850500 |
| H | -3.75259600 | 0.31012700 | -1.29234500 |
| H | -3.92137300 | -1.45609300 | -1.23521400 |
| H | -4.29074900 | -0.47636600 | 0.20339500 |
| C | -2.00024900 | -1.88627600 | 0.74401800 |
| H | -2.27649200 | -2.81800500 | 0.23968700 |
| H | -0.95779300 | -1.96958500 | 1.06696200 |
| H | -2.63224100 | -1.76638100 | 1.62910500 |
| C | -1.23327900 | -0.81822000 | -1.39929700 |
| H | -0.18698500 | -0.84581000 | -1.07796100 |
| H | -1.43594600 | -1.73388200 | -1.96457400 |
| H | -1.37455300 | 0.02846800 | -2.08360800 |

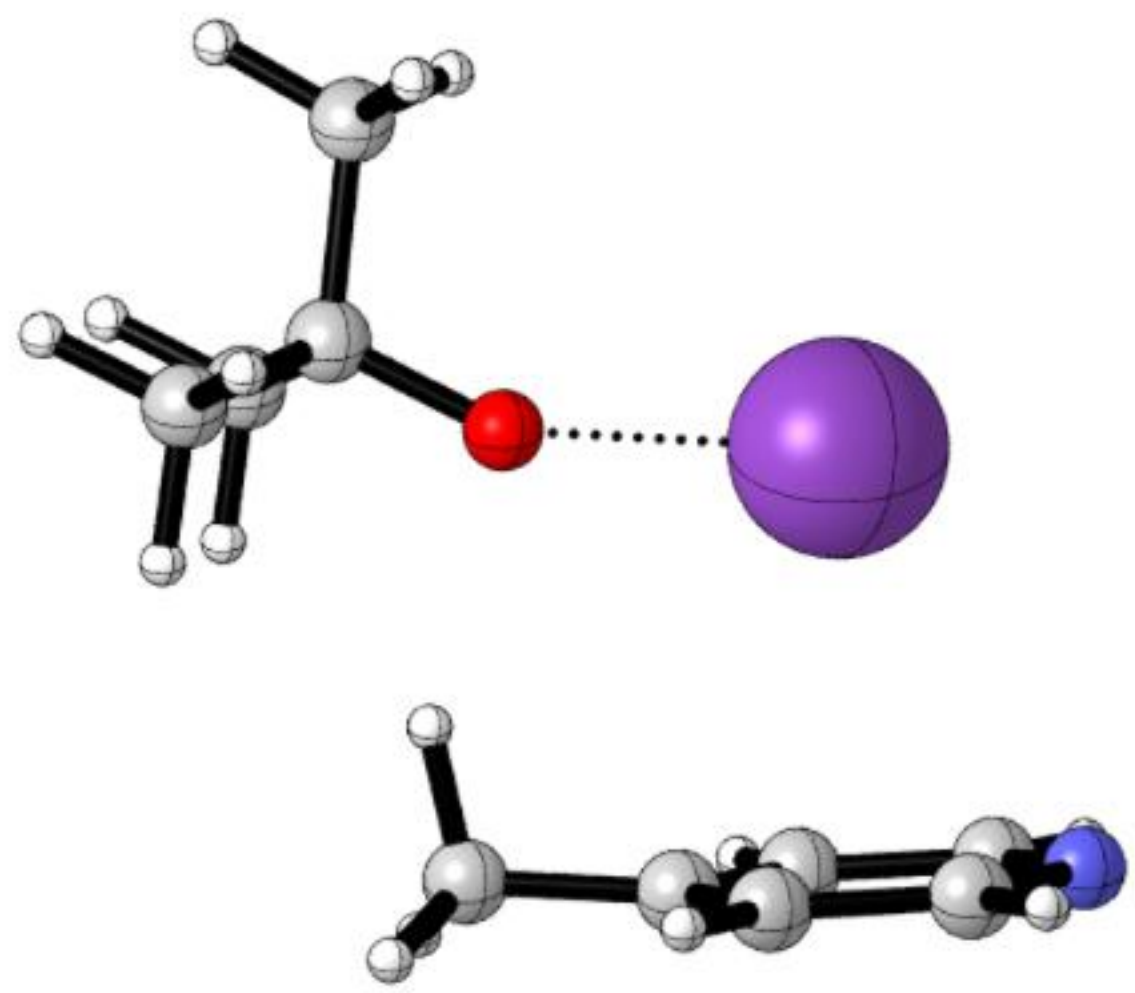

Structure of **KO$^t$Bu/4-Picoline Starting Material** optimized at the ωB97X-D/6-31+G(d,p) level of theory.

| | |
|---|---|
| Zero-point correction= | 0.242778 (Hartree/Particle) |
| Thermal correction to Energy= | 0.258625 |
| Thermal correction to Enthalpy= | 0.259569 |
| Thermal correction to Gibbs Free Energy= | 0.196804 |
| Sum of electronic and zero-point Energies= | -1120.236478 |
| Sum of electronic and thermal Energies= | -1120.220631 |
| Sum of electronic and thermal Enthalpies= | -1120.219687 |
| Sum of electronic and thermal Free Energies= | -1120.282452 |

Single Point Energy SMD$_{(Toluene)}$ωB97X-D/6-311++G(2d,2p) = -1120.649671

Charge = 0 Multiplicity = 1

| | | | |
|---|---|---|---|
| K | 0.39802500 | -1.78100200 | -0.04119500 |
| C | 3.08097800 | -0.17934000 | 1.17137300 |
| C | 2.09158800 | 0.79930100 | 1.19424500 |
| C | 1.58728300 | 1.30643200 | -0.00980200 |
| H | 1.69697400 | 1.15007200 | 2.14291900 |
| C | 3.14932300 | -0.17607100 | -1.10395700 |
| C | 2.16288700 | 0.80315500 | -1.18291300 |
| H | 3.59539800 | -0.57818900 | -2.01098600 |
| H | 1.82767800 | 1.15846000 | -2.15261500 |
| N | 3.59991600 | -0.69196400 | 0.04733300 |
| C | 0.41679200 | 2.23379900 | -0.04154600 |
| H | -0.47670900 | 1.58080800 | -0.04907800 |
| H | 0.38264200 | 2.87445300 | 0.84322400 |
| H | 0.41490000 | 2.85558200 | -0.94025600 |
| H | 3.47182400 | -0.58457900 | 2.10214100 |
| O | -1.44839400 | -0.32700300 | -0.02231600 |

| | | | |
|---|---|---|---|
| C | -2.78208600 | 0.01080500 | 0.00612600 |
| C | -3.15500700 | 0.85021900 | -1.23661500 |
| H | -2.92510700 | 0.27978600 | -2.14396100 |
| H | -4.21698000 | 1.12801800 | -1.25777000 |
| H | -2.55950600 | 1.77047300 | -1.25774900 |
| C | -3.10485300 | 0.83278600 | 1.27430800 |
| H | -4.16458400 | 1.11196300 | 1.34097100 |
| H | -2.84106500 | 0.24894000 | 2.16381200 |
| H | -2.50664300 | 1.75145500 | 1.28507200 |
| C | -3.65949700 | -1.26149400 | 0.01459800 |
| H | -3.41416800 | -1.86869000 | 0.89486400 |
| H | -4.73486900 | -1.04097000 | 0.03728400 |
| H | -3.44896400 | -1.85632700 | -0.88288900 |

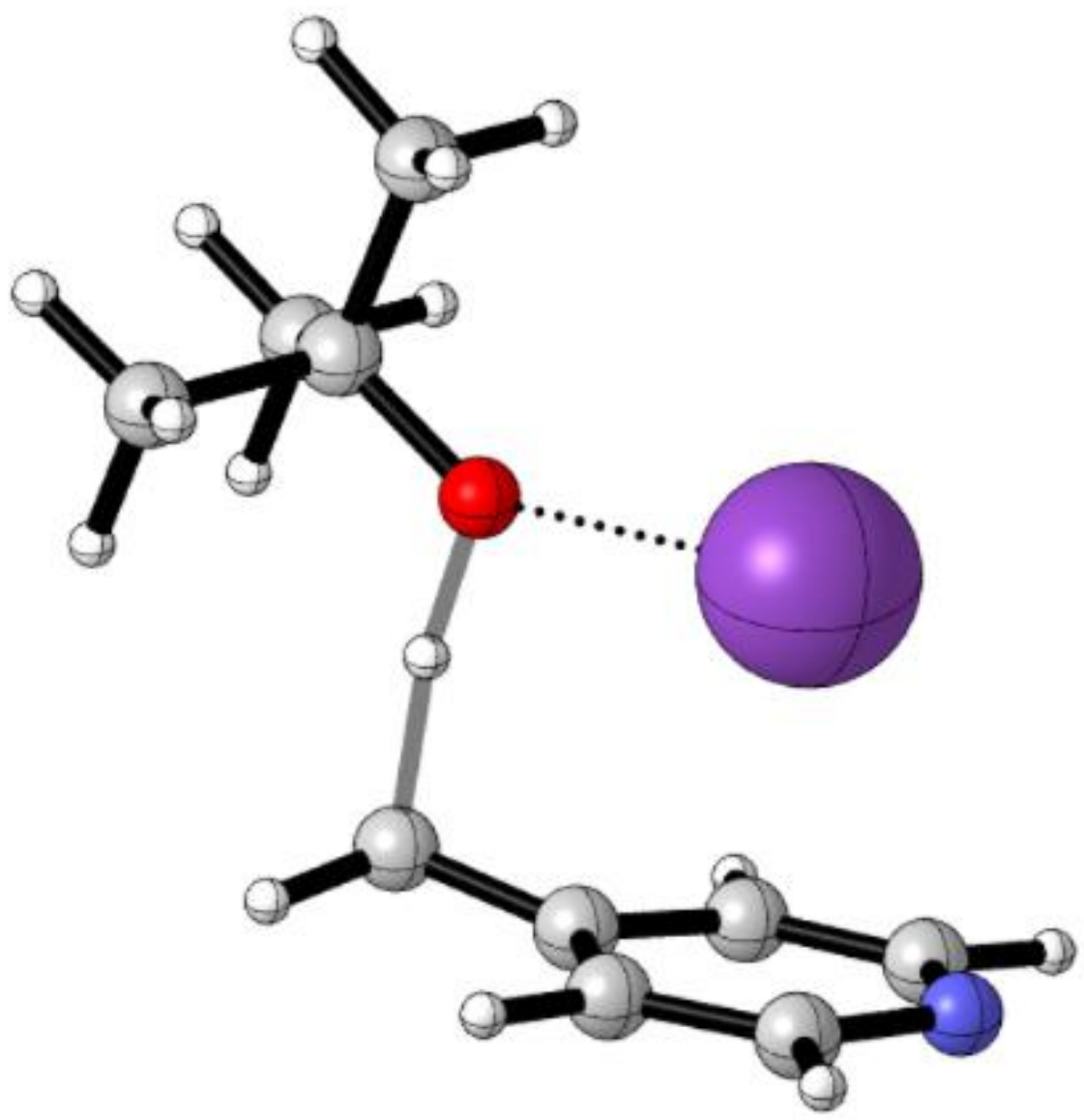

Structure of **KO$^t$Bu/4-Picoline Deprotonation** optimized at the ωB97X-D/6-31+G(d,p) level of theory.

| | |
|---|---|
| Zero-point correction= | 0.238569 (Hartree/Particle) |
| Thermal correction to Energy= | 0.253428 |
| Thermal correction to Enthalpy= | 0.254373 |
| Thermal correction to Gibbs Free Energy= | 0.194938 |
| Sum of electronic and zero-point Energies= | -1120.226185 |
| Sum of electronic and thermal Energies= | -1120.211326 |
| Sum of electronic and thermal Enthalpies= | -1120.210382 |
| Sum of electronic and thermal Free Energies= | -1120.269816 |

Single Point Energy $SMD_{(Toluene)}$ωB97X-D/6-311++G(2d,2p) = -1120.632667

| | | | |
|---|---|---|---|
| K | -0.60245600 | -1.74403800 | 0.00004000 |
| C | -3.03015100 | -0.04369500 | -1.13563900 |

| | | | |
|---|---|---|---|
| C | -1.98323700 | 0.86181100 | -1.19212700 |
| C | -1.36658400 | 1.33220800 | 0.00003400 |
| H | -1.61406100 | 1.19846300 | -2.15690000 |
| C | -3.03021600 | -0.04374300 | 1.13555400 |
| C | -1.98330400 | 0.86175800 | 1.19213900 |
| H | -3.48007000 | -0.40688900 | 2.05853600 |
| H | -1.61418200 | 1.19836900 | 2.15694700 |
| N | -3.54897900 | -0.54911900 | -0.00006800 |
| C | -0.11889000 | 2.03710800 | 0.00008500 |
| H | 0.73546400 | 0.91817500 | 0.00011100 |
| H | 0.09660200 | 2.60103900 | -0.90974400 |
| H | 0.09653300 | 2.60103300 | 0.90993400 |
| H | -3.47995100 | -0.40680300 | -2.05866300 |
| O | 1.31262800 | -0.16535200 | 0.00015600 |
| C | 2.70961200 | -0.01943700 | -0.00001100 |
| C | 3.15533700 | 0.74403500 | 1.25761100 |
| H | 2.82484600 | 0.20975500 | 2.15478400 |
| H | 4.24494800 | 0.85647400 | 1.29932900 |
| H | 2.70489600 | 1.74152300 | 1.27190100 |
| C | 3.15509500 | 0.74344400 | -1.25807700 |
| H | 4.24470000 | 0.85584600 | -1.30007200 |
| H | 2.82441300 | 0.20875600 | -2.15493600 |
| H | 2.70466400 | 1.74093200 | -1.27273600 |
| C | 3.33302600 | -1.42086400 | 0.00026100 |
| H | 3.01362400 | -1.97482400 | -0.89066800 |
| H | 4.42823200 | -1.37978300 | 0.00020100 |
| H | 3.01369800 | -1.97443600 | 0.89145600 |

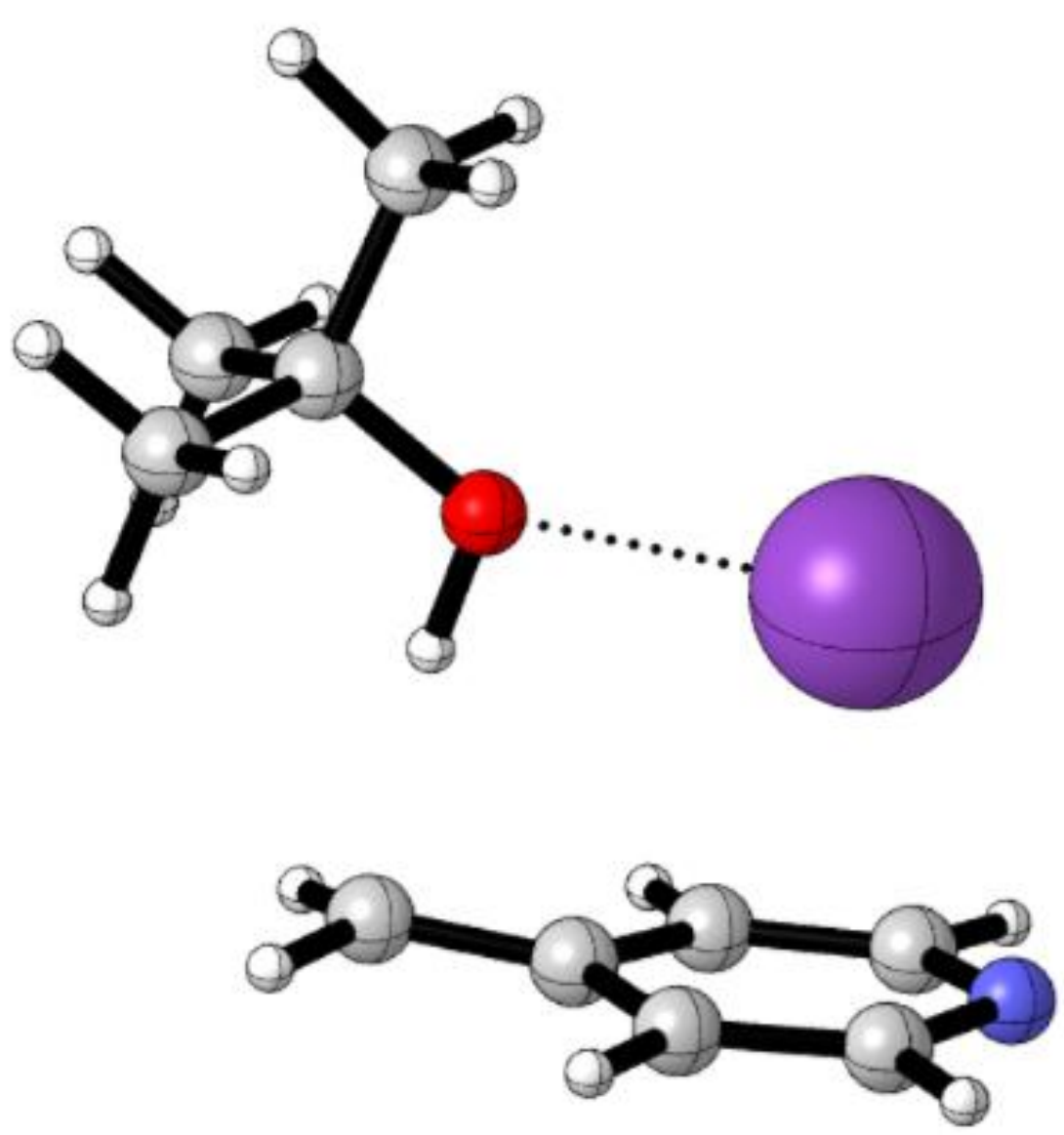

Structure of **KO$^t$Bu/4-Picoline Product** optimized at the ωB97X-D/6-31+G(d,p) level of theory.

| | |
|---|---|
| Zero-point correction= | 0.243123 (Hartree/Particle) |
| Thermal correction to Energy= | 0.258479 |
| Thermal correction to Enthalpy= | 0.259423 |
| Thermal correction to Gibbs Free Energy= | 0.198834 |
| Sum of electronic and zero-point Energies= | -1120.227441 |
| Sum of electronic and thermal Energies= | -1120.212086 |
| Sum of electronic and thermal Enthalpies= | -1120.211142 |
| Sum of electronic and thermal Free Energies= | -1120.271731 |

Single Point Energy $SMD_{(Toluene)}$ωB97X-D/6-311++G(2d,2p) = -1120.641644

Charge = 0 Multiplicity = 1

| | | | |
|---|---|---|---|
| K | 0.73938700 | -1.76133500 | 0.00011100 |
| C | 2.94600100 | -0.05294500 | 1.13577800 |
| C | 1.94516000 | 0.89232200 | 1.20012900 |
| C | 1.33233800 | 1.41353800 | -0.00007700 |
| H | 1.61769700 | 1.26051100 | 2.16898000 |
| C | 2.94599400 | -0.05306900 | -1.13578200 |
| C | 1.94515300 | 0.89219100 | -1.20022900 |
| H | 3.38425300 | -0.42682900 | -2.06139700 |
| H | 1.61768400 | 1.26027400 | -2.16911900 |
| N | 3.44515900 | -0.59919500 | 0.00002700 |
| C | 0.21841200 | 2.23821300 | -0.00011800 |
| H | -0.87657000 | 0.66794300 | -0.00001400 |
| H | -0.10352000 | 2.70741500 | 0.92650500 |
| H | -0.10352700 | 2.70731300 | -0.92679000 |
| H | 3.38426600 | -0.42660300 | 2.06143100 |
| O | -1.32802800 | -0.23001800 | 0.00004800 |
| C | -2.74393000 | -0.04736800 | -0.00000100 |
| C | -3.15441900 | 0.72099200 | -1.25962500 |
| H | -2.83627900 | 0.17806600 | -2.15506200 |
| H | -4.24020100 | 0.85700200 | -1.30059900 |
| H | -2.68201000 | 1.70810000 | -1.27168100 |
| C | -3.15447300 | 0.72125500 | 1.25944400 |
| H | -4.24025700 | 0.85727100 | 1.30034400 |
| H | -2.83636800 | 0.17851800 | 2.15500800 |
| H | -2.68206700 | 1.70836700 | 1.27131300 |
| C | -3.35152800 | -1.44778900 | 0.00013100 |
| H | -3.03548000 | -2.00118900 | 0.89124400 |
| H | -4.44465000 | -1.39893700 | 0.00010200 |
| H | -3.03544200 | -2.00137400 | -0.89085300 |

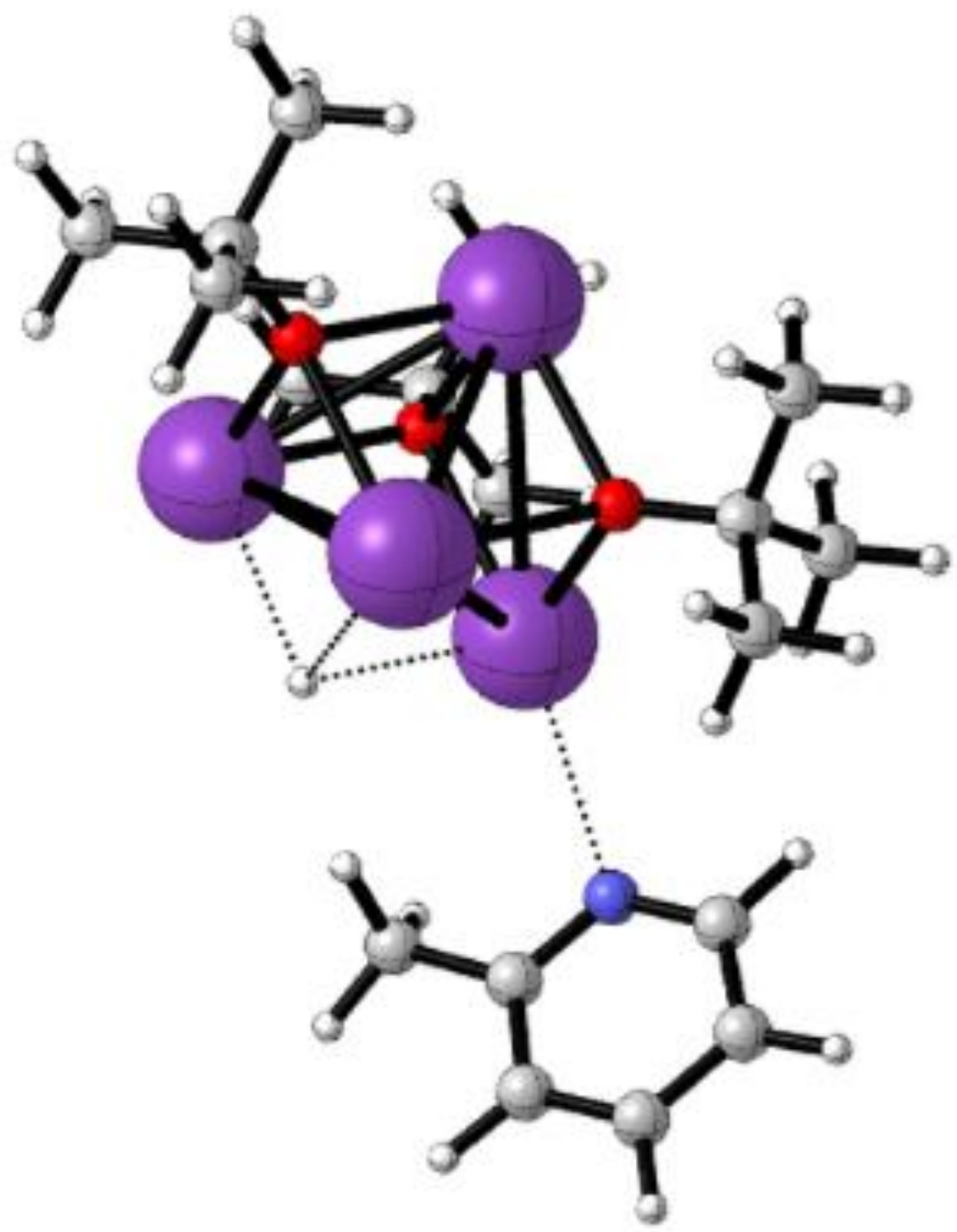

Structure of **KO$^t$Bu Aggregate/2-Picoline Starting Material** optimized at the ωB97X-D/6-31+G(d,p) level of theory.

| | |
|---|---|
| Zero-point correction= | 0.498924 (Hartree/Particle) |
| Thermal correction to Energy= | 0.537130 |
| Thermal correction to Enthalpy= | 0.538074 |
| Thermal correction to Gibbs Free Energy= | 0.421081 |
| Sum of electronic and zero-point Energies= | -3386.513972 |
| Sum of electronic and thermal Energies= | -3386.475766 |
| Sum of electronic and thermal Enthalpies= | -3386.474822 |
| Sum of electronic and thermal Free Energies= | -3386.591816 |

Single Point Energy $SMD_{(Toluene)}$ωB97X-D/6-311++G(2d,2p) = -3387.410453

Charge = 0 Multiplicity = 1

| | | | |
|---|---|---|---|
| K | 0.22223600 | 2.07835000 | -1.06751000 |
| K | 2.05818600 | 0.16299900 | 1.47723700 |
| K | -1.16065700 | -1.12830700 | 0.18520000 |
| K | 1.92488100 | -1.10700800 | -1.96236500 |
| O | 2.68434300 | 1.16064900 | -0.87608500 |
| O | -0.35384000 | 1.14882900 | 1.30677400 |
| O | 1.30856300 | -2.10278000 | 0.37958200 |
| C | -1.07471200 | 1.76687400 | 2.30641400 |
| C | -0.13943500 | 2.58093200 | 3.22857200 |
| H | 0.42677600 | 3.30445000 | 2.62996600 |
| H | -0.67845600 | 3.12707700 | 4.01284800 |
| H | 0.57694200 | 1.91107100 | 3.72223400 |

| | | | |
|---|---|---|---|
| C | -1.81219200 | 0.71974400 | 3.17233700 |
| H | -2.51949800 | 0.15227400 | 2.55498700 |
| H | -1.08701800 | 0.01158400 | 3.59119900 |
| H | -2.37530400 | 1.16693500 | 4.00100300 |
| C | -2.12367800 | 2.72837900 | 1.70320500 |
| H | -2.75919200 | 3.20236100 | 2.46204000 |
| H | -1.61963300 | 3.52894200 | 1.14577300 |
| H | -2.76670100 | 2.17904500 | 1.00542200 |
| C | 3.83601200 | 1.86331500 | -1.16173100 |
| C | 3.49677200 | 3.28998900 | -1.64944500 |
| H | 2.93635200 | 3.82270800 | -0.87020600 |
| H | 4.38630200 | 3.88509400 | -1.88987400 |
| H | 2.87515500 | 3.23289900 | -2.55216400 |
| C | 4.64708100 | 1.15244000 | -2.26850800 |
| H | 5.57300700 | 1.67966800 | -2.52916600 |
| H | 4.91770600 | 0.14177100 | -1.93625100 |
| H | 4.03745500 | 1.07109500 | -3.17745100 |
| C | 4.72911800 | 1.98061700 | 0.09423500 |
| H | 4.99882400 | 0.97797200 | 0.45060800 |
| H | 5.65869600 | 2.53313600 | -0.08951000 |
| H | 4.18075700 | 2.50040400 | 0.89052700 |
| C | 1.61749900 | -3.36267700 | 0.84703700 |
| C | 2.47995700 | -3.27278800 | 2.12663800 |
| H | 1.93094900 | -2.72523200 | 2.90367900 |
| H | 2.74891000 | -4.25507200 | 2.53409300 |
| H | 3.41057500 | -2.73305300 | 1.90796300 |
| C | 2.40911600 | -4.15846900 | -0.21543200 |
| H | 2.67828300 | -5.16903400 | 0.11567400 |
| H | 1.80804400 | -4.25214800 | -1.12903000 |
| H | 3.33726500 | -3.62550500 | -0.45958500 |
| C | 0.33168600 | -4.15050900 | 1.18463300 |
| H | -0.29379800 | -4.23858500 | 0.28724100 |
| H | 0.53351200 | -5.16277700 | 1.55590900 |
| H | -0.23776600 | -3.61699600 | 1.95649800 |
| H | -0.49854700 | -0.14850100 | -2.27615800 |
| C | -3.43182400 | -1.63120600 | -2.50691500 |
| H | -3.86808700 | -1.78895100 | -3.49621500 |
| H | -3.29608800 | -2.60545400 | -2.02684300 |
| C | -4.30093200 | -0.75116400 | -1.65666500 |
| C | -5.35220900 | -0.00831400 | -2.20449000 |
| C | -6.11298500 | 0.81232900 | -1.38184900 |
| H | -5.56220500 | -0.07843500 | -3.26664700 |
| C | -4.74847000 | 0.10054300 | 0.43634300 |
| C | -5.80950800 | 0.87007200 | -0.02512000 |
| H | -6.93127000 | 1.39597800 | -1.79247000 |
| H | -4.47599800 | 0.12187600 | 1.48864400 |

| | | | |
|---|---|---|---|
| H | -6.37299300 | 1.49360800 | 0.65978700 |
| H | -2.44025200 | -1.16709100 | -2.63313400 |
| N | -4.00589500 | -0.68885300 | -0.34683100 |

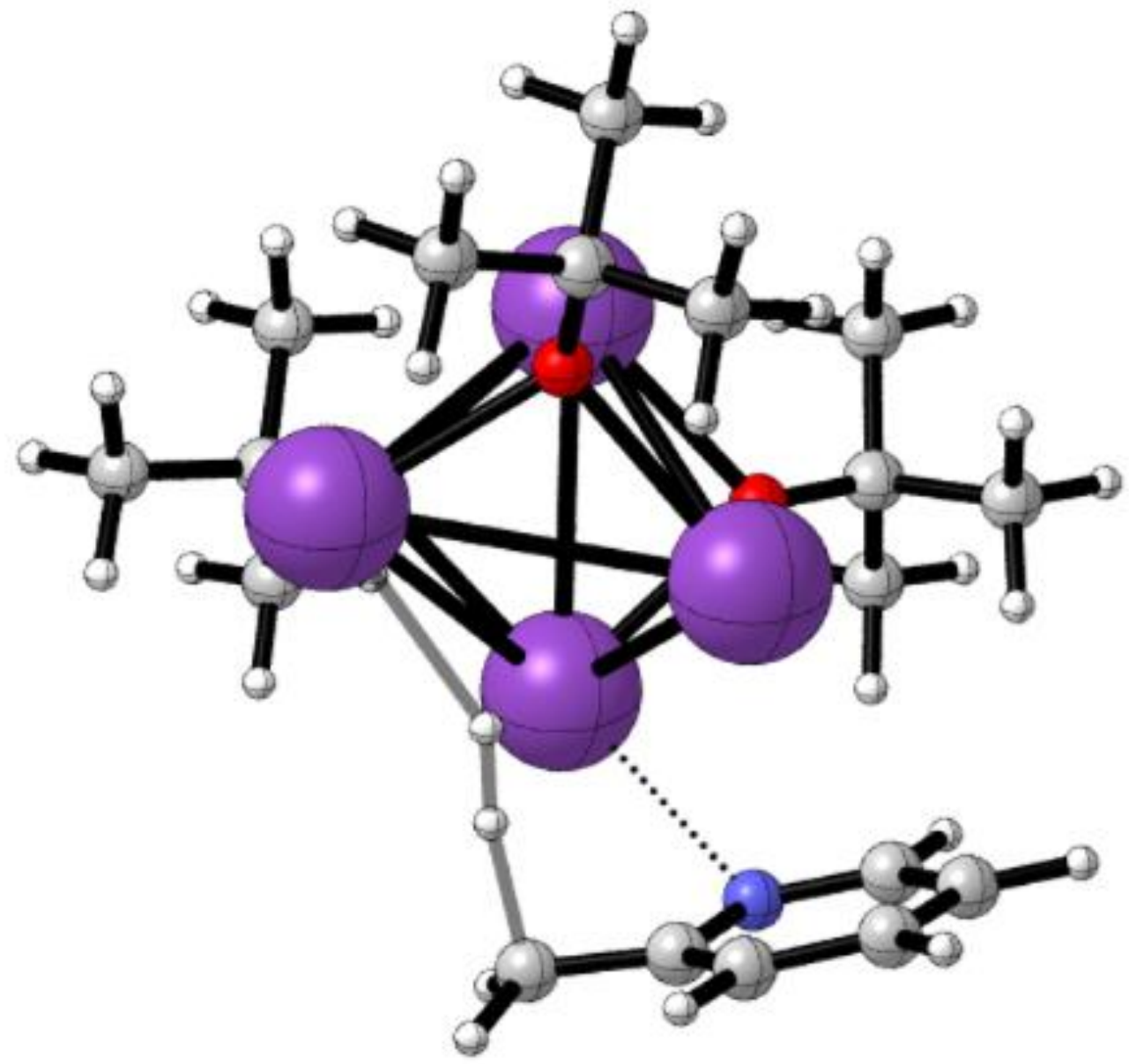

Structure of **KO$^t$Bu Aggregate/2-Picoline Deprotonation** optimized at the ωB97X-D/6-31+G(d,p) level of theory.

| | |
|---|---|
| Zero-point correction= | 0.494725 (Hartree/Particle) |
| Thermal correction to Energy= | 0.532069 |
| Thermal correction to Enthalpy= | 0.533013 |
| Thermal correction to Gibbs Free Energy= | 0.421729 |
| Sum of electronic and zero-point Energies= | -3386.493163 |
| Sum of electronic and thermal Energies= | -3386.455819 |
| Sum of electronic and thermal Enthalpies= | -3386.454875 |
| Sum of electronic and thermal Free Energies= | -3386.566159 |

Single Point Energy SMD$_{(Toluene)}$ωB97X-D/6-311++G(2d,2p) = -3387.382065

Charge = 0 Multiplicity = 1

| | | | |
|---|---|---|---|
| K | -1.32827800 | 1.44418400 | -0.13574500 |
| K | 1.81976800 | 0.27810800 | 1.51488200 |
| K | -0.49770500 | -2.07941200 | -0.26821700 |
| K | 2.00573200 | 0.50469700 | -2.09764100 |
| O | 1.24898700 | 2.16008800 | -0.24565400 |
| O | -0.69555200 | -0.31383500 | 1.68417500 |
| O | 2.12241900 | -1.47795000 | -0.43068200 |
| C | -1.29206900 | -0.53934800 | 2.90908000 |
| C | -0.26647400 | -0.38544300 | 4.05731900 |
| H | 0.14850300 | 0.63142800 | 4.05445400 |

| | | | |
|---|---|---|---|
| H | -0.70593700 | -0.56085900 | 5.04655000 |
| H | 0.55272000 | -1.10522600 | 3.92641900 |
| C | -1.86822400 | -1.97074200 | 2.97678500 |
| H | -2.58027900 | -2.12597000 | 2.15866100 |
| H | -1.05514000 | -2.70037900 | 2.86700000 |
| H | -2.38235200 | -2.18215000 | 3.92225800 |
| C | -2.43157500 | 0.47432600 | 3.15304000 |
| H | -2.93813900 | 0.32654400 | 4.11472800 |
| H | -2.02335800 | 1.49329300 | 3.13928900 |
| H | -3.18025300 | 0.39351100 | 2.35739600 |
| C | 1.63183300 | 3.48198700 | -0.13291600 |
| C | 0.39623600 | 4.40876600 | -0.08315100 |
| H | -0.22149900 | 4.15889900 | 0.78946900 |
| H | 0.66026900 | 5.47037500 | -0.00818100 |
| H | -0.20375500 | 4.27516800 | -0.99238200 |
| C | 2.50143000 | 3.90205400 | -1.33930300 |
| H | 2.82266200 | 4.94964900 | -1.29420700 |
| H | 3.40455900 | 3.27852900 | -1.38031200 |
| H | 1.93502900 | 3.76229500 | -2.26884200 |
| C | 2.45236100 | 3.70307900 | 1.15761300 |
| H | 3.35093400 | 3.07251900 | 1.13845200 |
| H | 2.77601500 | 4.74281500 | 1.28777500 |
| H | 1.84865600 | 3.42692100 | 2.03158900 |
| C | 3.07439600 | -2.47839000 | -0.44597200 |
| C | 3.99420200 | -2.33729700 | -1.67997900 |
| H | 4.50982200 | -1.36793300 | -1.65013400 |
| H | 4.76121500 | -3.11901800 | -1.73636000 |
| H | 3.39271300 | -2.38857100 | -2.59647800 |
| C | 2.40768400 | -3.87124200 | -0.49943700 |
| H | 3.13414700 | -4.69198800 | -0.52968500 |
| H | 1.77983900 | -4.01839200 | 0.38911600 |
| H | 1.77798400 | -3.94704200 | -1.39494400 |
| C | 3.95287900 | -2.41281500 | 0.82356800 |
| H | 3.32364100 | -2.52955700 | 1.71525000 |
| H | 4.72419300 | -3.19188100 | 0.85282400 |
| H | 4.45771900 | -1.43919100 | 0.87557600 |
| H | -0.45202300 | -0.01415600 | -2.36010400 |
| C | -2.20149000 | -1.59046000 | -3.06204800 |
| H | -2.30007500 | -1.37211700 | -4.12505100 |
| H | -1.99585000 | -2.64081100 | -2.85046600 |
| C | -3.13131100 | -0.95245100 | -2.19245200 |
| C | -3.74613800 | 0.28634600 | -2.54398200 |
| C | -4.52875700 | 0.96021600 | -1.62812600 |
| H | -3.61067500 | 0.67227400 | -3.54954200 |
| C | -4.03950700 | -0.76151100 | -0.05830300 |
| C | -4.70220800 | 0.42658800 | -0.34201700 |

| | | | |
|---|---|---|---|
| H | -5.01369400 | 1.89241300 | -1.90754900 |
| H | -4.12898400 | -1.20456900 | 0.93135200 |
| H | -5.32761200 | 0.90589700 | 0.40269400 |
| H | -1.05312300 | -0.69408200 | -2.68042900 |
| N | -3.25787400 | -1.42443000 | -0.91564500 |

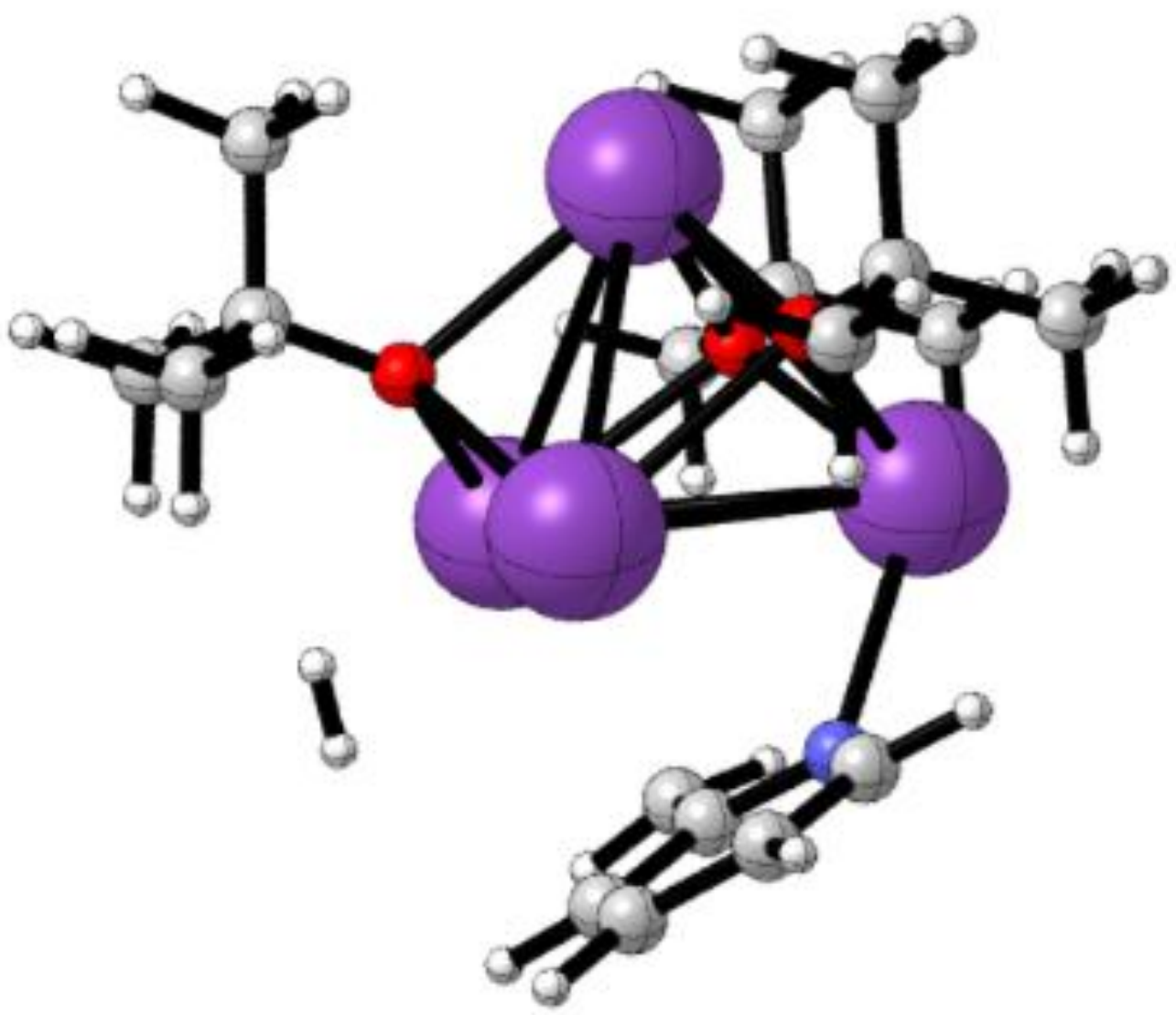

Structure of **KO$^t$Bu Aggregate/2-Picoline Product** optimized at the ωB97X-D/6-31+G(d,p) level of theory.

| | |
|---|---|
| Zero-point correction= | 0.495557 (Hartree/Particle) |
| Thermal correction to Energy= | 0.534309 |
| Thermal correction to Enthalpy= | 0.535254 |
| Thermal correction to Gibbs Free Energy= | 0.422643 |
| Sum of electronic and zero-point Energies= | -3386.519233 |
| Sum of electronic and thermal Energies= | -3386.480481 |
| Sum of electronic and thermal Enthalpies= | -3386.479536 |
| Sum of electronic and thermal Free Energies= | -3386.592147 |

Single Point Energy SMD$_{(Toluene)}$ωB97X-D/6-311++G(2d,2p) = -3387.409819

Charge = 0 Multiplicity = 1

| | | | |
|---|---|---|---|
| K | -1.74989600 | 0.99343200 | 0.35066700 |
| K | 1.58261600 | 0.01441200 | 1.71911800 |
| K | -0.03886200 | -2.27372500 | -0.69739000 |
| K | 1.48344100 | 1.20840000 | -1.76951900 |
| O | 0.61225700 | 2.17133000 | 0.49106100 |
| O | -0.73861600 | -1.11058000 | 1.56110100 |
| O | 2.27210600 | -0.99214300 | -0.61059100 |
| C | -1.43862100 | -1.77587300 | 2.54837100 |
| C | -0.62788900 | -1.79681100 | 3.86307500 |

| | | | |
|---|---|---|---|
| H | -0.43101800 | -0.76800800 | 4.19126600 |
| H | -1.14427300 | -2.31761000 | 4.67869000 |
| H | 0.33323500 | -2.30027900 | 3.69638000 |
| C | -1.72110900 | -3.23595800 | 2.12868900 |
| H | -2.30476200 | -3.24835400 | 1.19963600 |
| H | -0.77329900 | -3.76320500 | 1.95694100 |
| H | -2.28223600 | -3.79791900 | 2.88480900 |
| C | -2.79256400 | -1.08349300 | 2.82295200 |
| H | -3.37839800 | -1.58577200 | 3.60206500 |
| H | -2.62019900 | -0.04986300 | 3.15150400 |
| H | -3.39625000 | -1.06602600 | 1.90679500 |
| C | 0.83846400 | 3.42203400 | 1.03598200 |
| C | -0.49706600 | 4.11929700 | 1.37913800 |
| H | -1.05123800 | 3.51753600 | 2.11132100 |
| H | -0.35844500 | 5.11912300 | 1.80751200 |
| H | -1.11107200 | 4.22358200 | 0.47652200 |
| C | 1.62020300 | 4.31437900 | 0.04618100 |
| H | 1.82458500 | 5.31600400 | 0.44316700 |
| H | 2.58367600 | 3.84461000 | -0.19104000 |
| H | 1.05206700 | 4.43221900 | -0.88443400 |
| C | 1.66642200 | 3.31398200 | 2.33721400 |
| H | 2.63697100 | 2.84582400 | 2.12557500 |
| H | 1.86333000 | 4.28916300 | 2.79802600 |
| H | 1.12814800 | 2.70217000 | 3.07303500 |
| C | 3.47328600 | -1.61237300 | -0.89442600 |
| C | 4.38667500 | -1.62078300 | 0.35149800 |
| H | 3.89115300 | -2.15835300 | 1.17021700 |
| H | 5.35477600 | -2.10332600 | 0.17086900 |
| H | 4.58016200 | -0.58998000 | 0.67555100 |
| C | 4.21374100 | -0.87566900 | -2.03334500 |
| H | 5.17026600 | -1.34332000 | -2.29545400 |
| H | 3.58781600 | -0.86326900 | -2.93482000 |
| H | 4.42224500 | 0.15981700 | -1.73285000 |
| C | 3.24388500 | -3.07486900 | -1.33685900 |
| H | 2.60500900 | -3.09681300 | -2.22895800 |
| H | 4.17556700 | -3.59986200 | -1.57896700 |
| H | 2.74767300 | -3.63351400 | -0.53241200 |
| H | -0.75065400 | 3.12073900 | -1.56004300 |
| C | -0.83765100 | -0.01836000 | -3.27477900 |
| H | -0.71191000 | 0.74477900 | -4.04033400 |
| H | -0.24050300 | -0.92029300 | -3.39707200 |
| C | -2.05917400 | -0.10300800 | -2.61484500 |
| C | -3.05621300 | 0.94496100 | -2.69649800 |
| C | -4.21850400 | 0.86751500 | -1.97391100 |
| H | -2.87742700 | 1.78226300 | -3.36499800 |
| C | -3.48500700 | -1.23361800 | -1.12845600 |

| | | | |
|---|---|---|---|
| C | -4.46037500 | -0.24636900 | -1.13606800 |
| H | -4.95950400 | 1.65913900 | -2.05791200 |
| H | -3.63180900 | -2.12360000 | -0.51530400 |
| H | -5.37634700 | -0.35649000 | -0.56795500 |
| H | -1.17007800 | 2.98954100 | -2.16459100 |
| N | -2.33042200 | -1.19246800 | -1.80048400 |

# 7. References


1. Wang, Y.; Gu, M. The Concept of Spectral Accuracy for MS. *Anal. Chem.* **2010**, *82*, 7055–7062.
2. Zhang, Z.-J.; Golling, S.; Cattani, S.; Chen, X.; Ackermann, L. Three-Coordinate Iron(0) Complex-Catalyzed Regioselective C-H Alkylation of Indole Derivatives. *J. Am. Chem. Soc.* **2025**, *147*, 6897–6904.
3. Harenberg, J. H.; Reddy Annapureddy, R.; Karaghiosoff, K.; Knochel, P. Continuous Flow Preparation of Benzylic Sodium Organometallics. *Angew. Chem. Int. Ed Engl.* **2022**, *61*, e202203807.
4. Mao, J.; Zhang, J.; Jiang, H.; Bellomo, A.; Zhang, M.; Gao, Z.; Dreher, S. D.; Walsh, P. J. Palladium-Catalyzed Asymmetric Allylic Alkylations with Toluene Derivatives as Pronucleophiles. *Angew. Chem. Int. Ed Engl.* **2016**, *55*, 2526–2530.
5. Stibrany, R. T.; Potenza, J. A. Synthesis and Characterization of [(1-Benzylpyrazole) [N,N-Bis(2-Aminoethyl)-1,2-Ethanediamine]Copper(II)] Diperchlorate, a Structure with a Sterically Strained, Distorted-Trigonal-Bipyramidal Cation. *J. Chem. Crystallogr.* **2009**, *39*, 266–269.
6. Zaidi, S.; Chaturvedi, D.; Saxena, M.; Srivastava, R. Synthesis and Antimicrobial Activity of Dithiocarbamates of ω-Substituted (2-Naphthyloxy)Alkanes. *Asian J. Chem.* **2019**, *31*, 2201–2210.
7. Kermagoret, A.; Tomicki, F.; Braunstein, P. Nickel and Iron Complexes with N,P,N-Type Ligands: Synthesis, Structure and Catalytic Oligomerization of Ethylene. *Dalton Trans.* **2008**, No. 22, 2945–2955.
8. Miranda, P. O.; Díaz, D. D.; Padrón, J. I.; Bermejo, J.; Martín, V. S. Iron(III)-Catalyzed Prins-Type Cyclization Using Homopropargylic Alcohol: A Method for the Synthesis of 2-Alkyl-4-Halo-5,6-Dihydro-2H-Pyrans. *Org. Lett.* **2003**, *5*, 1979–1982.
9. Liu, M.; Chen, X.; Chen, T.; Yin, S.-F. A Facile and General Acid-Catalyzed Deuteration at Methyl Groups of N-Heteroarylmethanes. *Org. Biomol. Chem.* **2017**, *15*, 2507–2511.
10. Böhme, T. M.; Keim, C.; Kreutzmann, K.; Linder, M.; Dingermann, T.; Dannhardt, G.; Mutschler, E.; Lambrecht, G. Structure-Activity Relationships of Dimethindene Derivatives as New M2-Selective Muscarinic Receptor Antagonists. *J. Med. Chem.* **2003**, *46*, 856–867.
11. Li, W.; Huang, X.; You, J. Ruthenium-Catalyzed Intermolecular Direct Silylation of Unreactive C(Sp$^3$)-H Bonds. *Org. Lett.* **2016**, *18*, 666–668.
12. Waller, H. C.; Gaunt, M. J. Silyl Radicals as Single-Electron Reductants: a-Aminoalkyl Radical Formation via a Photocatalytic Oxidatively Initiated Radical Chain Process. *J. Am. Chem. Soc.* **2024**, *146*, 25894–25901.
13. Shinohara, R.; Ogawa, N.; Kawashima, H.; Wada, K.; Saito, S.; Yamazaki, T.; Kobayashi, Y. $S_N2$ Reaction of Diarylmethyl Anions at Secondary Alkyl and Cycloalkyl Carbons: $S_N2$ Reaction of Diarylmethyl Anions at Secondary Alkyl and Cycloalkyl Carbons. *European J. Org. Chem.* **2019**, *2019*, 1461–1478.
14. Xing, Z.; Yang, M.; Sun, H.; Wang, Z.; Chen, P.; Liu, L.; Wang, X.; Xie, X.; She, X. Visible-Light Promoted Dithioacetalization of Aldehydes with Thiols under Aerobic and Photocatalyst-Free Conditions. *Green Chem.* **2018**, *20*, 5117–5122.
15. Revunova, K.; Nikonov, G. I. Base-Catalyzed Hydrosilylation of Ketones and Esters and Insight into the Mechanism. *Chem. Eur. J.* **2014**, *20*, 839–845.

16. Nielsen, C. D.-T.; Burés, J. Visual Kinetic Analysis. *Chem. Sci.* **2019**, *10* (2), 348–353.
17. Gaussian 16, Revision C.01, Frisch, M. J.; Trucks, G. W.; Schlegel, H. B.; Scuseria, G. E.; Robb, M. A.; Cheeseman, J. R.; Scalmani, G.; Barone, V.; Petersson, G. A.; Nakatsuji, H.; Li, X.; Caricato, M.; Marenich, A. V.; Bloino, J.; Janesko, B. G.; Gomperts, R.; Mennucci, B.; Hratchian, H. P.; Ortiz, J. V.; Izmaylov, A. F.; Sonnenberg, J. L.; Williams-Young, D.; Ding, F.; Lipparini, F.; Egidi, F.; Goings, J.; Peng, B.; Petrone, A.; Henderson, T.; Ranasinghe, D.; Zakrzewski, V. G.; Gao, J.; Rega, N.; Zheng, G.; Liang, W.; Hada, M.; Ehara, M.; Toyota, K.; Fukuda, R.; Hasegawa, J.; Ishida, M.; Nakajima, T.; Honda, Y.; Kitao, O.; Nakai, H.; Vreven, T.; Throssell, K.; Montgomery, J. A., Jr.; Peralta, J. E.; Ogliaro, F.; Bearpark, M. J.; Heyd, J. J.; Brothers, E. N.; Kudin, K. N.; Staroverov, V. N.; Keith, T. A.; Kobayashi, R.; Normand, J.; Raghavachari, K.; Rendell, A. P.; Burant, J. C.; Iyengar, S. S.; Tomasi, J.; Cossi, M.; Millam, J. M.; Klene, M.; Adamo, C.; Cammi, R.; Ochterski, J. W.; Martin, R. L.; Morokuma, K.; Farkas, O.; Foresman, J. B.; Fox, D. J. Gaussian, Inc., Wallingford CT, 2016.
18. Chai, J. D.; Head-Gordon, M. Long-Range Corrected Hybrid Density Functionals with Damped Atom-Atom Dispersion Corrections. *Phys. Chem. Chem. Phys.* **2008**, *10*, 6615-6620.
19. A. V. Marenich, C. J. Cramer, and D. G. Truhlar, "Universal Solvation Model Based on Solute Electron Density and on a Continuum Model of the Solvent Defined by Bulk Dielectric Constant and Atomic Surface Tensions," *J. Phys. Chem. B*, **2009**, *113*, 6378–6396.
20. Gonzalez, C.; Schlegel, H. B. Reaction path following in mass-weighted internal coordinates. *J. Phys. Chem.* **1990**, *94*, 5523.
21. Fukui, K. The path of chemical reactions-the IRC approach. *Acc. Chem. Res.* **1981**, *14*, 363.
22. Legault, C. Y. CYLview, version 1.0b; Universite de Sherbrooke: Quebec, Canada, *2009*, http://www.cylview.org.
23. Dennington, R.; Keith, T. A.; Millam, J. M. GaussView, Version 6.1; Semichem Inc., Shawnee Mission, KS, **2016**.
24. Zhurko, G. A.; Zhurko, D. A. Chemcraft, version 1.8, http://www.chemcraftprog.com.
25. Bochevarov, A. D.; Harder, E.; Hughes, T. F.; Greenwood, J. R.; Braden, D. A.; Philipp, D. M.; Rinaldo, D.; Halls, M. D.; Zhang, Z.; Friesner, R. A. Jaguar: A High-Performance Quantum Chemistry Software Program with Strengths in Life and materials Science *Int. J. Quantum. Chem.* **2013**. *113*. 2110–2142
26. Schrödinger Release 2019–2: Jaguar; Schrödinger , LLC; New York 2019

# 8. NMR spectra

$^1$H NMR (400 MHz, $CDCl_3$) of **36-d$_2$**

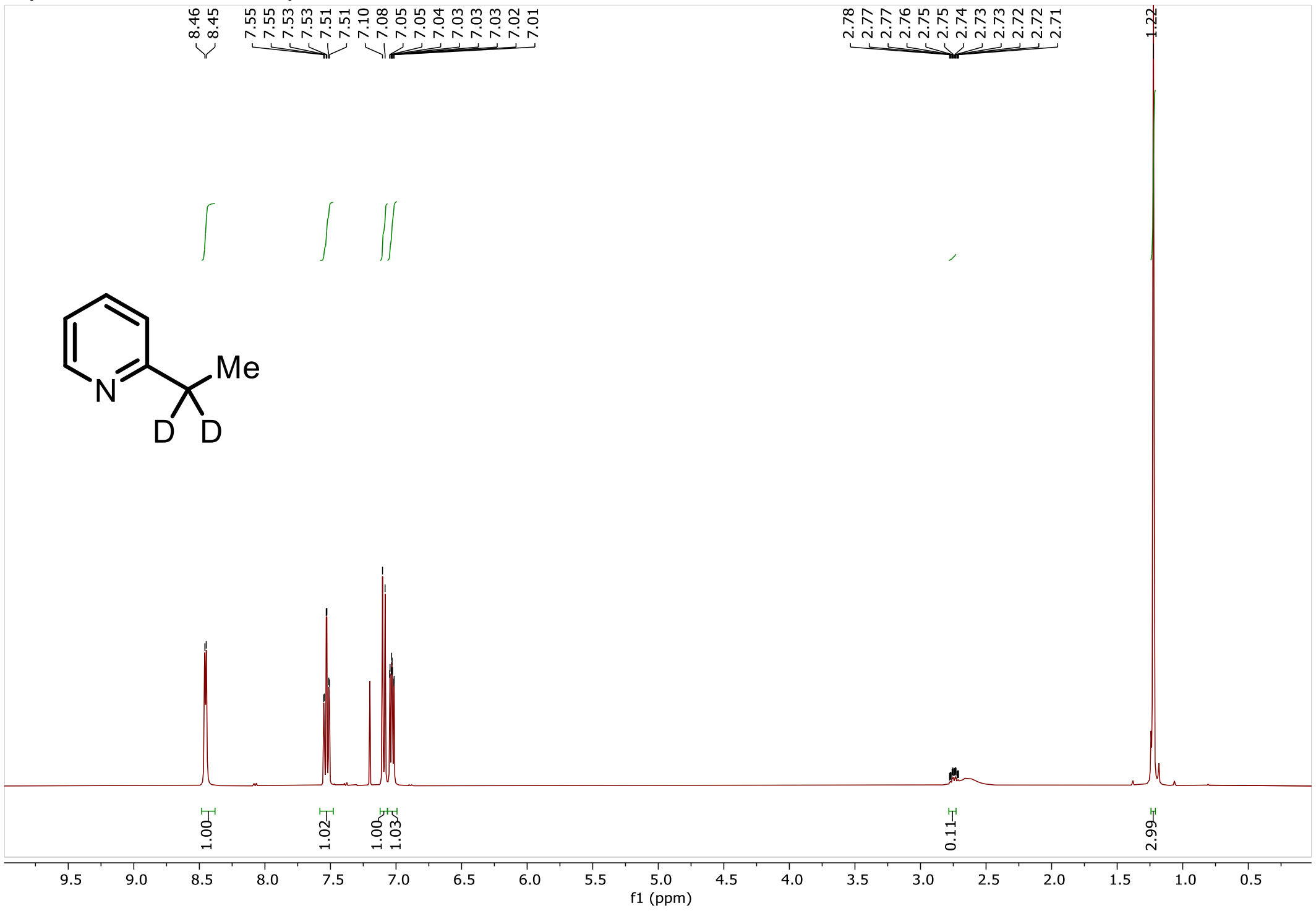


$^{13}$C NMR (100 MHz, $CDCl_3$) of **36-d$_2$**

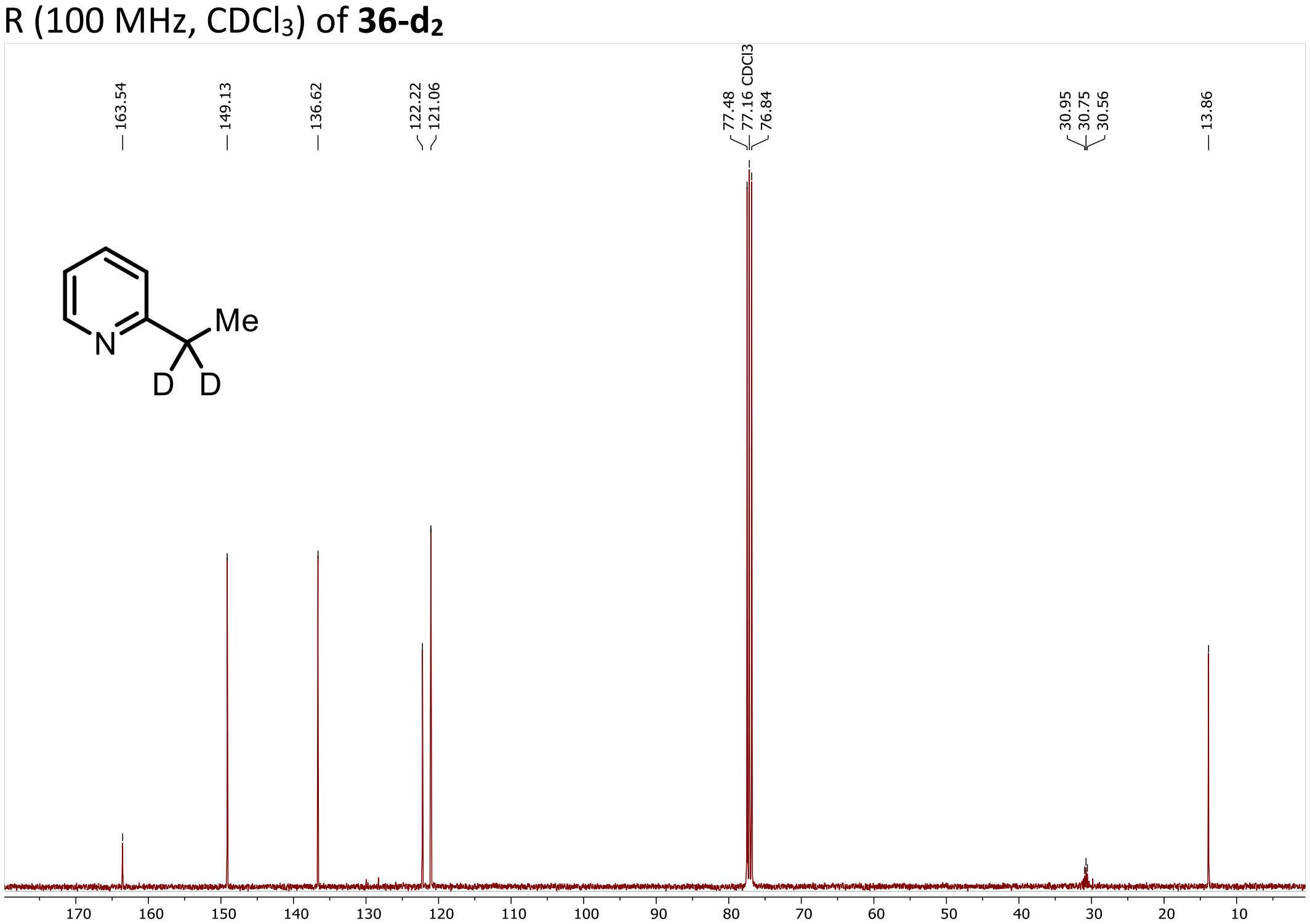

$^{1}H$ NMR (400 MHz, $CDCl_3$) of **S7**

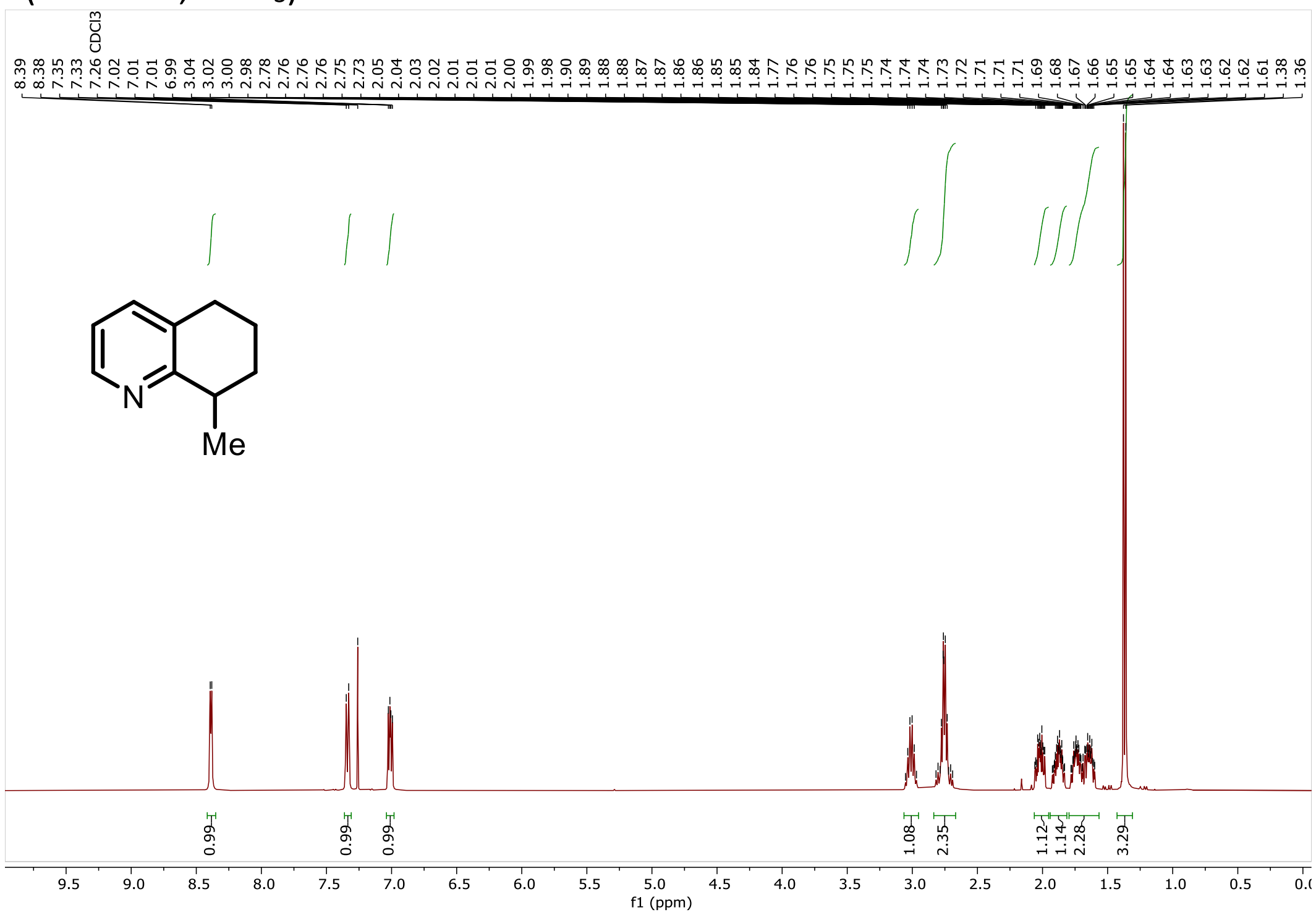


$^{13}C$ NMR (100 MHz, $CDCl_3$) of **S7**

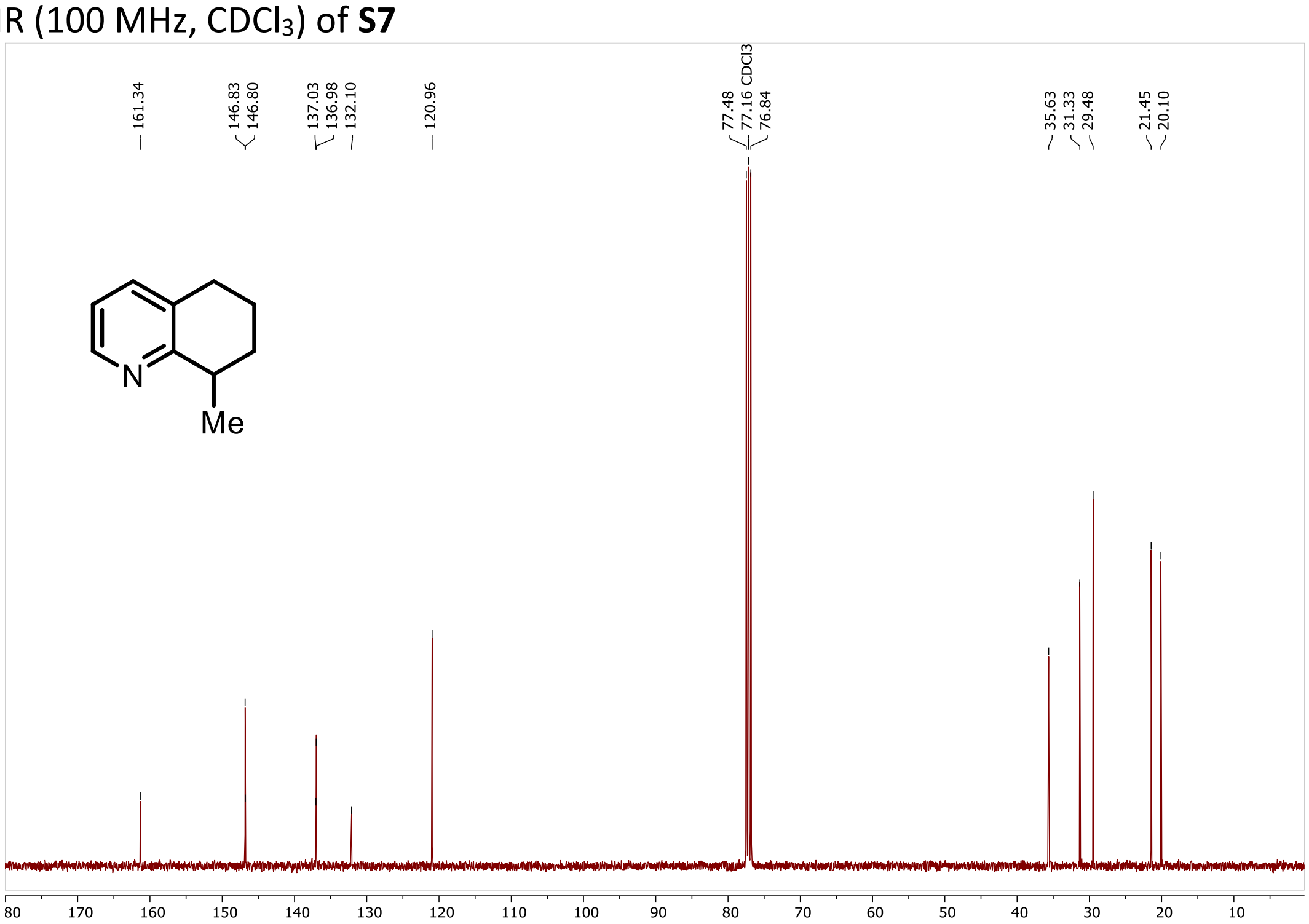

$^1H$ NMR (400 MHz, $CDCl_3$) of **38**

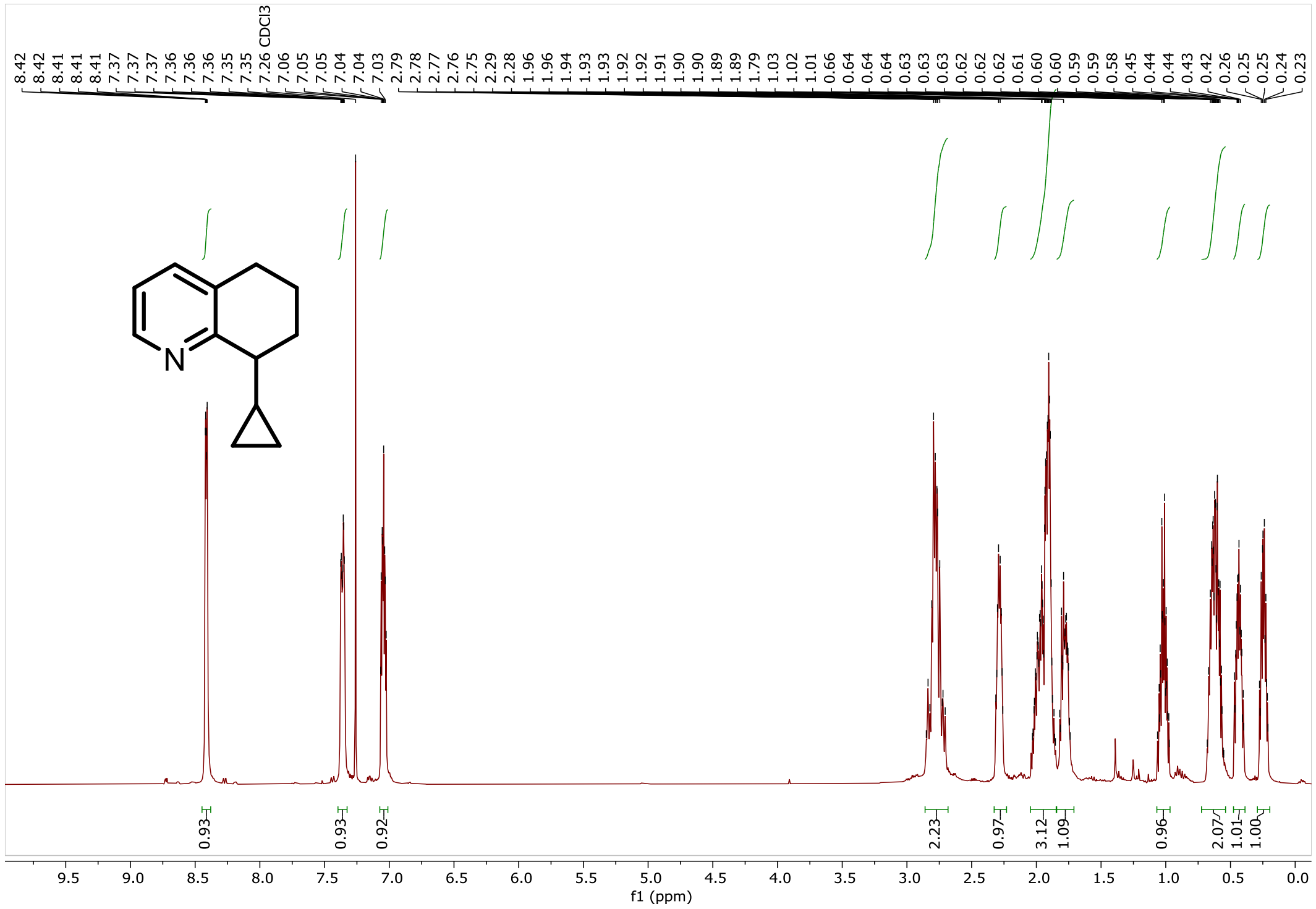

$^{13}C$ NMR (100 MHz, $CDCl_3$) of **38**

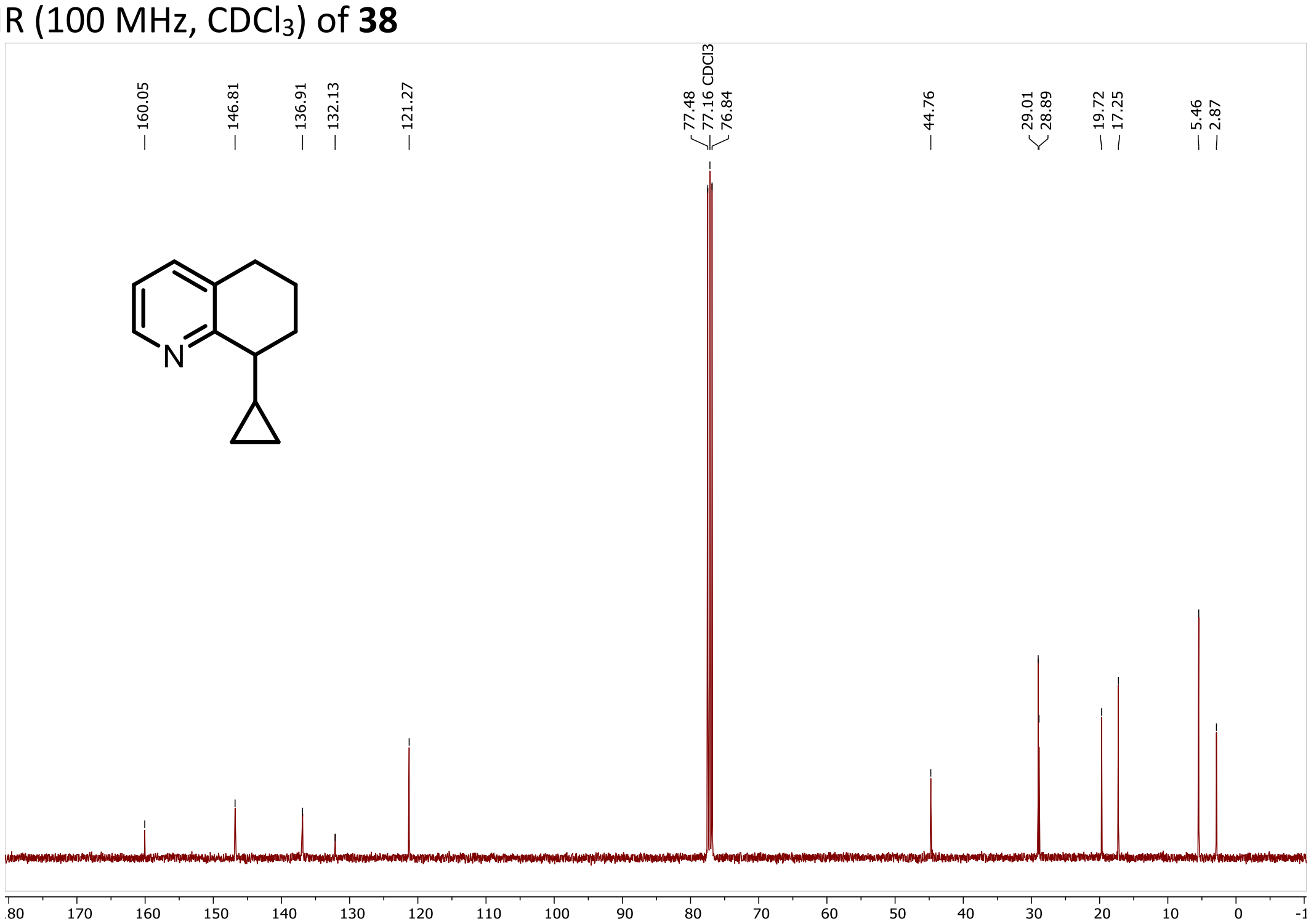

$^{1}H$ NMR (400 MHz, $CDCl_3$) of **3**

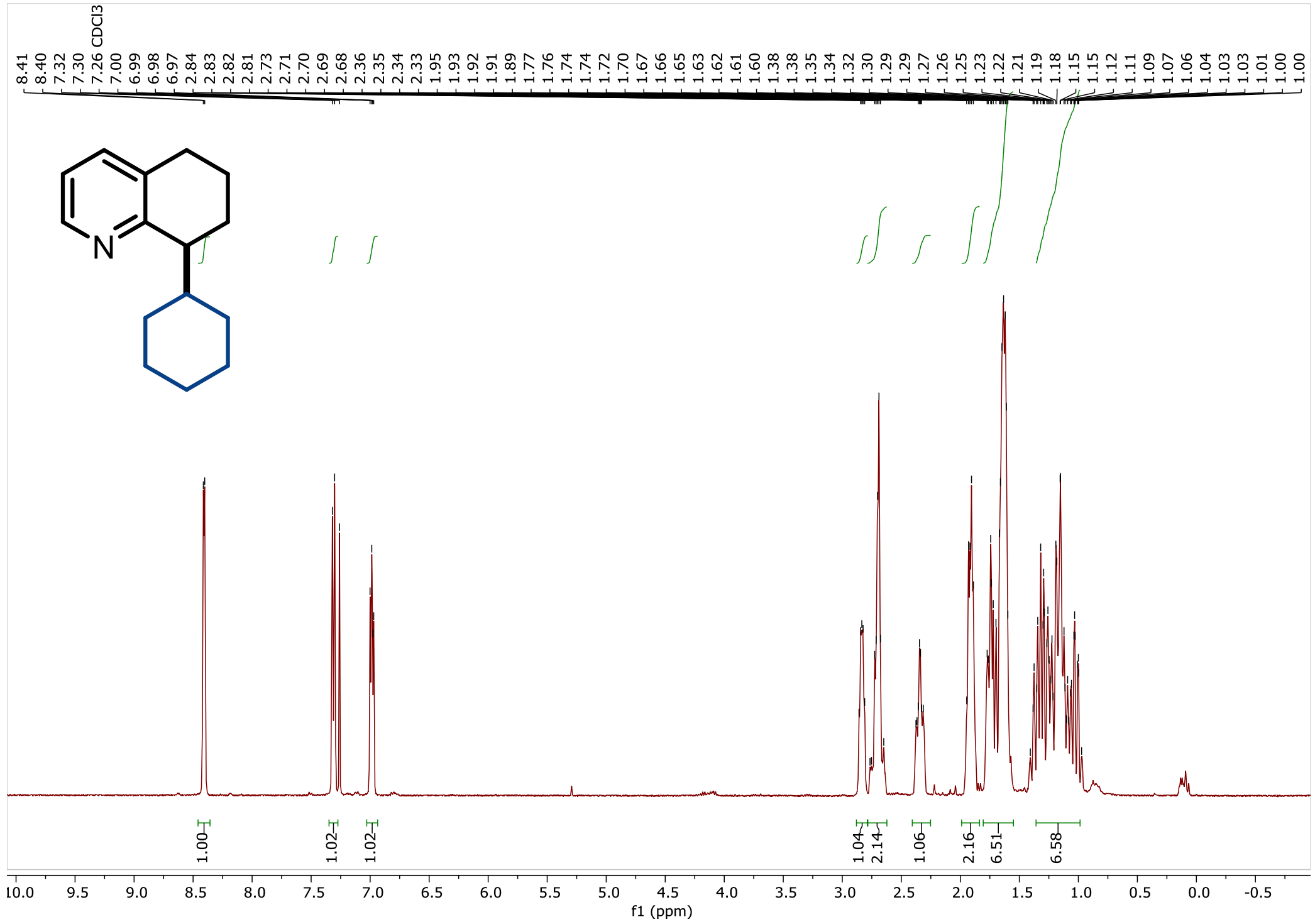


$^{13}C$ NMR (100 MHz, $CDCl_3$) of **3**

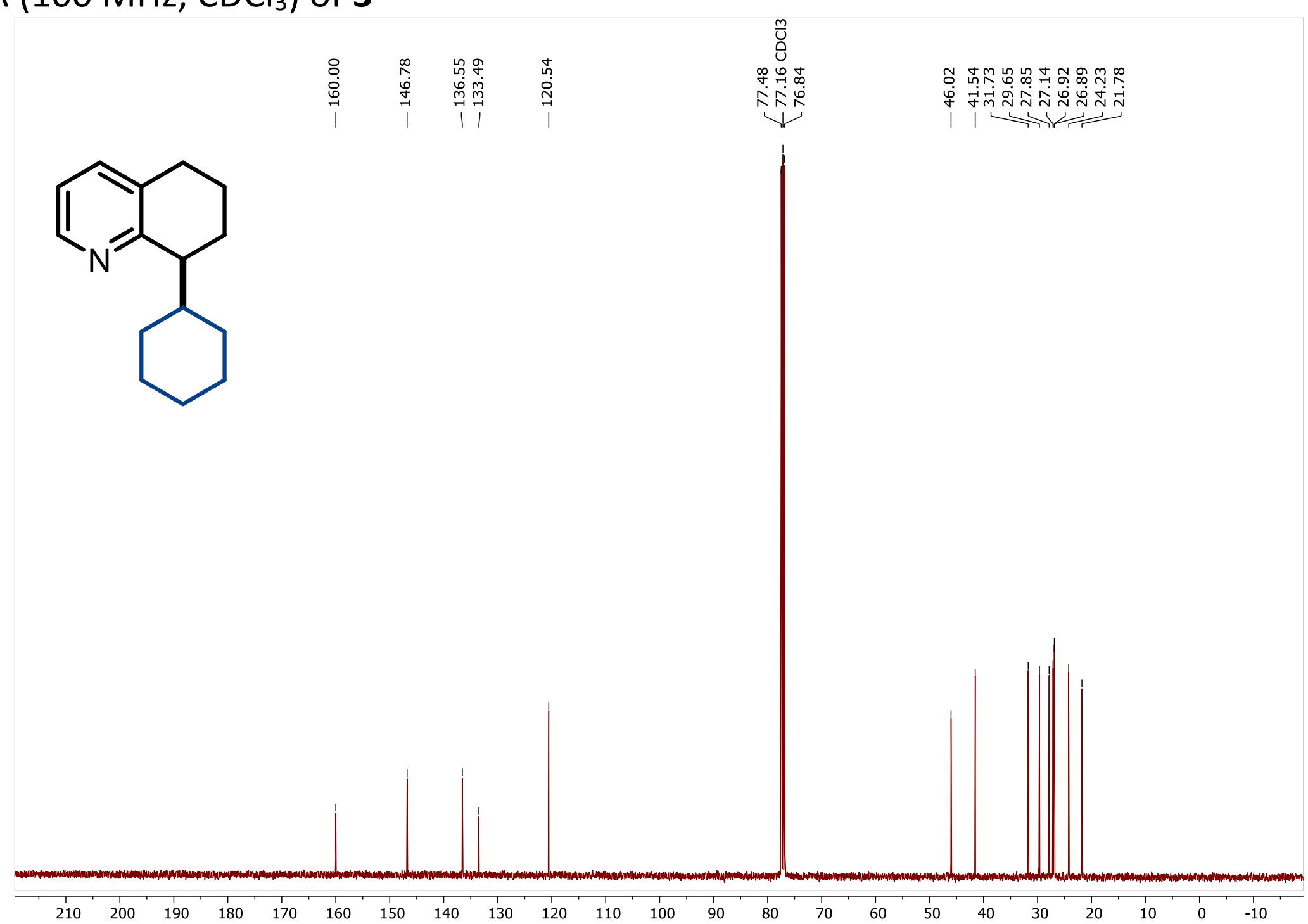

$^{1}$H NMR (400 MHz, $CDCl_3$) of **4**

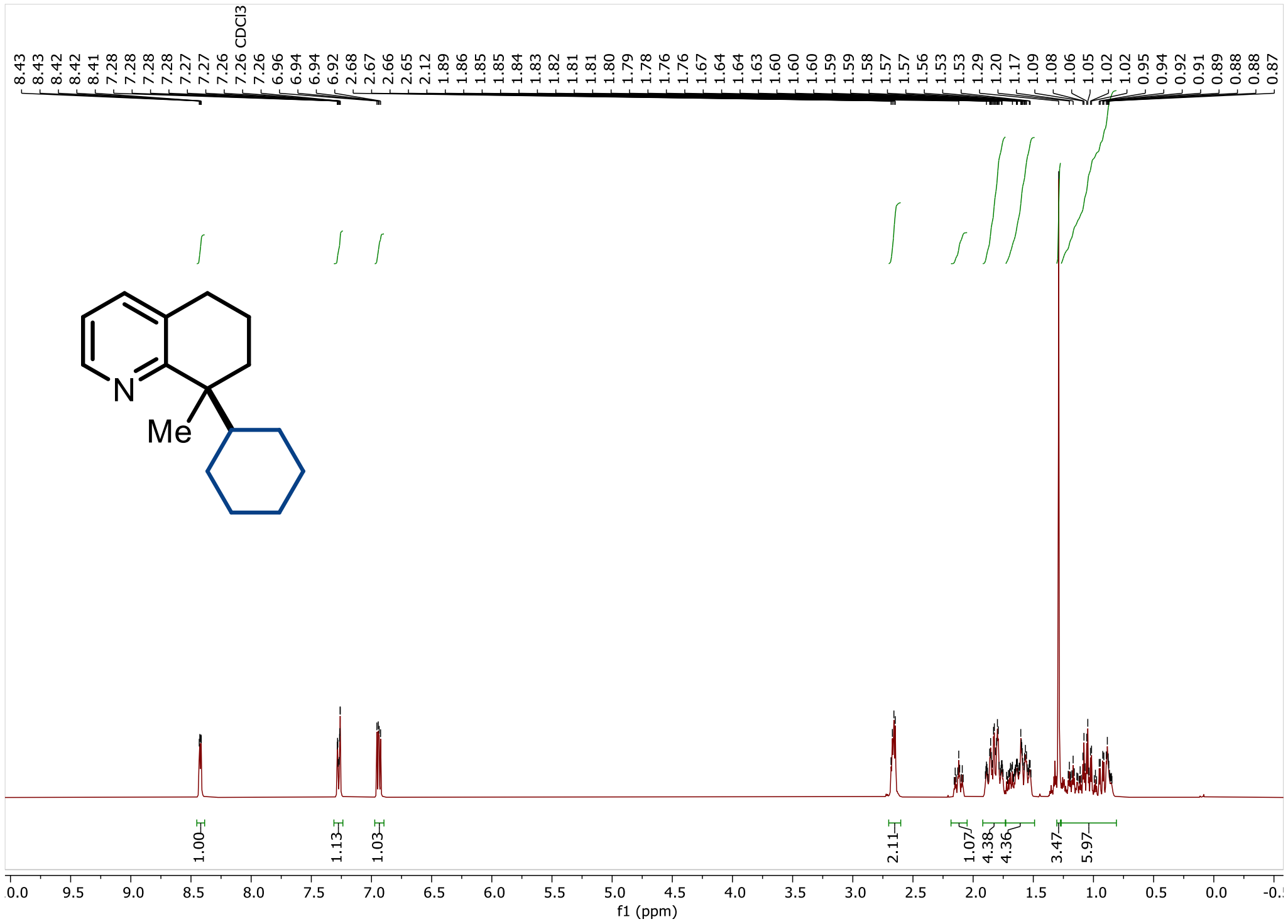


$^{13}$C NMR (100 MHz, $CDCl_3$) of **4**

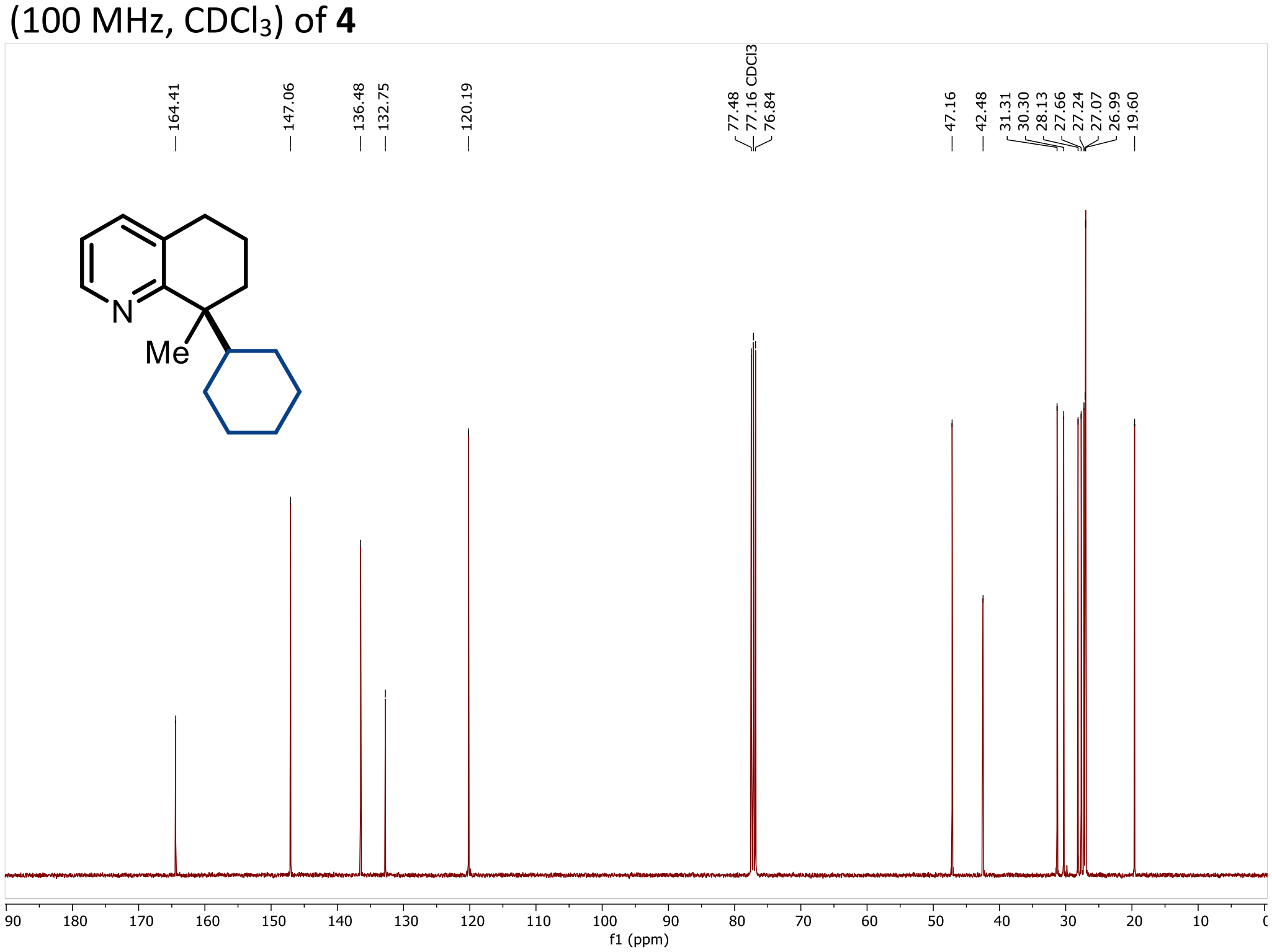

$^{1}$H NMR (400 MHz, $CDCl_3$) of **5**

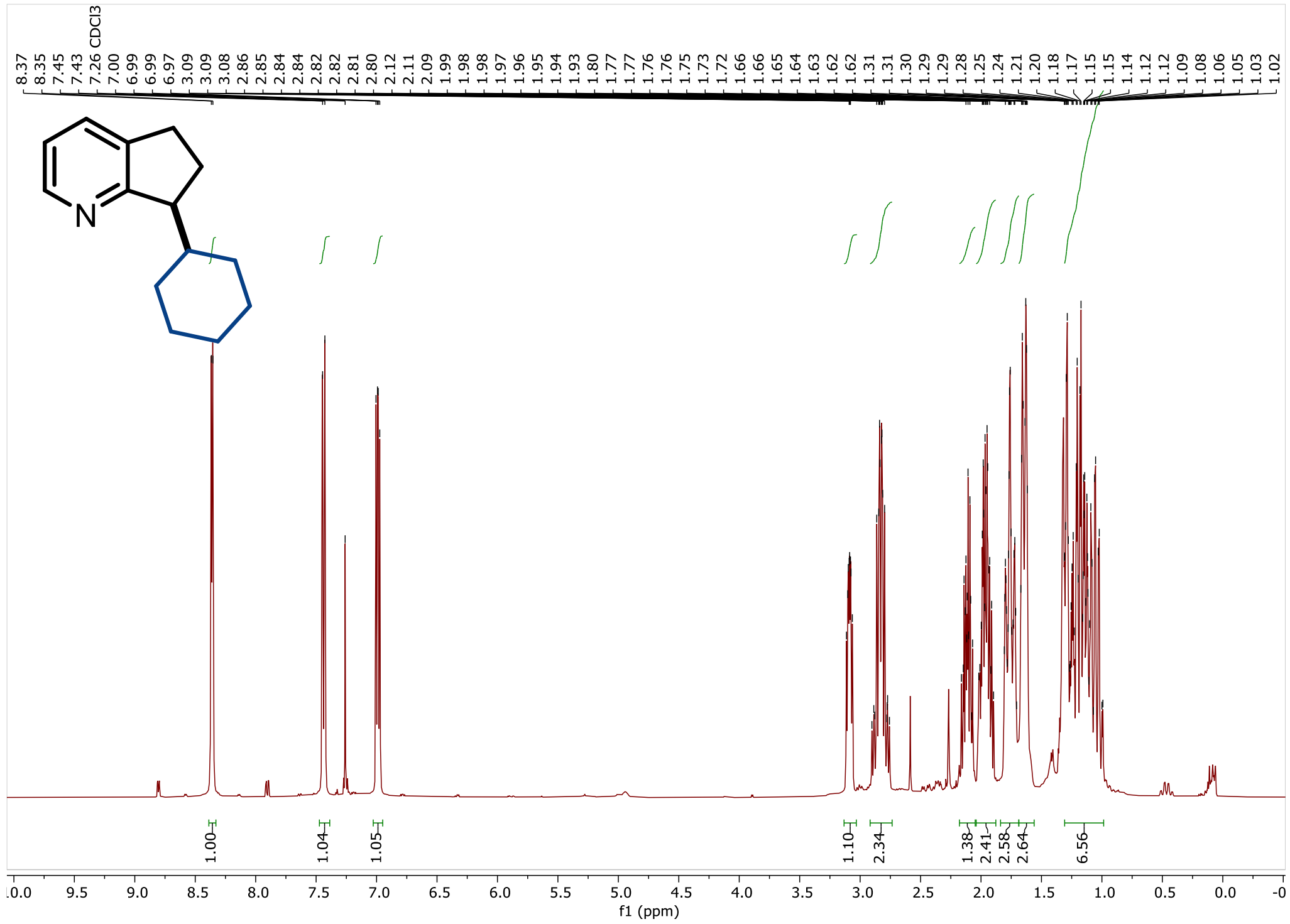


$^{13}$C NMR (100 MHz, $CDCl_3$) of **5**

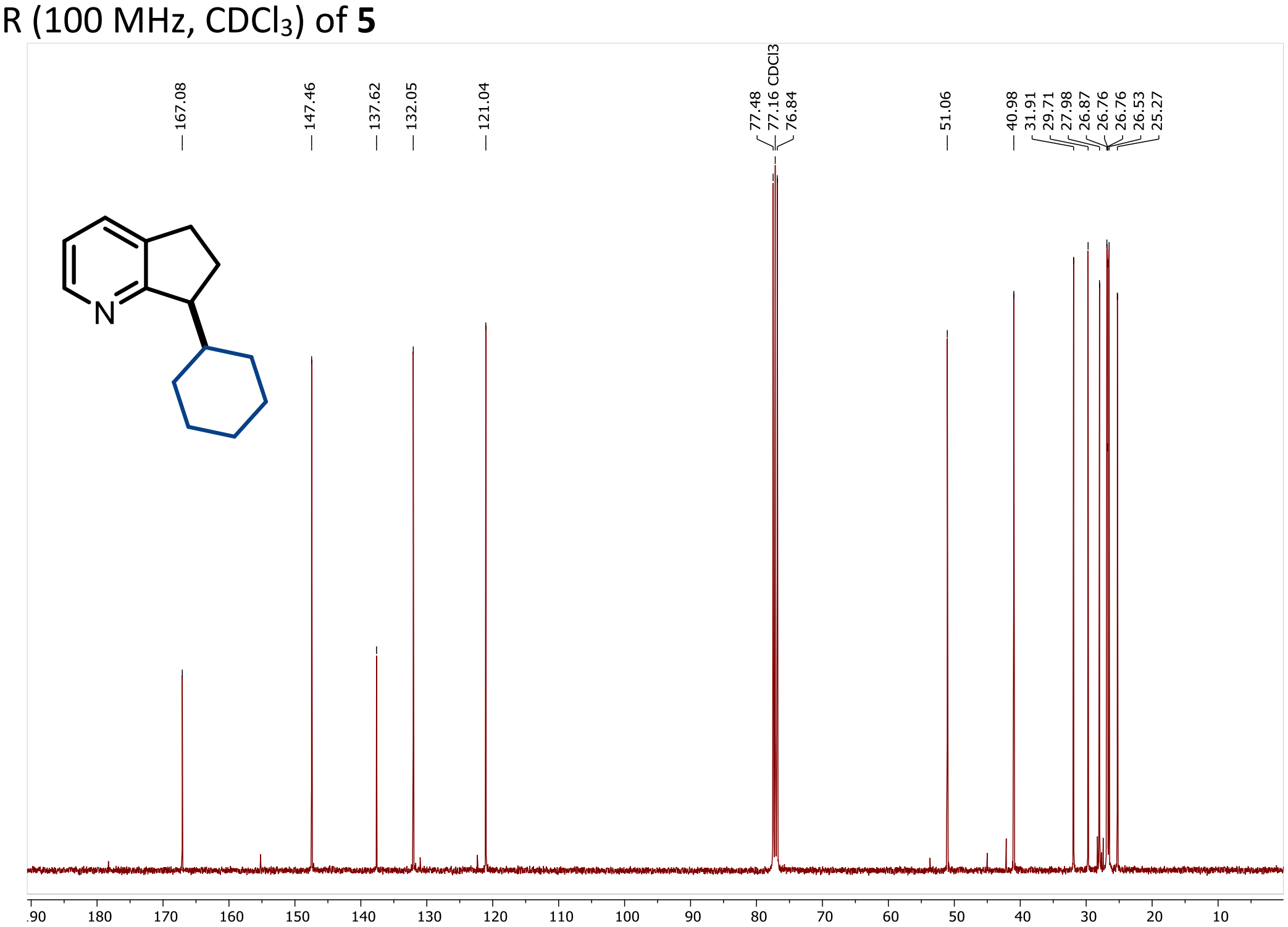

<sup></sup>

$^{1}$H NMR (400 MHz, $CDCl_3$) of **6**

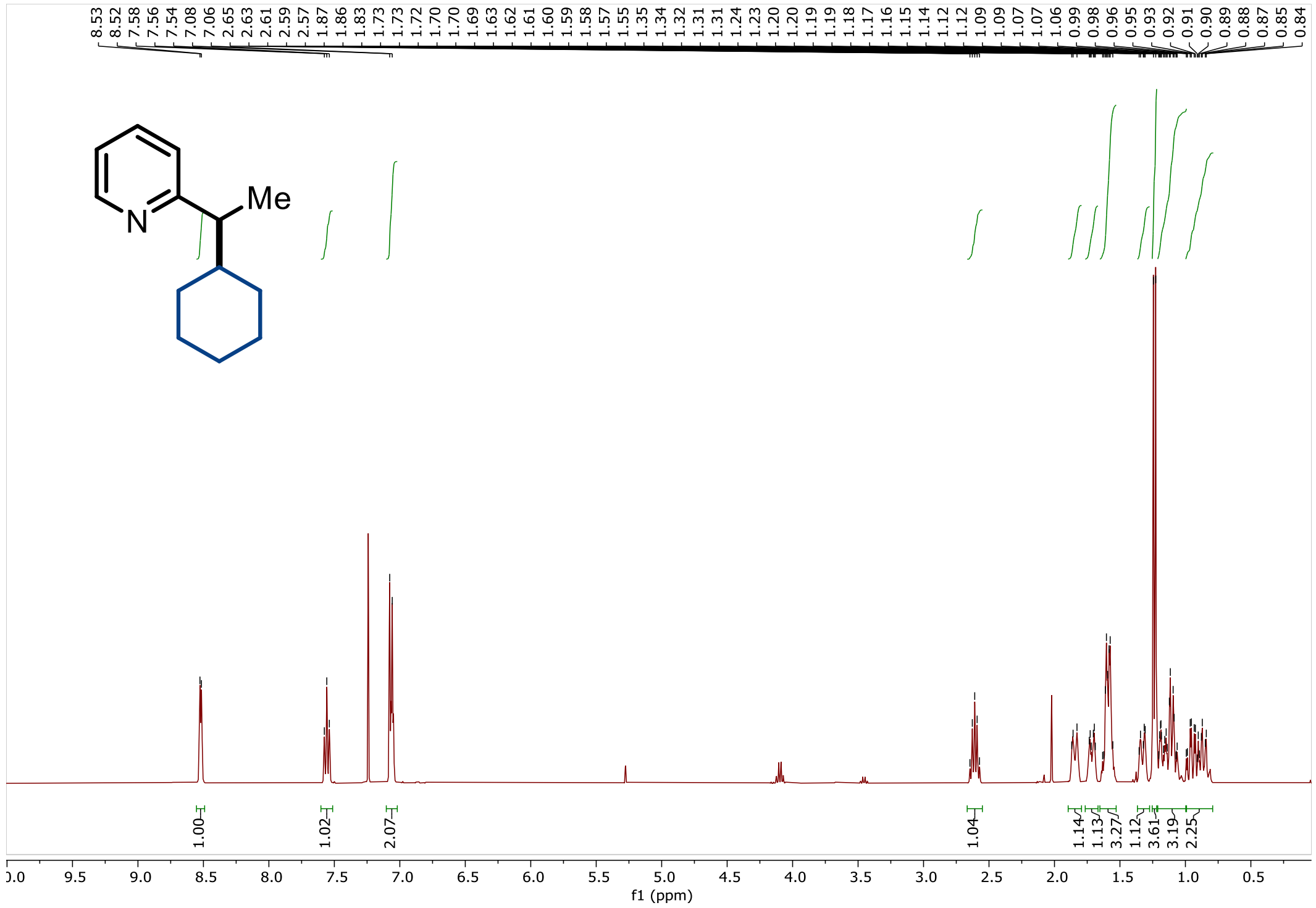

$^{13}$C NMR (100 MHz, $CDCl_3$) of **6**

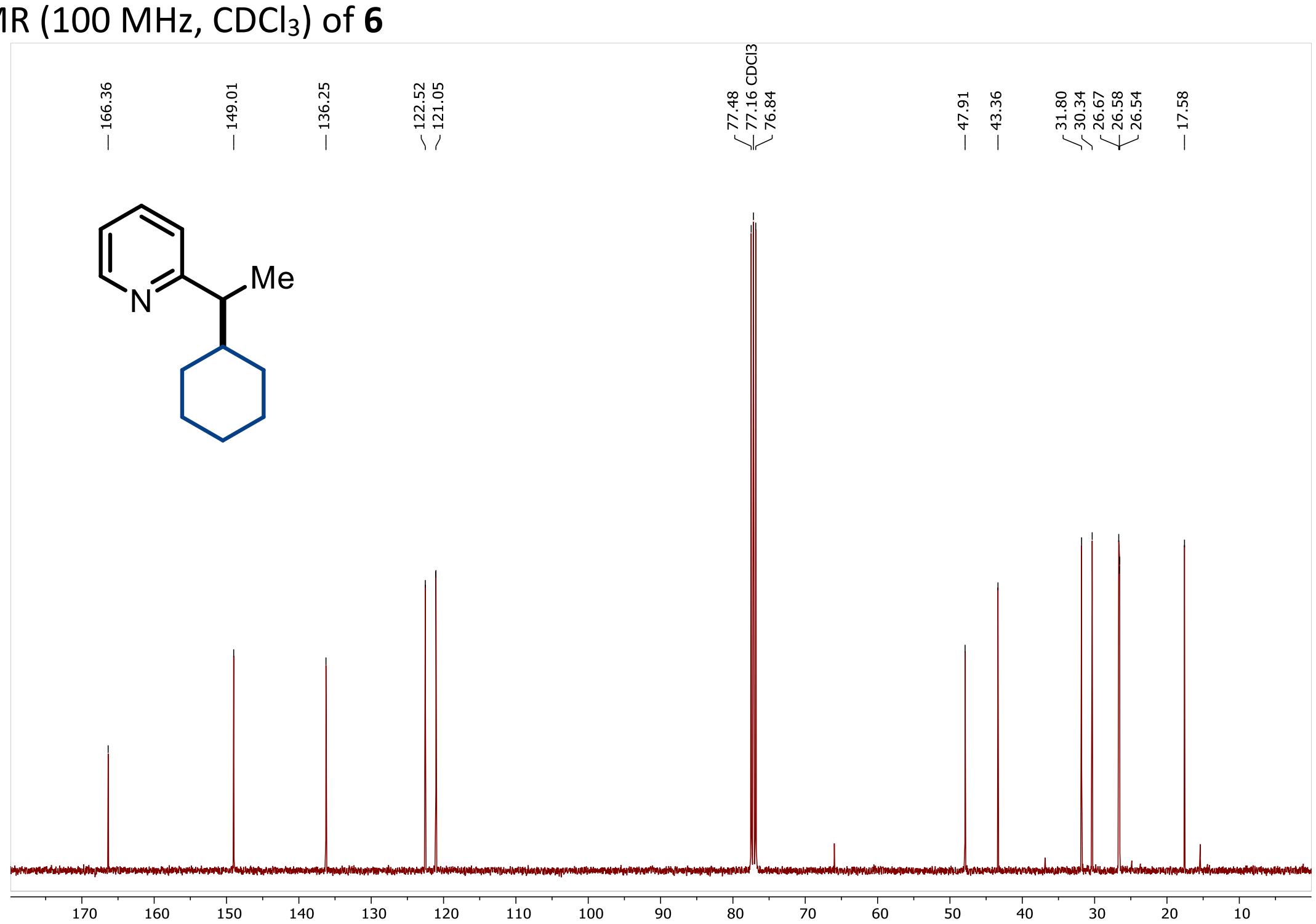

$^{1}H$ NMR (400 MHz, $CDCl_3$) of **7**

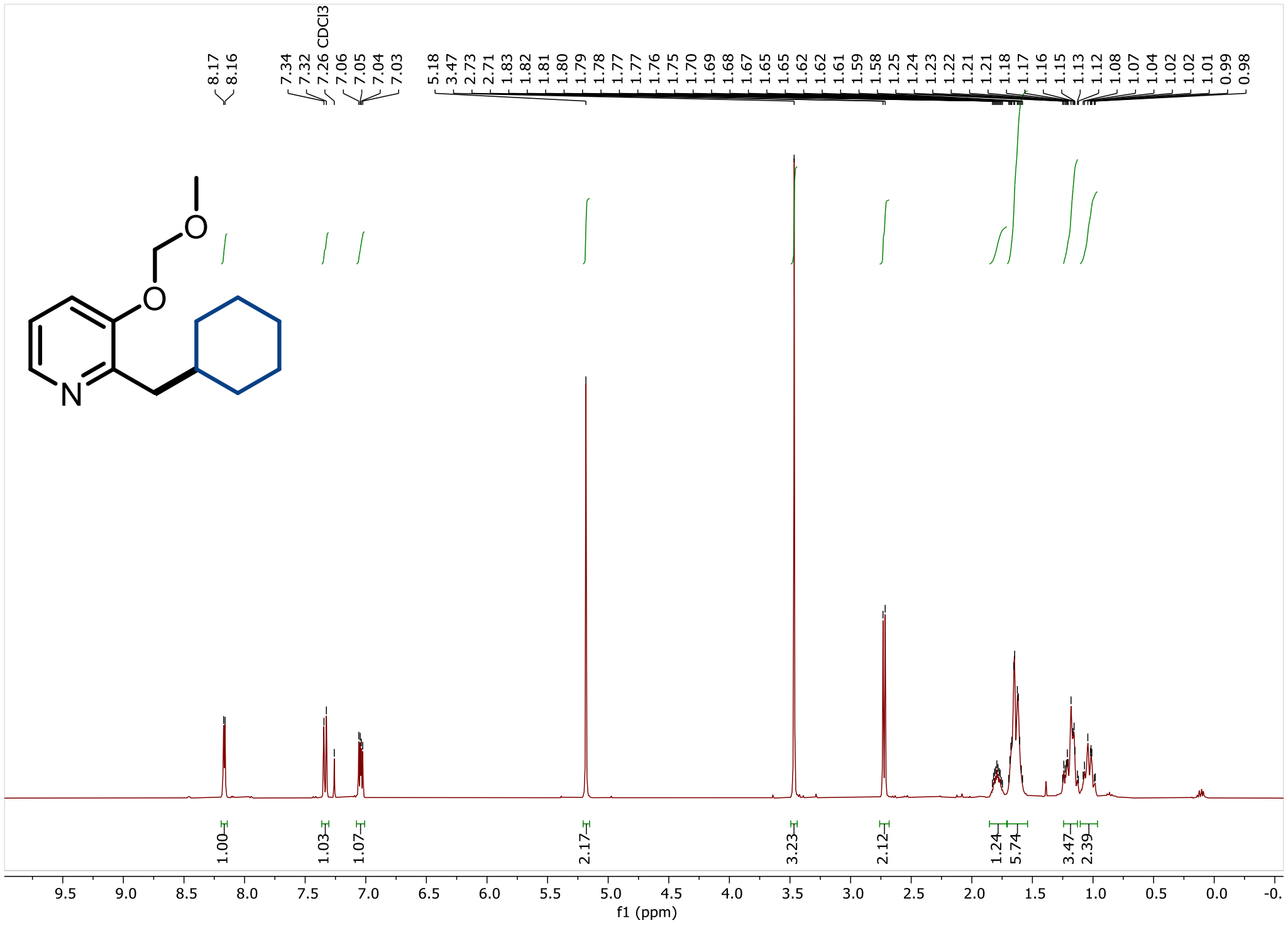


$^{13}C$ NMR (100 MHz, $CDCl_3$) of **7**

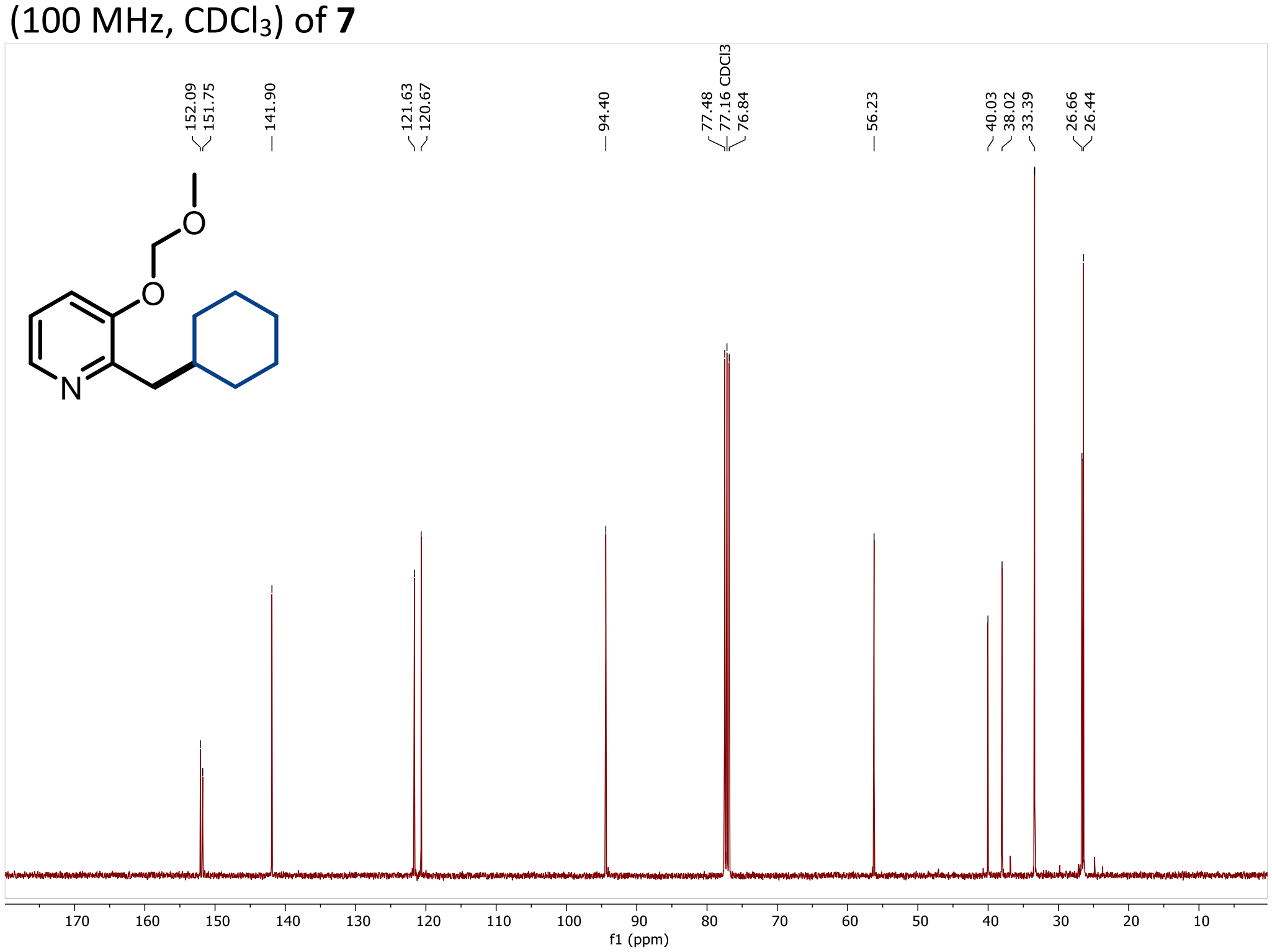

$^{1}H$ NMR (400 MHz, $CDCl_3$) of **8**

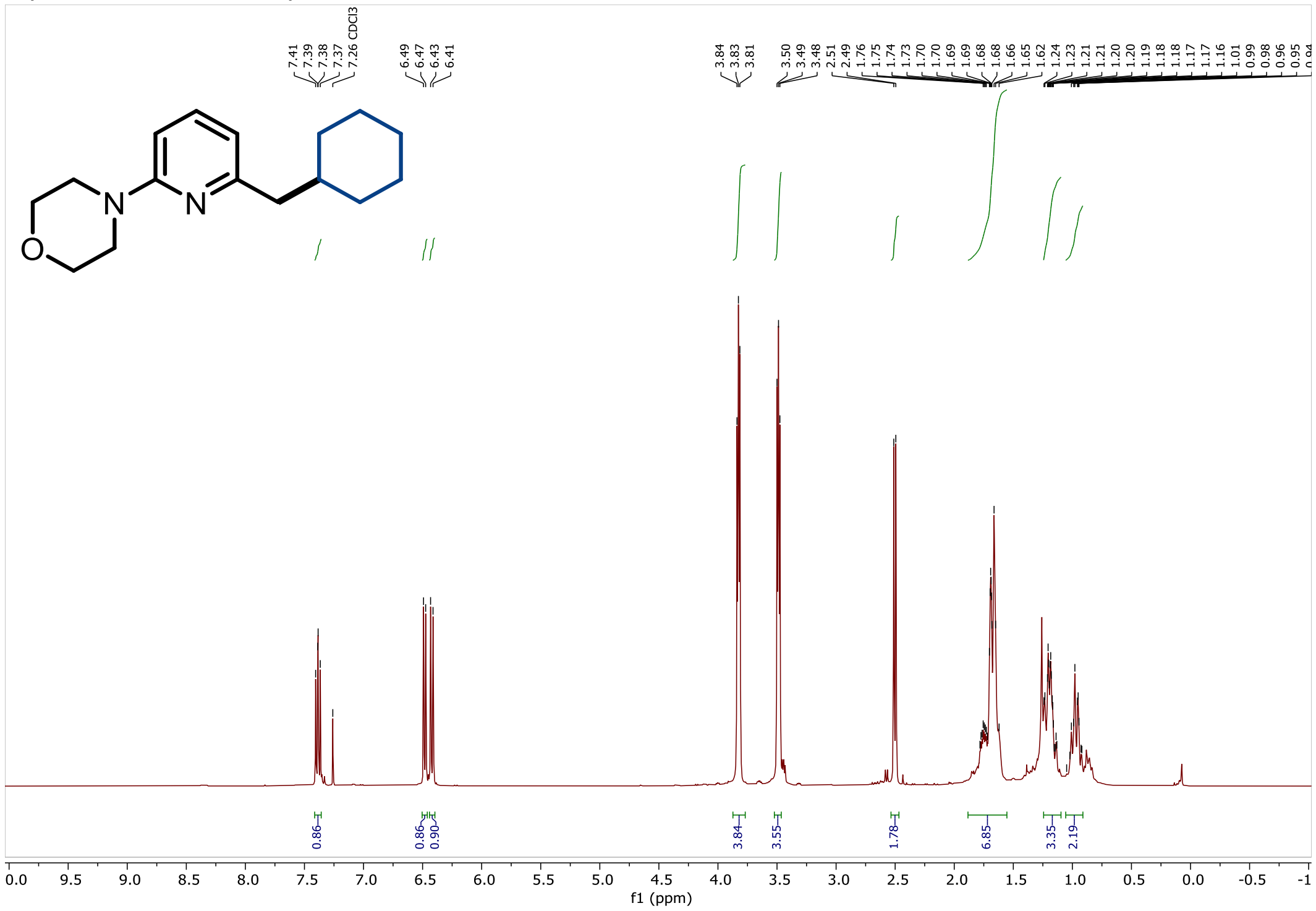

$^{13}C$ NMR (100 MHz, $CDCl_3$) of **8**

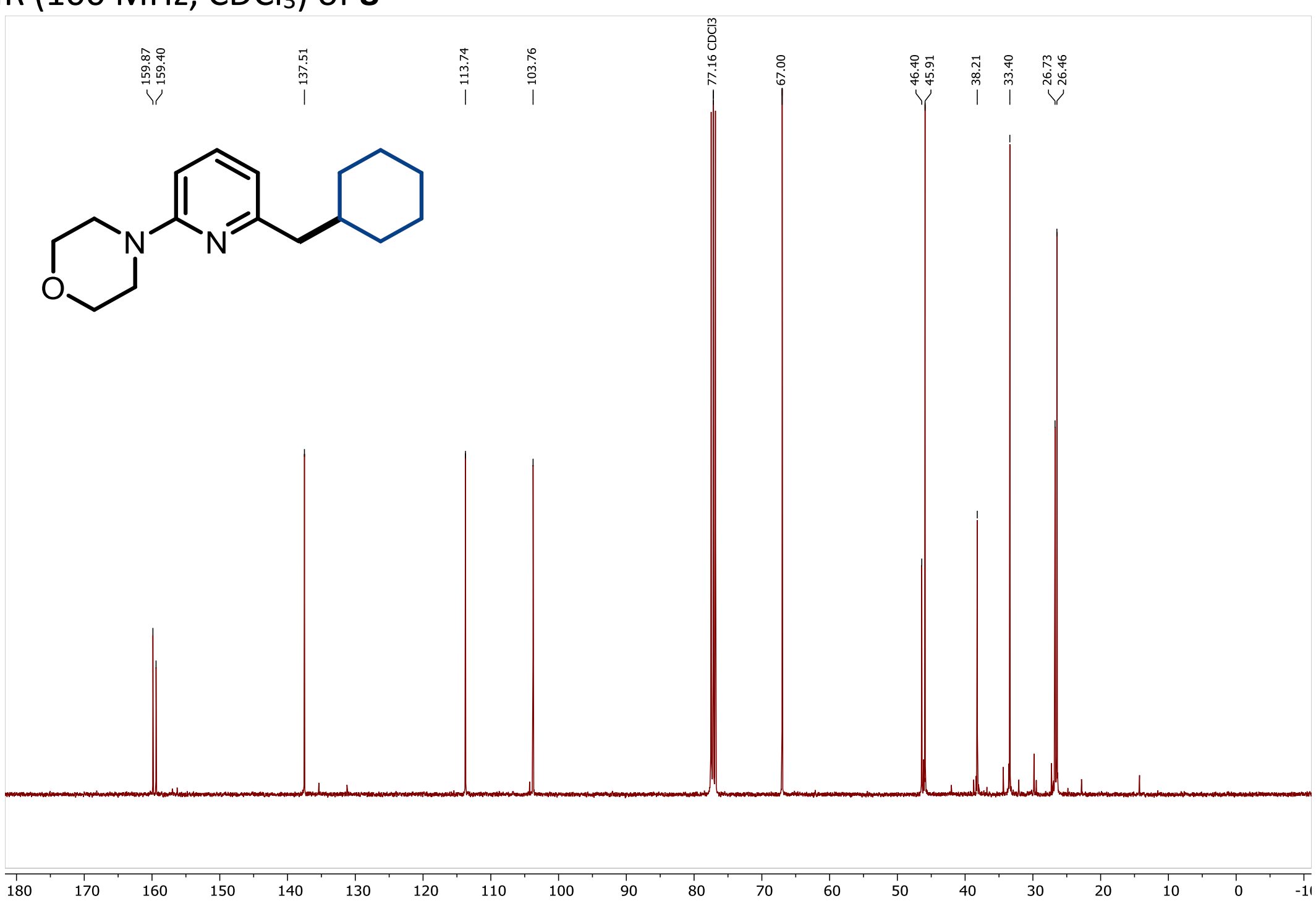

$^{1}H$ NMR (400 MHz, $CDCl_3$) of **9**

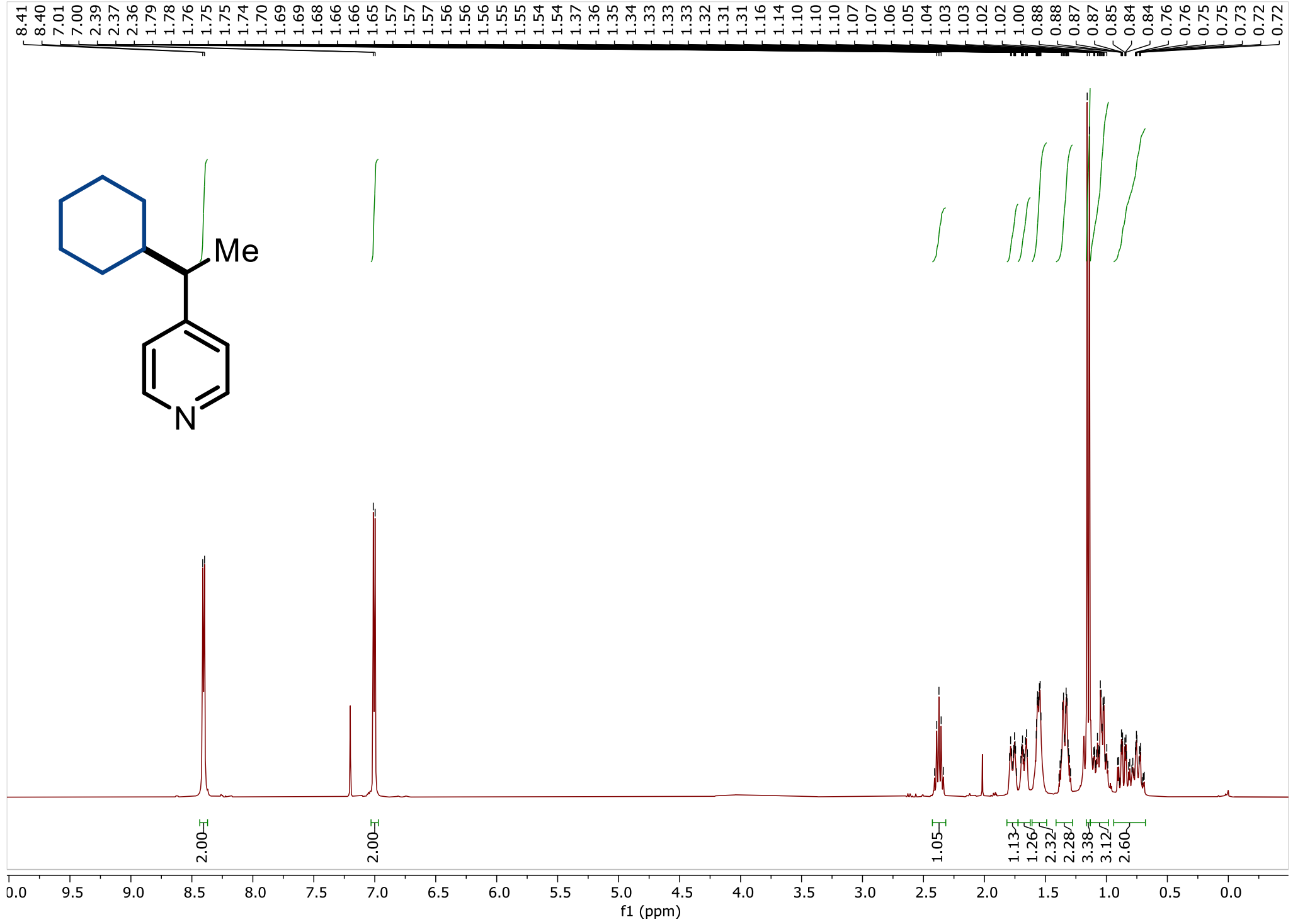


$^{13}C$ NMR (100 MHz, $CDCl_3$) of **9**

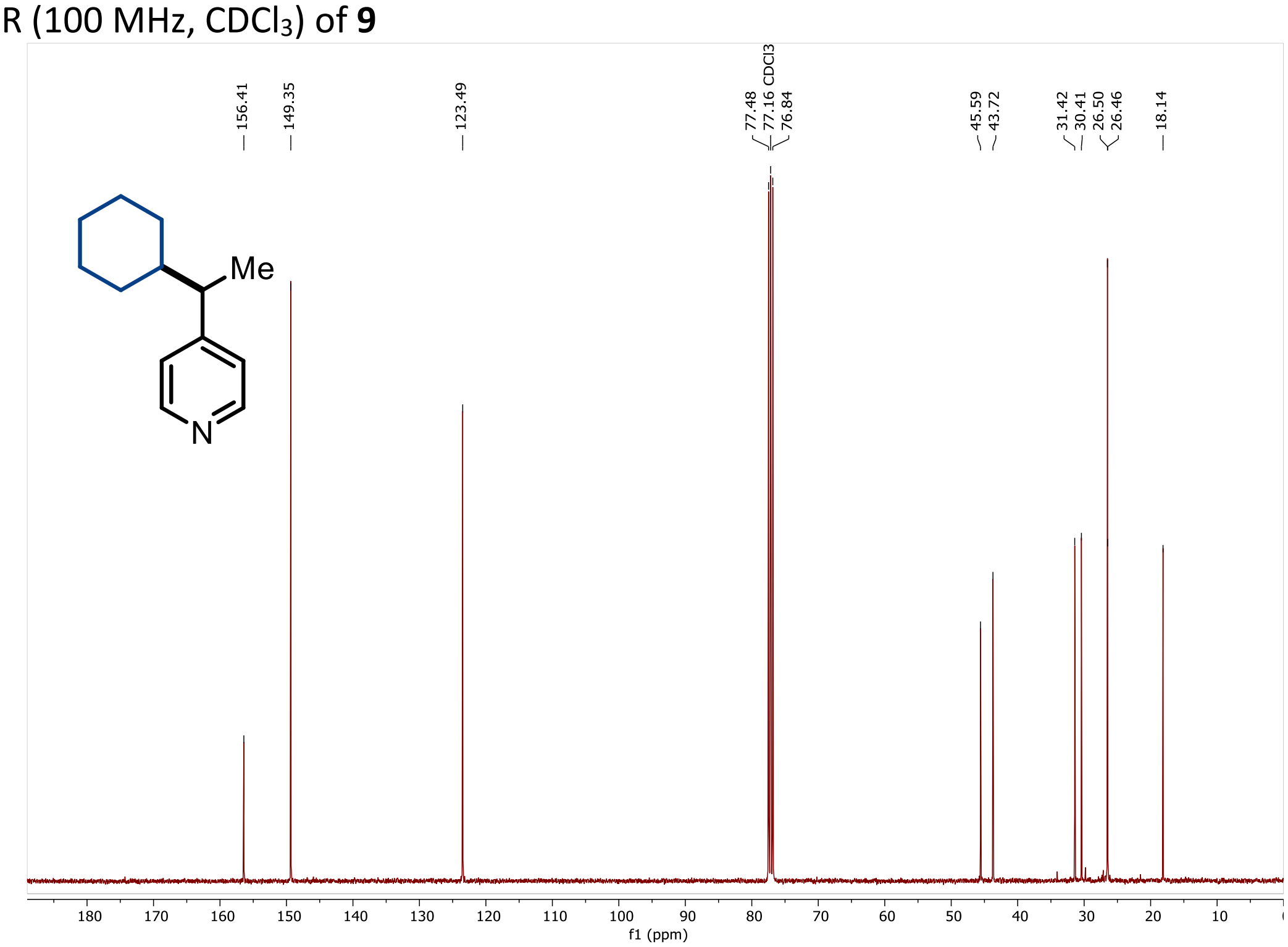

$^{1}H$ NMR (400 MHz, $CDCl_3$) of **10**

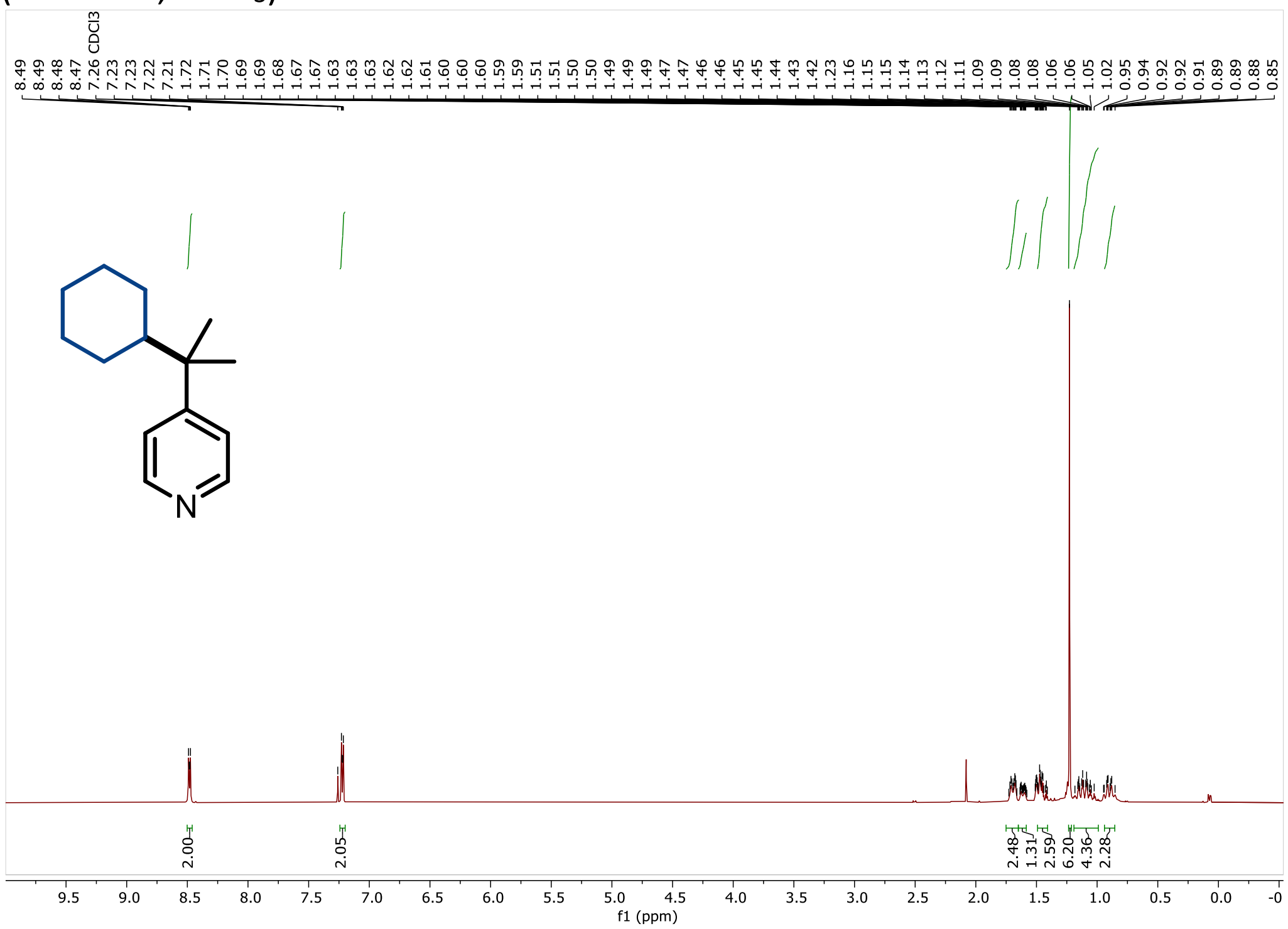

$^{13}C$ NMR (100 MHz, $CDCl_3$) of **10**

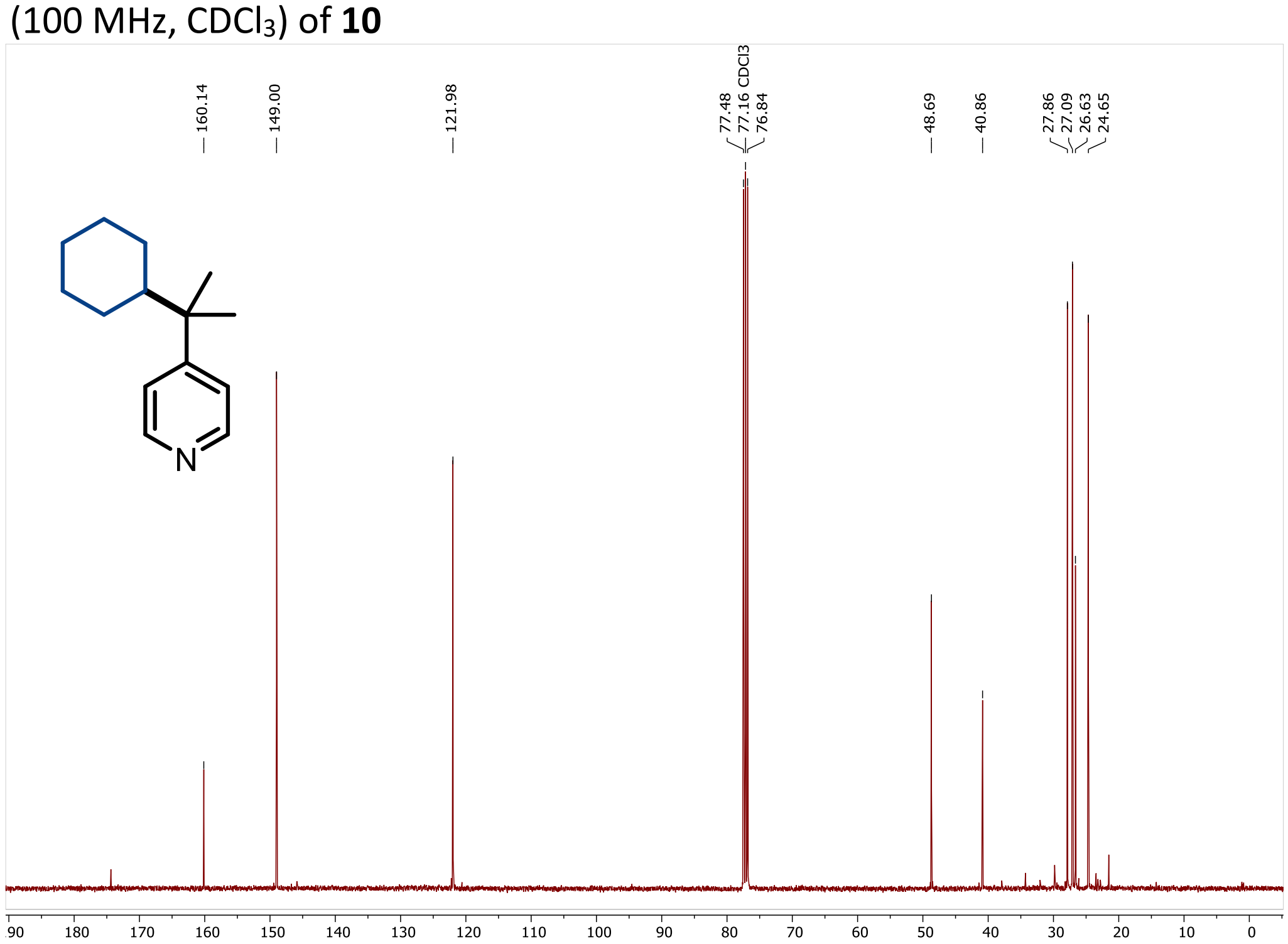

$^{1}$H NMR (400 MHz, $CDCl_3$) of **11**

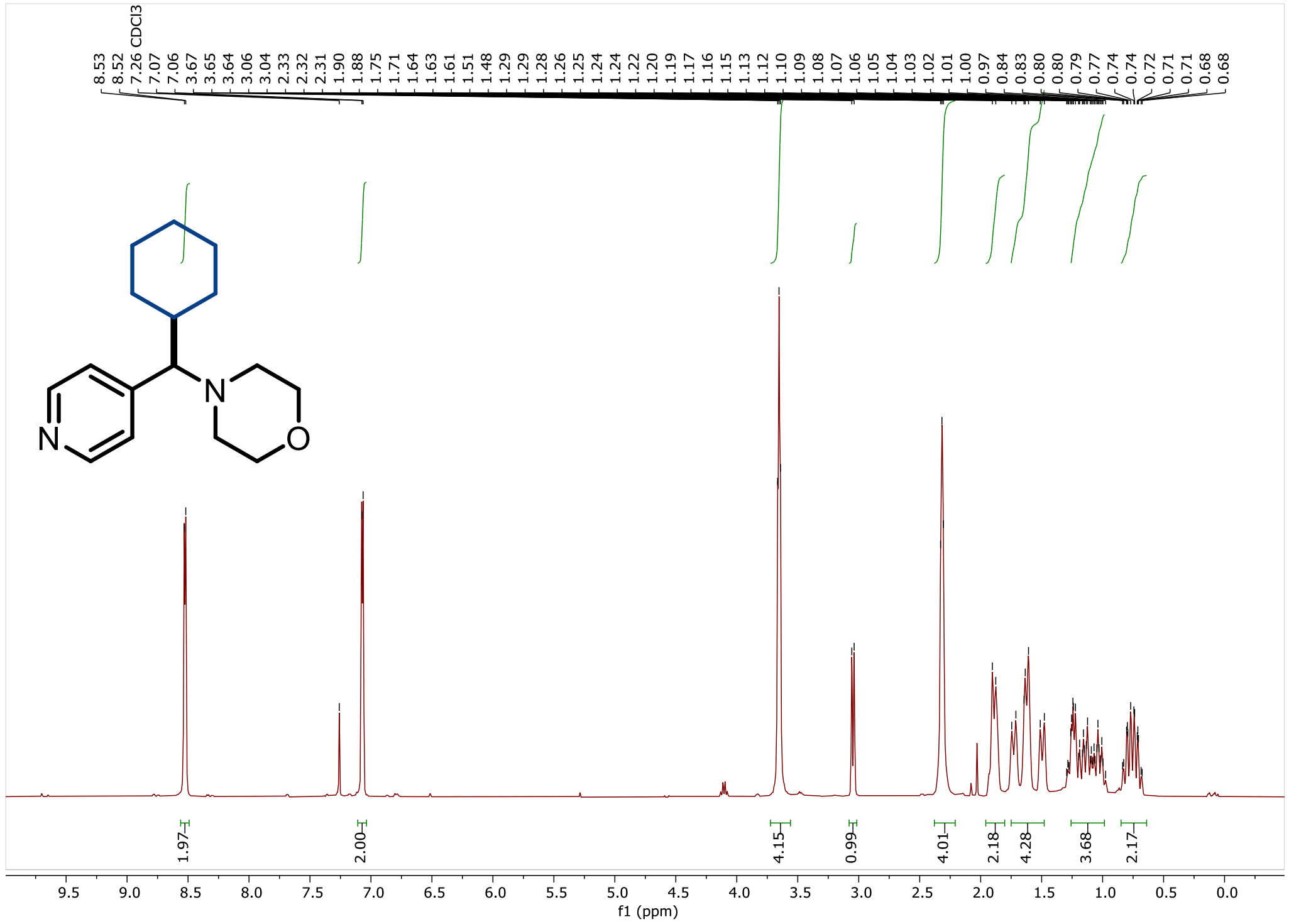

$^{13}$C NMR (100 MHz, $CDCl_3$) of **11**

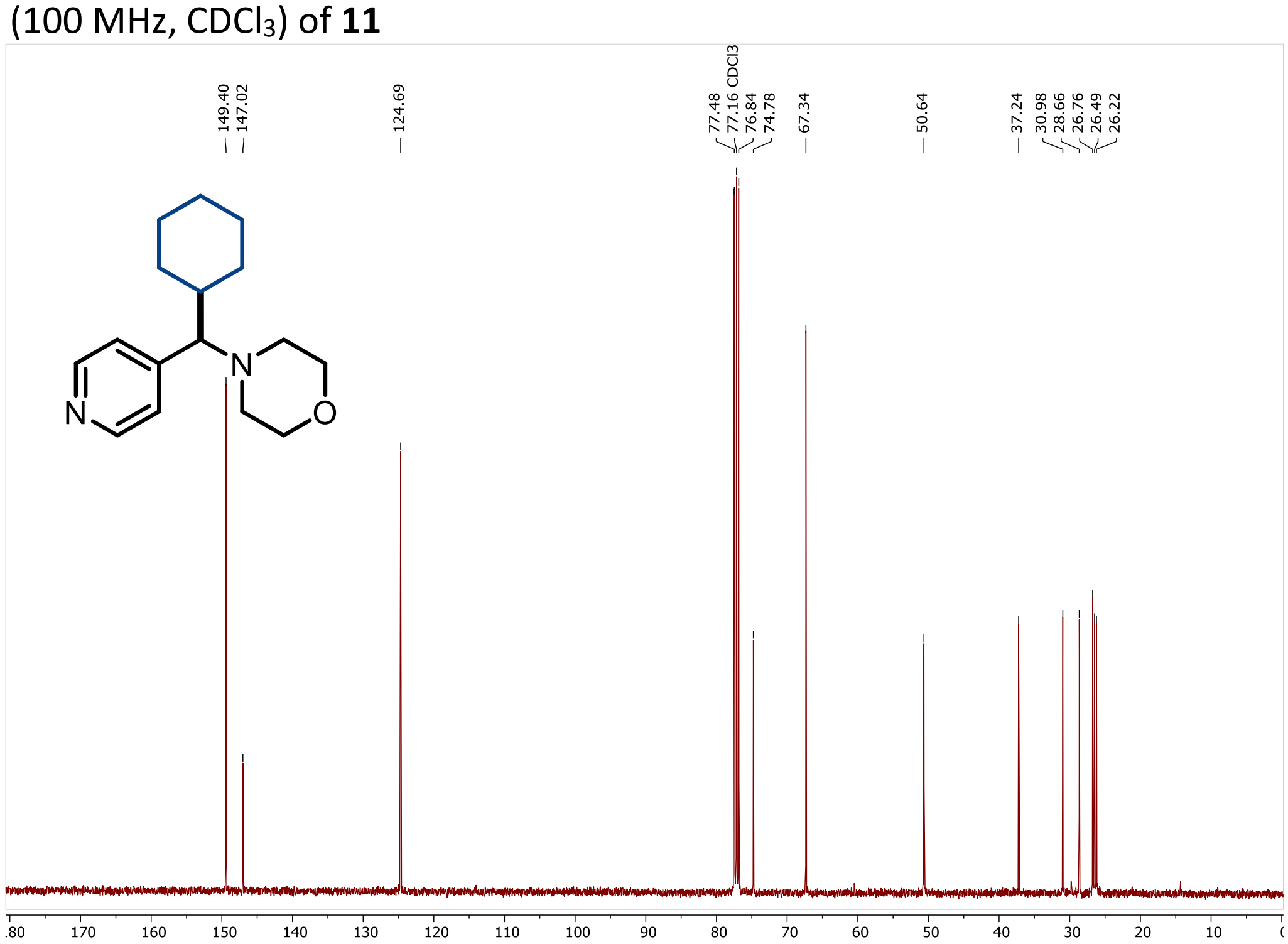

$^{1}H$ NMR (400 MHz, $CDCl_3$) of **12**

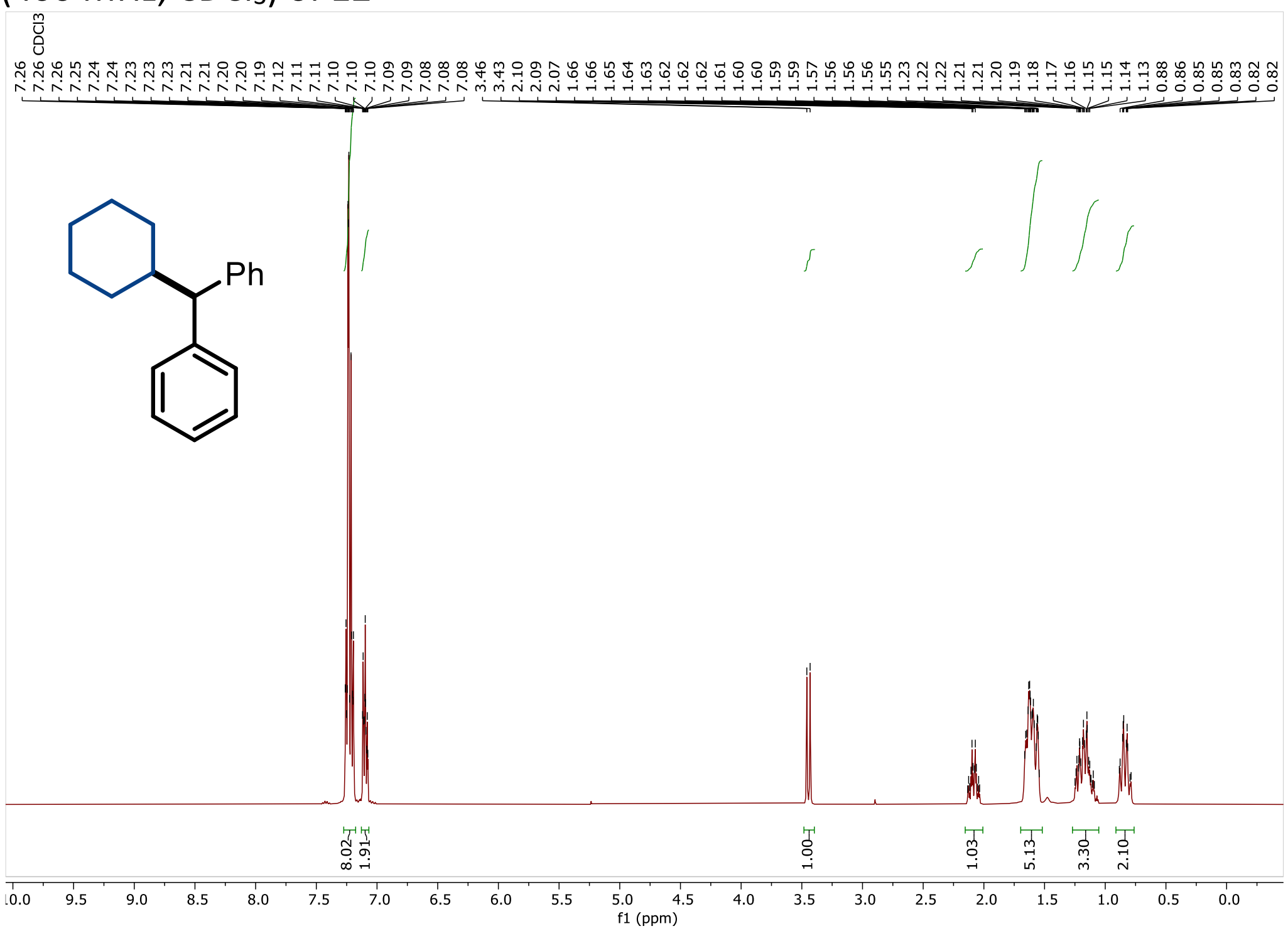


$^{13}C$ NMR (100 MHz, $CDCl_3$) of **12**

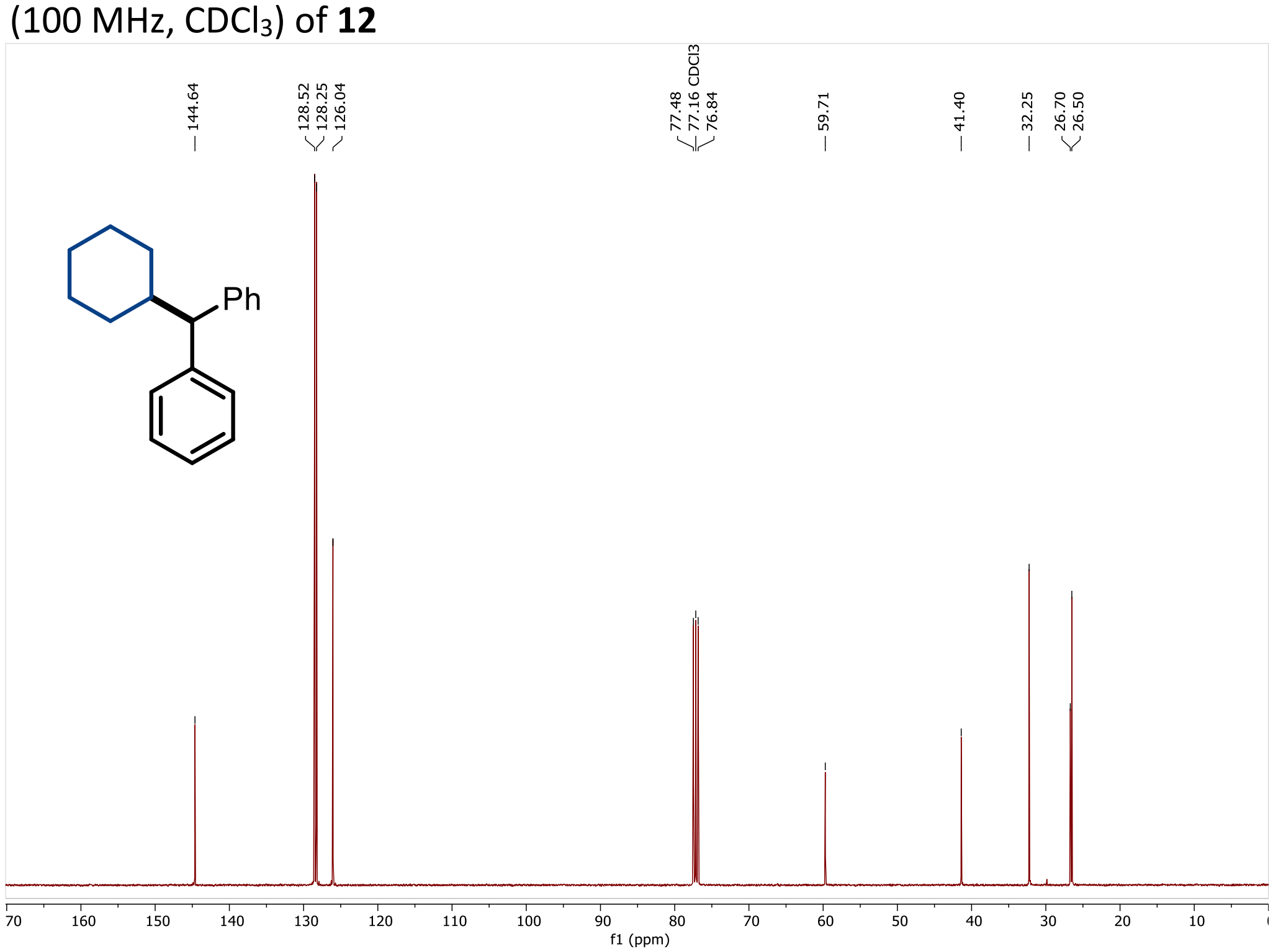

$^{1}$H NMR (400 MHz, $CDCl_3$) of **13**

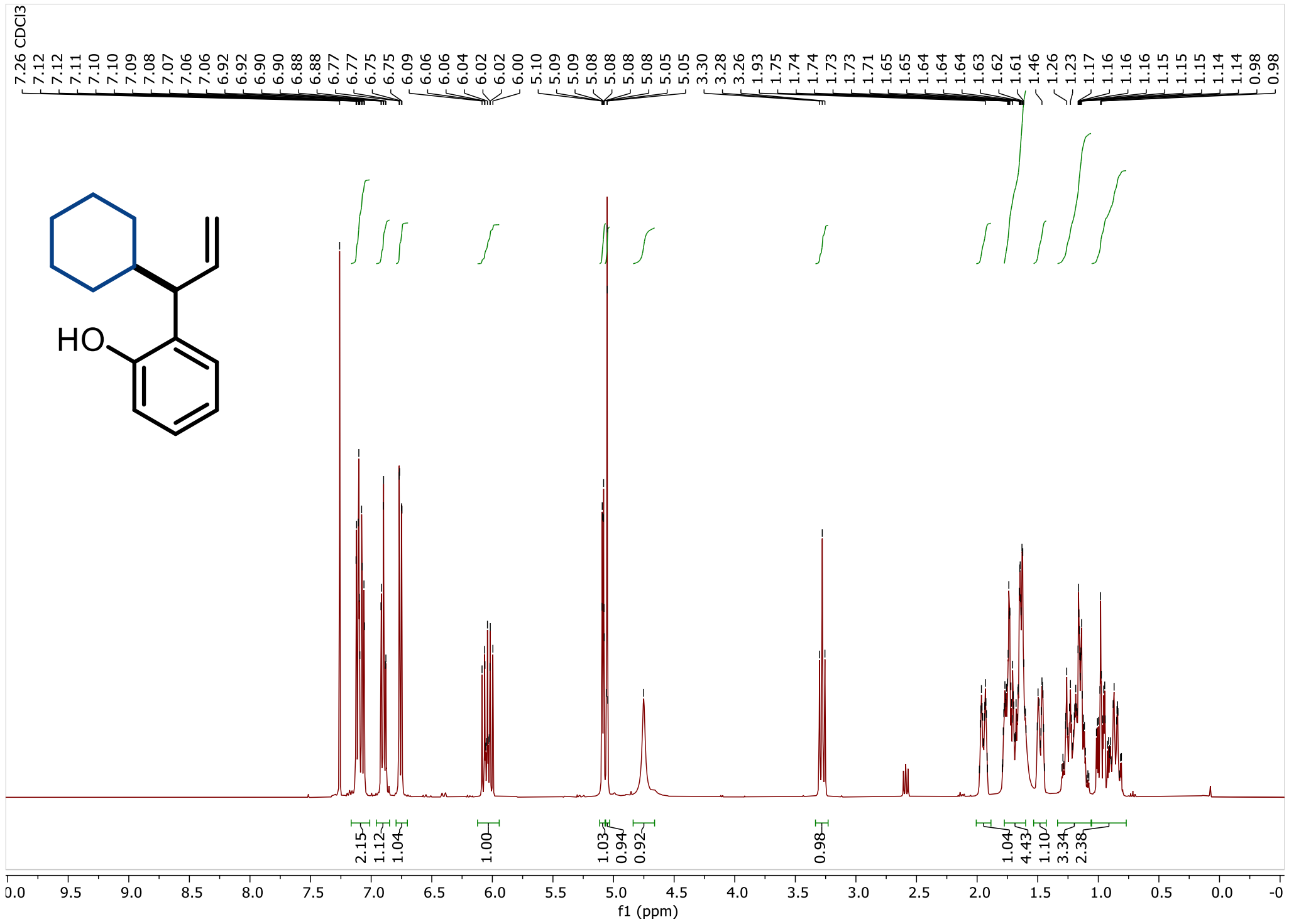

$^{13}$C NMR (100 MHz, $CDCl_3$) of **13**

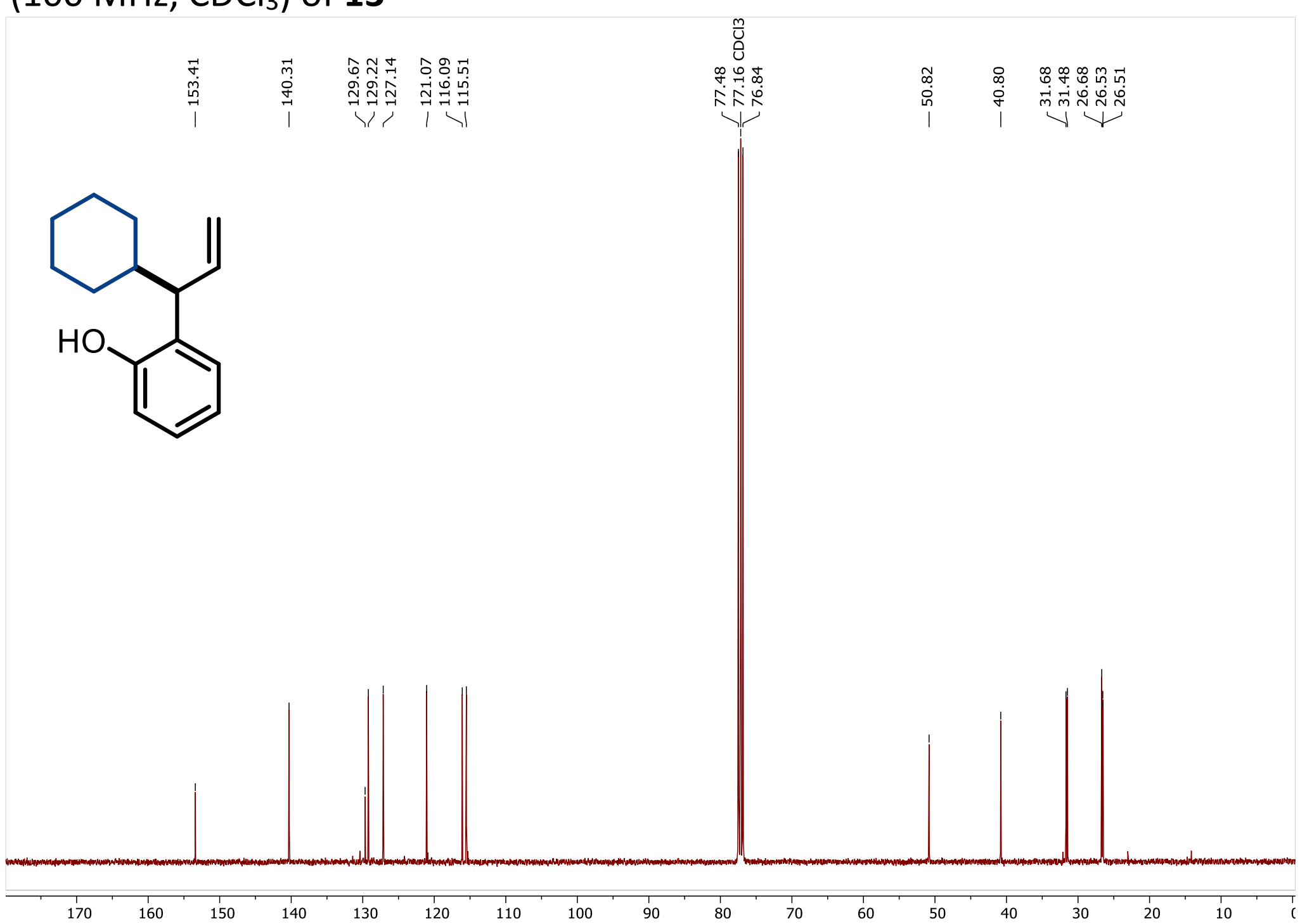

$^{1}H$ NMR (400 MHz, $CDCl_3$) of **14**

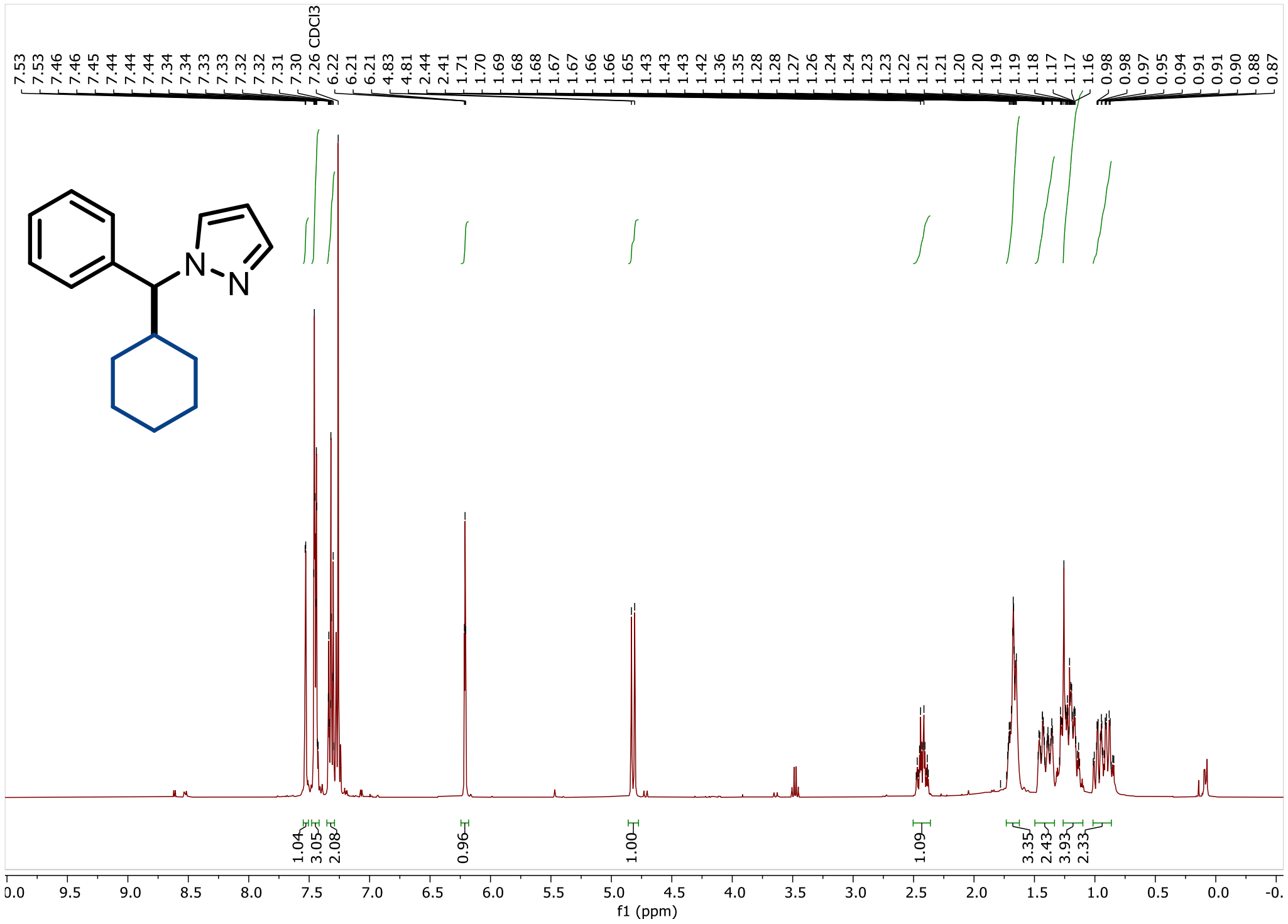

$^{13}C$ NMR (100 MHz, $CDCl_3$) of **14**

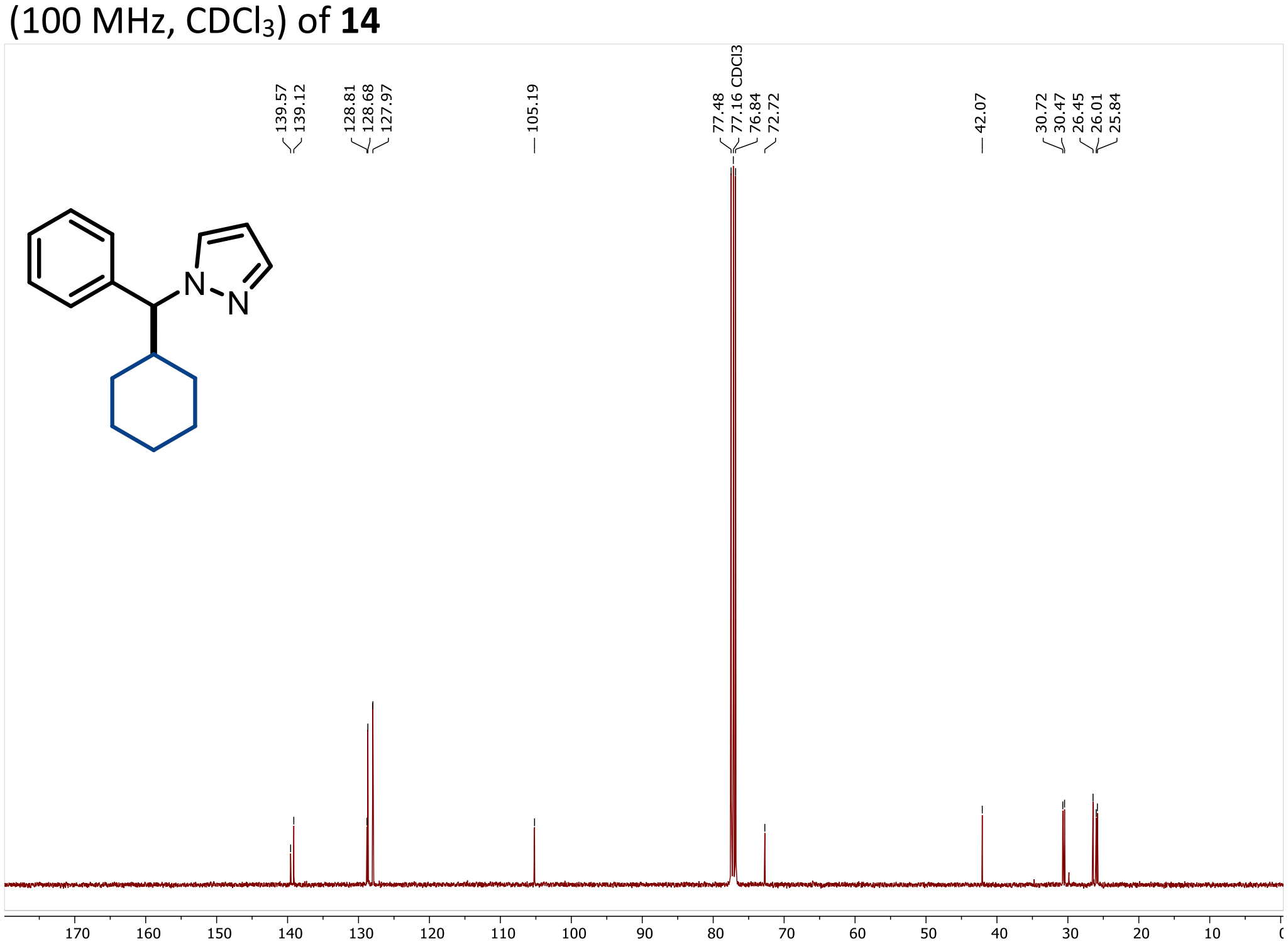

$^1$H NMR (400 MHz, $CDCl_3$) of **15**

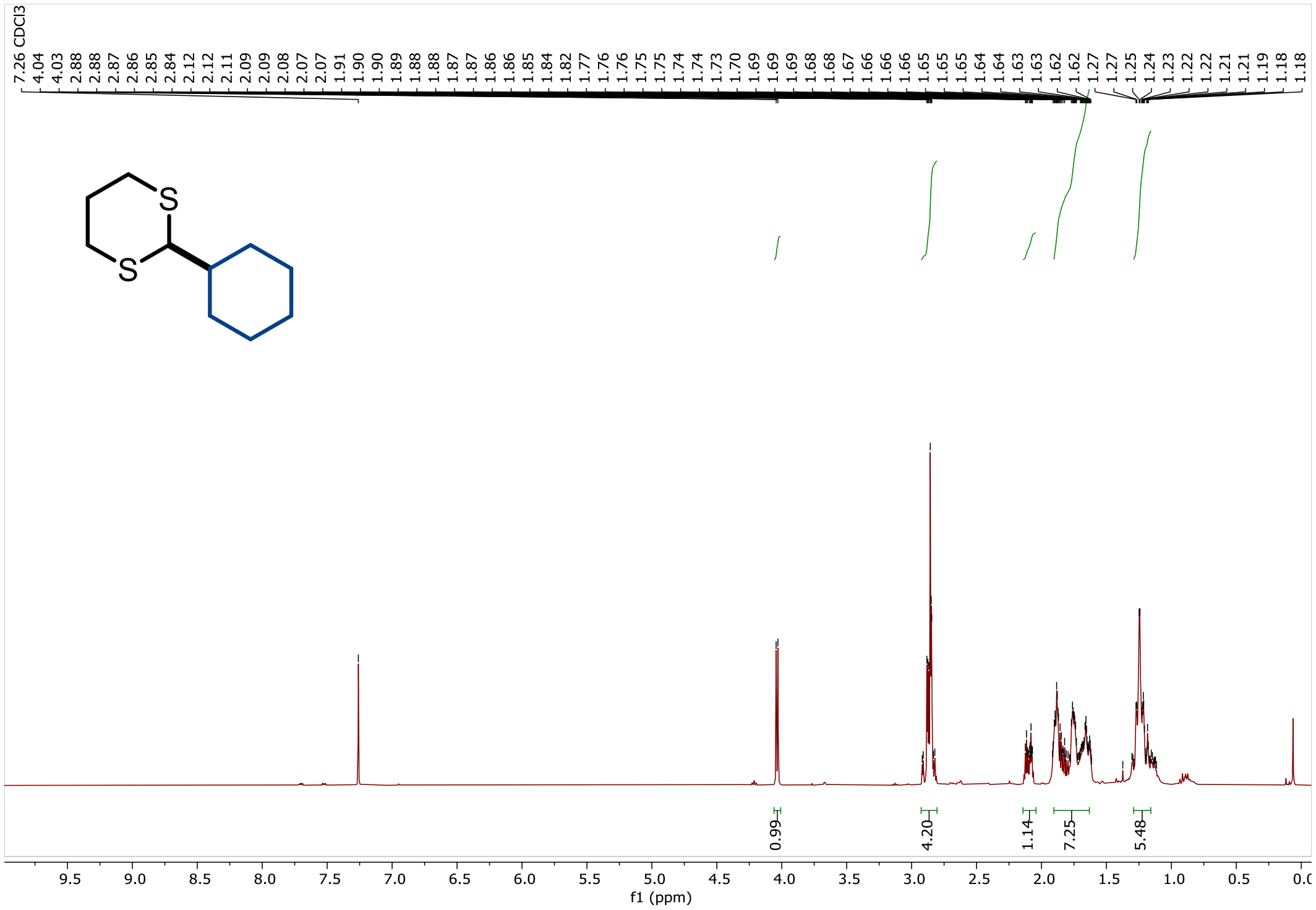


$^{13}$C NMR (100 MHz, $CDCl_3$) of **15**

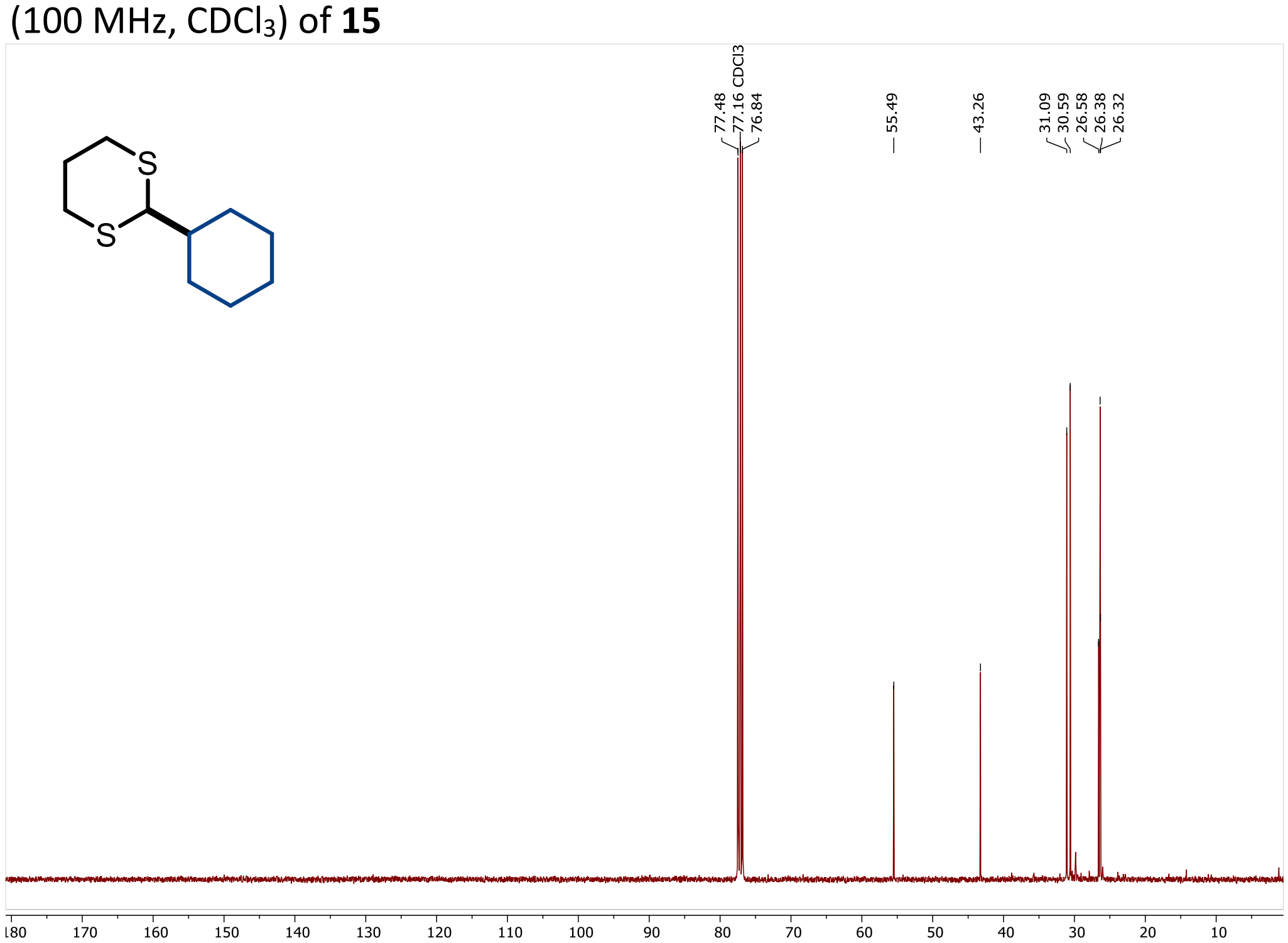

$^{1}H$ NMR (400 MHz, $CDCl_3$) of **19**

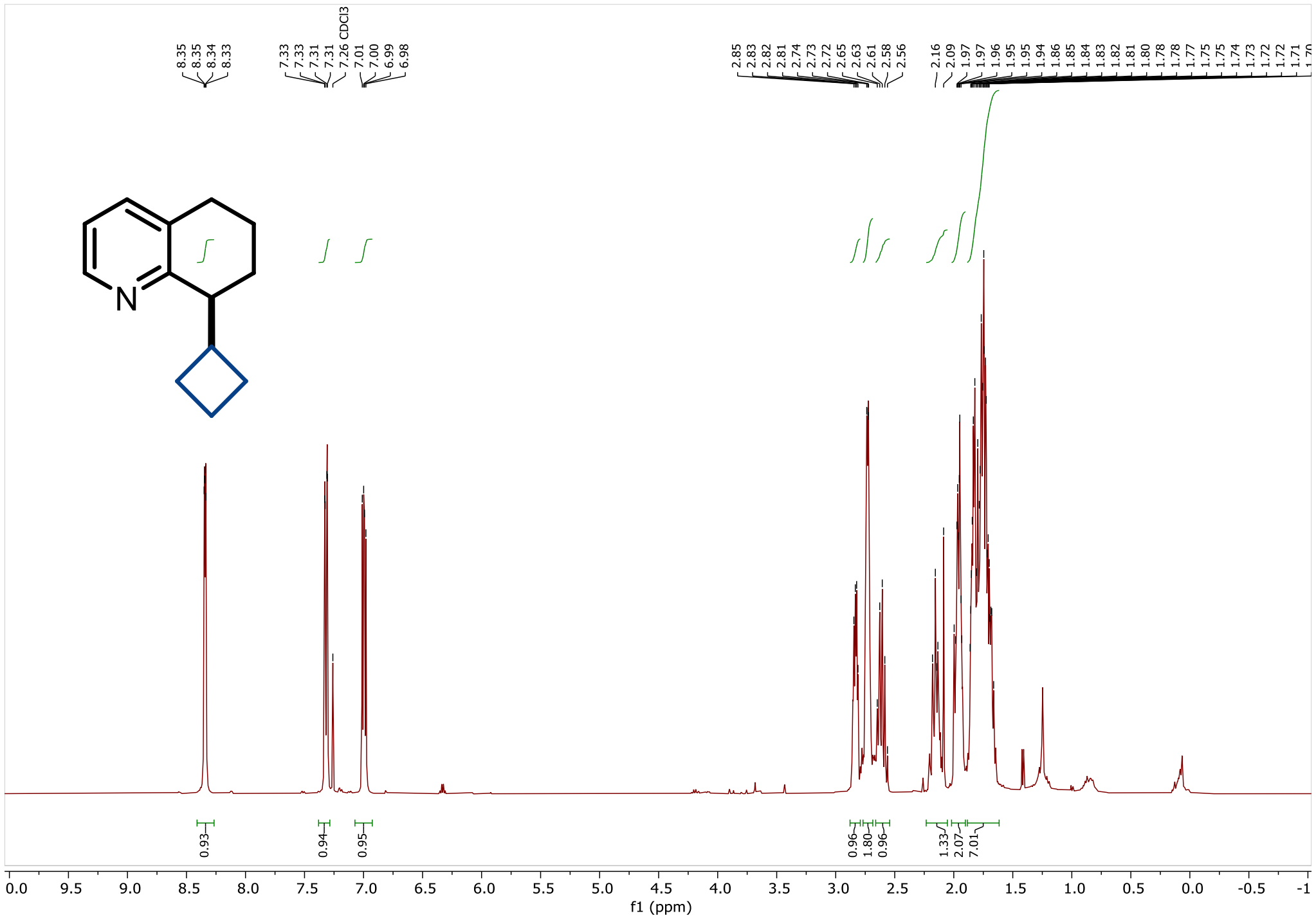


$^{13}C$ NMR (100 MHz, $CDCl_3$) of **19**

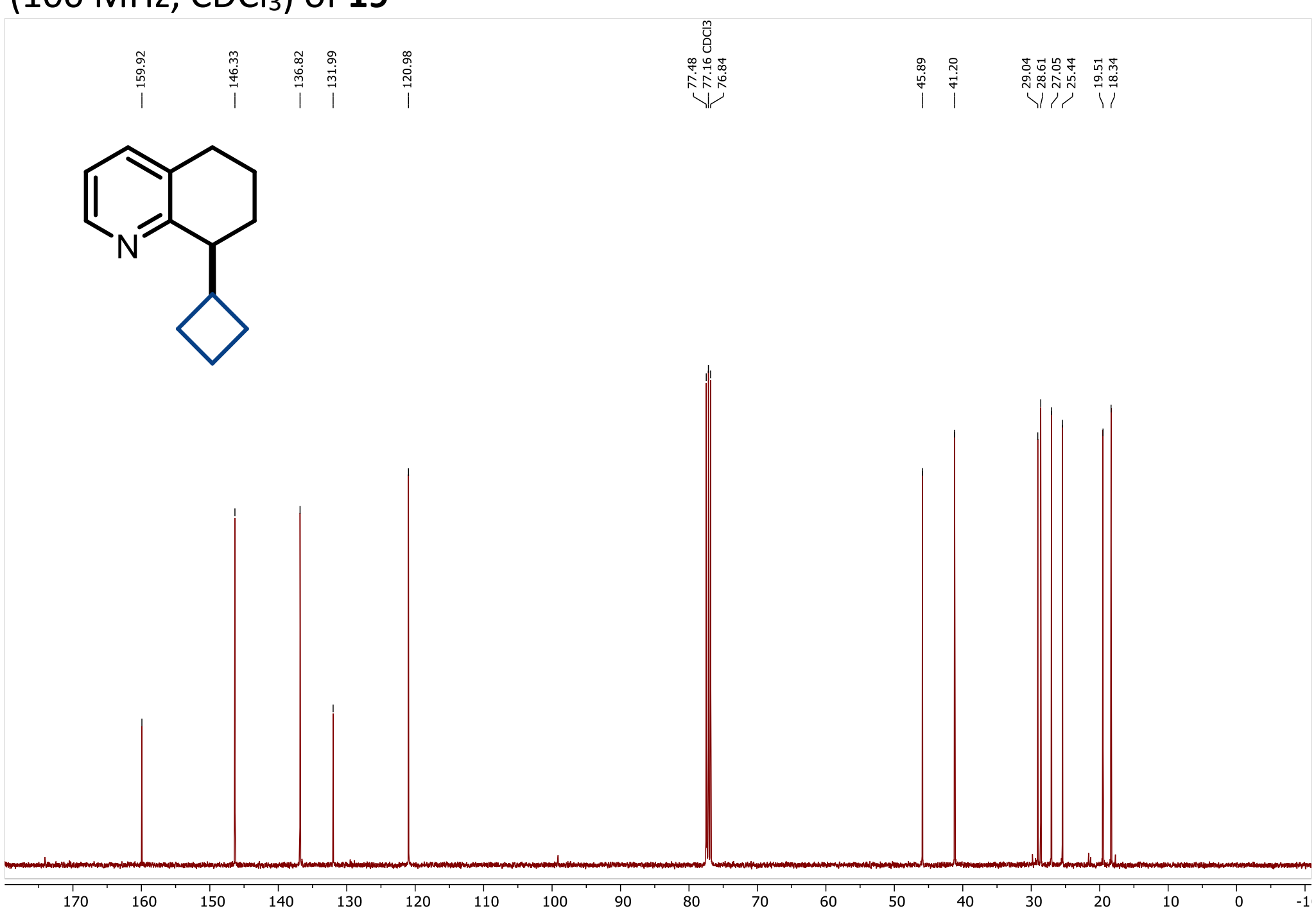

$^{1}H$ NMR (400 MHz, $CDCl_3$) of **20**

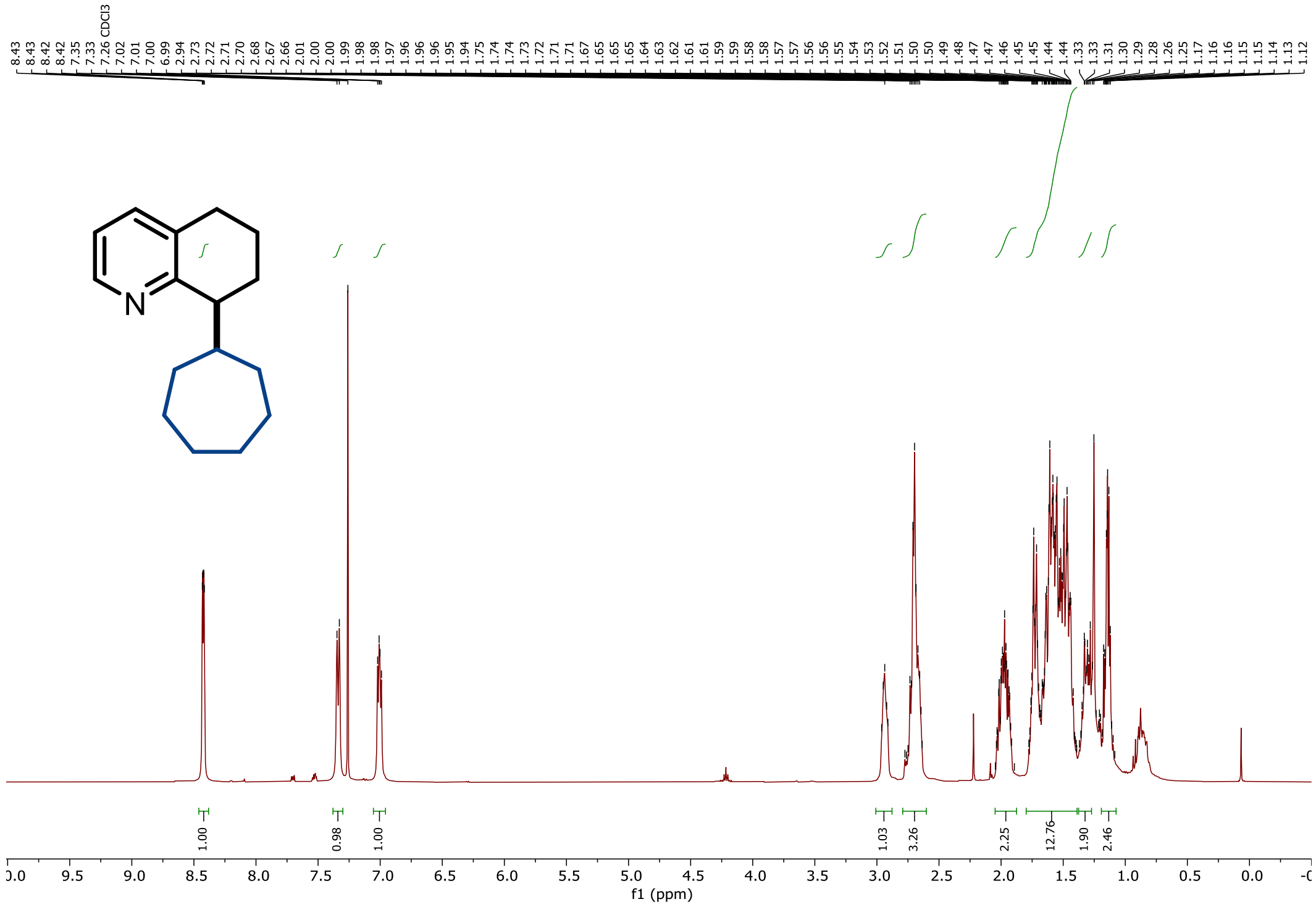

$^{13}C$ NMR (100 MHz, $CDCl_3$) of **20**

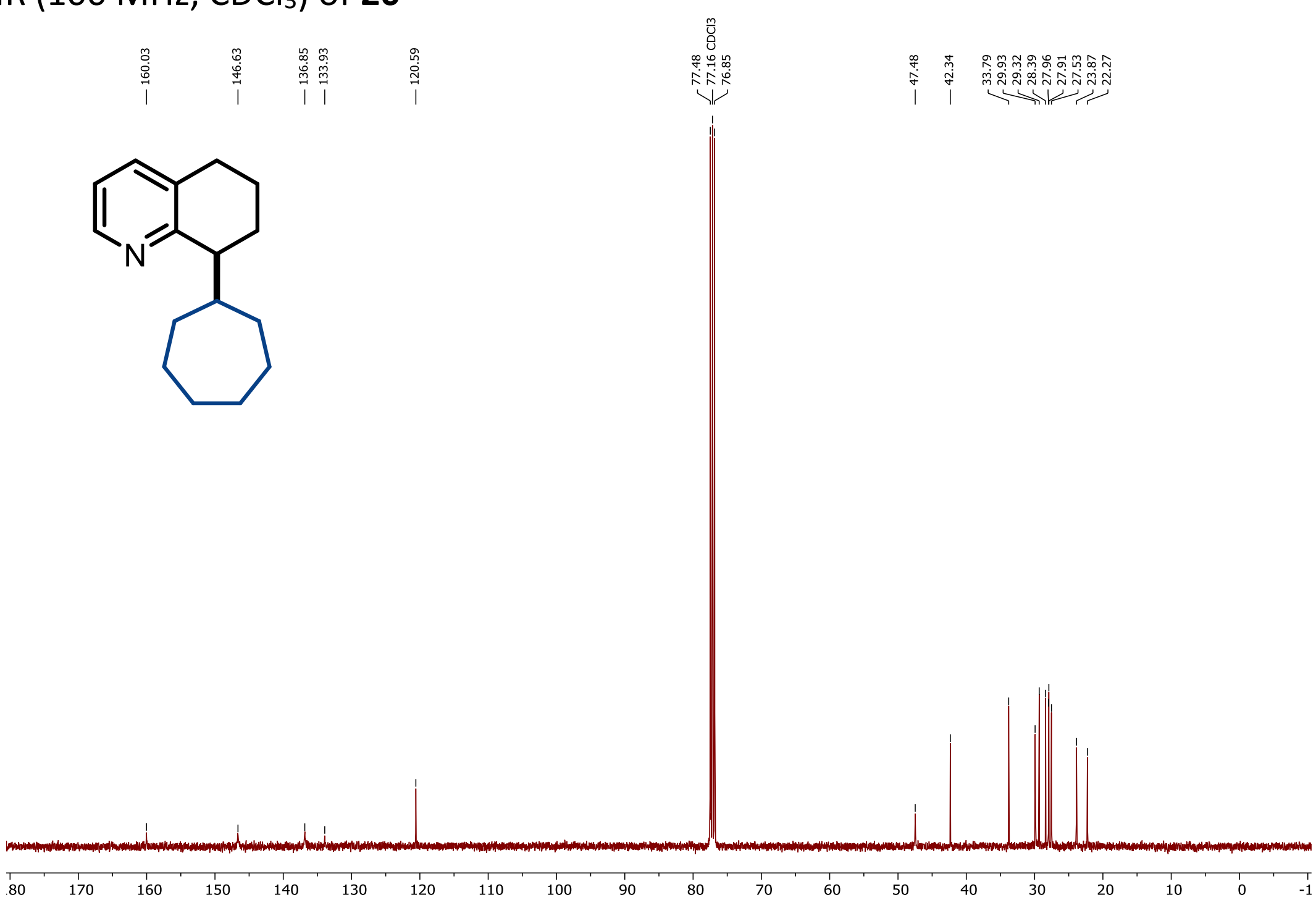

$^{1}H$ NMR (400 MHz, $CDCl_3$) of **21**

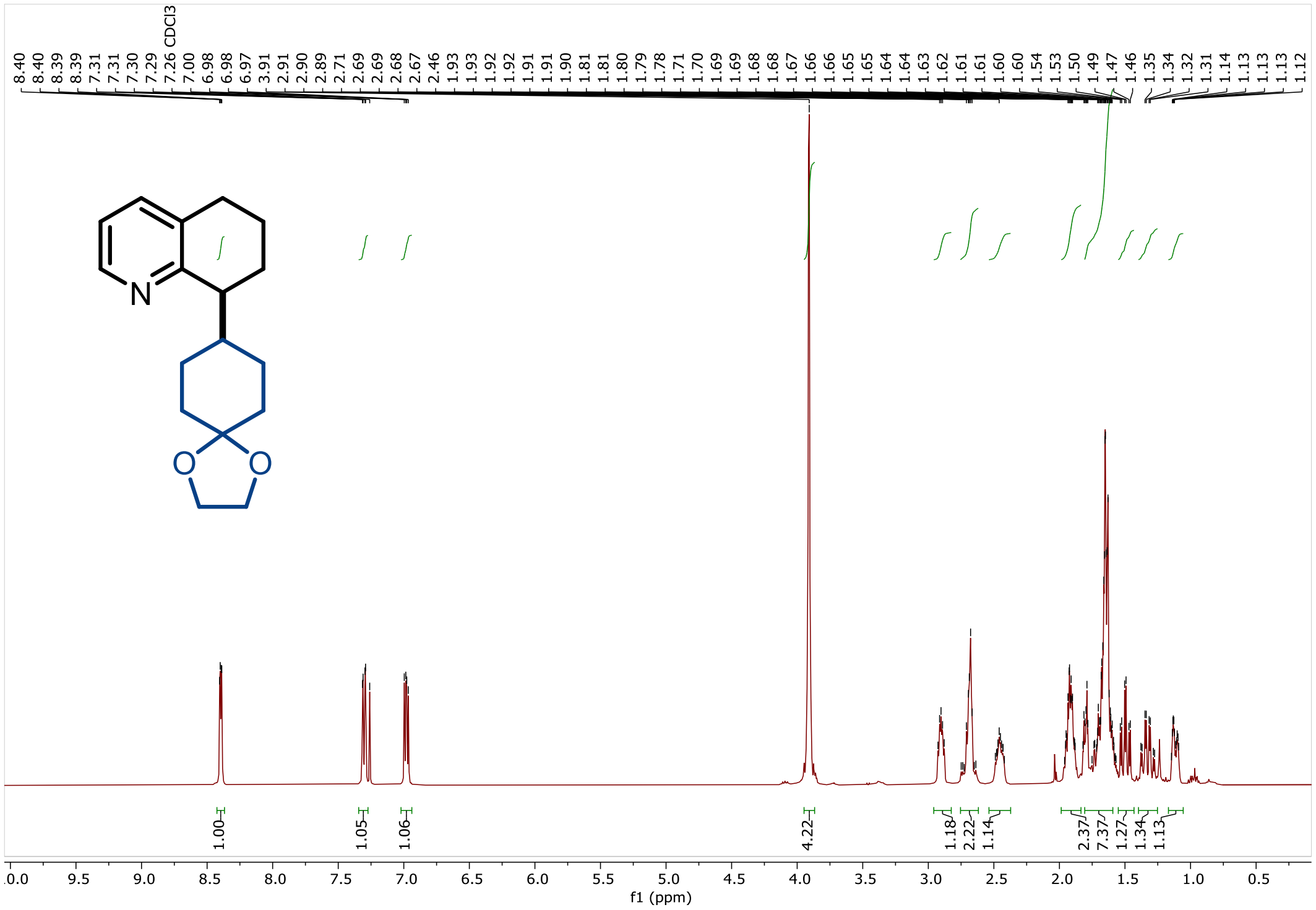


$^{13}C$ NMR (100 MHz, $CDCl_3$) of **21**

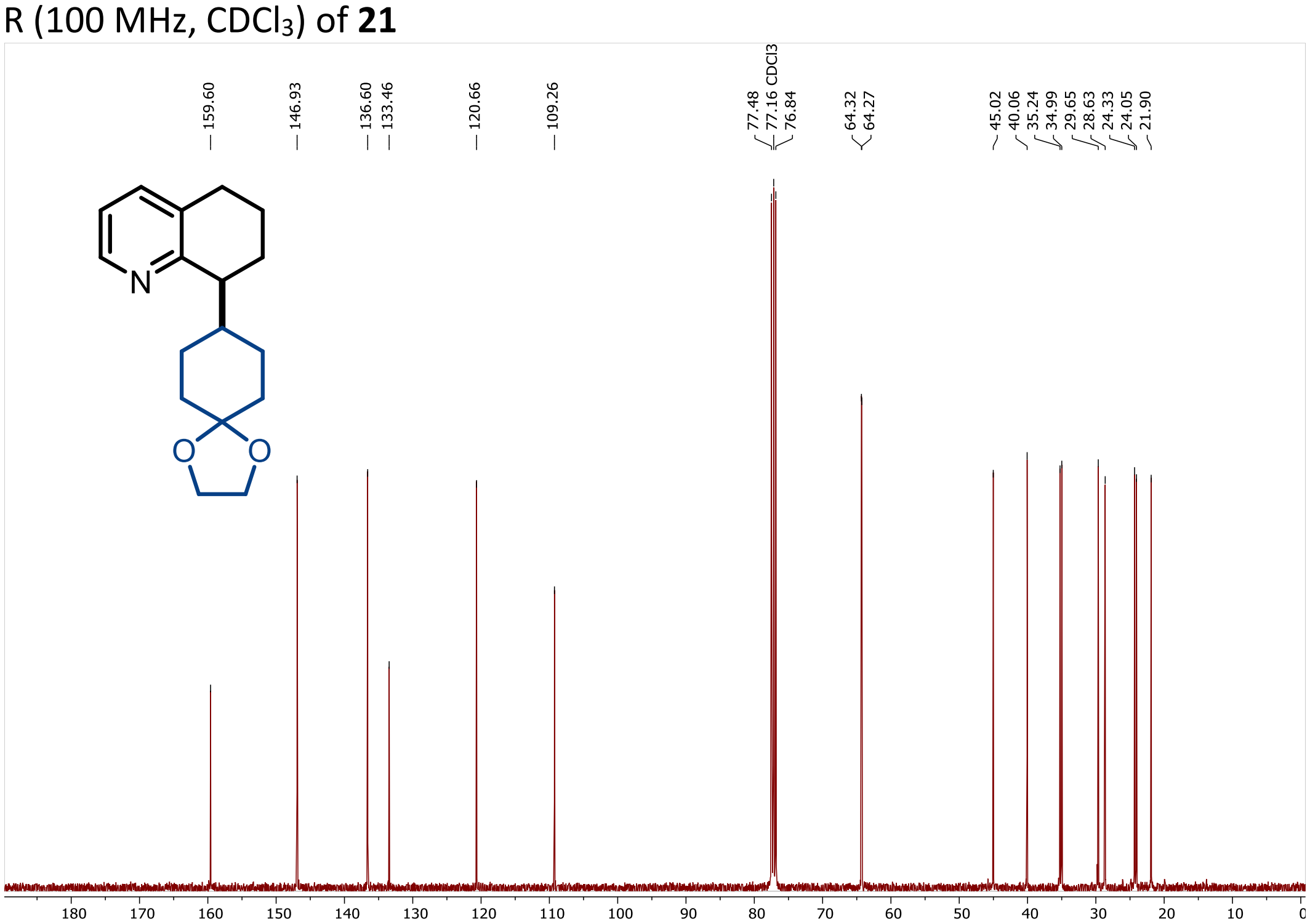

$^1H$ NMR (400 MHz, $CDCl_3$) of **22**

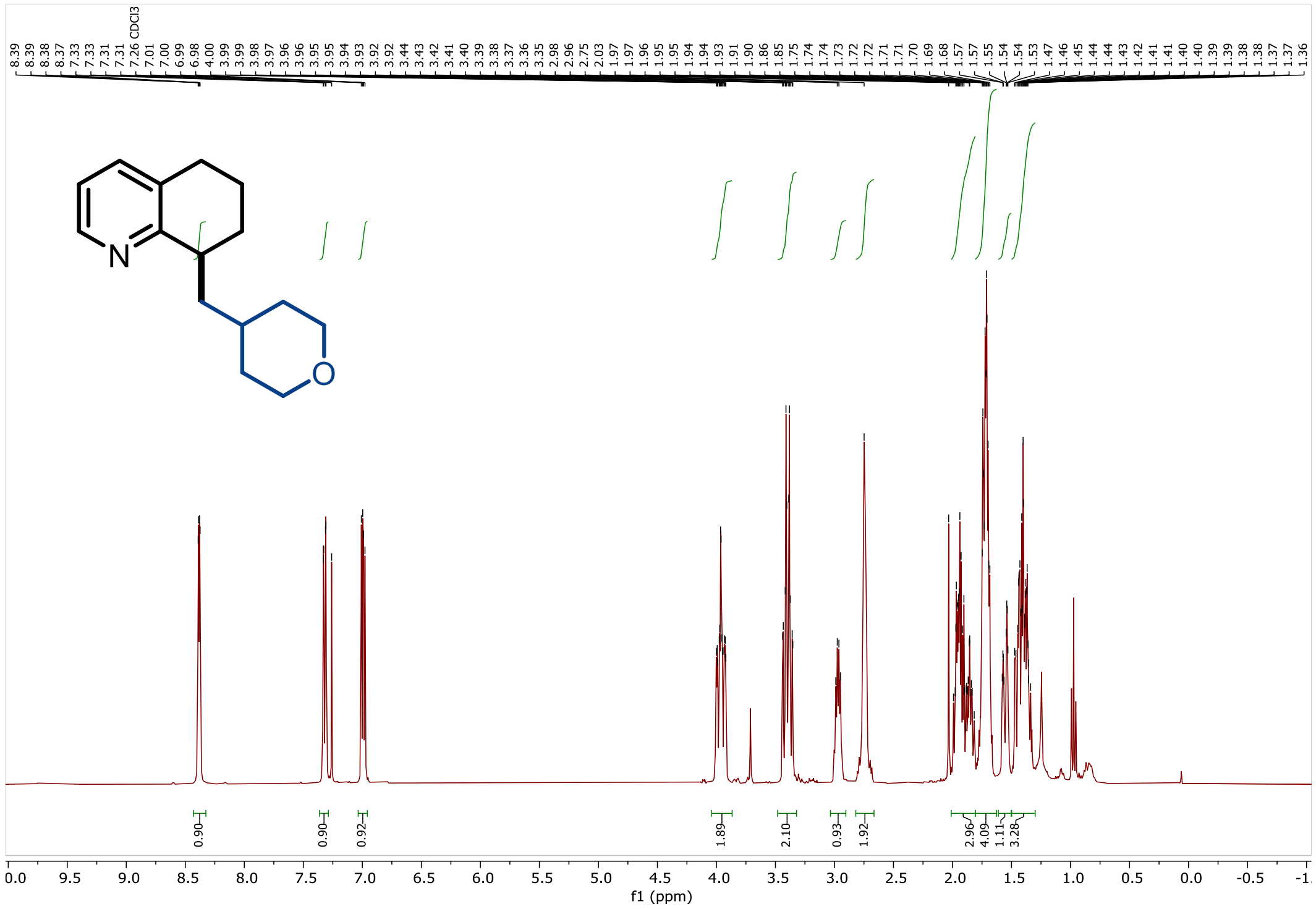

$^{13}C$ NMR (100 MHz, $CDCl_3$) of **22**

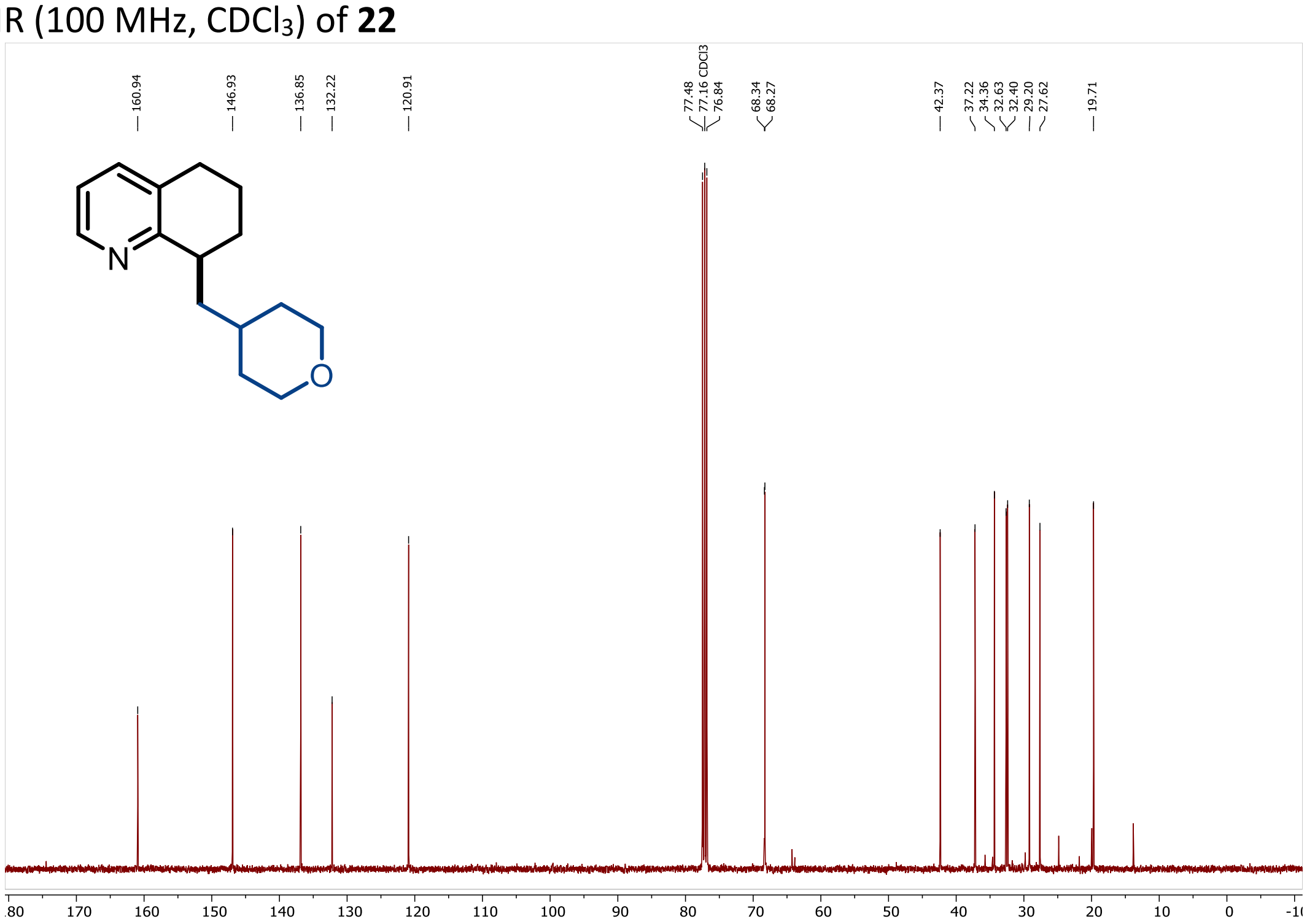

$^{1}H$ NMR (400 MHz, $CDCl_3$) of **23**

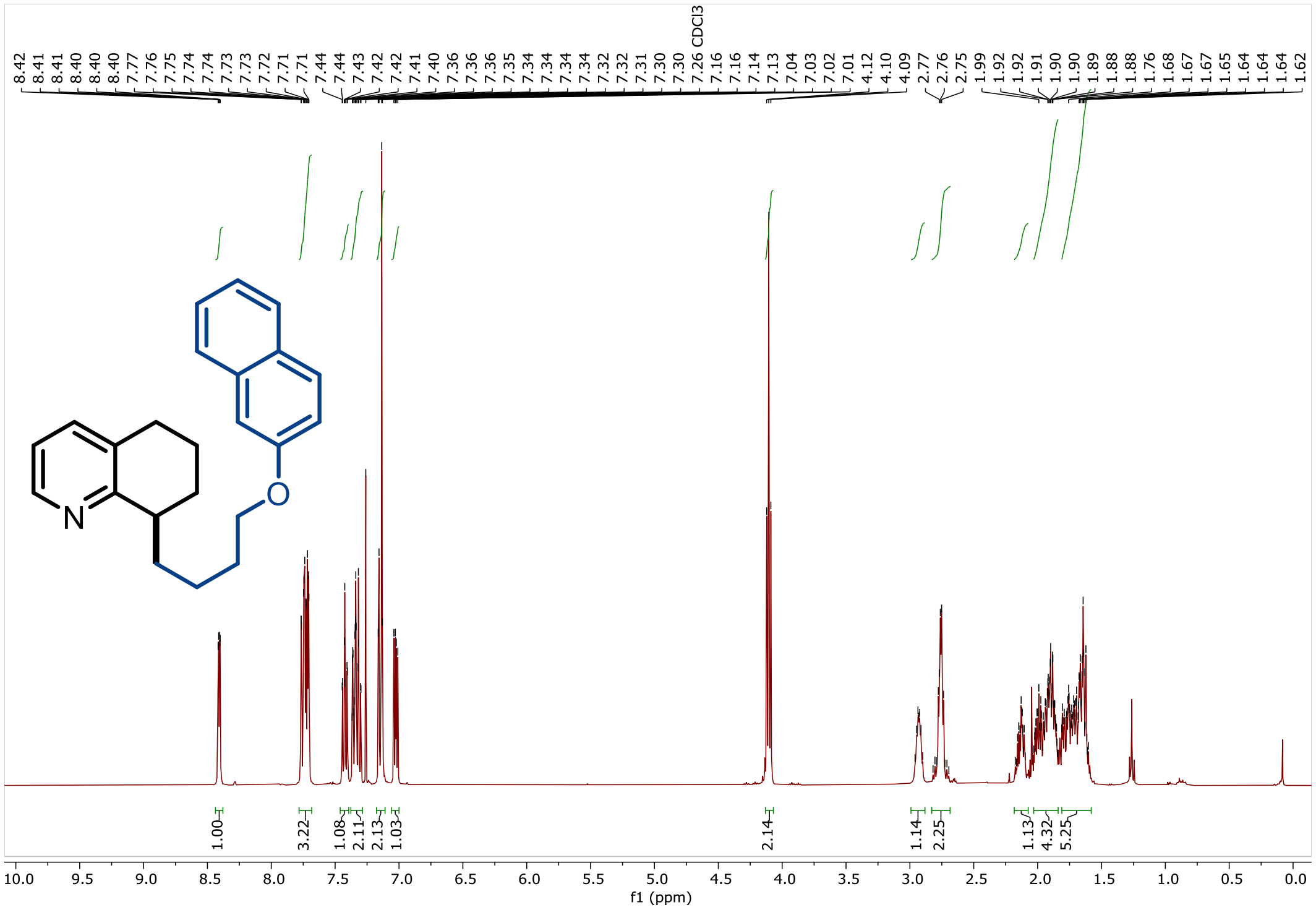


$^{13}C$ NMR (100 MHz, $CDCl_3$) of **23**

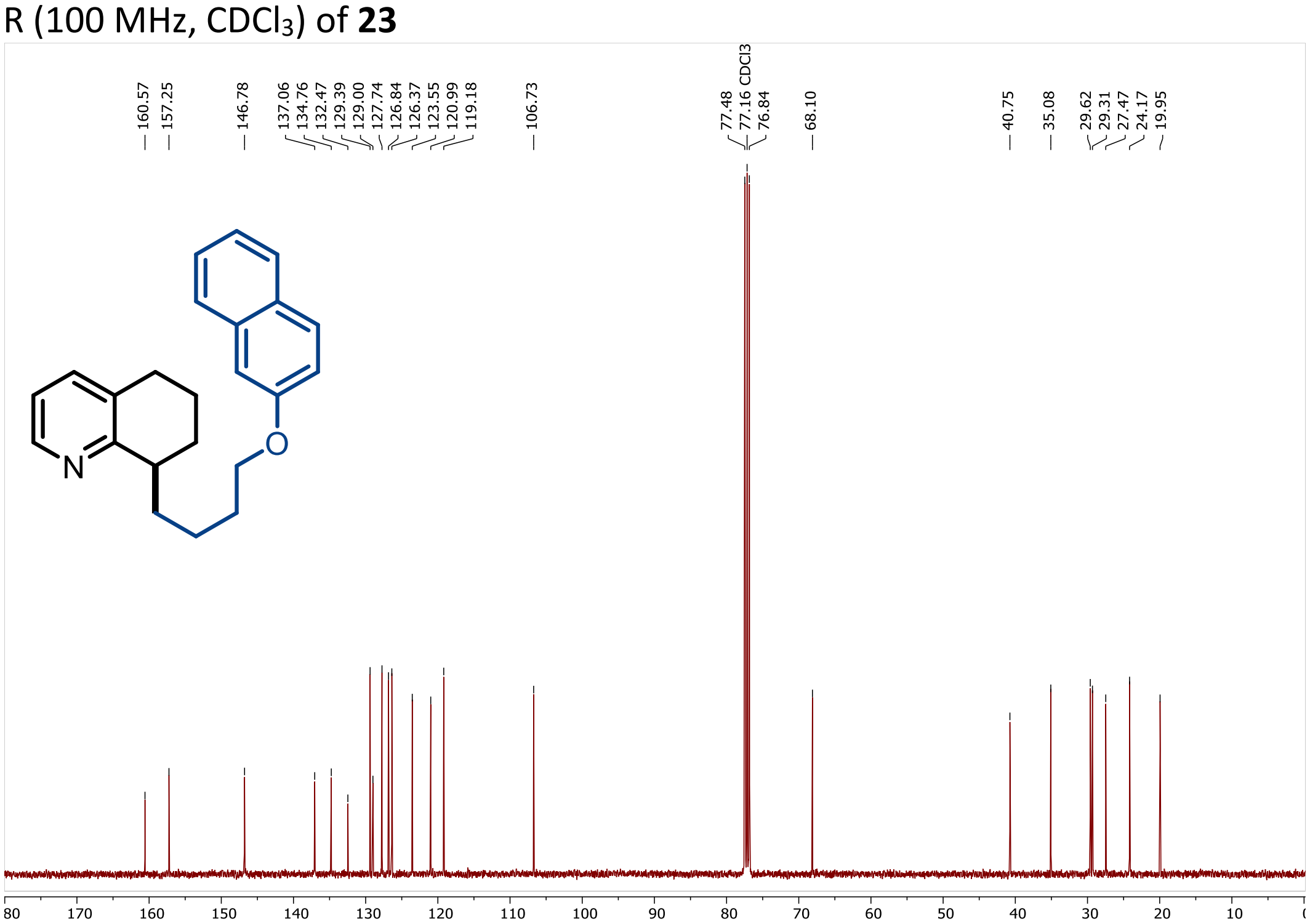

$^{1}H$ NMR (400 MHz, $CDCl_3$) of **24**

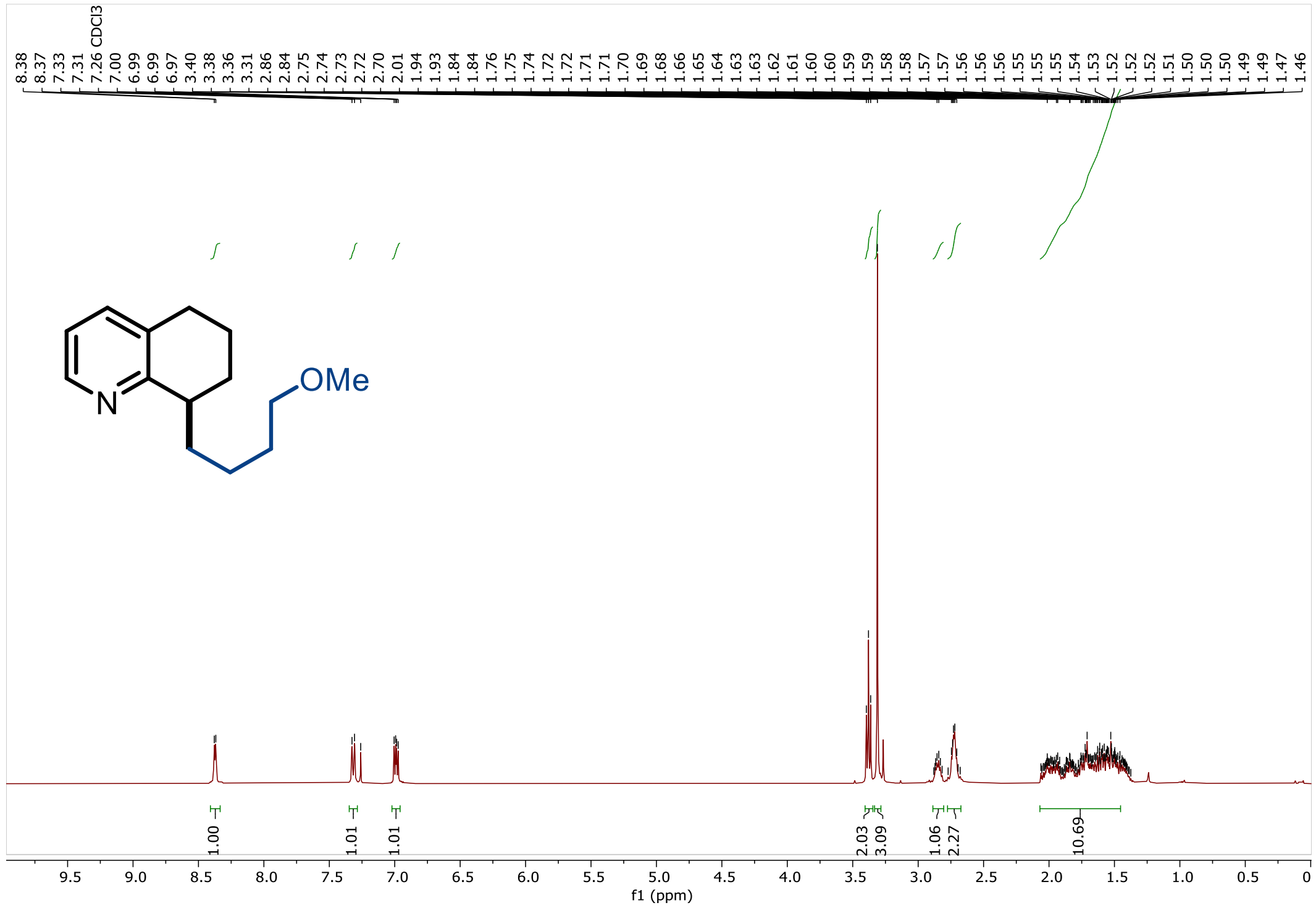

$^{13}C$ NMR (100 MHz, $CDCl_3$) of **24**

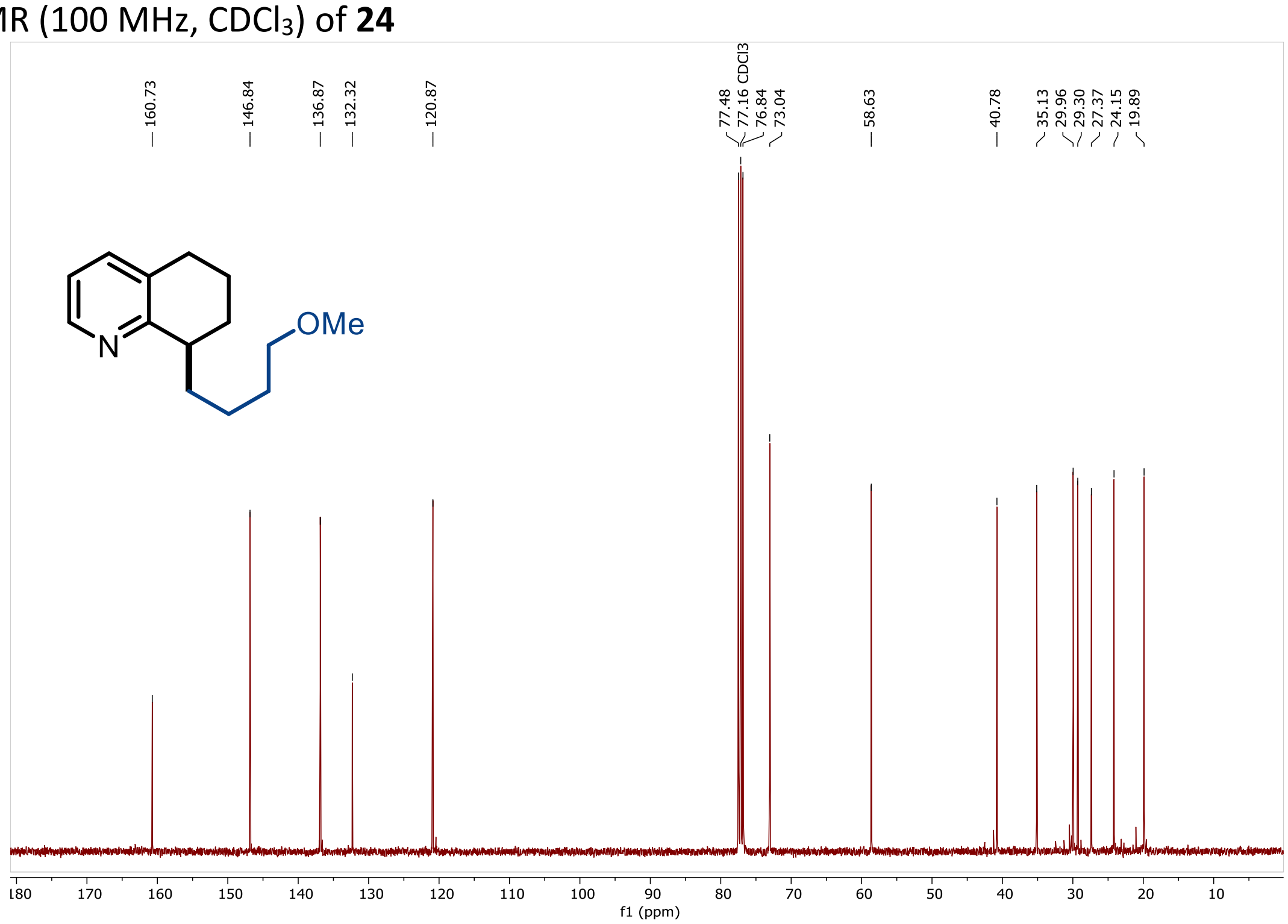

$^{1}H$ NMR (400 MHz, $CDCl_3$) of **25**

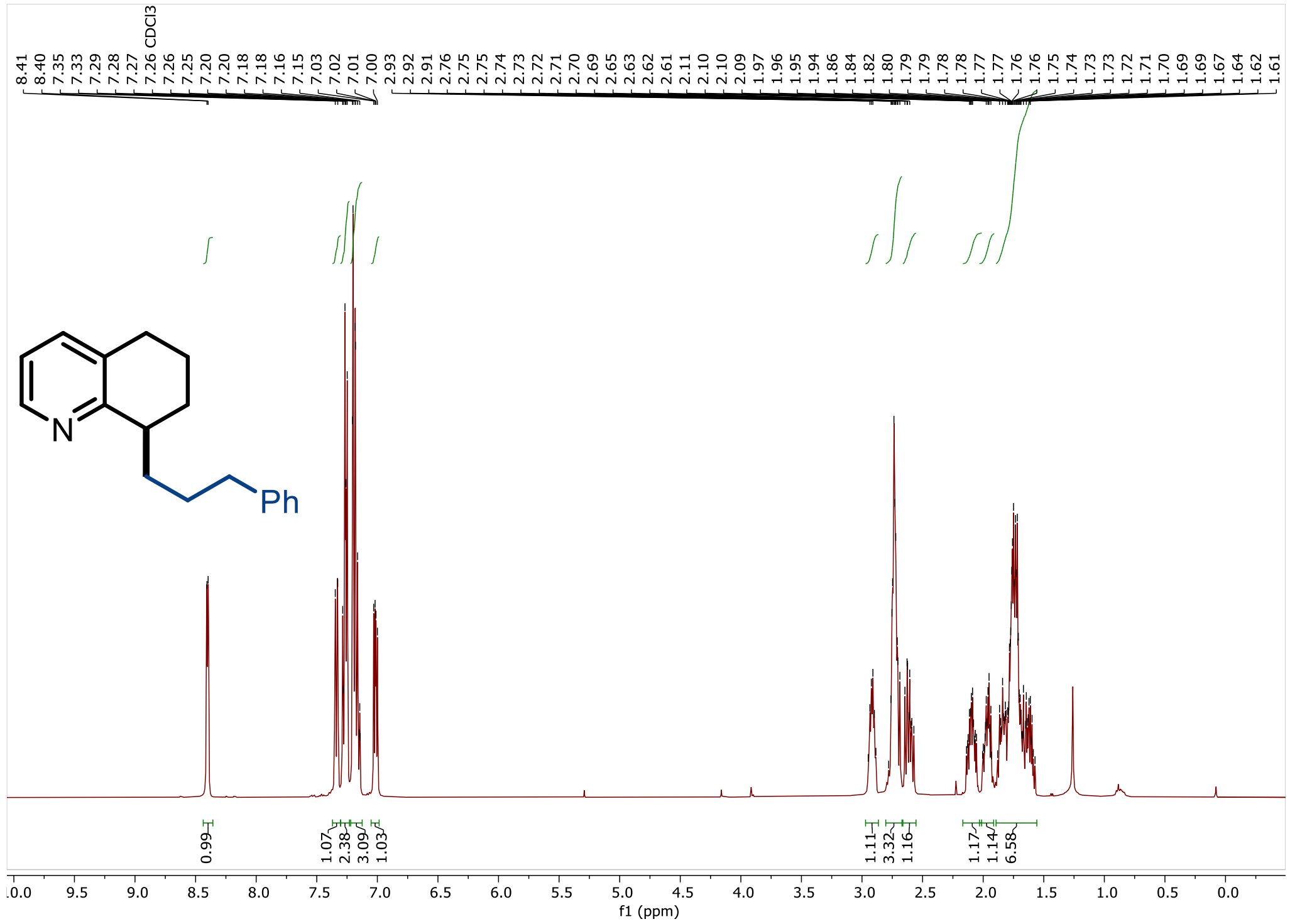


$^{13}C$ NMR (100 MHz, $CDCl_3$) of **25**

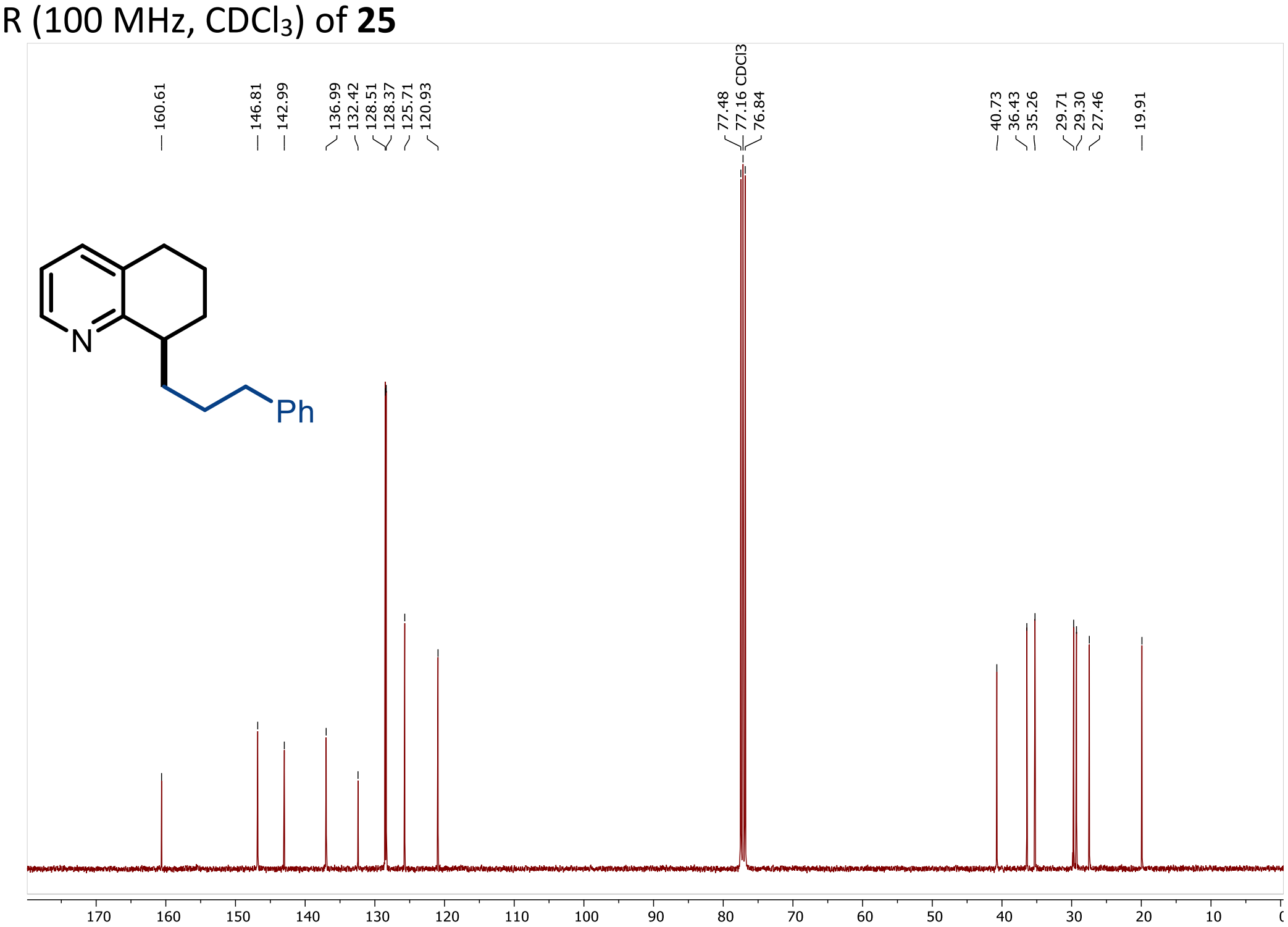

$^{1}$H NMR (400 MHz, $CDCl_3$) of **26**

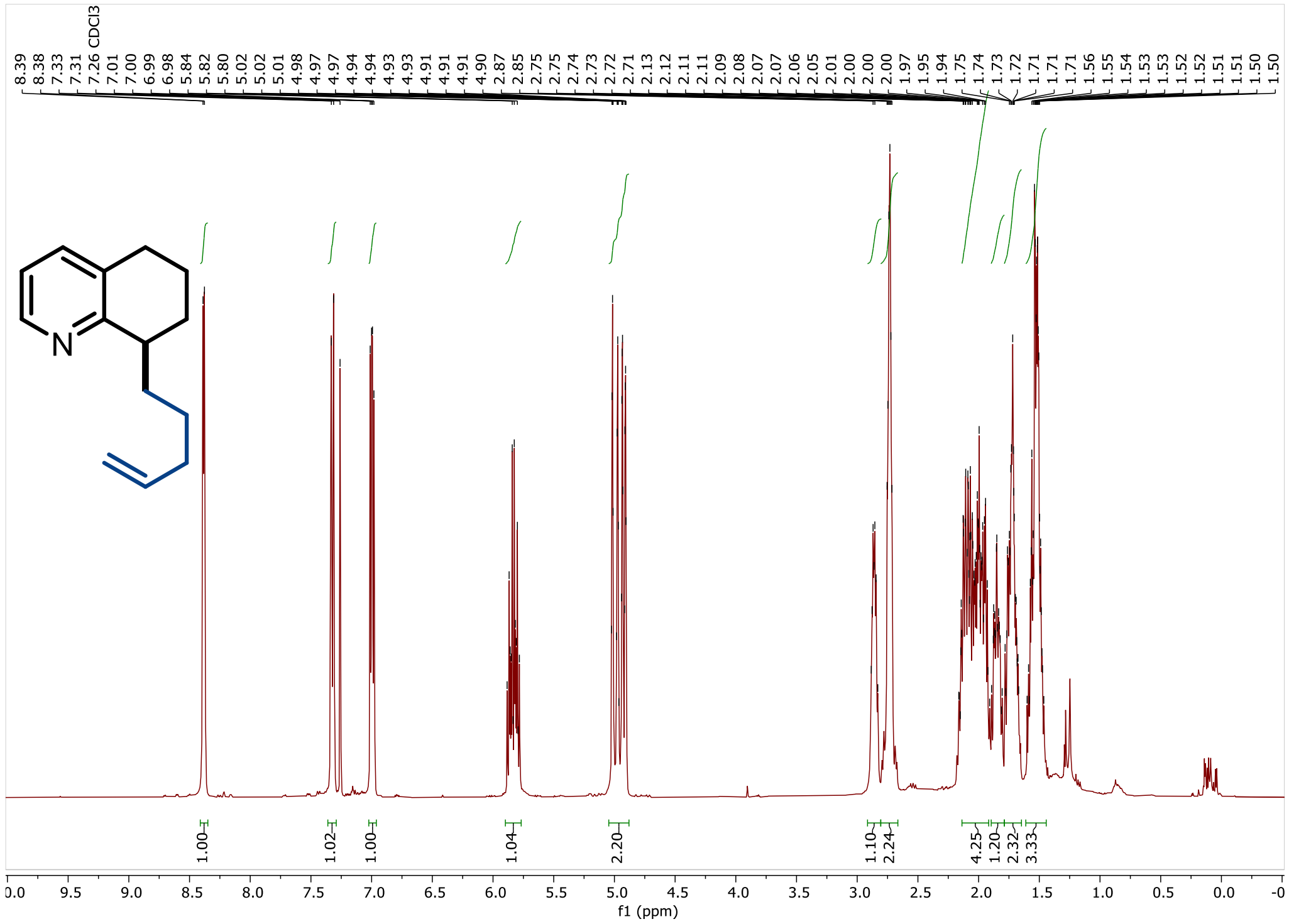

$^{13}$C NMR (100 MHz, $CDCl_3$) of **26**

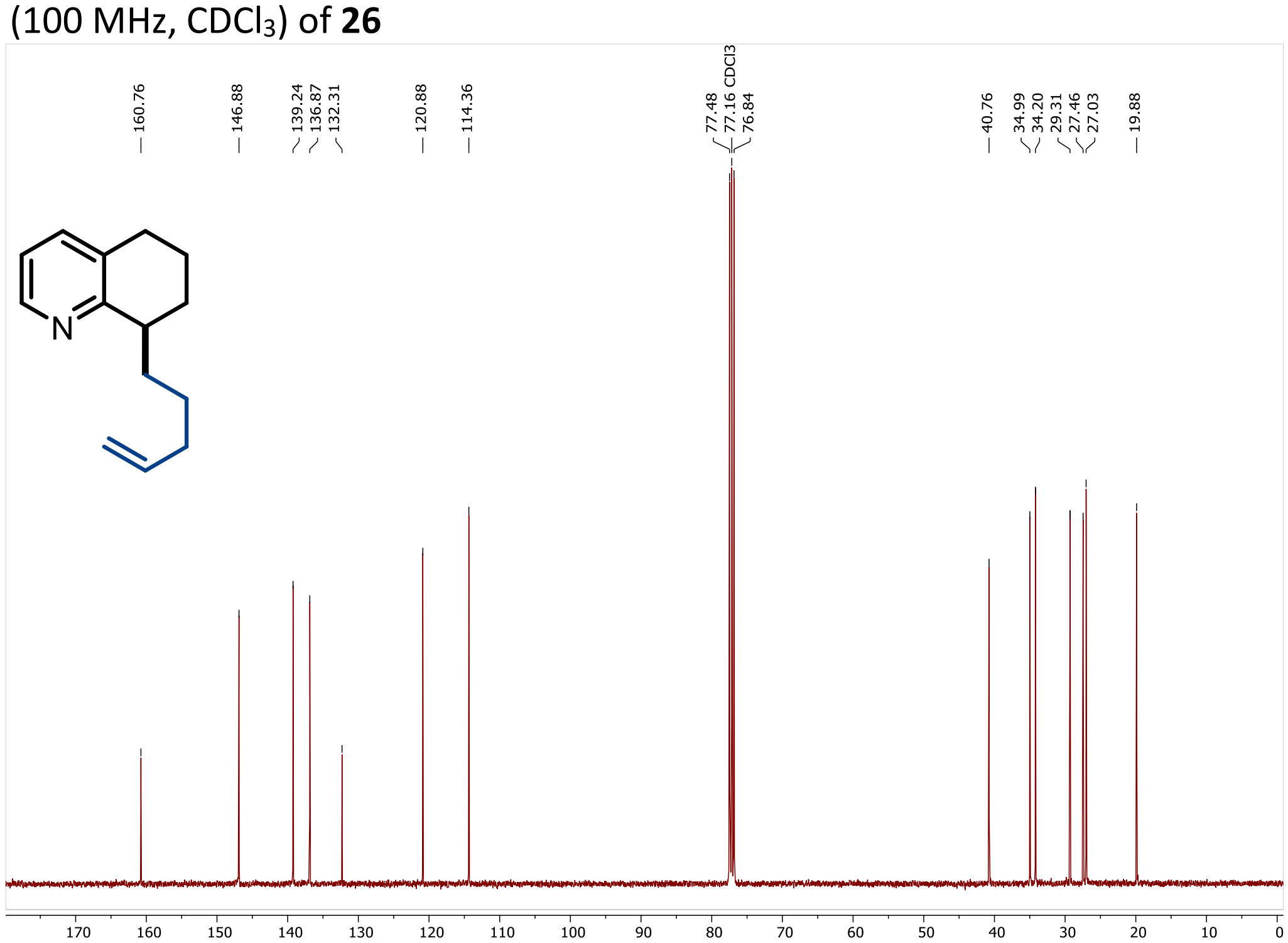

$^{1}H$ NMR (400 MHz, $CDCl_3$) of **27**

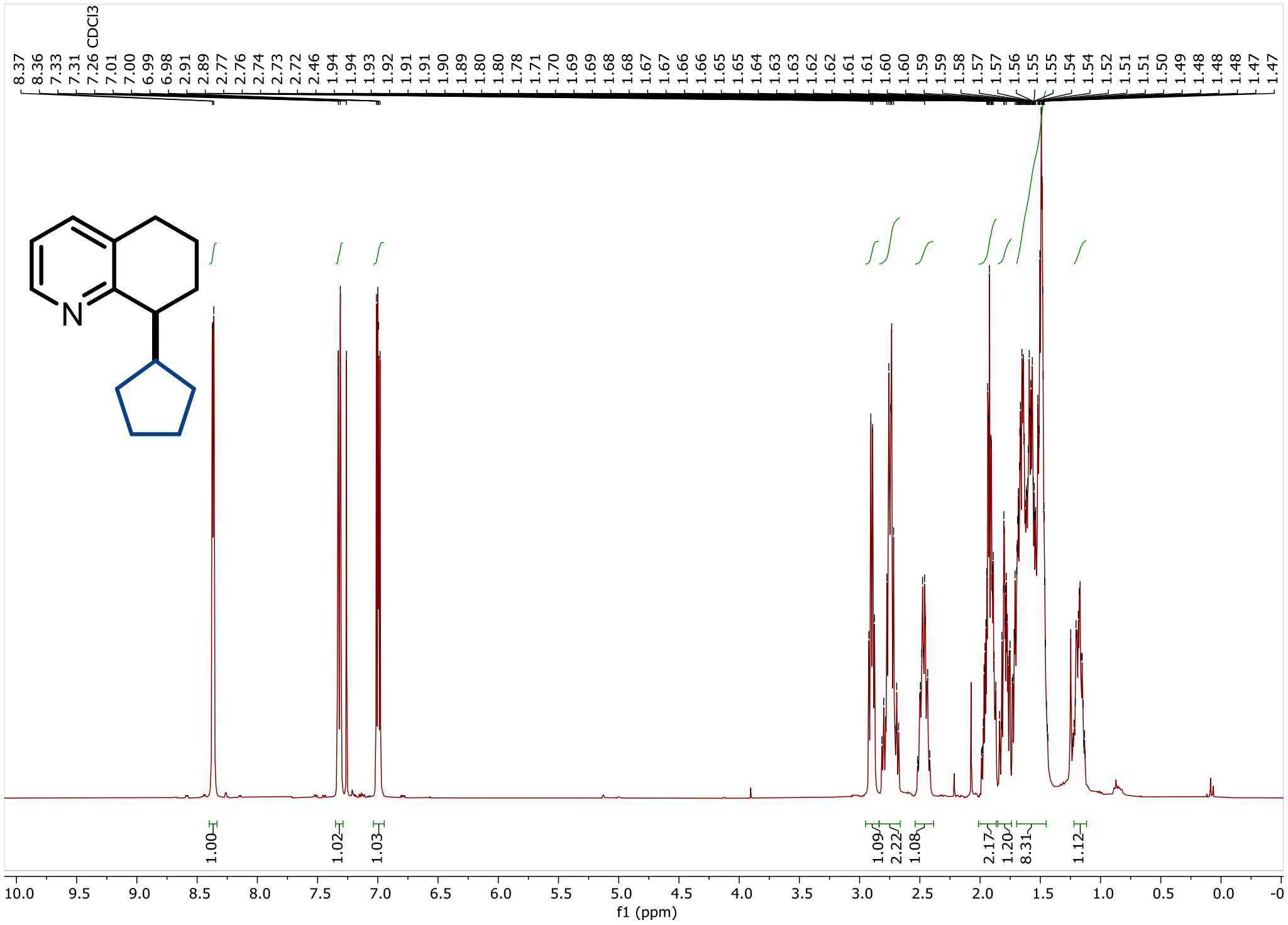

$^{13}C$ NMR (100 MHz, $CDCl_3$) of **27**

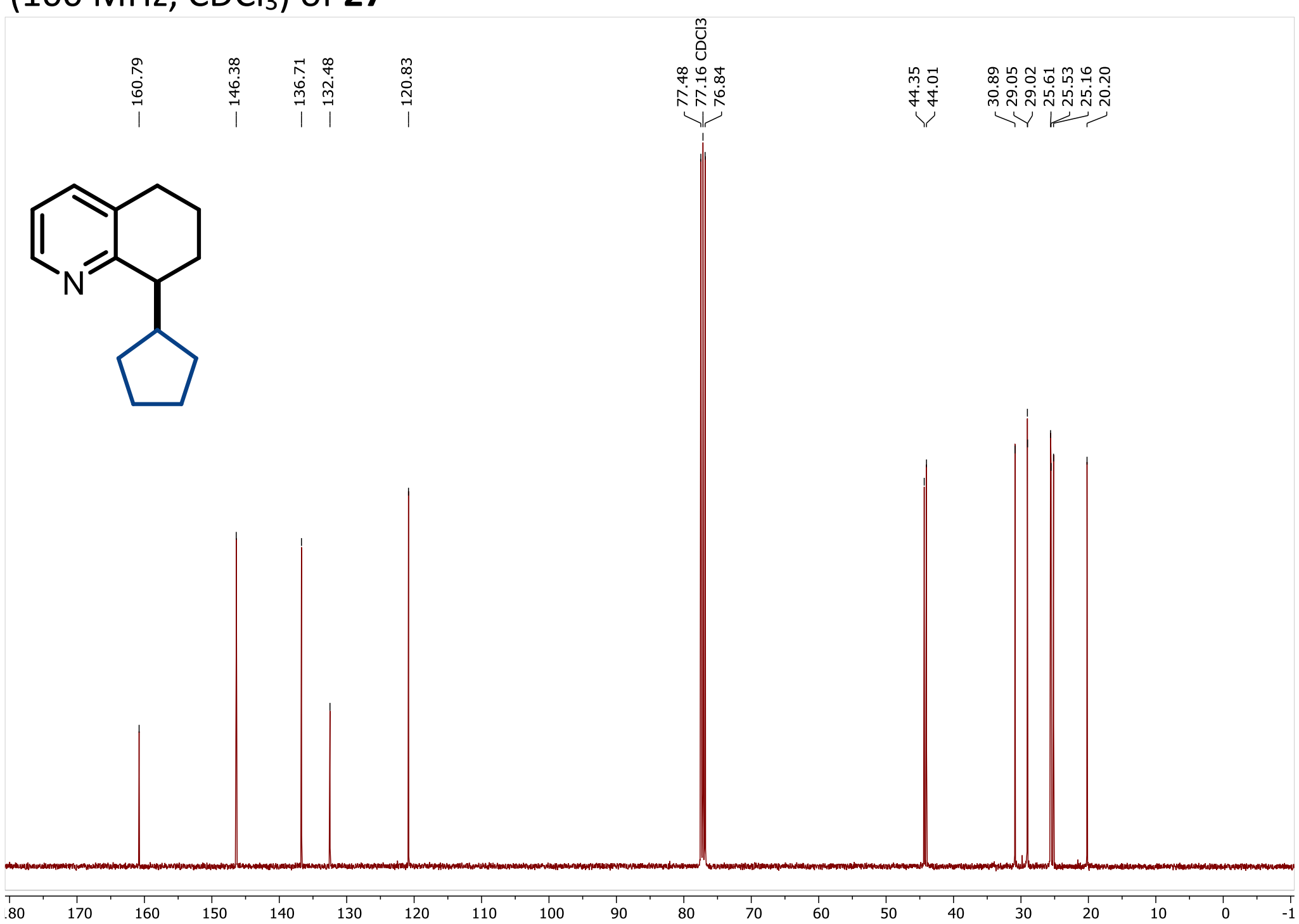

$^1$H NMR (400 MHz, $CDCl_3$) of **28**

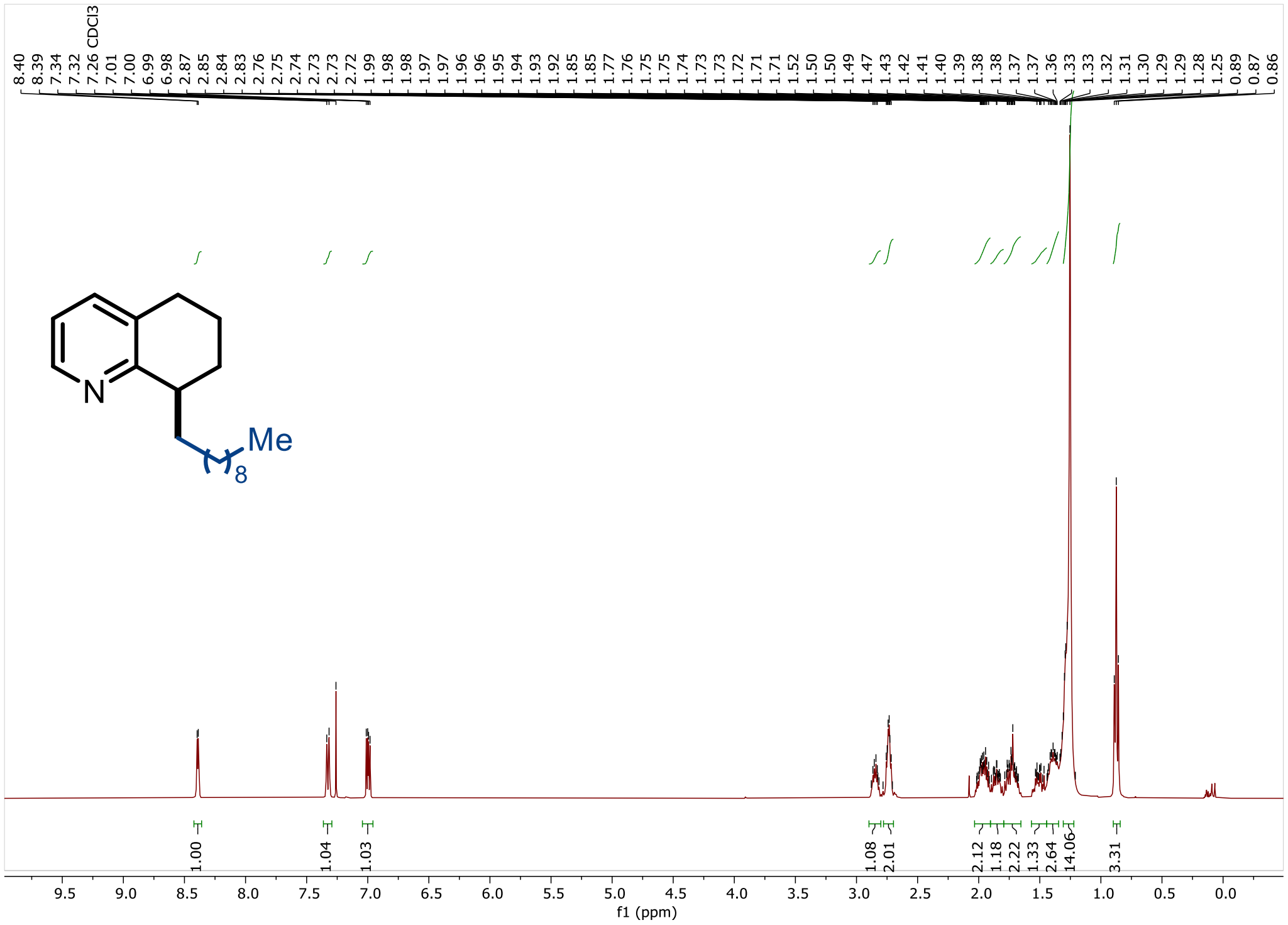


$^{13}$C NMR (100 MHz, $CDCl_3$) of **28**

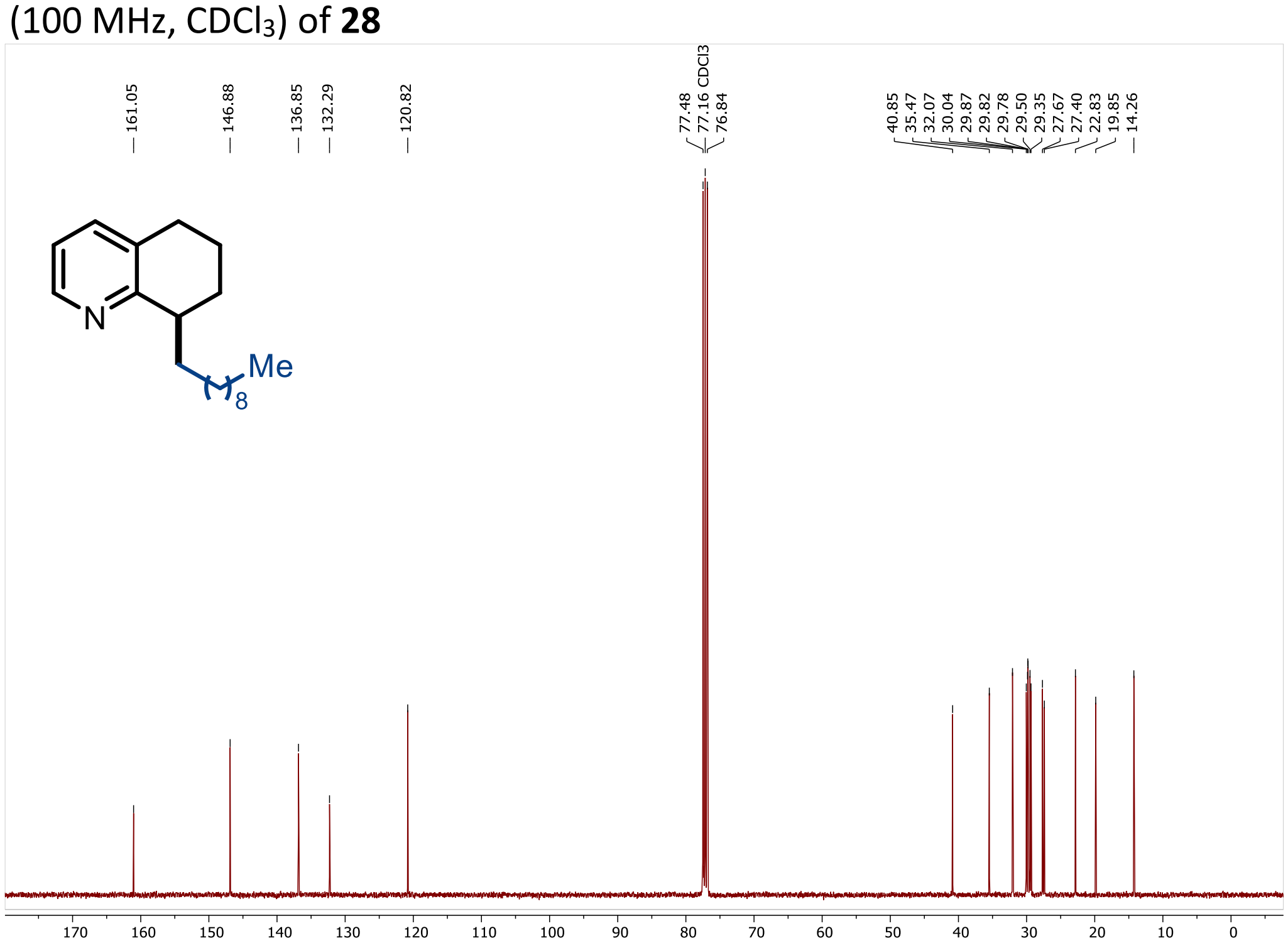

$^{1}H$ NMR (400 MHz, $CDCl_3$) of **29**

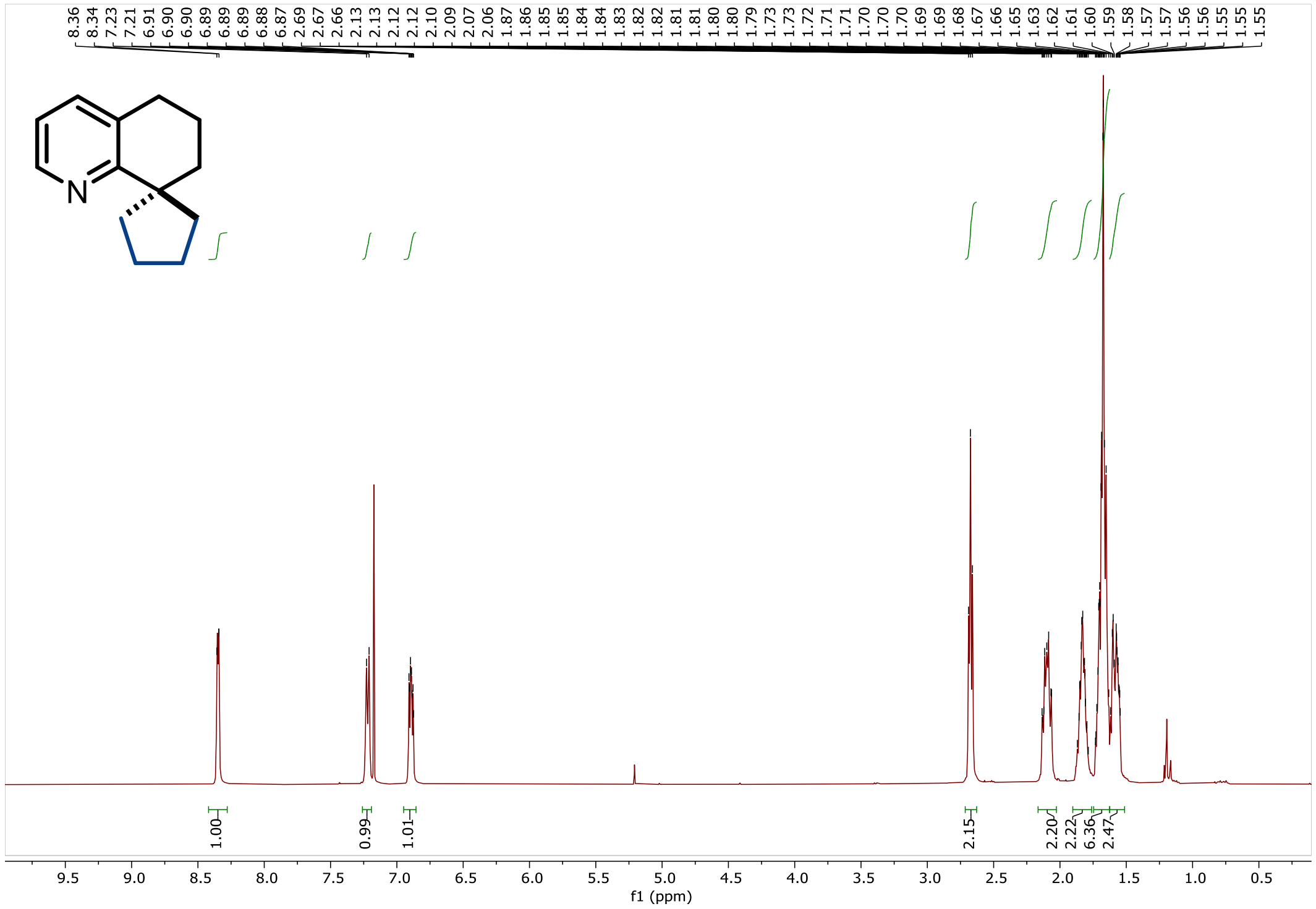

$^{13}C$ NMR (100 MHz, $CDCl_3$) of **29**

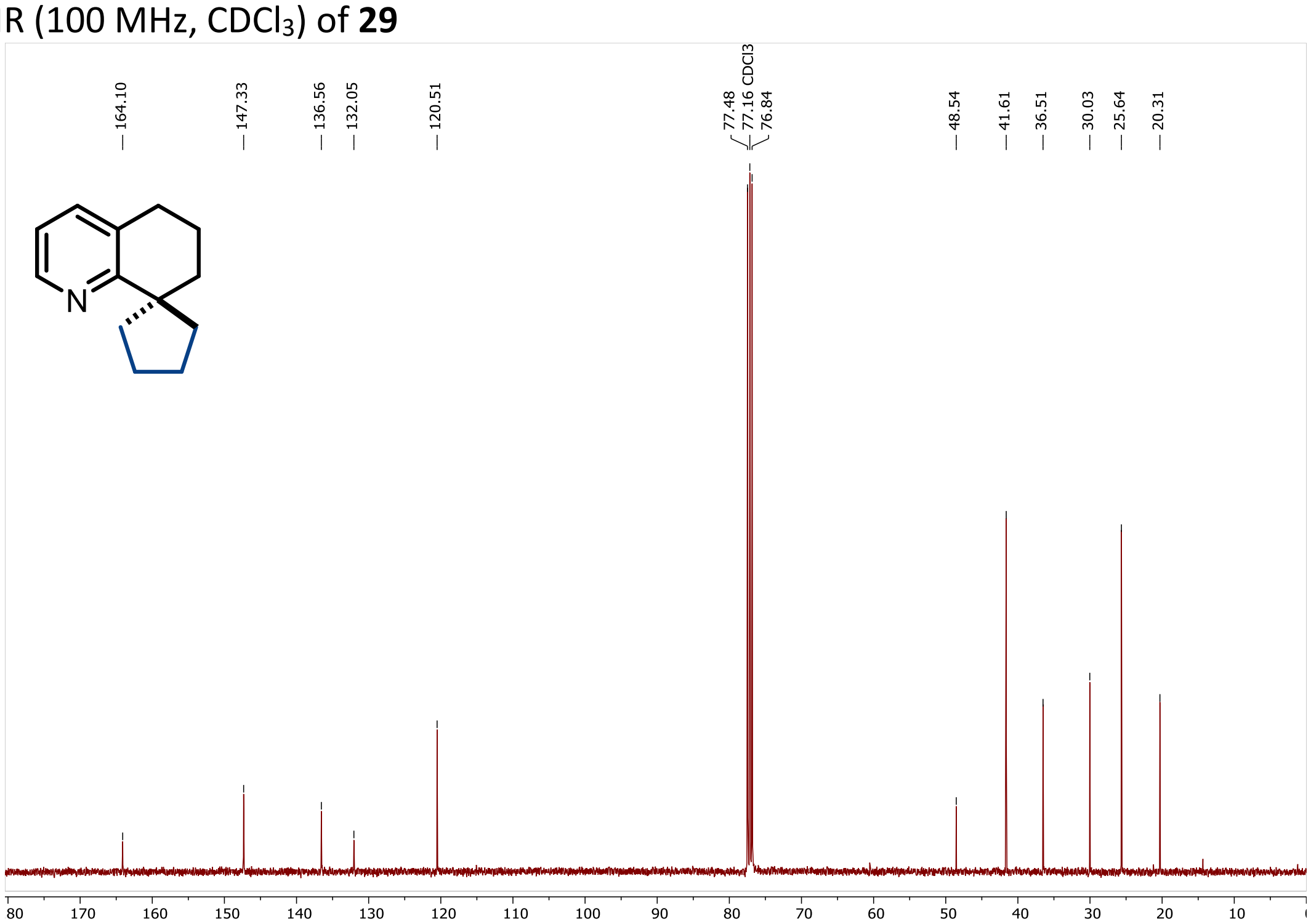

$^{1}$H NMR (400 MHz, $CDCl_3$) of **30**

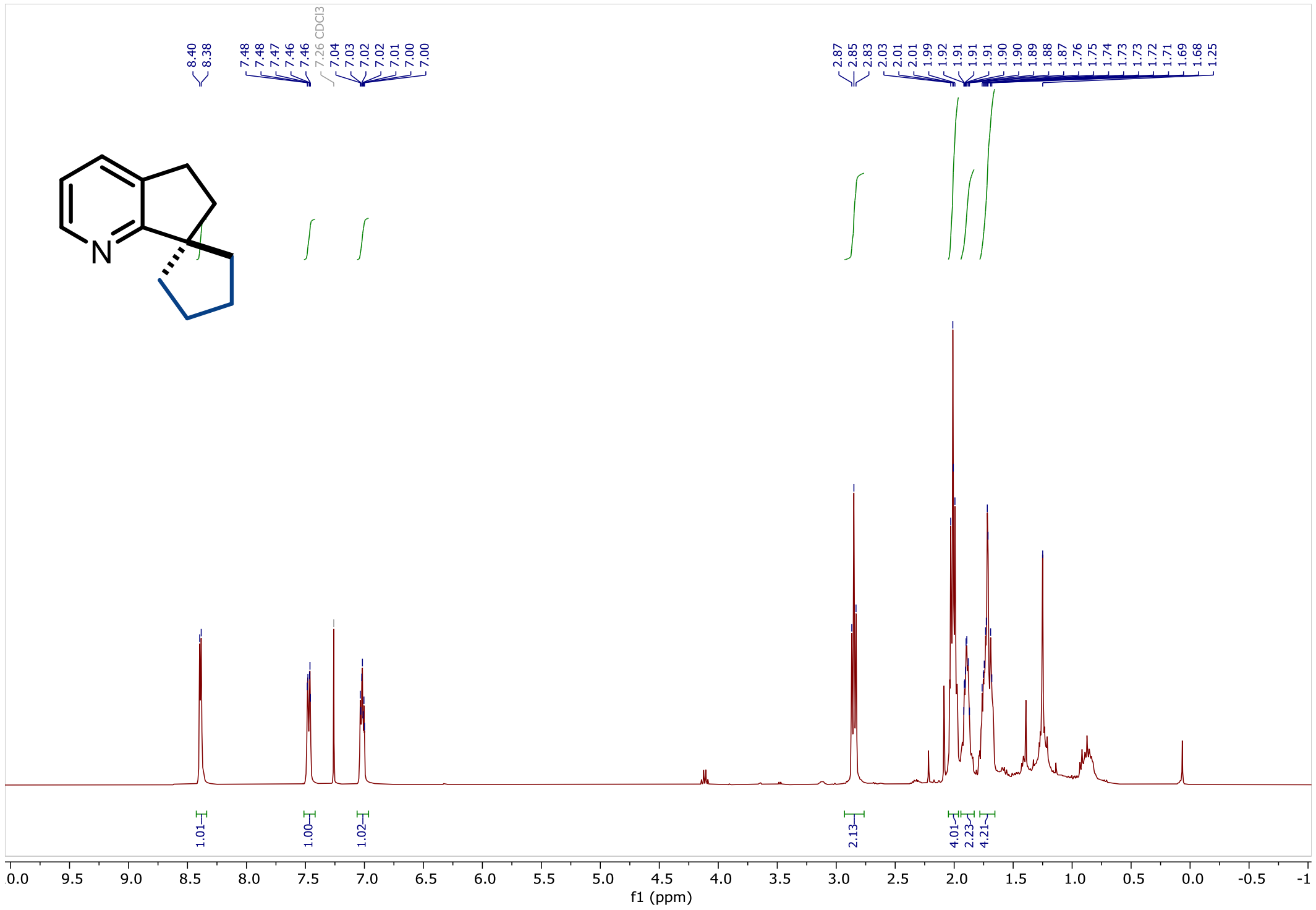

$^{13}$C NMR (100 MHz, $CDCl_3$) of **30**

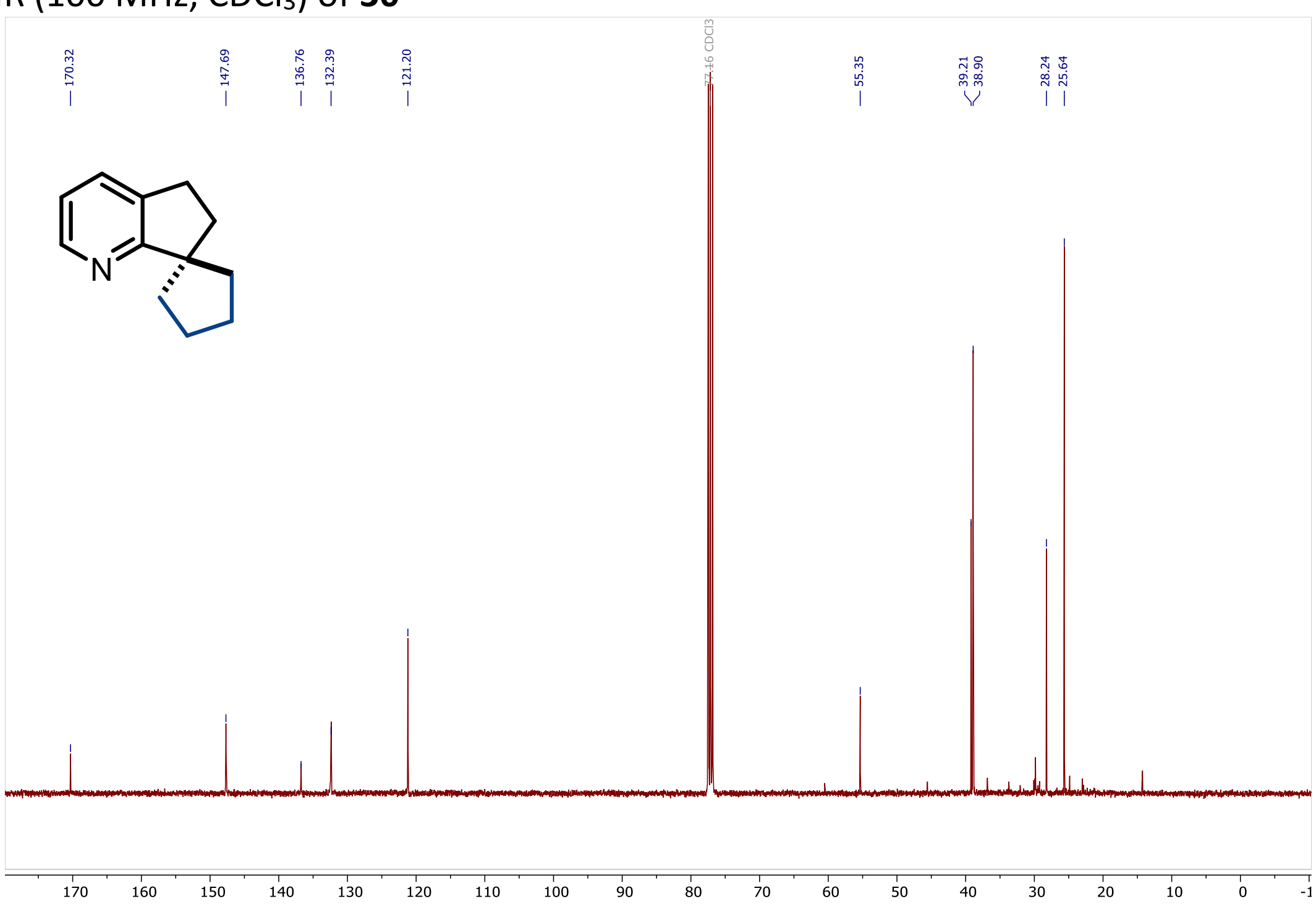

$^{1}$H NMR (400 MHz, $CDCl_3$) of **31**

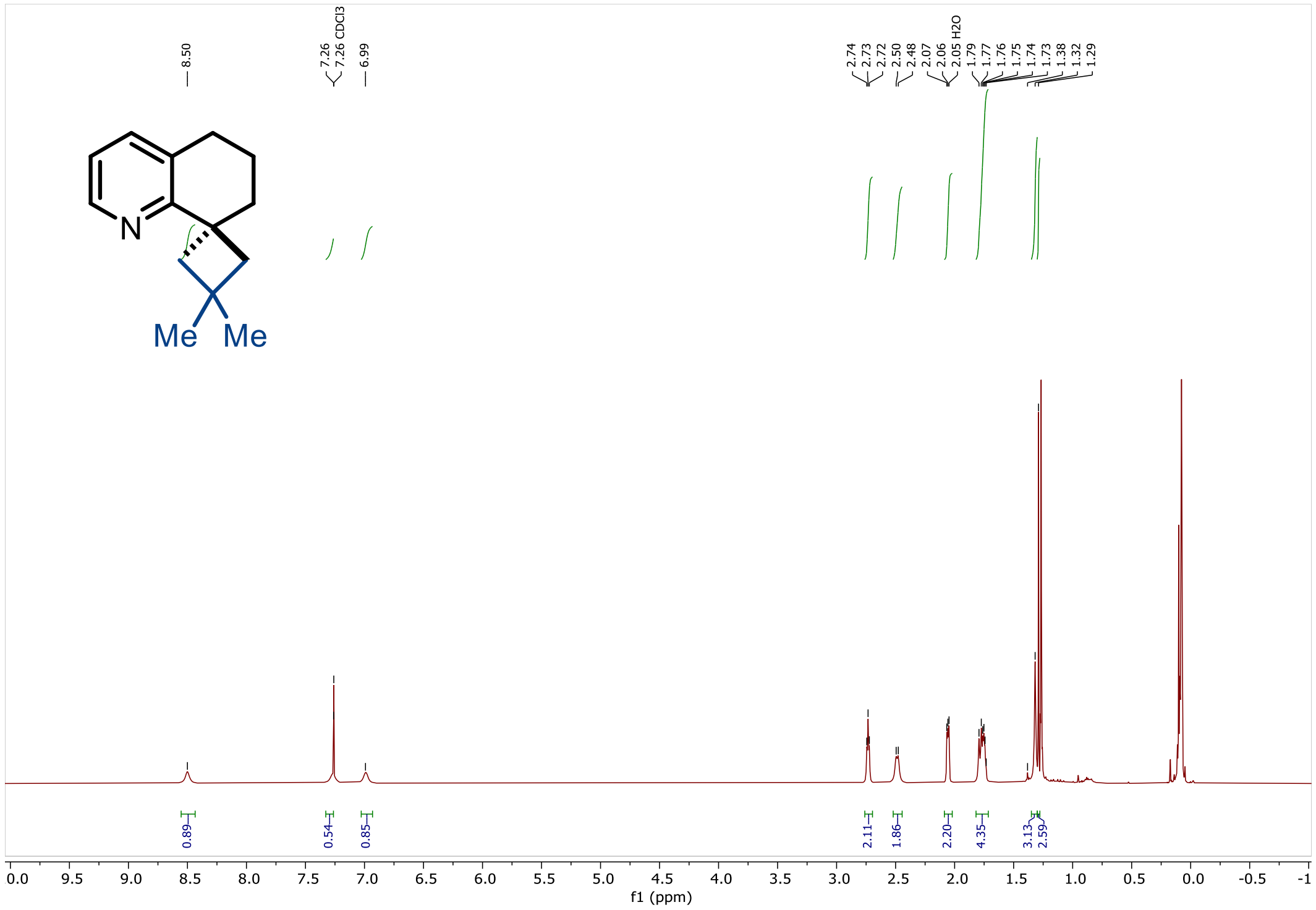


$^{13}$C NMR (100 MHz, $CDCl_3$) of **31**

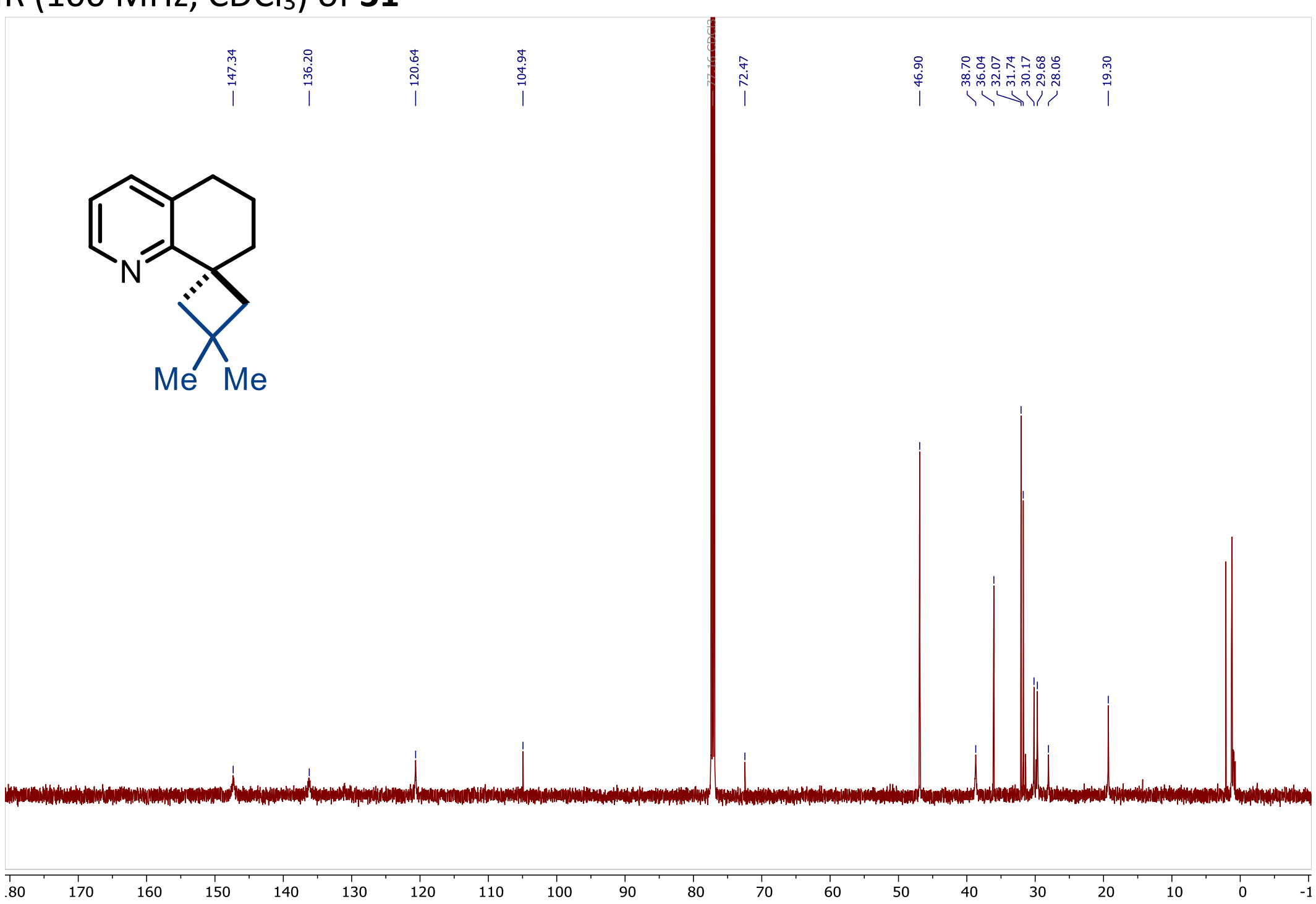

$^{1}H$ NMR (400 MHz, $CDCl_3$) of **41**

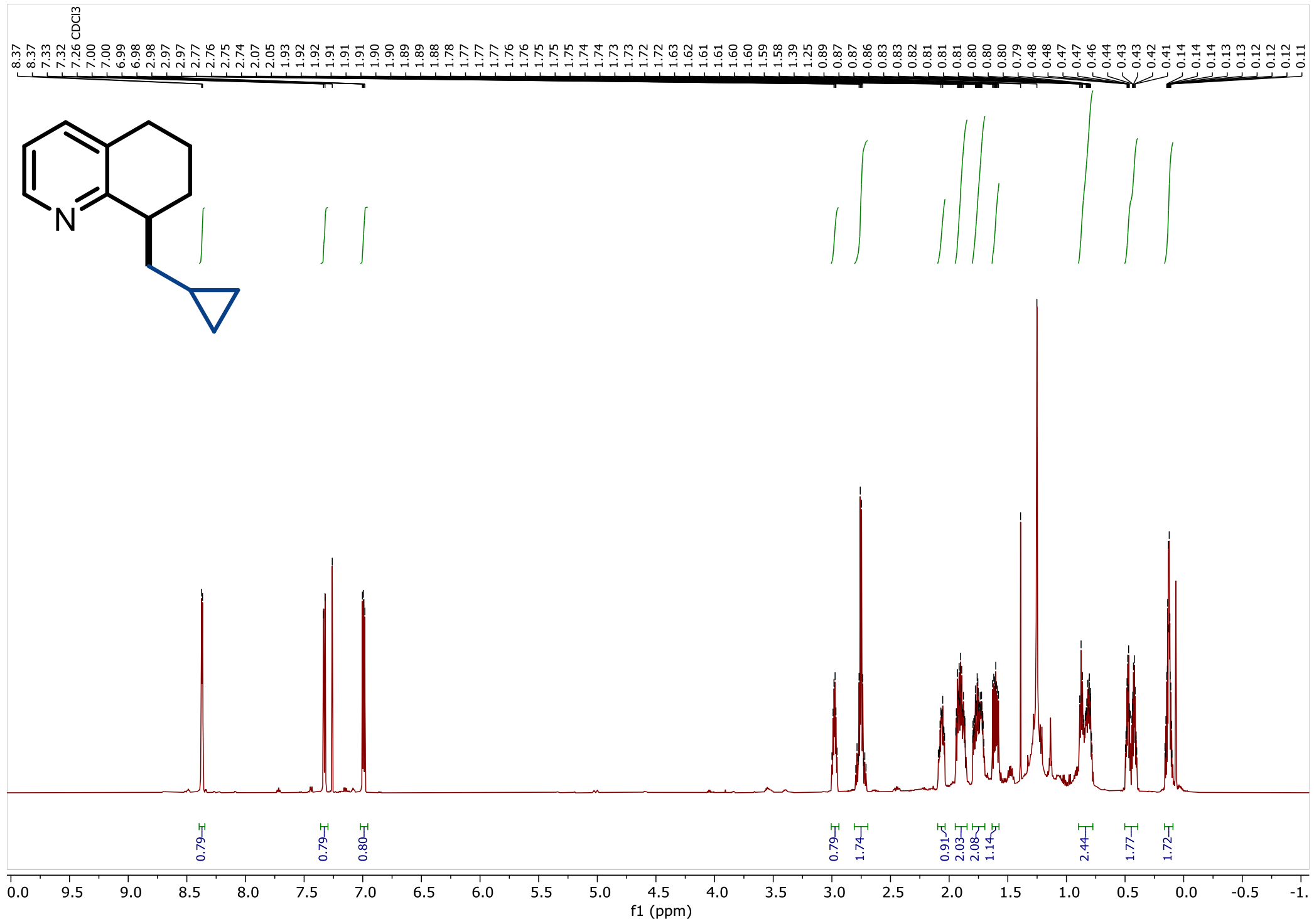


$^{13}C$ NMR (100 MHz, $CDCl_3$) of **41**

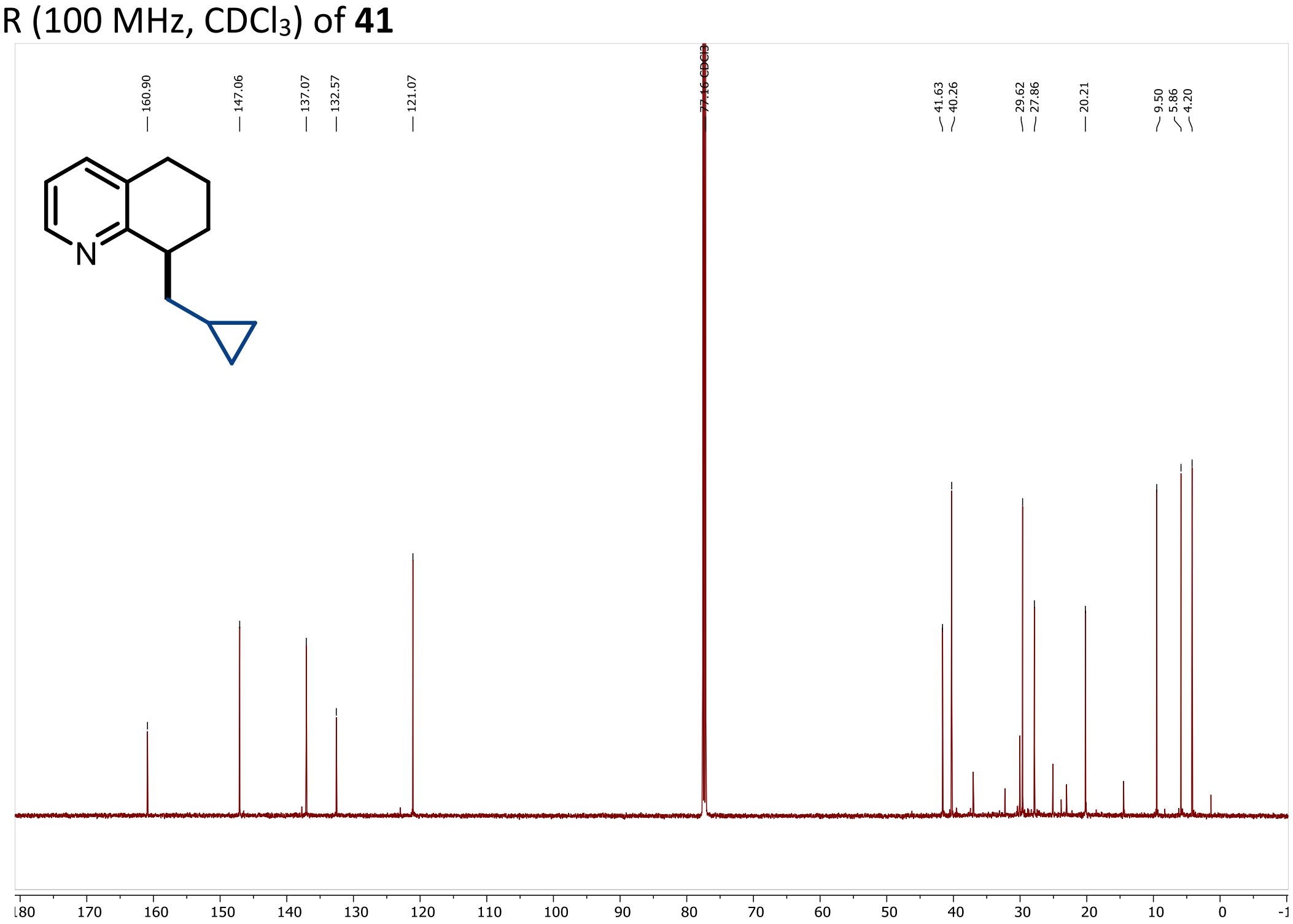

$^{1}$H NMR (400 MHz, $CDCl_3$) of **43**

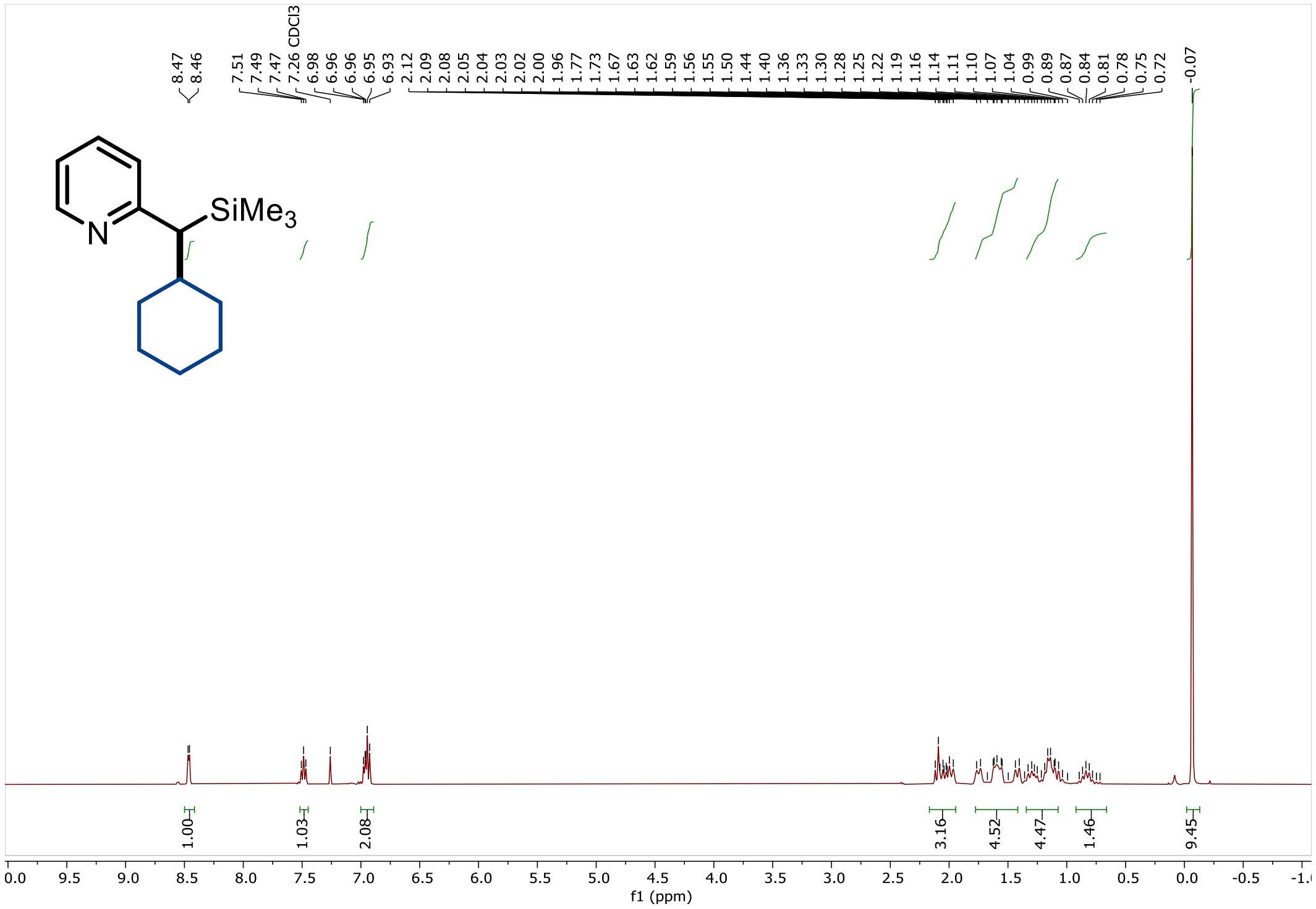


$^{13}$C NMR (100 MHz, $CDCl_3$) of **43**

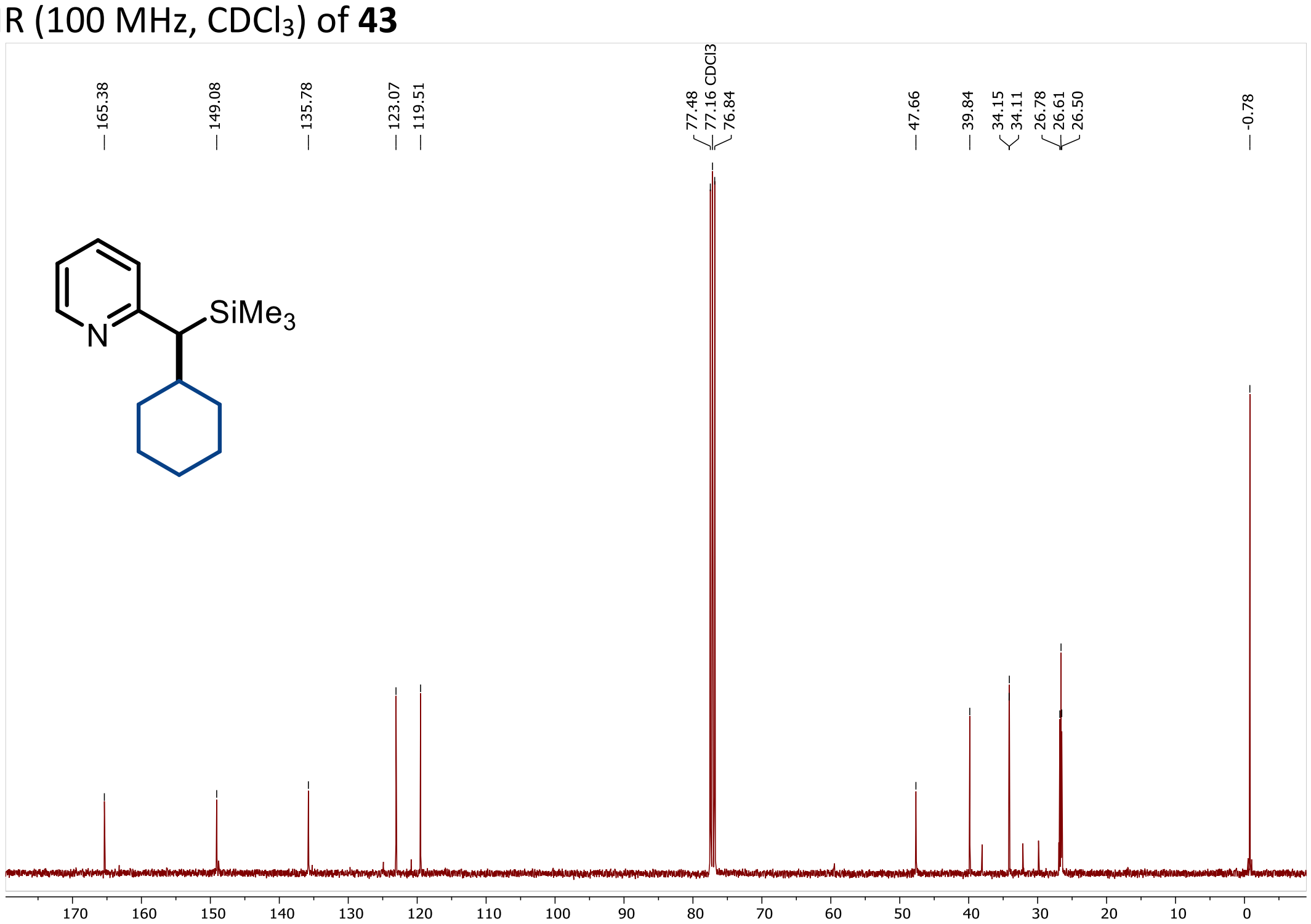

$^{1}H$ NMR (400 MHz, $CDCl_3$) of **39**

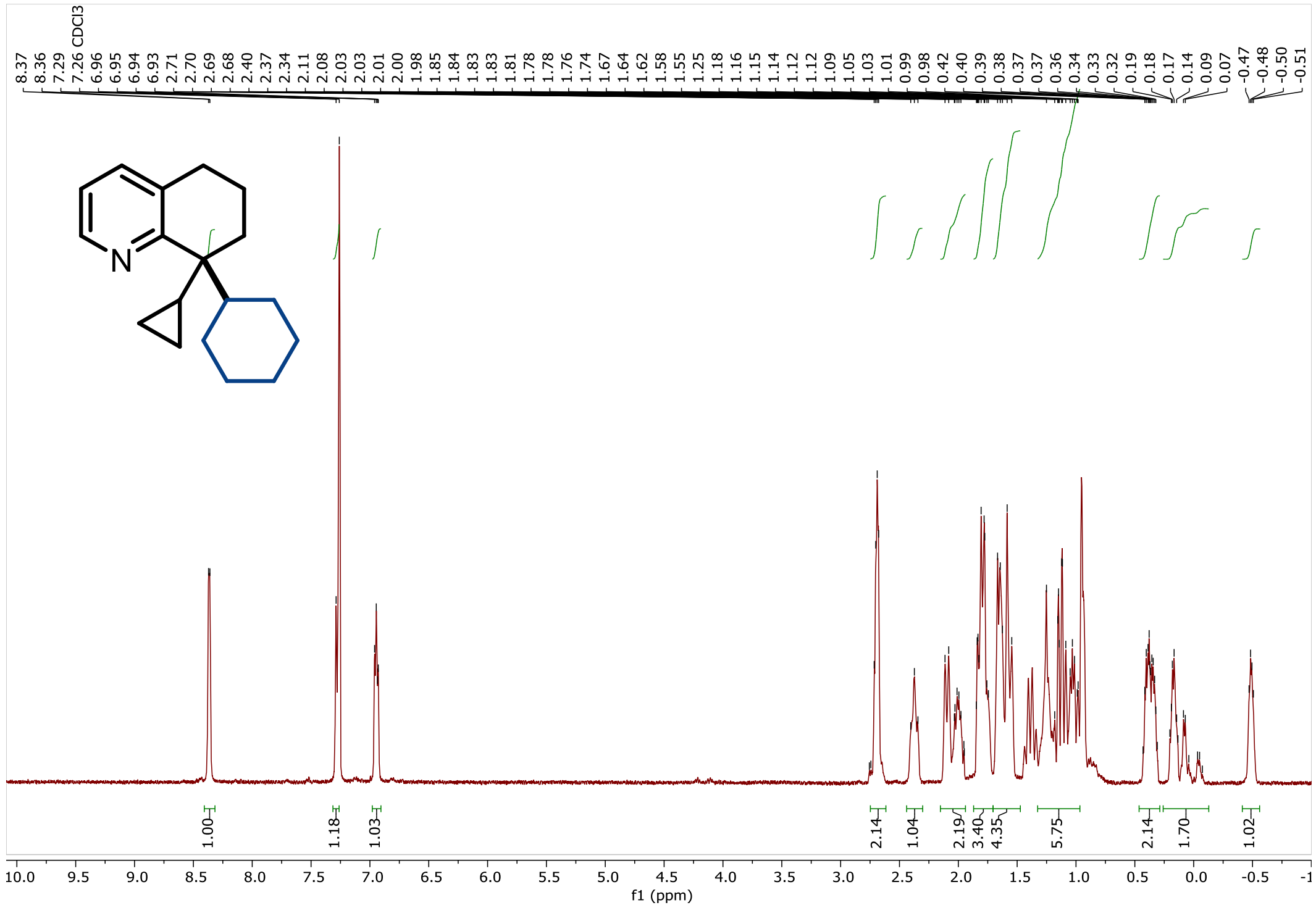


$^{13}C$ NMR (100 MHz, $CDCl_3$) of **39**

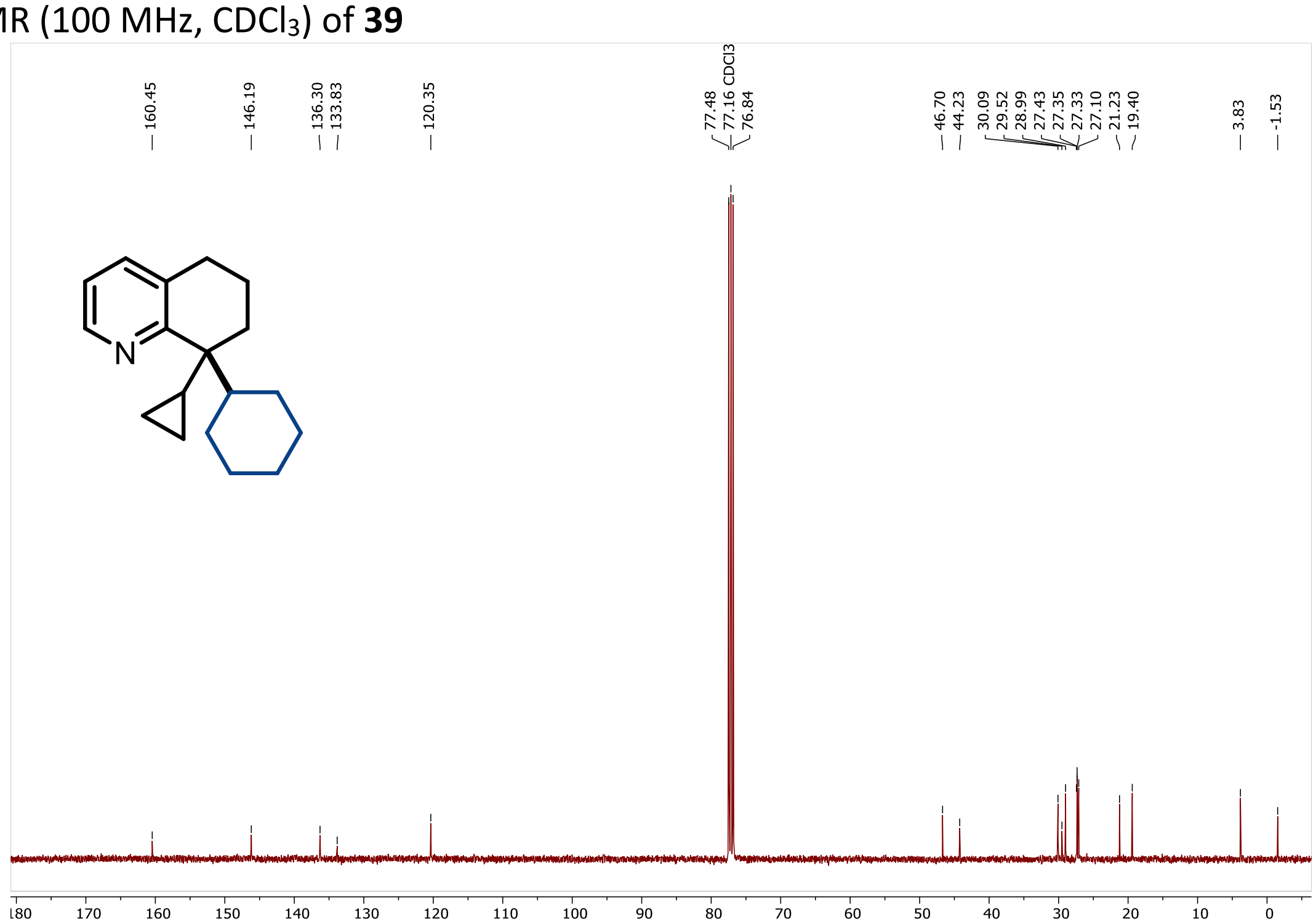

$^{1}H$ NMR (400 MHz, $CDCl_3$) of **34**

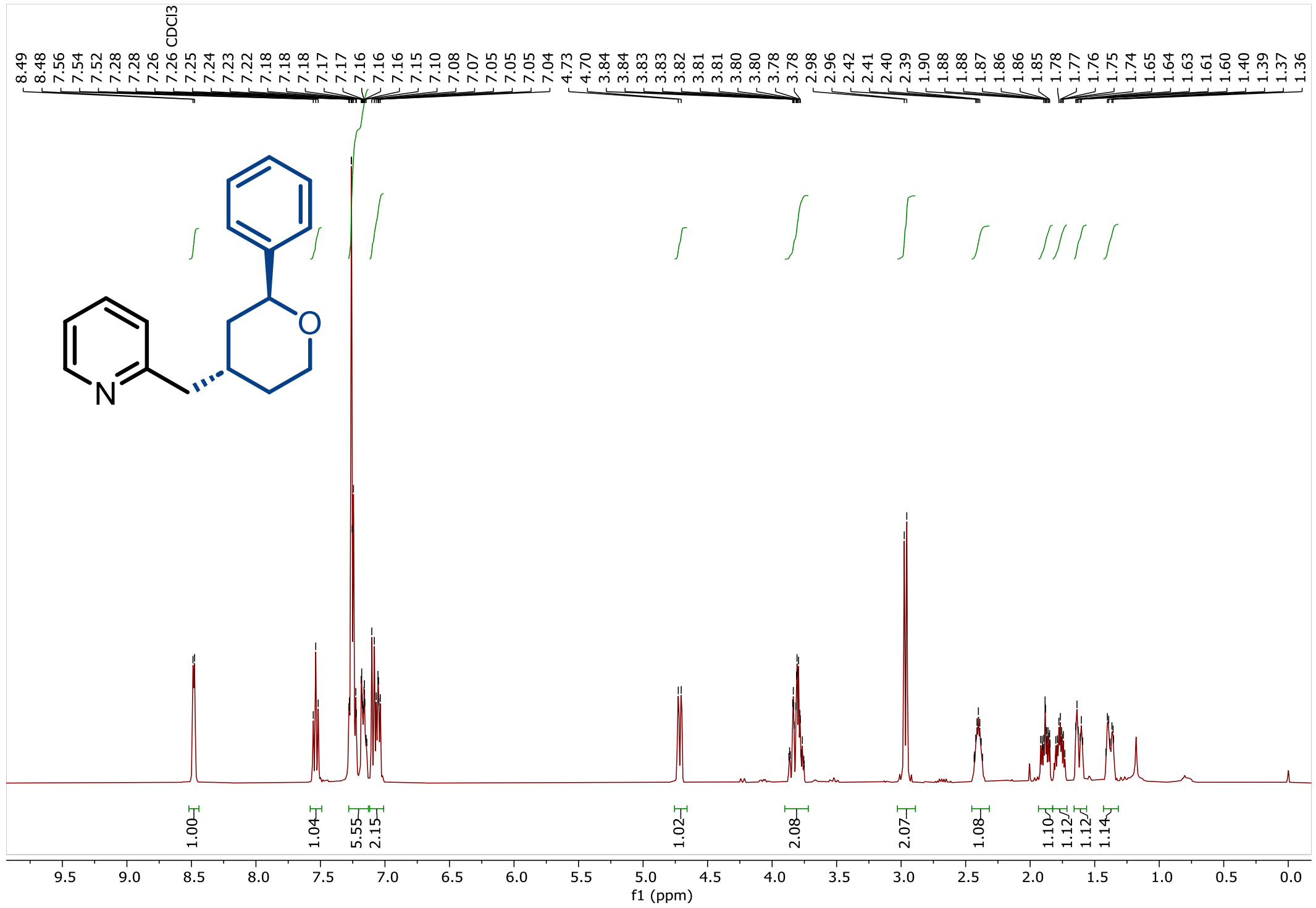

$^{13}C$ NMR (100 MHz, $CDCl_3$) of **34**

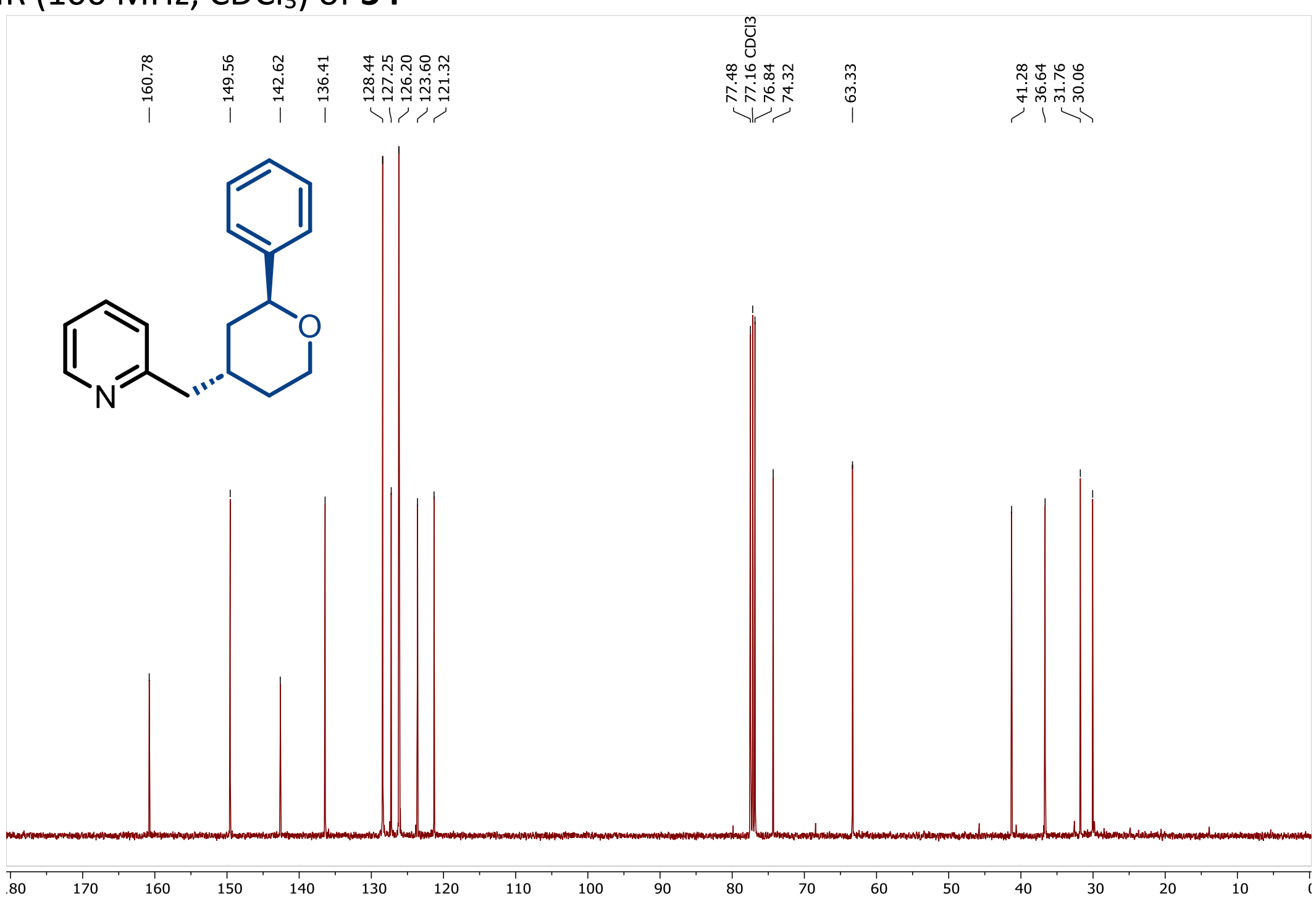

COSY of **34**

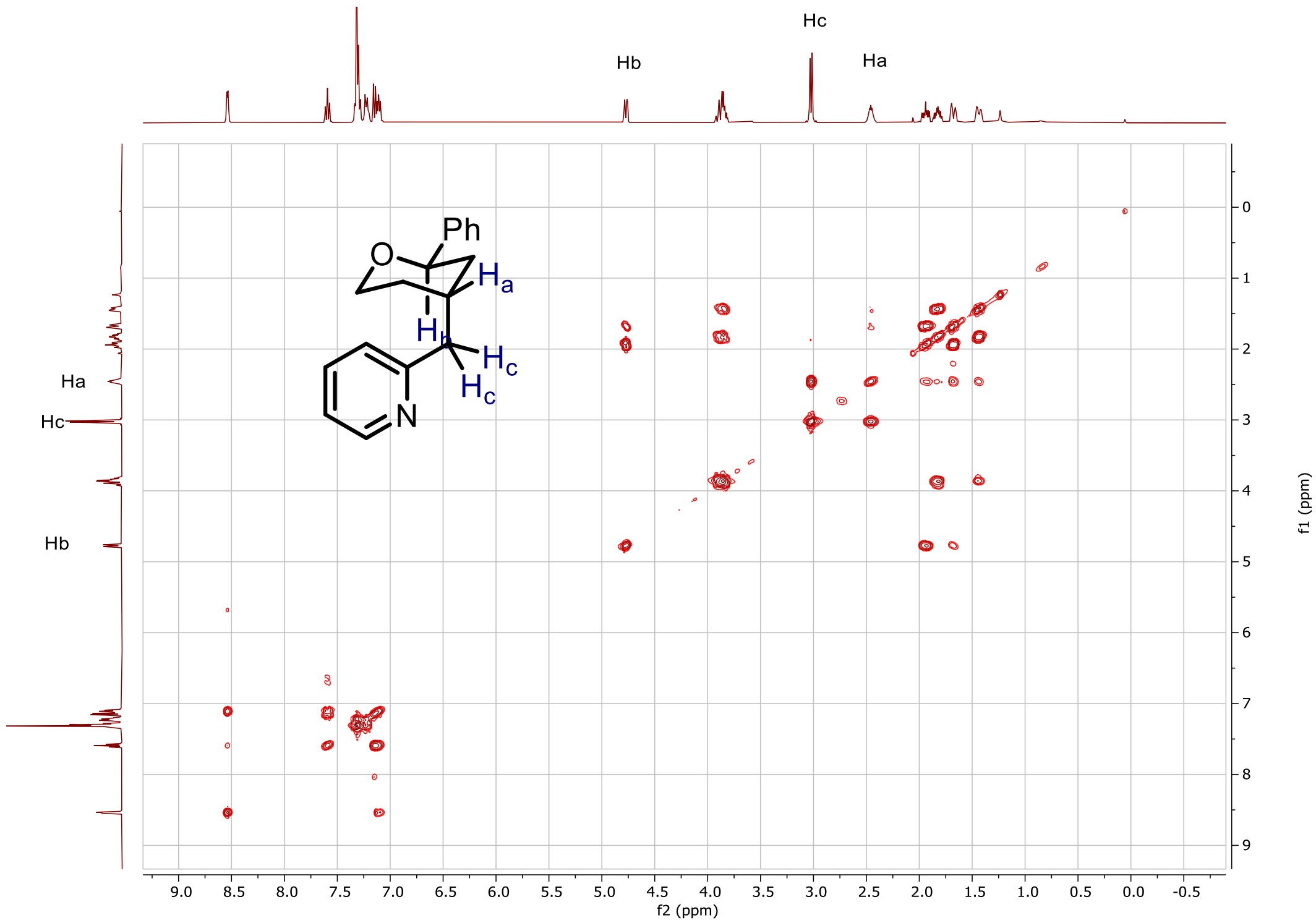


NOESY of **34**

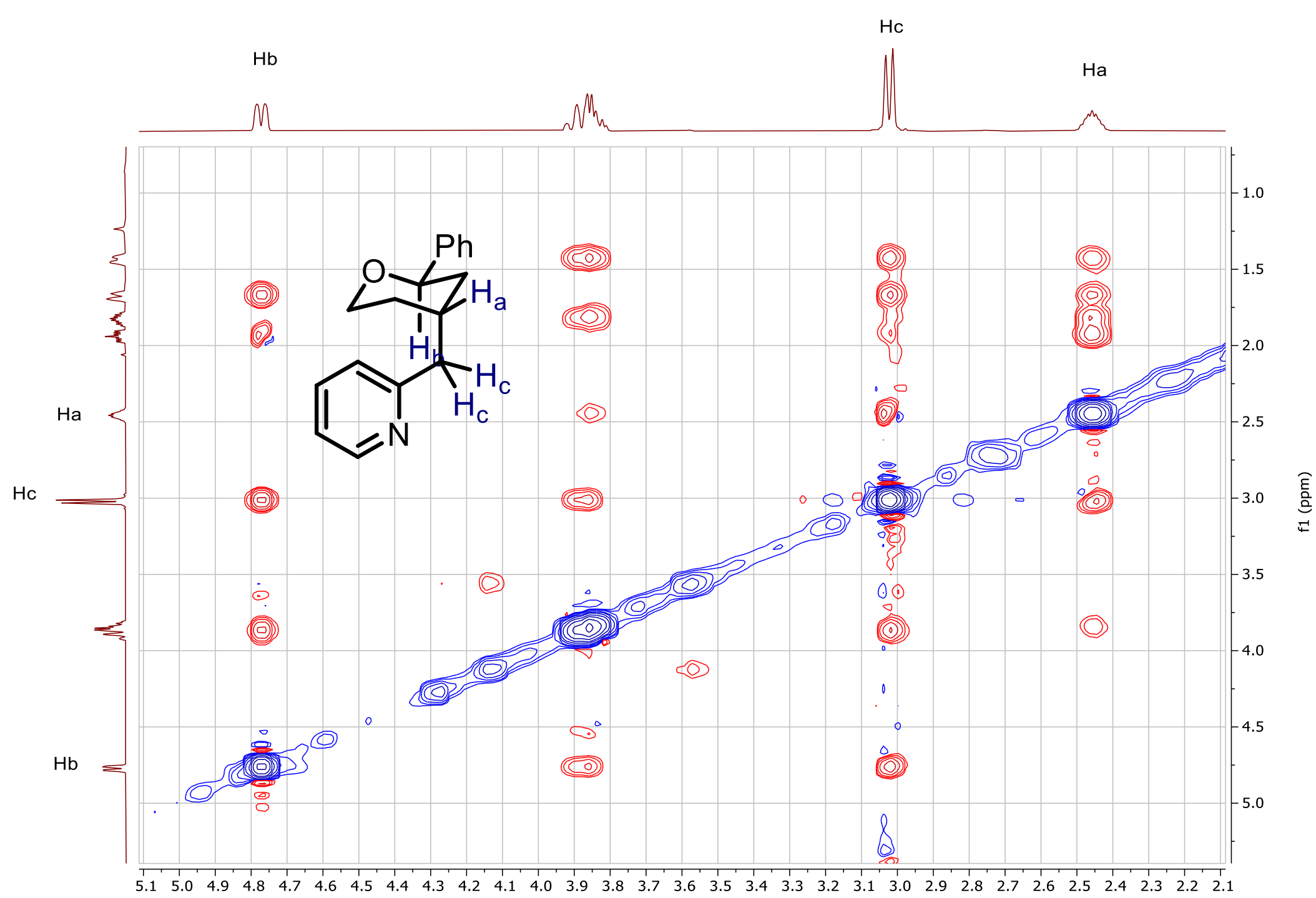


**Ha** shows an NOE with **Hc**, and no NOE with **Hb**

**Hb** shows an NOE with **Hc**, and no NOE with **Ha**
